# Structural Optimization of BIPPO Analogs as Potent Antimalarials

**DOI:** 10.3390/molecules28134939

**Published:** 2023-06-23

**Authors:** Yang Zheng, An Matheeussen, Louis Maes, Guy Caljon, Geert Jan Sterk, Rob Leurs

**Affiliations:** 1 Division of Medicinal Chemistry, Amsterdam Institute for Molecules, Medicines and Systems, Faculty of Science, VU University Amsterdam, De Boelelaan 1083, 1081 HV Amsterdam, The Netherlands; 2 Laboratory of Microbiology, Parasitology and Hygiene, University of Antwerp, 2610 Wilrijk, Belgium

**Keywords:** antimalarial, BIPPO analogs, structural optimization

## Abstract

Malaria continues to pose a significant health threat, causing thousands of deaths each year. The limited availability of vaccines and medications, combined with the emergence of drug resistance, further complicates the fight against this disease. In this study, we aimed to enhance the antimalarial potency of the previously reported hit compound BIPPO (pIC_50_ 5.9). Through systematic modification of pyrazolopyrimidinone analogs, we discovered the promising analog **30** (NPD-3547), which exhibited approximately one log unit higher in vitro potency (pIC_50_ 6.8) against *Plasmodium falciparum*. Furthermore, we identified several other BIPPO analogs (**23**, **28**, **29** and **47a**) with potent antimalarial activity (pIC_50_ > 6.0) and favorable metabolic stability in mouse liver microsomes. These compounds can serve as new tools for further optimization towards the development of potential candidates for antimalarial studies.

## 1. Introduction

Malaria is a mosquito-transmitted parasitic disease caused by *Plasmodium* spp. Although the world has witnessed a decrease in reported malaria cases, the current situation is still worrying [1]. In 2019, there was an estimated number of 229 million infections with 409,000 deaths globally [2]. With a substantial amount (20%) of malaria research funding invested in vaccine discovery annually, there is so far only one vaccine (RTS,S), which was approved in 2015 [3,4]. However, due to its low efficacy, the WHO does not recommend its routine use in infants (6–12 weeks), who suffer greatly from malaria [5]. Another emerging malaria vaccine is R21, which is still under assessment for its safety and effectiveness at the WHO [6]. In humans, malaria is caused by five different species of *Plasmodium* [7]. Among them, *P. falciparum* and *P. vivax* are responsible for most infections; other cases are caused by *P. ovale, P. malariae* and *P. knowlesi.* Of all five different species, infections caused by *P. falciparum* lead to the highest number of deaths. Therefore, the available treatment mainly focuses on this species.

The current drug treatment of malaria relies on the combination therapy (ACT) of artemisinin with its analogs (dihydroartemisinin, artesunate or artemether) and a different class of antimalarial drug (e.g., amodiaquine or mefloquine) [8]. Due to the unique life cycle of malaria, recrudescence, relapse or reinfection may occur after some symptom-free periods [9]. Moreover, drug resistance has already become a problem in some Southwest Asian countries, although the WHO recommends ACT to delay the rise of resistance [10,11]. Above all, malaria causes a heavy life threat and economic burden in epidemic areas. Therefore, it is of high urgency to develop novel effective antimalarial treatments.

**Figure 1:**
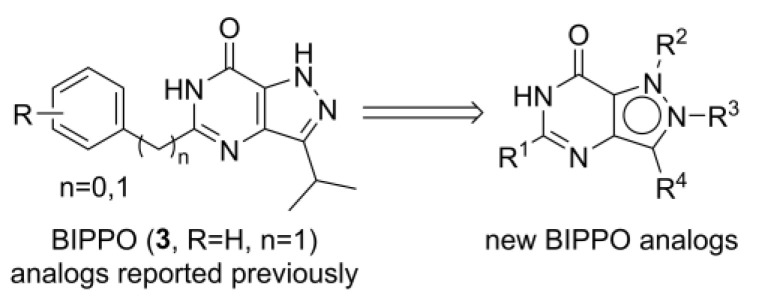
Design of BIPPO analogs in the present study.

In 2015, Howard et al. reported the discovery of BIPPO (5-benzyl-3-isopropyl-1,6-dihydro-7*H*-pyrazolo [4,3-*d*]pyrimidin-7-one, **3**, Figure 1 and Table 1) and its analogs as potent antimalarial agents against asexual blood-stage *P. falciparum* with low toxicity [12]. Together with its favorable physiochemical properties, e.g., low molecular weight (268 Dalton), low cLogP (2.4), good solubility and metabolic stability (not shown), we considered BIPPO as a good starting point for an antimalarial drug discovery program. In this paper, we present a systematic modification of BIPPO (Figure 1) to improve its antimalarial activity.

## 2. Results and Discussion

### 2.1. Design and Synthesis of BIPPO Analogs

**Scheme 1:**
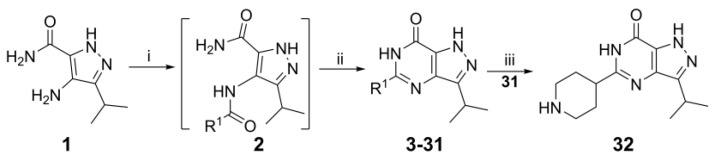
Synthetic routes of R^1^ analogs.

**Scheme 2:**
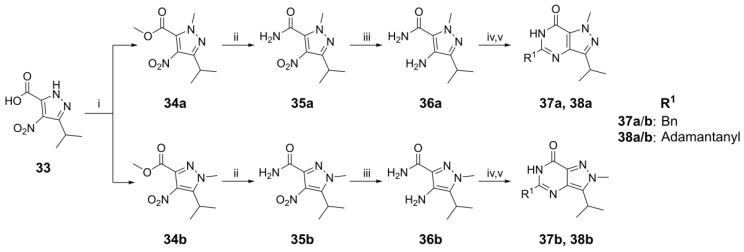
Synthetic routes of R^2^ and R^3^ analogs.

Previously, structural modifications focused mainly on substituents on the benzyl group of BIPPO and its phenyl analogs [12]. To explore the structure–activity relationship of this scaffold in more detail, first a few structurally close BIPPO analogs with various R^1^ substituents were synthesized with the route [13] shown in Scheme 1. The first step started from a commercially available 4-aminopyrazole **1** with a condensation reaction to form the amide intermediate **2**, after which a ring closure reaction under basic conditions yields the desired products **3–32**. Interestingly, during the synthesis of the R^1^ analogs, instead of the originally designed 4-cyclohexanone analog, two alcohol diastereomers, **26** and **27**, were obtained after the ring closure reaction, probably because the carbonyl group was reduced under basic conditions in the microwave reaction; reactions with similar conditions were reported previously [14,15]. The structures of these two diastereomers could be confirmed with selective proton decoupling ^1^H NMR. Next, to explore the chemical space of R^2^ and R^3^ positions, a methyl group was introduced. Previously, Howard et al. reported the synthesis of R^2^ methylated BIPPO analog (**37a**) from BIPPO with dimethyl sulfate (DMS). However, the analog with a methyl group at the R^3^ position (**37b**) was not reported. Here, we report the synthesis of these two analogs with a different synthetic route (Scheme 2) and confirm their regiochemistry. Starting from the carboxylic acid intermediate **33 [13]**, the pyrazole methylation in the first step leads to the two regio-isomers **34a** and **34b**, which were separated by column chromatography and structurally identified with a 1D-NOESY NMR method. The subsequent amidation and reduction reactions resulted in intermediates **36a** and **36b** in high yields (92% and 96%), without purification of the intermediates **35a** and **35b**. Amide coupling and ring closure reactions under basic conditions, as shown in Scheme 1, yielded analogs with a methyl group at R^2^ (**37a**, **38a**) and R^3^ (**37b**, **38b**) positions in moderate to good yields (37–70%). 

**Scheme 3:**
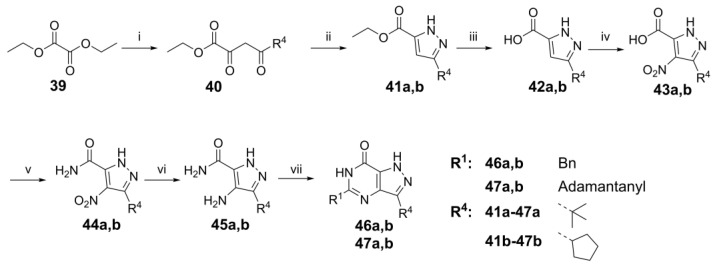
Synthetic route of R^4^ analogs.

The last modification focused on the R^4^ position, where a *tert*-butyl group and a cyclopentyl group were introduced instead of the isopropyl group in **3**. These analogs (**46a**/**b**, **47a**/**b**) could be obtained with the previously reported route for **3** (Scheme 3) [13]. 

During the chemical characterization of the compounds, it was observed that some carbon signals from the non-*N*-substituted pyrazoles were not visible in ^13^C NMR spectra. By using ^13^C NMR combined with 2D NMR (HSQC and HMBC), the compounds were unambiguously characterized [12,16].

Reagents and conditions: (i) R^1^COOH, TEA, PyBrop, DCE, MW 120 °C, 20 min; (ii) KO*^t^*Bu, *^i^*PrOH, MW 130 °C, 30 min; and (iii) 1.0 M aq. HCl, 1,4-dioxane, RT, 16 h.

Reagents and conditions: (i) MeI, K_2_CO_3_, DMF, 60 °C, 1 h; (ii) 7 M NH_3_ in MeOH, RT, 16 h; (iii) 10% Pd/C, H_2_ (g), EtOH, 60 °C, 16 h; (iv) R^1^COOH, TEA, PyBrop, DCE, MW 120 °C, 20 min; and (v) KO*^t^*Bu, *^i^*PrOH, MW 130 °C, 30 min.

Reagents and conditions: (i) R^4^(CO)CH_3_, NaOEt, EtOH 60 °C, 2 h; (ii) N_2_H_4_·H_2_O, EtOH, RT, 18 h; (iii) 1.0 M aq. NaOH, 1,4-dioxane, RT, 3 h; (iv) 65% HNO_3_, 98% H_2_SO_4_, 60 °C, 3 h; (v) (1) *cat*. DMF, (COCl)_2_, DCM, RT, 2 h; (2) 7 M NH_3_ in MeOH, RT, 6 h; (vi) 10% Pd/C, H_2_ (g), EtOH, 60 °C, 16 h; and (vii) (1) R^1^COOH, TEA, PyBrop, DCE, MW 120 °C, 20 min; (2) KO*^t^*Bu, *^i^*PrOH, MW 130 °C, 30 min.

### 2.2. Antimalarial Activities of BIPPO Analogs

Previously, Howard et al. reported (sub)micromolar activity against *P. falciparum* for a series of BIPPO analogs with benzyl substituents [12]. To further understand the structure–activity relationship (SAR) of this interesting scaffold, close analogs of **3** with substituents at R^1^–R^4^ positions were tested against *P. falciparum* and human MRC-5 cells as the control for non-specific toxicity (Table 1). 

To improve the chemical diversity and solubility of **3**, instead of the benzyl group, a pyridylmethyl group (**4**) was introduced at the R^1^ position, which led to a sixfold decreased antimalarial potency compared to **3**. Analogs **5**, **6** and **12–17** were designed to understand the influence of linker flexibility, linker length and chemical space around the linker. Except for **13**, **14** and **17** with an equal potency compared to **3**, the antimalaria potencies of other analogs (**5**, **6**, **12** and **15–16**) were 5- to 25-fold lower than **3**. All analogs with aromatic substituents (**6–11**) directly attached at the R^1^ position exhibited significantly decreased activity compared with **3**. 

For the R^2^, R^3^ and R^4^ analogs, decreased antimalarial activities were observed for the *N*-methyl analogs **37a** (pIC_50_ of 5.0) and **37b** (pIC_50_ of 5.3) compared with **3**. At the R^4^ position, the isopropyl substituent of **3** was replaced with a *tert*-butyl (**46a**) or cyclopentyl groups (**46b**). Since both analogs show similar antimalarial potencies compared with **3**, no further modifications were made at the R^4^ position, as the larger substituents also led to a decreased aqueous solubility (e.g., cLogS of -4.0 for **46b** compared to -2.9 for **3**). 

Following our initial screening of close analogs of **3**, the large activity differences against *P. falciparum* following variation at the R^1^ position (especially between **3** and **6**, benzyl group versus phenyl group) and equal potency of **13** (α-methyl) suggested R^1^ as a promising position for follow-up modifications. To further explore the R^1^ position of this scaffold, a series of BIPPO analogs with various substituents at the R^1^ position was synthesized and tested against *P. falciparum* and MCR-5 cells. From a series of analogs without a substituent at R^1^ or with alkyl R^1^ substituents with increasing sizes (**18–23**), it appeared that the antimalarial potency increased with the size of the R^1^ substituent. Analogs **19–21** with relatively small acyclic aliphatic substituents and **18** show lower or equal potency compared with **3**, while **22**, **23** and **28–30** with bulkier substituents exhibit improved activity (Table 2). Notably, the introduction of a cyclohexyl group (**23**) and an adamantanyl group (**30**) leads to a five- and eightfold potency increase, respectively, compared with **3**. To improve the solubility of **23**, three heterocyclic substituted analogs (**24**, **25**, **32**) and two analogs (**26**, **27**) with a hydroxyl group were synthesized. Unfortunately, these modifications all resulted in less active analogs compared to **23** (Table 2), indicating that heteroatoms and polar groups are not tolerated at this position. 

From all our efforts to improve the potency of **3** at the R^1^ position, **30** with an adamantanyl group turned out to be the most potent compound (pIC_50_ 6.8) against *P. falciparum* without showing noticeable toxicity for human MCR-5 cells. Taking **30** as a starting point, further modifications focused on R^2–4^ positions based on the previous synthetic routes (Scheme 1 and Scheme 2). Unfortunately, no potency improvement was achieved within this series (Table 3). Biological analysis of the R^2–4^ analogs of **30** resulted in a similar SAR as for the related analogs of **3** (Table 3). The introduction of a methyl group at the R^2^ position (**38a**) led to a more than 400-fold potency decrease. Analog **38b** with a methyl group at the R^3^ position was more than 15-fold less active compared with **30**. The introduction of a *tert*-butyl group (**47a**) and a cyclopentyl group (**47b**) at the R^4^ position led to a 4- and 2.5-fold potency decrease, respectively.

### 2.3. Metabolic Stability Test

**Figure 2:**
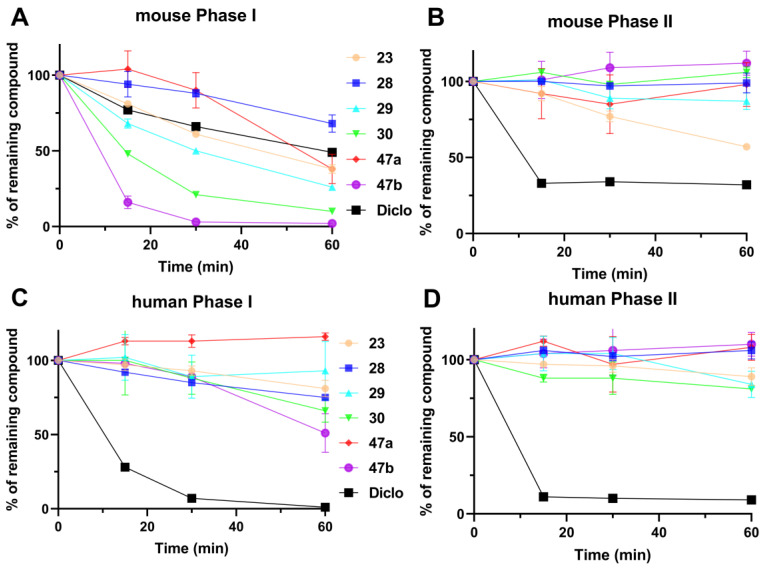
In vitro mouse and human microsomal stability of analogs of **3**. (**A**) Mouse microsomal stability (Phase I) of selected compounds; (**B**) Mouse microsomal stability (Phase II) of selected compounds; (**C**) Human microsomal stability (Phase I) of selected compounds; (**D**) Human microsomal stability (Phase II) of selected compounds. Diclofenac (black) was used as a reference compound. Source data are provided in Table S1.

The modifications of **3** resulted ultimately in an increase in the pIC_50_ of **3** from 5.9 to 6.8 by introducing an adamantanyl group (**30**) at the R^1^ position. Thus, compounds **23**, **28–30**, **47a** and **47b** with high potencies (pIC_50_ > 6) were evaluated for their in vitro metabolic stability in human and mouse liver microsomes (S9 fraction) with diclofenac as a reference compound. As summarized in Figure 2, substituents in the R^4^ position affect metabolic stability. Analogs **23**, **28** and **29** with an isopropyl group at the R^4^ position showed similar metabolic stability. They exhibited good stability with human liver microsomes, with more than 50% of the parent compounds remaining after one hour incubation in conditions with Phase I and Phase II metabolism. Their stability against mouse liver microsomes is lower, especially for their Phase I metabolism; only 38% and 26% of the parent compounds are left for **23** and **29** after one hour of incubation. Analog **28** with a difluoro-cyclohexyl group at the R^1^ position exhibited slightly improved metabolic stability with mouse liver microsomes; 68% of the parent compound was observed after one hour of incubation. For the adamantanyl analogs **30**, **47a** and **47b**, metabolic stability differs drastically, with Phase I identified as the main metabolic pathway. Analog **47b** was metabolized for >95% within 30 min with mouse liver microsomes, while 90% of **47a** (*tert*-butyl analog) was left after one hour of incubation. In general, all compounds showed better metabolic stability than diclofenac in the mouse Phase II and both human Phase I/II assays. The analogs **23**, **29**, **30** and **47b** were metabolized faster than diclofenac in the mouse Phase I assay. 

## 3. Conclusions

Based on its potency as an antimalarial and its drug-like properties, we took BIPPO (**3**) as a starting point for a hit optimization program. Systematic modification identified the R^1^ position in the structure of BIPPO as a key position to improve antimalarial potency. The introduction of aliphatic substituents at this position yielded the adamantanyl analog **30**, which is around one log unit more potent than the parent compound BIPPO against asexual blood-stage *Plasmodium*. The metabolic stability assay indicates that BIPPO analogs **23**, **28**, **29** and **47a** have a sufficient stability profile for in vivo studies. In summary, this systematic modification of BIPPO yields a series of analogs with high antimalarial potency against the blood-stage form of *P. falciparum*. Together with their good drug-like properties and in vitro metabolic stability, they can serve as tool compounds for further hit-to-lead optimization towards candidates for advanced antimalarial studies.

## 4. Materials and Methods

### 4.1. Chemistry

All starting materials were obtained from commercial suppliers and used without purification. Synthesis of **1**, **3**, **6**, **18**, **19**, **36b**, **37a** and **43b** was reported previously [12,13,16,17,18,19,20]. Anhydrous THF, DCM and DMF were obtained by passing through an activated alumina column prior to use. All reactions were carried out under a nitrogen atmosphere unless mentioned otherwise. TLC analyses were performed using Merck F_254_ aluminum-backed silica plates and visualized with 254 nm UV light. Flash column chromatography was executed using Biotage Isolera equipment. All HRMS spectra were recorded on a Bruker microTOF mass spectrometer using ESI in positive-ion mode. All NMR spectra were recorded on either a Bruker Avance 300, 500 or 600 spectrometer. The peak multiplicities are defined as follows: s, singlet; d, doublet; t, triplet; q, quartet; p, pentet; dd, doublet of doublets; dt, doublet of triplets; td, triplet of doublets; br, broad; m, multiplet; and app, apparent. The spectra were referenced to the internal solvent peak as follows: CDCl_3_ (δ = 7.26 ppm in ^1^H NMR, δ = 77.16 ppm in ^13^C NMR) and DMSO-*d*_6_ (δ = 2.50 ppm in ^1^H NMR, δ = 39.52 ppm in ^13^C NMR). IUPAC names were adapted from ChemBioDraw Ultra 19.0. Purities were measured with the aid of analytical LC-MS using a Shimadzu LC-20AD liquid chromatography pump system with a Shimadzu SPDM20A diode array detector with the MS detection performed with a Shimadzu LCMS-2010EV mass spectrometer operating in positive ionization mode. The column used was an Xbridge (C18) 5 μm column (100 mm × 4.6 mm). The following solutions were used for the eluents. Acidic mode eluent A: H_2_O/HCOOH 999:1, and solvent B: MeCN/HCOOH 999:1. Basic mode eluent A: 0.04% (*w*/*v*) (NH_4_)HCO_3_ aqueous solution, and solvent B: 0.04% (NH_4_)HCO_3_ (*w*/*v*) in MeCN:H_2_O 9:1. The eluent program used is as follows: flow rate: 1.0 mL/min, start with 95% A in a linear gradient to 10% A over 4.5 min, hold 1.5 min at 10% A, in 0.5 min in a linear gradient to 95% A, hold 1.5 min at 95% A, total run time: 8.0 min. Compound purities were calculated as the percentage peak area of the analyzed compound by UV detection at 254 nm. Note: not all ^13^C signals are visible in spectrum due to tautomerism of non-*N*-substituted pyrazoles; 2D NMR (HSQC and HMBC) spectra were measured to assign ^13^C signals if applicable.

The general method for the synthesis of final compounds: An amine (1.0 eq) and the corresponding acid (1.0 eq), PyBrop (1.1 eq) and TEA (2.0 eq) were combined in DCE and heated using microwave irradiation at 120 °C for 20 min. The reaction mixture was purified using column chromatography to obtain the amide intermediates. Then, the amide intermediate was combined with KO*^t^*Bu (2.0 eq) in *^i^*PrOH and heated using microwave irradiation at 130 °C for 30 min. The reaction mixture was concentrated in vacuo and purified using column chromatography to obtain the final product.

**5-Benzyl-3-isopropyl-1,6-dihydro-7*H*-pyrazolo[4,3-*d*]pyrimidin-7-one**, **3** (NPD-0019). Prepared from **1** via the general method to give the title compound as a white solid (87 mg, 68% for two steps). ^1^H NMR (500 MHz, DMSO-*d*_6_) δ 13.63 (br s, 1H), 12.18 (br s, 1H), 7.37–7.28 (m, 4H), 7.25–7.20 (m, 1H), 3.90 (s, 2H), 3.24 (hept, *J* = 6.8 Hz, 1H) and 1.32 (d, *J* = 7.0 Hz, 6H). ^13^C NMR (151 MHz, DMSO-*d*_6_) δ 152.4 (HMBC), 150.3 (HMBC), 137.1, 128.7, 128.4, 126.6, 40.3, 25.8 (HSQC) and 21.8. LC-MS: t_R_ = 3.66 min, purity: >99%, m/z [M + H]^+^: 269; HR-MS: calc. for C_15_H_16_N_4_O [M + H]^+^; 269.1397, found 269.1385. Spectral data agree with a previous report [12].

**3-Isopropyl-5-(pyridin-4-ylmethyl)-1,6-dihydro-7*H*-pyrazolo[4,3-*d*]pyrimidin-7-one**, **4** (NPD-2960). Prepared from **1** via the general method to give the title compound as a white solid (75 mg, 59% for two steps). ^1^H NMR (500 MHz, DMSO-*d*_6_ + 1 drop of D_2_O) δ 8.48 (d, *J* = 5.3 Hz, 2H), 7.32 (d, *J* = 5.6 Hz, 2H), 3.95 (s, 2H), 3.22 (hept, *J* = 6.9 Hz, 1H) and 1.29 (d, *J* = 7.0 Hz, 6H). ^13^C NMR (126 MHz, DMSO-*d*_6_ + 1 drop of D_2_O) δ 150.6 (HMBC), 149.6, 145.9, 124.2, 39.5, 26.1 (HSQC) and 21.8. LC-MS: t_R_ = 2.26 min, purity: 98%, m/z [M + H]^+^: 270; HR-MS: calc. for C_14_H_15_N_5_O [M + H]^+^; 270.1349, found 270.1341.

**5-(Benzyloxy)-3-isopropyl-1,6-dihydro-7*H*-pyrazolo[4,3-*d*]pyrimidin-7-one**, **5** (NPD-0434). Prepared from **1** via the general method to give the title compound as a white solid (200 mg, 60% for two steps). ^1^H NMR (600 MHz, DMSO-*d*_6_) δ 13.73 (br s, 1H), 12.40 (br s, 1H), 7.33–7.28 (m, 2H), 7.08–7.03 (m, 2H), 6.99–6.95 (m, 1H), 4.95 (s, 2H), 3.26 (app s, 1H) and 1.33 (d, *J* = 7.0 Hz, 6H). ^13^C NMR (151 MHz, DMSO-*d*_6_) δ 157.9, 150.8 (HMBC), 148.9 (HMBC), 142.3 (HMBC), 129.5, 121.2, 114.8, 67.8, 26.2 (HSQC) and 21.8. LC-MS: t_R_ = 3.80 min, purity: >99%, m/z [M + H]^+^: 285; HR-MS: calc. for C_15_H_16_N_4_O_2_ [M + H]^+^; 285.1346, found 285.1341.

**3-Isopropyl-5-phenyl-1,6-dihydro-7*H*-pyrazolo[4,3-*d*]pyrimidin-7-one**, **6** (NPD-3200). Prepared from **1** via the general method to give the title compound as a white solid (73 mg, 32% for two steps). ^1^H NMR (500 MHz, DMSO-*d*_6_ + 1 drop of D_2_O) δ 8.04–7.98 (m, 2H), 7.55–7.48 (m, 3H), 3.33 (hept, *J* = 7.0 Hz, 1H) and 1.37 (d, *J* = 7.0 Hz, 6H). ^13^C NMR (126 MHz, DMSO-*d*_6_ + 1 drop of D_2_O) δ 151.8 (HMBC), 150.4 (HMBC), 143.3 (HMBC), 133.6, 131.3, 129.3, 128.0, 26.6 (HSQC) and 22.4. LC-MS: t_R_ = 3.78 min, purity: >99%, m/z [M + H]^+^: 255; HR-MS: calc. for C_14_H_14_N_4_O [M + Na]^+^; 277.1060, found 277.1070. Spectral data agree with a previous report [12].

**3-Isopropyl-5-(4-(4-methylpiperazin-1-yl)phenyl)-1,6-dihydro-7*H*-pyrazolo[4,3-*d*]pyrimidin-7-one**, **7** (NPD-3282). Prepared from **1** via the general method to give the title compound as a white solid (0.11 g, 35% for two steps). ^1^H NMR (600 MHz, CDCl_3_) δ 10.78 (br s, 1H), 7.89 (d, *J* = 7.9 Hz, 2H), 6.87 (app s, 2H), 3.48 (hept, *J* = 6.6 Hz, 1H), 3.22 (app s, 4H), 2.53 (app s, 4H), 2.33 (s, 3H) and 1.51 (d, *J* = 6.9 Hz, 6H). ^13^C NMR (151 MHz, CDCl_3_) δ 155.8, 152.9, 152.2 (HMBC), 149.5, 139.0, 128.3, 122.7, 114.8, 54.8, 47.7, 46.2, 26.9 and 22.0. LC-MS: t_R_ = 2.53 min, purity: 98%, m/z [M + H]^+^: 353; HR-MS: calc. for C_19_H_24_N_6_O [M + H]^+^; 353.2084, found 353.2078.

**3-Isopropyl-5-(4-(piperidin-1-yl)phenyl)-1,6-dihydro-7*H*-pyrazolo[4,3-*d*]pyrimidin-7-one**, **8** (NPD-3283). Prepared from **1** via the general method to give the title compound as a white solid (70 mg, 23% for two steps). ^1^H NMR (300 MHz, DMSO-*d*_6_) δ 13.62 (br s, 1H), 12.03 (br s, 1H), 7.97 (d, *J* = 8.9 Hz, 2H), 7.00 (d, *J* = 9.0 Hz, 2H), 3.32–3.26 (m, 5H), 1.60 (app s, 6H) and 1.39 (d, *J* = 7.0 Hz, 6H). ^13^C NMR (151 MHz, DMSO-*d*_6_) δ 152.5, 150.6 (HMBC), 141.8 (HMBC), 128.4, 121.5, 114.1, 48.3, 26.3 (HMBC), 24.9, 24.0 and 21.9. LC-MS: t_R_ = 4.34 min, purity: >99%, m/z [M + H]^+^: 338; HR-MS: calc. for C_19_H_23_N_5_O [M + H]^+^; 338.1975, found 338.1964.

**3-Isopropyl-5-(thiazol-4-yl)-1,6-dihydro-7*H*-pyrazolo[4,3-*d*]pyrimidin-7-one**, **9** (NPD-2973). Prepared from **1** via the general method to give the title compound as a white solid (67 mg, 54% for two steps). ^1^H NMR (500 MHz, DMSO-*d*_6_ + 1 drop of D_2_O) δ 9.26 (s, 1H), 8.50 (s, 1H), 3.33 (app s, 1H) and 1.38 (d, *J* = 6.8 Hz, 6H). ^13^C NMR (126 MHz, DMSO-*d*_6_ + 1 drop of D_2_O) δ 155.4 (HSQC), 149.1, 149.0 (HMBC), 144.5, 135.8 (HMBC), 121.9, 25.8 and 22.0. LC-MS: t_R_ = 3.40 min, purity: >99%, m/z [M + H]^+^: 262; HR-MS: calc. for C_11_H_11_N_5_OS [M + H]^+^; 262.0757, found 262.0756.

**3-Isopropyl-5-(1-methyl-6-oxo-1,6-dihydropyridin-3-yl)-1,6-dihydro-7*H*-pyrazolo[4,3-*d*]pyrimidin-7-one**, **10** (NPD-2968). Prepared from **1** via the general method to give the title compound as a white solid (46 mg, 34% for two steps). ^1^H NMR (300 MHz, DMSO-*d*_6_) δ 13.73 (br s, 1H), 12.03 (br s, 1H), 8.57 (d, *J* = 2.6 Hz, 1H), 8.10 (dd, *J* = 9.6, 2.6 Hz, 1H), 6.51 (d, *J* = 9.6 Hz, 1H), 3.51 (s, 3H), 3.33 (1H, confirmed by HSQC) and 1.38 (d, *J* = 7.0 Hz, 6H). ^13^C NMR (126 MHz, DMSO-*d*_6_) δ 162.4, 151.5 (HMBC), 142.7 (HMBC), 141.0, 138.9, 119.2, 112.2, 38.1, 26.5 (HSQC) and 22.3. LC-MS: t_R_ = 2.91 min, purity: >99%, m/z [M + H]^+^: 286; HR-MS: calc. for C_14_H_15_N_5_O_2_ [M + H]^+^; 286.1299, found 286.1293.

**3-Isopropyl-5-(1-methyl-2-oxo-1,2-dihydropyridin-4-yl)-1,6-dihydro-7*H*-pyrazolo[4,3-*d*]pyrimidin-7-one**, **11** (NPD-2970). Prepared from **1** via the general method to give the title compound as a white solid (47 mg, 35% for two steps). ^1^H NMR (500 MHz, DMSO-*d*_6_ + 1 drop of D_2_O) δ 7.78 (d, *J* = 7.1 Hz, 1H), 7.11 (s, 1H), 6.87 (dd, *J* = 7.1, 1.7 Hz, 1H), 3.45 (s, 3H), 3.32 (hept, *J* = 7.0 Hz, 1H) and 1.37 (d, *J* = 6.9 Hz, 6H). ^13^C NMR (126 MHz, DMSO-*d*_6_ + 1 drop of D_2_O) δ 162.2, 144.0, 140.2, 117.5, 103.7, 37.2, 26.2 (HSQC) and 22.1. LC-MS: t_R_ = 2.86 min, purity: >99%, m/z [M + H]^+^: 286; HR-MS: calc. for C_14_H_15_N_5_O_2_ [M + K]^+^; 324.0857, found 324.0857.

**3-Isopropyl-5-phenethyl-1,6-dihydro-7*H*-pyrazolo[4,3-*d*]pyrimidin-7-one**, **12** (NPD-3281). Prepared from **1** via the general method to give the title compound as a white solid (96 mg, 71% for two steps). ^1^H NMR (600 MHz, DMSO-*d*_6_) δ 13.52 (br s, 1H), 12.11 (br s, 1H), 7.29–7.25 (m, 4H), 7.20–7.16 (m, 1H), 3.26 (app s, 1H), 3.07–2.97 (m, 2H), 2.88 (app s, 2H) and 1.34 (d, *J* = 6.9 Hz, 6H). ^13^C NMR (151 MHz, DMSO-*d*_6_) δ 154.3 (HMBC), 153.5 (HMBC), 150.8 (HMBC), 141.3, 137.5 (HMBC), 128.9, 128.7, 126.5, 36.2, 33.2, 26.6 (HSQC) and 22.3. LC-MS: t_R_ = 3.87 min, purity: >99%, m/z [M + H]^+^: 283; HR-MS: calc. for C_16_H_18_N_4_O [M + H]^+^; 283.1553, found 283.1545. Spectral data agree with a previous report [12].

**(*racemic*)-3-Isopropyl-5-(1-phenylethyl)-1,6-dihydro-7*H*-pyrazolo[4,3-*d*]pyrimidin-7-one**, **13** (NPD-2969). Prepared from **1** via the general method to yield the title compound as a white solid (95 mg, 71% for two steps). ^1^H NMR (500 MHz, CDCl_3_) δ 10.48 (br s, 1H), 7.34 (d, *J* = 7.2 Hz, 2H), 7.24 (app t, *J* = 7.5 Hz, 2H), 7.18 (t, *J* = 7.2 Hz, 1H), 4.20 (q, *J* = 7.0 Hz, 1H), 3.51 (hept, *J* = 7.0 Hz, 1H), 1.71 (d, *J* = 7.0 Hz, 3H) and 1.52 (d, *J* = 7.0 Hz, 6H). ^13^C NMR (126 MHz, DMSO-*d*_6_) δ 155.9, 155.3, 151.3, 141.5, 137.6, 129.0, 127.7, 127.6, 127.2, 45.0, 26.8, 21.9, 21.9 and 19.7. Note: one extra carbon signal observed due to hindered rotation of the isopropyl group. LC-MS: t_R_ = 4.41 min, purity: >99%, m/z [M + H]^+^: 283; HR-MS: calc. for C_16_H_18_N_4_O [M + H]^+^; 283.1553, found 283.1542.

**3-Isopropyl-5-(2-phenylpropan-2-yl)-1,6-dihydro-7*H*-pyrazolo[4,3-*d*]pyrimidin-7-one**, **14** (NPD-3743). Prepared from **1** via the general method to yield the title compound as a white solid (58 mg, 22% for two steps). ^1^H NMR (500 MHz, DMSO-*d*_6_ + 1 drop of D_2_O) δ 7.34–7.28 (m, 2H), 7.25–7.17 (m, 3H), 3.31 (hept, *J* = 7.4 Hz, 1H), 1.68 (s, 6H) and 1.40 (d, *J* = 7.0 Hz, 6H). ^13^C NMR (126 MHz, DMSO-*d*_6_) δ 158.0 (HMBC), 150.7 (HMBC), 146.3, 128.3, 126.4, 126.2, 44.7, 27.8 (HSQC), 26.1 and 21.9. LC-MS: t_R_ = 4.57 min, purity: >99%, m/z [M + H]^+^: 297; HR-MS: calc. for C_17_H_20_N_4_O [M + H]^+^; 297.1710, found 297.1706.

**(*racemic*)-3-Isopropyl-5-(methoxy(phenyl)methyl)-1,6-dihydro-7*H*-pyrazolo[4,3-*d*]pyrimidin-7-one**, **15** (NPD-3744). Prepared from **1** via the general method to yield the title compound as a white solid (47 mg, 18% for two steps). ^1^H NMR (500 MHz, DMSO-*d*_6_) δ 13.69 (br s, 1H), 12.15 (br s, 1H), 7.53–7.49 (m, 2H), 7.38–7.33 (m, 2H), 7.32–7.27 (m, 1H), 5.25 (s, 1H), 3.35 (s, 3H), 3.29–3.18 (m, 1H), 1.32 (d, *J* = 2.4 Hz, 3H) and 1.31 (d, *J* = 2.4 Hz, 3H). ^13^C NMR (126 MHz, DMSO-*d*_6_) δ 152.6 (HMBC), 150.8 (HMBC), 141.9 (HMBC), 138.4, 128.4, 128.3, 127.0, 82.5, 56.9, 26.0 (HSQC), 21.9 and 21.8. Note: one extra carbon signal was observed due to hindered rotation of the isopropyl group. LC-MS: t_R_ = 3.86 min, purity: >99%, m/z [M + H]^+^: 299; HR-MS: calc. for C_17_H_20_N_4_O [M + H]^+^; 299.1503, found 299.1499.

**3-Isopropyl-5-(1-phenylcyclopropyl)-1,6-dihydro-7*H*-pyrazolo[4,3-*d*]pyrimidin-7-one**, **16** (NPD-3746). Prepared from **1** via the general method to yield the title compound as a white solid (74 mg, 28% for two steps). ^1^H NMR (500 MHz, CDCl_3_) δ 8.58 (br s, 1H), 7.48–7.36 (m, 5H), 3.39 (hept, *J* = 7.0 Hz, 1H), 1.83 (app q, *J* = 3.9 Hz, 2H), 1.44 (d, *J* = 7.0 Hz, 6H) and 1.37 (app q, *J* = 3.9 Hz, 2H). ^13^C NMR (126 MHz, CDCl_3_) δ 155.5, 154.1, 151.7, 138.6, 138.1, 131.0, 129.8, 128.9, 126.2, 29.4, 26.8, 21.8 and 18.0. LC-MS: t_R_ = 4.21 min, purity: >99%, m/z [M + H]^+^: 295; HR-MS: calc. for C_17_H_20_N_4_O [M + H]^+^; 295.1553, found 295.1553.

**3-Isopropyl-5-(1-phenylcyclobutyl)-1,6-dihydro-7*H*-pyrazolo[4,3-*d*]pyrimidin-7-one**, **17** (NPD-3745). Prepared from **1** via the general method to yield the title compound as a white solid (77 mg, 28% for two steps). ^1^H NMR (500 MHz, CDCl_3_) δ 8.65 (s, 1H), 7.40–7.22 (m, 5H), 3.49 (hept, *J* = 7.0 Hz, 1H), 3.06–2.97 (m, 2H), 2.71–2.62 (m, 2H), 2.22–2.09 (m, 1H), 2.03–1.91 (m, 1H) and 1.52 (d, *J* = 6.9 Hz, 6H). ^13^C NMR (126 MHz, CDCl_3_) δ 156.5, 154.9, 152.0, 144.1, 137.5, 129.3, 127.5, 126.6, 126.3, 51.3, 32.7, 27.0, 21.9 and 16.5. LC-MS: t_R_ = 4.67 min, purity: >99%, m/z [M + H]^+^: 309; HR-MS: calc. for C_17_H_20_N_4_O [M + H]^+^; 309.1710, found 309.1709.

**3-Isopropyl-1,6-dihydro-7*H*-pyrazolo[4,3-*d*]pyrimidin-7-one**, **18** (NPD-3378). Prepared from **1** via the general method to give the title compound as a white solid (75 mg, 32% for two steps). ^1^H NMR (600 MHz, DMSO-*d*_6_) δ 13.71 (br s, 1H), 12.08 (br s, 1H), 7.80 (s, 1H), 3.26 (hept, *J* = 6.9 Hz, 1H) and 1.34 (d, *J* = 7.0 Hz, 6H). ^13^C NMR (151 MHz, DMSO-*d*_6_) δ 153.4 (HMBC), 150.4 (HMBC), 141.8, 136.3 (HMBC), 25.9 (HSQC) and 21.8. LC-MS: t_R_ = 2.36 min, purity: >99%, m/z [M + H]^+^: 179; HR-MS: calc. for C_8_H_10_N_4_O [M + H]^+^; 179.0927, found 179.0935. Spectral data are in agreement with a previous report [17,18].

**3-Isopropyl-5-methyl-1,6-dihydro-7*H*-pyrazolo[4,3-*d*]pyrimidin-7-one**, **19** (NPD-3380). Prepared from **1** via the general method to give the title compound as a white solid (0.16 g, 90% for two steps). ^1^H NMR (300 MHz, DMSO-*d*_6_) δ 13.52 (br s, 1H), 11.99 (br s, 1H), 3.23 (hept, *J* = 6.2 Hz, 1H), 2.31 (s, 3H) and 1.32 (d, *J* = 7.0 Hz, 6H). ^13^C NMR (126 MHz, DMSO-*d*_6_) δ 151.1, 141.1 (HMBC), 26.4 (HSQC), 22.4 and 21.5. LC-MS: t_R_ = 2.46 min, purity: >99%, m/z [M + H]^+^: 193; HR-MS: calc. for C_9_H_12_N_4_O [M + H]^+^; 193.1084, found 193.1090. Spectral data agree with a previous report [19].

**3,5-Diisopropyl-1,6-dihydro-7*H*-pyrazolo[4,3-*d*]pyrimidin-7-one**, **20** (NPD-3379). Prepared from **1** via the general method to give the title compound as a white solid (0.17 g, 87% for two steps). ^1^H NMR (300 MHz, DMSO-*d*_6_) δ 13.54 (br s, 1H), 11.88 (br s, 1H), 3.23 (hept, *J* = 6.8 Hz, 1H), 2.87 (hept, *J* = 7.8 Hz, 1H), 1.34 (d, *J* = 6.9 Hz, 6H) and 1.22 (d, *J* = 6.8 Hz, 6H). ^13^C NMR (151 MHz, DMSO-*d*_6_) δ 158.1 (HMBC), 150.7 (HMBC), 32.8, 26.3 (HSQC), 21.8 and 20.7. LC-MS: t_R_ = 3.42 min, purity: >99%, m/z [M + H]^+^: 221; HR-MS: calc. for C_11_H_16_N_4_O [M + H]^+^; 221.1397, found 221.1405.

**5-Butyl-3-isopropyl-1,6-dihydro-7*H*-pyrazolo[4,3-*d*]pyrimidin-7-one**, **21** (NPD-3645). Prepared from **1** via the general method to give the title compound as a white solid (88 mg, 42% for two steps). ^1^H NMR (500 MHz, DMSO-*d*_6_) δ 13.52 (br s, 1H), 12.02 (br s, 1H), 3.29–3.17 (m, 1H), 2.61–2.53 (m, 2H), 1.65 (app p, *J* = 7.6 Hz, 2H), 1.37–1.28 (m, 8H) and 0.89 (t, *J* = 7.4 Hz, 3H). ^13^C NMR (126 MHz, DMSO-*d*_6_) δ 154.5 (HMBC), 150.6 (HMBC), 141.4 (HMBC), 34.2, 29.7, 26.5, 22.3, 22.1 and 14.2. LC-MS: t_R_ = 3.58 min, purity: >99%, m/z [M + H]^+^: 235; HR-MS: calc. for C_12_H_18_N_4_O [M + H]^+^; 235.1553, found 235.1562.

**5-Cyclopentyl-3-isopropyl-1,6-dihydro-7*H*-pyrazolo[4,3-*d*]pyrimidin-7-one**, **22** (NPD-3373). Prepared from **1** via the general method to give the title compound as a white solid (0.13 g, 60% for two steps). ^1^H NMR (600 MHz, DMSO-*d*_6_) δ 13.51 (br s, 1H), 11.97 (br s, 1H), 3.28–3.19 (m, 1H), 3.07–3.00 (m, 1H), 2.00–1.91 (m, 2H), 1.89–1.81 (m, 2H), 1.77–1.69 (m, 2H), 1.64–1.55 (m, 2H) and 1.34 (d, *J* = 7.0 Hz, 6H). ^13^C NMR (151 MHz, DMSO-*d*_6_) δ 157.4 (HMBC), 150.6 (HMBC), 141.9 (HMBC), 43.3, 31.0, 26.7, 25.2 and 21.8. LC-MS: t_R_ = 3.90 min, purity: >99%, m/z [M + H]^+^: 247; HR-MS: calc. for C_13_H_18_N_4_O [M + H]^+^; 247.1553, found 247.1562.

**5-Cyclohexyl-3-isopropyl-1,6-dihydro-7*H*-pyrazolo[4,3-*d*]pyrimidin-7-one**, **23** (NPD-3518). Prepared from **1** via the general method to give the title compound as a white solid (98 mg, 42% for two steps). ^1^H NMR (300 MHz, CD_3_OD) δ 3.48–3.34 (m, 1H), 2.60 (tt, *J* = 12.0, 3.5 Hz, 1H), 2.03–1.83 (m, 4H), 1.81–1.57 (m, 3H) and 1.52–1.28 (m, 9H). ^13^C NMR (151 MHz, CD_3_OD) δ 180.4 (HMBC), 159.3 (HMBC), 153.1 (HMBC), 144.1 (HMBC), 44.7, 32.1, 27.8 (HSQC), 27.1, 26.9 and 22.2. LC-MS: t_R_ = 4.18 min, purity: >99%, m/z [M + H]^+^: 261; HR-MS: calc. for C_14_H_20_N_4_O [M + H]^+^; 261.1710, found 261.1698.

**3-Isopropyl-5-(tetrahydro-2*H*-pyran-4-yl)-1,6-dihydro-7*H*-pyrazolo[4,3-*d*]pyrimidin-7-one**, **24** (NPD-3542). Prepared from **1** via the general method to give the title compound as a white solid (95 mg, 41% for two steps). ^1^H NMR (300 MHz, DMSO-*d*_6_) δ 13.64 (br s, 1H), 11.70 (br s, 1H), 4.01–3.88 (m, 2H), 3.47–3.17 (m, 3H), 2.92–2.74 (m, 1H), 1.85–1.73 (m, 4H) and 1.34 (d, *J* = 6.9 Hz, 6H). ^13^C NMR (151 MHz, DMSO-*d*_6_) δ 156.1 (HMBC), 150.4 (HMBC), 139.8 (HMBC), 66.6, 39.2, 30.3, 26.1 (HSQC) and 21.8. LC-MS: t_R_ = 3.04 min, purity: >99%, m/z [M + H]^+^: 263; HR-MS: calc. for C_13_H_18_N_4_O_2_ [M + H]^+^; 263.1503, found 263.1497.

**3-Isopropyl-5-(1-methylpiperidin-4-yl)-1,6-dihydro-7*H*-pyrazolo[4,3-*d*]pyrimidin-7-one diformate**, **25** (NPD-3374). Prepared from **1** to give the title compound as a white solid (39 mg, 9% for two steps). ^1^H NMR (600 MHz, DMSO-*d*_6_ + 1 drop of D_2_O) δ 8.31 (s, 2H), 3.36 (app d, *J* = 12.2 Hz, 2H), 3.24 (hept, *J* = 7.2 Hz, 1H), 2.88 (app t, *J* = 11.1 Hz, 2H), 2.80 (app t, *J* = 11.1 Hz, 1H), 2.66 (s, 3H), 2.06 (app d, *J* = 12.4 Hz, 2H), 1.93 (app q, *J* = 11.3 Hz, 2H) and 1.30 (d, *J* = 7.0 Hz, 6H). ^13^C NMR (151 MHz, DMSO-*d*_6_ + 1 drop of D_2_O) δ 167.5, 155.4, 151.4 (HMBC), 53.7, 43.7, 37.9, 27.9, 26.4 and 22.4. LC-MS: t_R_ = 2.14 min, purity: >99%, m/z [M + H]^+^: 276; HR-MS: calc. for C_14_H_21_N_5_O [M + H]^+^; 276.1819, found 276.1822.

***cis*-5-(4-Hydroxycyclohexyl)-3-isopropyl-1,6-dihydro-7*H*-pyrazolo[4,3-*d*]pyrimidin-7-one**, **26** (NPD-3543) and ***trans*-5-(4-Hydroxycyclohexyl)-3-isopropyl-1,6-dihydro-7*H*-pyrazolo [4,3-*d*]pyrimidin-7-one**, **27** (NPD-3544). Prepared from **1** with 4-oxocyclohexanecarboxylic acid via the general method to give the title compound **26** as a white solid (79 mg, 24% for two steps) and **27** as a white solid (65 mg, 20% for two steps). **26**: ^1^H NMR (500 MHz, DMSO-*d*_6_) δ 13.53 (br s, 1H), 11.88 (br s, 1H), 4.36 (s, 1H), 3.83 (s, 1H), 3.30–3.20 (m, 1H), 2.62–2.54 (m, 1H), 2.00–1.88 (m, 2H), 1.78–1.67 (m, 2H), 1.65–1.55 (m, 2H), 1.52–1.43 (m, 2H) and 1.34 (d, *J* = 6.9 Hz, 6H). ^13^C NMR (126 MHz, DMSO-*d*_6_) δ 157.4 (HMBC), 150.3 (HMBC), 140.8 (HMBC), 63.8, 41.0, 32.0, 26.1 (HSQC), 24.8 and 21.9. LC-MS: t_R_ = 2.85 min, purity: >99%, m/z [M + H]^+^: 277; HR-MS: calc. for C_14_H_20_N_4_O_2_ [M + H]^+^; 277.1659, found 277.1659. **27**: ^1^H NMR (500 MHz, DMSO-*d*_6_) δ 13.54 (br s, 1H), 11.90 (br s, 1H), 4.59 (d, *J* = 4.4 Hz, 1H), 3.41 (tt, *J* = 8.5, 5.2 Hz, 1H), 3.23 (hept, *J* = 7.6, 7.1 Hz, 1H), 2.49–2.45 (m, 1H), 1.98–1.81 (m, 4H), 1.66–1.52 (m, 2H), 1.32 (d, *J* = 7.0 Hz, 6H) and 1.27–1.14 (m, 2H). ^13^C NMR (151 MHz, DMSO-*d*_6_) δ 157.1 (HMBC), 150.2 (HMBC), 141.2 (HMBC), 68.3, 41.6, 35.0, 29.0, 26.2 (HSQC) and 21.9. LC-MS: t_R_ = 2.76 min, purity: >99%, m/z [M + H]^+^: 277; HR-MS: calc. for C_14_H_20_N_4_O_2_ [M + H]^+^; 277.1665, found 277.1653. NMR proof of diastereomers can be found in the Supporting information (Figure S76).

**5-(4,4-Difluorocyclohexyl)-3-isopropyl-1,6-dihydro-7*H*-pyrazolo[4,3-*d*]pyrimidin-7-one**, **28** (NPD-3545). Prepared from **1** via the general method to give the title compound as a white solid (84 mg, 32% for two steps). ^1^H NMR (500 MHz, DMSO-*d*_6_) δ 13.63 (br s, 1H), 12.01 (br s, 1H), 3.24 (hept, *J* = 7.0 Hz, 1H), 2.79–2.70 (m, 1H), 2.19–2.09 (m, 2H), 2.05–1.96 (m, 2H), 1.95–1.75 (m, 4H) and 1.33 (d, *J* = 7.0 Hz, 6H). ^13^C NMR (126 MHz, DMSO-*d*_6_) δ 155.6, 150.2 (HMBC), 125.7, 123.8, 121.9, 39.0 (HSQC), 32.4 (t, *J* = 24.3 Hz), 26.8, 26.7, 26.0 (HSQC) and 21.8. LC-MS: t_R_ = 3.95 min, purity: >99%, m/z [M + H]^+^: 297; HR-MS: calc. for C_14_H_18_F_2_N_4_O [M + H]^+^; 297.1521, found 297.1523.

**5-(Bicyclo[2.2.2]octan-1-yl)-3-isopropyl-1,6-dihydro-7*H*-pyrazolo[4,3-*d*]pyrimidin-7-one**, **29** (NPD-3546). Prepared from **1** via the general method to give the title compound as a white solid (78 mg, 31% for two steps). ^1^H NMR (500 MHz, DMSO-*d*_6_) δ 3.22 (hept, *J* = 7.0 Hz, 1H), 1.88–1.81 (m, 6H), 1.67–1.62 (m, 1H), 1.62–1.55 (m, 6H) and 1.34 (d, *J* = 7.0 Hz, 6H). ^13^C NMR (126 MHz, DMSO-*d*_6_) δ 159.4, 150.1 (HMBC), 136.3 (HMBC), 37.0, 28.9, 26.4, 25.8, 24.0 and 22.2. LC-MS: t_R_ = 4.75 min, purity: >99%, m/z [M + H]^+^: 287; HR-MS: calc. for C_16_H_22_N_4_O [M + H]^+^; 287.1866, found 287.1873.

**5-(Adamantan-1-yl)-3-isopropyl-1,6-dihydro-7*H*-pyrazolo[4,3-*d*]pyrimidin-7-one**, **30** (NPD-3547). Prepared from **1** via the general method to give the title compound as a white solid (85 mg, 31% for two steps). ^1^H NMR (500 MHz, DMSO-*d*_6_) δ 13.56 (br s, 1H), 11.55 (br s, 1H), 3.23 (hept, *J* = 6.8 Hz, 1H), 2.03 (app s, 3H), 1.99 (app s, 6H), 1.75–1.66 (m, 6H) and 1.35 (d, *J* = 7.0 Hz, 6H). ^13^C NMR (126 MHz, DMSO-*d*_6_) δ 159.7 (HMBC), 150.9 (HMBC), 39.5, 38.9, 36.3, 28.2, 26.5 (HSQC) and 22.3. LC-MS: t_R_ = 5.14 min, purity: >99%, m/z [M + H]^+^: 313; HR-MS: calc. for C_18_H_24_N_4_O [M + H]^+^; 313.2023, found 313.2030.

**3-Isopropyl-5-(piperidin-4-yl)-1,6-dihydro-7*H*-pyrazolo[4,3-*d*]pyrimidin-7-one**, **32** (NPD-3593). Prepared from **1** (0.20 g, 1.2 mmol), *N*-BOC-piperidine-4-carboxylic acid (0.27 g, 1.2 mmol) and PyBrop (0.61 g, 1.3 mmol) via the general method to give the intermediate **31** as a white solid (119 mg, 28% for two steps). A solution of 1.0 M HCl was added dropwise to a 1,4-dioxane (50 mL) solution of **31** and stirred for 16 h. The reaction mixture was concentrated in vacuo and purified with a reverse-phase column to give the title compound as a white solid (46 mg, 53%). ^1^H NMR (500 MHz, CD_3_OD) δ 3.54 (dt, *J* = 12.9, 3.7 Hz, 2H), 3.40–3.33 (m, 1H), 3.14 (td, *J* = 12.6, 3.2 Hz, 2H), 2.96 (tt, *J* = 9.9, 4.1 Hz, 1H), 2.26–2.20 (m, 2H), 2.17–2.08 (m, 2H) and 1.43 (d, *J* = 7.0 Hz, 6H). ^13^C NMR (126 MHz, CD_3_OD) δ 155.7 (HMBC), 154.1 (HMBC), 44.5, 38.9, 28.0, 27.7 (HSQC) and 22.2. LC-MS: t_R_ = 2.04 min, purity: >99%, m/z [M + H]^+^: 262; HR-MS: calc. for C_13_H_19_N_5_O [M + H]^+^; 262.1662, found 262.1668.

**Methyl 3-isopropyl-1-methyl-4-nitro-1*H*-pyrazole-5-carboxylate**, **34a** and **methyl 5-isopropyl-1-methyl-4-nitro-1*H*-pyrazole-3-carboxylate**, **34b**. To a mixture of K_2_CO_3_ (13.9 g, 100 mmol), 33 (5.00 g, 25.1 mmol) in DMF (50 mL) was added MeI (3.45 mL, 55.2 mmol); the reaction mixture was heated at 60 °C for 1 h. After that, this mixture was concentrated in vacuo, dissolved in water (50 mL), extracted with EtOAc (3 × 50 mL) and washed with brine. The combined organic layers were concentrated in vacuo, purified using flash column chromatography on silica gel eluting with EtOAc in cyclohexane (10% to 50%) to give the title compounds **34a** (1.33 g, 23%) and **34b** (1.46 g, 26%) as off-white solids. **34a**: ^1^H NMR (600 MHz, CDCl_3_) δ 3.98 (s, 3H), 3.96 (s, 3H), 3.43 (hept, *J* = 7.1 Hz, 1H) and 1.30 (d, *J* = 6.9 Hz, 6H). ^13^C NMR (151 MHz, CDCl_3_) δ 159.3, 153.3, 132.1, 131.9 (HMBC), 53.7, 39.2, 26.5 and 21.5. LC-MS: t_R_ = 4.46 min, purity: >99%, m/z [M + H]^+^: 228. **34b**: ^1^H NMR (600 MHz, CDCl_3_) δ 3.94 (s, 3H), 3.94 (s, 3H), 3.48 (hept, *J* = 7.2 Hz, 1H) and 1.40 (d, *J* = 7.2 Hz, 6H). ^13^C NMR (151 MHz, CDCl_3_) δ 160.8, 146.3, 137.4, 132.1 (HMBC), 53.1, 39.1, 25.8 and 19.4. LC-MS: t_R_ = 3.96 min, purity: >99%, m/z [M + H]^+^: 228. Regiochemistry confirmed with 1D NOESY spectra (Supporting information Figure S95).

**3-Isopropyl-1-methyl-4-nitro-1*H*-pyrazole-5-carboxamide**, **36a**. Ester **34a** (1.33 g, 5.84 mmol) was dissolved in 7 M NH_3_ in MeOH (4.17 mL, 29.2 mmol) and stirred at RT for 16 h. The reaction mixture was then concentrated in vacuo and added to the suspension of 10% palladium on carbon (0.200 g, 1.88 mmol) in EtOH (50 mL) and heated at 75 °C with H_2_ gas insert for 16 h. After that, the reaction mixture was filtered through celite, concentrated in vacuo and purified using flash column chromatography on silica gel with a gradient elution of MeOH in DCM (0% to 10%) to give the title compound as a pink solid (0.98 g, 92% for two steps). ^1^H NMR (300 MHz, DMSO-*d*_6_) δ 7.51 (br s, 2H), 4.09 (s, 2H), 3.86 (s, 3H), 2.97 (hept, *J* = 7.0 Hz, 1H) and 1.16 (d, *J* = 6.9 Hz, 6H). ^13^C NMR (151 MHz, DMSO-*d*_6_) δ 162.0, 146.1, 128.0, 124.3, 39.0, 24.3 and 21.8. LC-MS: t_R_ = 2.14 min, purity: 97%, m/z [M + H]^+^: 183.

**4-Amino-5-isopropyl-1-methyl-1*H*-pyrazole-3-carboxamide**, **36b**. Ester **34b** (1.46 g, 6.88 mmol) was dissolved in 7 M NH_3_ in MeOH (4.58 mL, 32.1 mmol) and stirred at RT for 16 h. The reaction mixture was then concentrated in vacuo and added to the suspension of 10% palladium on carbon (0.250 g, 2.35 mmol) in EtOH (50 mL) and heated at 75 °C with H_2_ gas insert for 16 h. After that, the reaction mixture was filtered through celite, concentrated in vacuo and purified using flash column chromatography on silica gel with a gradient elution of MeOH in DCM (0% to 10%) to give the title compound as a pink solid (1.20 g, 96% for two steps). ^1^H NMR (300 MHz, DMSO-*d*_6_) δ 7.07 (s, 1H), 6.94 (s, 1H), 4.42 (s, 2H), 3.71 (s, 3H), 3.06 (hept, *J* = 7.0 Hz, 1H) and 1.24 (d, *J* = 7.1 Hz, 6H). ^13^C NMR (151 MHz, DMSO-*d*_6_) δ 166.0, 132.5, 130.4, 129.8, 37.6, 24.3 and 20.0. LC-MS: t_R_ = 1.78 min, purity: >99%, m/z [M + H]^+^: 183. Spectral data are in agreement with a previous report [16].

**5-Benzyl-3-isopropyl-1-methyl-1,6-dihydro-7*H*-pyrazolo[4,3-*d*]pyrimidin-7-one**, **37a** (NPD-3647). Prepared from **36a** via the general method to yield the title compound **37a** as a white solid (0.16 g, 70% for two steps). ^1^H NMR (500 MHz, CD_3_OD) δ 7.34–7.28 (m, 4H), 7.26–7.21 (m, 1H), 4.16 (s, 3H), 3.97 (s, 2H), 3.36 (hept, *J* = 7.0 Hz, 1H) and 1.37 (d, *J* = 7.0 Hz, 6H). ^13^C NMR (151 MHz, CD_3_OD) δ 156.5, 154.7, 151.8, 138.6, 137.7, 129.7, 129.7, 128.1, 126.0, 41.7, 38.3, 27.4 and 22.4. LC-MS: t_R_ = 4.16 min, purity: >99%, m/z [M + H]^+^: 283; HR-MS: calc. for C_16_H_18_N_4_O [M + H]^+^; 283.1553, found 283.1556. Spectral data are in agreement with a previous report [12].

**5-Benzyl-3-isopropyl-2-methyl-2,6-dihydro-7*H*-pyrazolo[4,3-*d*]pyrimidin-7-one**, **37b** (NPD-3646). Prepared from **36b** via the general method to yield the title compound as a white solid (85 mg, 37% for two steps). ^1^H NMR (600 MHz, CD_3_OD) δ 7.36–7.28 (m, 4H), 7.25–7.21 (m, 1H), 4.05 (s, 3H), 3.93 (s, 2H), 3.40 (hept, *J* = 7.0 Hz, 1H) and 1.49 (d, *J* = 7.0 Hz, 6H). ^13^C NMR (151 MHz, CD_3_OD) δ 159.9, 153.4, 143.3, 137.9, 136.7, 134.9, 129.8, 129.6, 128.0, 42.0, 38.8, 27.4 and 21.4. LC-MS: t_R_ = 3.92 min, purity: >99%, m/z [M + H]^+^: 283; HR-MS: calc. for C_16_H_18_N_4_O [M + H]^+^; 283.1553, found 283.1554.

**5-(Adamantan-1-yl)-3-isopropyl-1-methyl-1,6-dihydro-7*H*-pyrazolo[4,3-*d*]pyrimidin-7-one**, **38a** (NPD-3642). Prepared from **36a** via the general method to give the title compound as a white solid (123 mg, 46% for two steps). ^1^H NMR (500 MHz, CDCl_3_) δ 10.02 (s, 1H), 4.23 (s, 3H), 3.34 (hept, *J* = 6.9 Hz, 1H), 2.13 (s, 3H), 2.04 (app s, 6H), 1.84–1.74 (m, 6H) and 1.42 (d, *J* = 6.9 Hz, 6H). ^13^C NMR (151 MHz, CDCl_3_) δ 158.8, 155.4, 151.2, 138.0, 124.7, 40.4, 38.9, 38.2, 36.5, 28.4, 27.0 and 22.0. LC-MS: t_R_ = 5.79 min, purity: 97%, m/z [M + H]^+^: 327; HR-MS: calc. for C_19_H_26_N_4_O [M + H]^+^; 327.2179, found 327.2170.

**5-(Adamantan-1-yl)-3-isopropyl-2-methyl-2,6-dihydro-7*H*-pyrazolo[4,3-*d*]pyrimidin-7-one**, **38b** (NPD-3641). Prepared from **36b** via the general method to give the title compound as a white solid (112 mg, 42% for two steps). ^1^H NMR (500 MHz, CDCl_3_) δ 8.72 (s, 1H), 4.04 (s, 3H), 3.30 (hept, *J* = 7.0 Hz, 1H), 2.13 (app s, 3H), 1.98 (d, *J* = 2.6 Hz, 6H), 1.77 (app q, *J* = 12.3 Hz, 6H) and 1.50 (d, *J* = 7.0 Hz, 6H). ^13^C NMR (126 MHz, DMSO-*d*_6_) δ 157.9, 157.8, 141.7, 135.1, 134.0, 40.4, 38.8, 38.8, 36.5, 28.3, 26.5 and 21.3. LC-MS: t_R_ = 5.31 min, purity: >99%, m/z [M + H]^+^: 327; HR-MS: calc. for C_19_H_26_N_4_O [M + H]^+^; 327.2179, found 327.2174.

**3-Cyclopentyl-1*H*-pyrazole-5-carboxylic acid**, **42b**. NaOEt (3.89 g, 54.9 mmol) was dissolved in EtOH (50 mL) at RT and a solution of diethyl oxalate (7.56 mL, 55.4 mmol) in 1-cyclopentylethanone (5.67 mL, 46.1 mmol) was added dropwise at RT for 30 min. The reaction mixture was diluted with EtOH (50 mL) and heated to 60 °C for 2 h, after which AcOH (8.9 mL, 55 mmol) and 64–65% N_2_H_4_ monohydrate (2.20 mL, 46.1 mmol) were added, and the mixture was stirred under reflux for 2 h. The reaction mixture was concentrated under reduced pressure and mixed with aqueous NaOH solution (97 mL, 97 mmol) in 1,4-dioxane (112 mL); the reaction mixture was heated to 50 °C and stirred for 20 h. Then, the reaction was cooled to RT, and 1,4-dioxane was removed under reduced pressure. The residue was washed with diethyl ether (100 mL). The water layer was acidified to pH 1 with concentrated HCl (37%). The white solid was filtered and dried in vacuo to yield the title product **42b** as a white solid (5.21 g, 63% for three steps). ^1^H NMR (600 MHz, DMSO-*d*_6_) δ 12.90 (br s, 1H), 6.46 (s, 1H), 3.04 (app p, *J* = 8.1 Hz, 1H), 2.02–1.94 (m, 2H), 1.73–1.66 (m, 2H) and 1.64–1.53 (m, 4H). ^13^C NMR (151 MHz, DMSO-*d*_6_) δ 104.6, 36.6 (HMBC), 32.7 and 24.6. LC-MS: t_R_ = 3.19 min, purity: >99%, m/z [M − H]^−^: 179.

**3-(*tert*-Butyl)-4-nitro-1*H*-pyrazole-5-carboxylic acid**, **43a**. Ester **41a** (25.0 g, 127 mmol) was dissolved in a mixture of THF (100 mL) and water (100 mL), after which NaOH (15.3 g, 382 mmol) was added. The reaction mixture was concentrated under reduced pressure after heating at 60 °C for 4 h, washed with EtOAc (3 × 100 mL), adjusted to pH 1 with concentrated HCl solution, and the off-white solid was filtered as intermediate **42a** (16.5 g, 77%), which was used for the next step without further purification. Acid **42a** (3.95 g, 23.5 mmol) was added portion-wise to concentrated H_2_SO_4_ (19.1 mL, 352 mmol) at RT with stirring. The reaction mixture was then heated to 60 °C, and 65% HNO_3_ (4.50 mL, 70.4 mmol) was added dropwise, keeping the temperature at 60 °C. The reaction was stirred at 60 °C for 3 h, cooled to RT and poured onto 200 g of ice with stirring. After 15 min, the white precipitate was isolated by filtration, washed with water and dried under reduced pressure to give the title product **43a** as a white solid (4.50, 90%). ^1^H NMR (300 MHz, DMSO-*d*_6_) δ 13.82 (s, 1H) and 1.34 (s, 9H). ^13^C NMR (151 MHz, DMSO-*d*_6_) δ 147.3 (HMBC), 32.4 (HSQC) and 28.2. LC-MS: t_R_ = 3.26 min, purity: 96%, m/z [M + H]^+^: 214.

**3-Cyclopentyl-4-nitro-1*H*-pyrazole-5-carboxylic acid**, **43b**. Acid **42b** (5.21 g, 28.9 mmol) was added portion-wise to concentrated H_2_SO_4_ (8.91 mL, 159 mmol) at RT with stirring. The reaction mixture was then heated to 60 °C, and 65% HNO_3_ (6.95 mL, 101 mmol) was added dropwise, keeping the temperature at 60 °C. The reaction was stirred at 60 °C for 3 h, cooled to RT and poured onto 200 g of ice with stirring. After 15 min, the white precipitate was isolated by filtration, washed with water and dried under reduced pressure to give the title product **43b** as a white solid (4.01 g, 61%). ^1^H NMR (600 MHz, DMSO-*d*_6_ + 1 drop of D_2_O) δ 3.47 (p, *J* = 8.6 Hz, 1H), 2.09–1.98 (m, 2H), 1.79–1.68 (m, 2H) and 1.68–1.55 (m, 4H). ^13^C NMR (151 MHz, DMSO-*d*_6_ + 1 drop of D_2_O) δ 36.0, 32.0 and 25.5. LC-MS: t_R_ = 3.26 min, purity: >99%, m/z [M − H]^−^: 224. Spectral data are in agreement with a previous report [17].

**4-Amino-3-(*tert*-butyl)-1*H*-pyrazole-5-carboxamide**, **45a**. Oxalyl chloride (6.16 mL, 70.4 mmol) was added dropwise to a suspension of **43a** (5.00 g, 23.5 mmol) in DCM (240 mL) containing DMF (0.082 mL, 1.1 mmol) under nitrogen at 0 °C. The reaction mixture was stirred at 0 °C for 1 h, allowed to warm to RT and stirred for a further 2 h. The reaction mixture was concentrated in vacuo and co-evaporated with toluene three times. The residue was dissolved in DCM (100 mL) and added dropwise to 7 M NH_3_ in MeOH (10.1 mL, 70.4 mmol) at 0 °C. After stirring for 3 h, the reaction mixture was concentrated in vacuo, combined with 10% palladium on carbon (0.85 g, 8.0 mmol) in EtOH (90 mL) and stirred under H_2_ gas insert at 60 °C for 6 h. The reaction mixture was filtered through celite, and the solid was washed with MeOH (50 mL). The filtrate was concentrated under reduced pressure, and the residue was used for the next step without further purification.

**4-Amino-3-cyclopentyl-1*H*-pyrazole-5-carboxamide**, **45b**. Oxalyl chloride (1.09 mL, 12.5 mmol) was added dropwise to a suspension of **43b** (0.94 g, 4.2 mmol) in DCM (20 mL) containing DMF (0.014 mL, 0.18 mmol) under nitrogen at 0 °C. The reaction was stirred at 0 °C for 1 h, allowed to warm to RT and stirred for a further 2 h. The reaction mixture was concentrated in vacuo, combined with 10% palladium on carbon (0.85 g, 8.0 mmol) in EtOH (90 mL) and stirred under H_2_ gas insert at 60 °C for 6 h. The reaction mixture was filtered through celite, and the solid was washed with MeOH (50 mL). The filtrate was concentrated under reduced pressure, and the residue was used for the next step without further purification.

**5-Benzyl-3-(*tert*-butyl)-1,6-dihydro-7*H*-pyrazolo[4,3-*d*]pyrimidin-7-one**, **46a** (NPD-3648). Prepared from **45a** via the general method to yield the title compound as a white solid (0.14 g, 46% for four steps). ^1^H NMR (500 MHz, CD_3_OD) δ 7.36–7.33 (m, 2H), 7.33–7.27 (m, 2H), 7.25–7.20 (m, 1H), 3.98 (s, 2H) and 1.50 (s, 9H). ^13^C NMR (126 MHz, CD_3_OD) δ 155.4 (HMBC), 153.0 (HMBC), 146.6 (HMBC), 138.0, 129.8, 129.6, 128.0, 42.0, 33.8 (HMBC) and 29.9. LC-MS: t_R_ = 4.20 min, purity: >99%, m/z [M + H]^+^: 283; HR-MS: calc. for C_17_H_18_N_4_O [M + H]^+^; 283.1553, found 283.1551.

**5-Benzyl-3-cyclopentyl-1,6-dihydro-7*H*-pyrazolo[4,3-*d*]pyrimidin-7-one**, **46b** (NPD-3604). Prepared from **45b** via the general method to give the title compound as a white solid (0.14 g, 14% for four steps). ^1^H NMR (500 MHz, DMSO-*d*_6_) δ 7.34–7.27 (m, 4H), 7.24–7.19 (m, 1H), 3.88 (s, 2H), 3.31 (app p, *J* = 8.1 Hz, 1H), 2.02–1.94 (m, 2H), 1.84–1.68 (m, 4H) and 1.66–1.56 (m, 2H). ^13^C NMR (126 MHz, DMSO-*d*_6_) δ 153.0, 137.4, 129.1, 129.0, 127.3, 40.6, 36.6, 32.6 and 25.4. LC-MS: t_R_ = 4.05 min, purity: >99%, m/z [M + H]^+^: 295; HR-MS: calc. for C_17_H_18_N_4_O [M + H]^+^; 295.1553, found 295.1542.

**5-(Adamantan-1-yl)-3-(*tert*-butyl)-1,6-dihydro-7*H*-pyrazolo[4,3-*d*]pyrimidin-7-one**, **47a** (NPD-3643). Prepared from **45a** via the general method to give the title compound as a white solid (132 mg, 29% for four steps). ^1^H NMR (500 MHz, CDCl_3_) δ 9.20 (s, 1H), 2.15 (app s, 3H), 2.02 (app s, 6H), 1.79 (app q, *J* = 12.3 Hz, 6H) and 1.53 (s, 9H). ^13^C NMR (126 MHz, DMSO-*d*_6_) δ 158.5, 155.1, 154.0, 137.7, 127.2, 40.5, 39.0, 36.5, 33.4, 29.4 and 28.3. LC-MS: t_R_ = 5.55 min, purity: 99%, m/z [M + H]^+^: 327; HR-MS: calc. for C_19_H_26_N_4_O [M + H]^+^; 327.2179, found 327.2170.

**5-(Adamantan-1-yl)-3-cyclopentyl-1,6-dihydro-7*H*-pyrazolo[4,3-*d*]pyrimidin-7-one**, **47b** (NPD-3644). Prepared from **45b** via the general method to give the title compound as a white solid (97 mg, 11% for four steps). ^1^H NMR (500 MHz, DMSO-*d*_6_ + 1 drop of D_2_O) δ 3.29 (p, *J* = 8.2 Hz, 1H), 2.05–1.88 (m, 11H) and 1.88–1.56 (m, 12H). ^13^C NMR (126 MHz, CD_3_OD + 1 drop of CDCl_3_) δ 159.8, 156.8, 148.0 (HMBC), 136.3 (HMBC), 40.3, 39.3, 37.3, 36.7, 32.8, 28.6 and 26.0. LC-MS: t_R_ = 5.52 min, purity: >99%, m/z [M + H]^+^: 339; HR-MS: calc. for C_20_H_26_N_4_O [M + H]^+^; 339.2179, found 339.2167.

### 4.2. Antimalarial Screening

The assay for antimalarial activity was carried out as described in detail in Pereira et al. [21].

### 4.3. Metabolic Stability

The assay for metabolic stability in human and mouse liver microsomal fractions (S9) was performed as described [22].

## Figures and Tables

**Table 1:** Exploration of BIPPO analogs against *P. falciparum*.

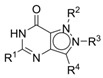								
Comp. No.	R^1^	R^2^	R^3^	R^4^	cLogP ^a^	tPSA ^a^	*P. fal* pIC_50_^b,c^	MRC-5 pIC_50_^b^
**3** (NPD-0019, BIPPO)	Bn	H		*^i^*Pr	2.9	59.3	5.9	< 4.2
**4** (NPD-2960)	4-PyCH_2_	H		*^i^*Pr	1.7	83.0	5.1	< 4.2
**5** (NPD-0434)	PhOCH_2_	H		*^i^*Pr	2.6	79.4	5.2	< 4.2
**6** (NPD-3200)	Ph	H		*^i^*Pr	2.9	70.1	4.5	< 4.2
**7** (NPD-3282)	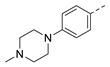	H		*^i^*Pr	2.8	76.6	5.1	< 4.4
**8** (NPD-3283)	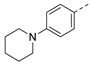	H		*^i^*Pr	3.2	73.4	< 4.3	< 4.2
**9** (NPD-2973)	4-thiazolyl	H		*^i^*Pr	2.0	83.0	< 4.2	< 4.2
**10** (NPD-2968)	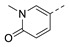	H		*^i^*Pr	1.0	90.5	< 4.2	< 4.2
**11** (NPD-2970)	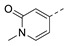	H		*^i^*Pr	1.0	90.5	< 4.2	< 4.2
**12** (NPD-3281)	Ph(CH_2_)_2_	H		*^i^*Pr	3.4	70.1	5.2	< 4.2
**13** (NPD-2969) ^d^	PhCH(CH_3_)	H		*^i^*Pr	3.5	70.1	5.9	< 4.2
**14** (NPD-3743)	PhC(CH_3_)_2_	H		*^i^*Pr	4.0	70.1	6.0	< 4.2
**15** (NPD-3744) ^d^	PhCH(OCH_3_)	H		*^i^*Pr	2.8	79.4	5.1	< 4.2
**16** (NPD-3746)	PhC(CH_2_)_2_	H		*^i^*Pr	3.5	70.1	5.5	< 4.2
**17** (NPD-3745)	PhC(CH_2_)_3_	H		*^i^*Pr	3.3	70.1	5.8	4.6
**37a** (NPD-3647)	Bn	Me	-	*^i^*Pr	2.9	59.3	5.0	< 4.2
**37b** (NPD-3646)	Bn	-	Me	*^i^*Pr	3.0	59.3	5.3	< 4.2
**46a** (NPD-3648)	Bn	H		*^t^*Bu	3.3	70.1	5.8	4.4
**46b** (NPD-3604)	Bn	H		*^c^*Pentyl	3.3	70.1	5.8	< 4.2

^a^ adopted from Collaborative Drug Discovery (CDD) database; ^b^ pIC_50_ values are average values (*n* ≥ 2, s.d.: < 0.3); ^c^ potency against asexual blood stage of *P. falciparum*; ^d^ racemic compound.

**Table 2:** Exploration of R^1^ analogs with aliphatic substitutions against *P. falciparum*.

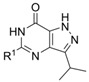					
Comp. No.	R^1^	cLogP ^a^	tPSA ^a^	*P. fal* pIC_50_ ^b,c^	MCR-5 pIC_50_ ^c^
**18** (NPD-3378)	H	1.0	70.1	<4.2	<4.2
**19** (NPD-3380)	Me	1.1	70.1	4.4	<4.2
**20** (NPD-3379)	*^i^*Pr	2.3	70.1	5.4	<4.2
**21** (NPD-3645)	*^n^*Bu	2.7	70.1	5.9	<4.2
**22** (NPD-3373)	*^c^*Pentyl	2.0	70.1	6.1	<4.2
**23** (NPD-3518)	*^c^*Hexyl	2.5	70.1	6.6	<4.2
**24** (NPD-3542)	4-tetrahydropyranyl	0.8	79.4	5.3	<4.2
**25** (NPD-3374) ^d^	4-(1-methylpiperidinyl)	0.3	73.4	<4.2	<4.2
**32** (NPD-3593)	4-piperidinyl	-0.8	82.2	<4.2	<4.2
**26** (NPD-3543)	(*cis*)-4-hydroxycyclohexyl	1.1	90.4	5.2	<4.2
**27** (NPD-3544)	(*trans*)-4-hydroxycyclohexyl	1.1	90.4	5.3	<4.2
**28** (NPD-3545)	4,4-difluorocyclohexyl	2.0	70.1	6.0	<4.2
**29** (NPD-3546)	1-bicyclo [2.2.2]octanyl	2.9	70.1	6.4	<4.2
**30** (NPD-3547)	1-adamantanyl	3.0	70.1	6.8	<4.2

^a^ adopted from CDD; ^b^ potency against asexual blood stage of *P. falciparum*; ^c^ pIC_50_ values are average values (*n* ≥ 2, s.d.: <0.3); ^d^ formate salt.

**Table 3:** Exploration of R^2–4^ analogs of **30** against *P. falciparum*.

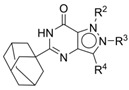							
Comp No.	R^2^	R^3^	R^4^	cLogP ^a^	tPSA ^a^	*P. fal* pIC_50_ ^b,c^	MRC-5 pIC_50_ ^c^
**30** (NPD-3547)	H		*^i^*Pr	3.0	70.1	6.8	<4.2
**38a** (NPD-3642)	Me	-	*^i^*Pr	3.1	59.3	<4.2	<4.2
**38b** (NPD-3641)	-	Me	*^i^*Pr	3.8	59.3	5.6	<4.2
**47a** (NPD-3643)	H		*^t^*Bu	3.5	70.1	6.4	<4.2
**47b** (NPD-3644)	H		*^c^*Pentyl	3.4	70.1	6.2	<4.2

^a^ adopted from CDD; ^b^ potency against asexual blood stage of *P. falciparum*; ^c^ pIC_50_ values are averages (*n* ≥ 2, s.d.: <0.3).

## Data Availability

Not applicable.

## Supplementary Materials

The following supporting information can be downloaded at https://www.mdpi.com/article/10.3390/molecules28134939/s1. Figures S1–S134: LC-MS and NMR spectra of intermediates and final compounds; Table S1: In vitro microsomal stability of BIPPO analogs.

## Abbreviations

ACT, artemisinin combination therapy; CYP, cytochrome P450; HMBC, heteronuclear multiple bond correlation; HSQC, heteronuclear single quantum coherence; NADPH, nicotinamide adenine dinucleotide phosphate; NOESY, nuclear Overhauser effect spectroscopy; PDE, phosphodiesterase; WHO, World Health Organization.
