# Supplementary material for: Structural Optimization of BIPPO Analogs as Potent Antimalarials
Source: Molecules. 2023 Jun 23;28(13):4939. doi: 10.3390/molecules28134939 (PMC10343887; doi:10.3390/molecules28134939)
Supplement: Supplementary file 1 [file molecules-28-04939-s001.zip › molecules-2447714-supplementary.pdf]

Structural optimization of BIPPO analogs as potent antimalarials

Yang Zheng,[1] An Matheeussen,[2] Louis Maes,[2] Guy Caljon,[2] Geert Jan Sterk[1] and Rob Leurs[1,*]

[1] Amsterdam Institute for Molecules, Medicines and Systems, Division of Medicinal Chemistry, Faculty of Science, VU University Amsterdam, De Boelelaan 1083, 1081 HV Amsterdam, the Netherlands.

[2] Laboratory of Microbiology, Parasitology and Hygiene, University of Antwerp, 2610 Wilrijk, Belgium.

Table of contents

Page S2. Table S1. In vitro microsomal stability of BIPPO analogs.
Page S3-S92. LCMS, NMR spectra of intermediates and final compounds.

Table S1. In vitro microsomal stability of BIPPO analogs. Diclofenac was used as a reference compound.

Species	Phase I/II	Time (min)	% **23** remaining		% **28** remaining		% **29** remaining		% **30** remaining		% **47a** remaining		% **47b** remaining		**Diclofenac**
			Mean	SD	Mean	SD	Mean	SD	Mean	SD	Mean	SD	Mean	SD	Mean
Mouse	CYP-NADPH	0	100	-	100	-	100	-	100	-	100	-	100	-	100
		15	81	2.0	94	8.4	68	3.1	48	2.4	104	12.1	16	4.2	77
		30	61	1.3	88	2.4	50	0.8	21	0.59	90	11.7	3	0.002	66
		60	38	2.9	68	5.7	26	1.6	10	0.31	38	9.7	2	1.3	49
	UGT Enzymes	0	100	-	100	-	100	-	100	-	100	-	100	-	100
		15	92	4.7	100	1.8	101	0.5	106	0.6	92	16.6	101	12.2	33
		30	77	3.6	97	1.1	89	7.0	98	0.5	85	19.3	109	10.2	34
		60	57	0.4	99	6.5	87	5.5	106	3.5	98	14.4	112	7.9	32
Human	CYP-NADPH	0	100	-	100	-	100	-	100	-	100	-	100	-	100
		15	97	3.9	92	0.6	102	15.4	100	23.2	113	2.5	98	4.3	28
		30	93	3.3	85	2.3	89	14.5	88	10.9	113	4.2	89	1.1	7
		60	81	5.5	75	1.3	93	19.7	66	7.7	116	2.6	51	13.1	1
	UGT Enzymes	0	100	-	100	-	100	-	100	-	100	-	100	-	100
		15	97	1.5	106	2.2	104	11.2	88	2.6	112	3.3	104	9.3	11
		30	96	4.3	102	2.7	104	10.4	88	10.5	97	18.1	106	16.4	10
		60	89	5.7	106	5.6	84	8.5	81	0.5	108	8.4	110	7.7	9

Acquired by	: Admin
Date Acquired	: 11/6/2019 8:48:12 AM
Sample Name	: BIPPO
Sample ID	:
Tray#	: 1
Vial#	: 1
Injection Volume	: 2
Data File	: C:\LabSolutions\Data\2019\2019-wk24\BIPPO.lcd
Background File	: Blanco11062019.lcd
Method File	: Method SCAN ACID standard.lcm
Report Format	: DefaultLCMS.lcr
Tuning File	: C:\LabSolutions\Tuning\Tuning-ESI-pos-neg01072015a.lct
Processed by	: Admin
Modified Date	: 11/6/2019 9:17:16 AM

PDA Graph

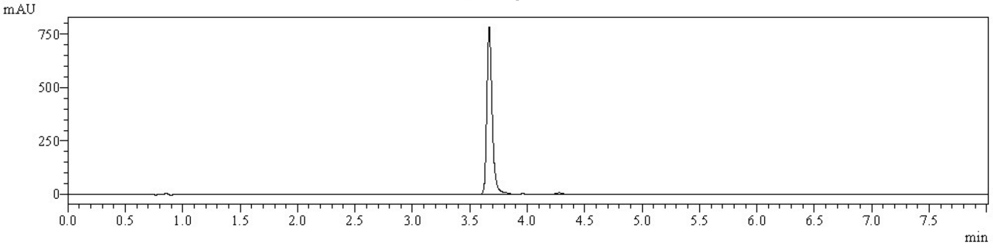

PeakTable

PDA Ch1 254nm 4nm

Peak#	Name	Ret. Time	Area	Area %
1		3.663	2492261	99.036
2		4.272	24258	0.964

MS Chromatogram

BIPPO C:\LabSolutions\Data\2019\2019-wk21-30\2019-wk24\BIPPO.lcd

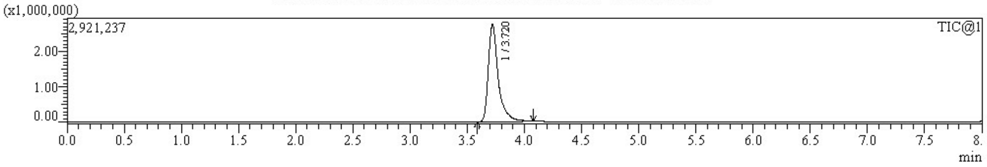

MS Spectrum Graph

#:1 Ret.Time:Averaged 3.710-3.730(Scan#:372-374)
BG Mode:Calc 3.590<->4.080(360<->409)
Mass Peaks:11 Base Peak:269.10(1938726) Polarity:Pos Segment1 - Event1

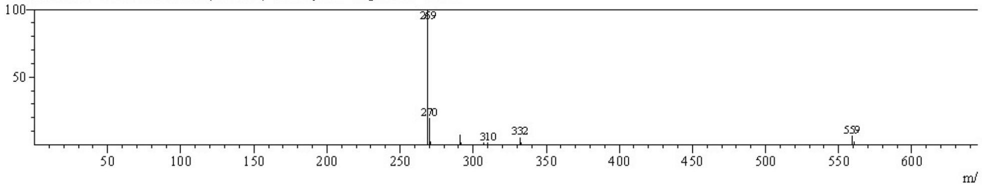

MS Spectrum Table

#:1 Ret.Time:
BG Mode:Calc 3.590<->4.080(360<->409)
Mass Peaks:11 Base Peak:269.10(1938726) Polarity:Pos Segment1 - Event1

#	m/z	Abs.Inten.	Rel.Inten.	Charge	Polarity	Monoisotopic
1	269.10	1938726	100.00			
2	270.10	372456	19.21			
3	271.05	38608	1.99			
4	291.10	138093	7.12			
5	292.05	27110	1.40			
6	307.05	21862	1.13			
7	310.15	25842	1.33			
8	332.10	103390	5.33			
9	333.10	21381	1.10			
10	559.30	122379	6.31			
11	560.30	39867	2.06			

Figure S1. LCMS spectrum of compound **3**.

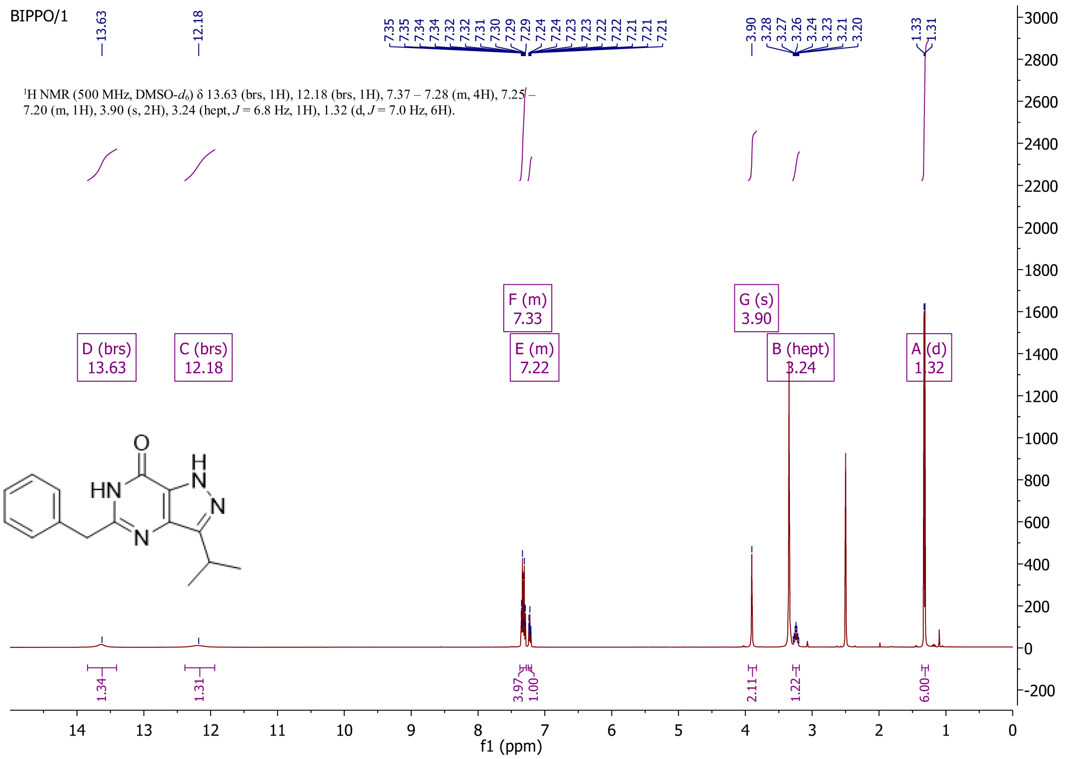

Figure S2. ^{1}H NMR spectrum of compound **3**.

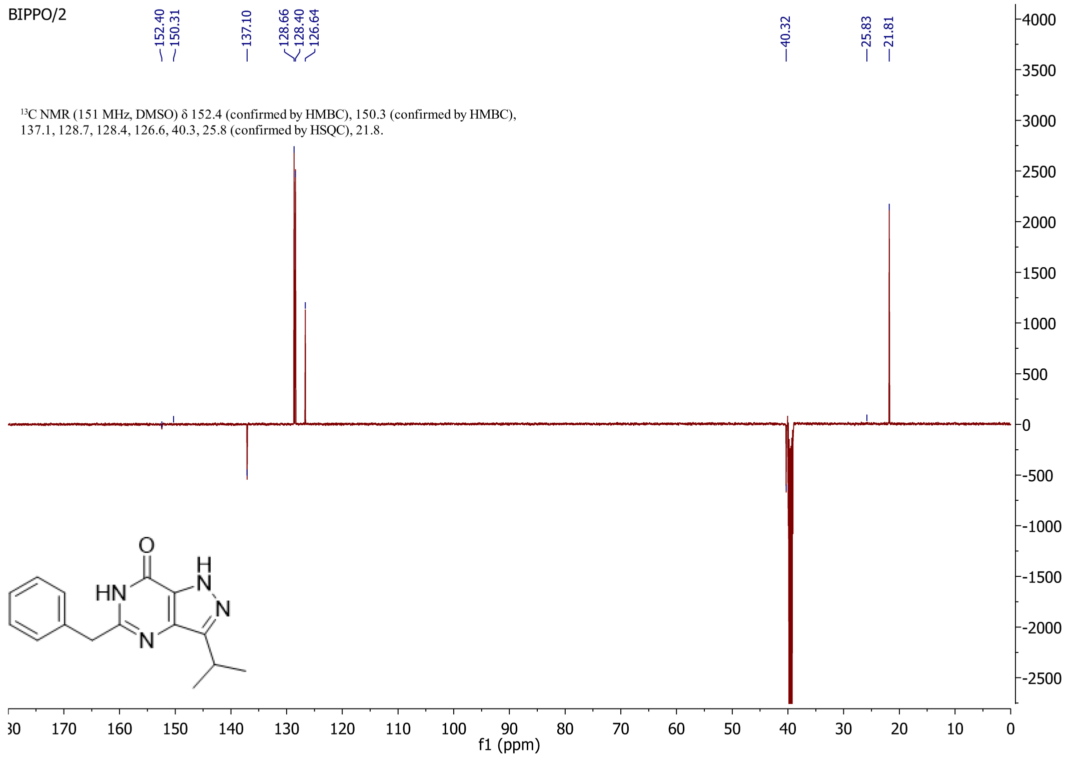

Figure S3. ^{13}C NMR spectrum of compound **3**.

Acquired by	: Admin
Date Acquired	: 4/20/2016 2:03:12 PM
Sample Name	: YAZH-115
Sample ID	:
Tray#	: 1
Vial#	: 33
Injection Volume	: 1
Data File	: C:\LabSolutions\Data\2016 - wk16\YAZH-115.lcd
Background File	: blanco 20042016.lcd
Method File	: Method SCAN ACID standard.lcm
Report Format	: DefaultLCMS.lcr
Tuning File	: C:\LabSolutions\Tuning\Tuning-ESI-pos-neg01072015a.lct
Processed by	: Admin
Modified Date	: 4/20/2016 2:22:13 PM

PDA Graph

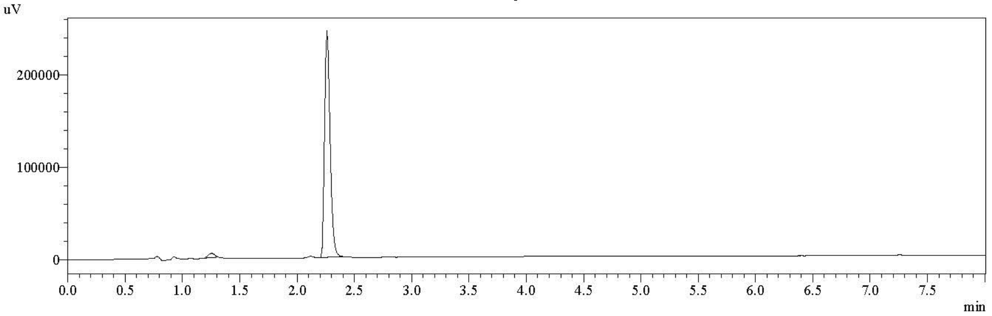

PDA Ch1 254nm 4nm

Peak#	Name	Ret. Time	Area	Area %
1		1.249	17766	2.151
2		2.257	808015	97.849

MS Spectrum Graph

#:1 Ret.Time:Averaged 2.340-2.360(Scan#:235-237)
Mass Peaks:11 Base Peak:270.05(167629) Polarity:Pos Segment1 - Event1

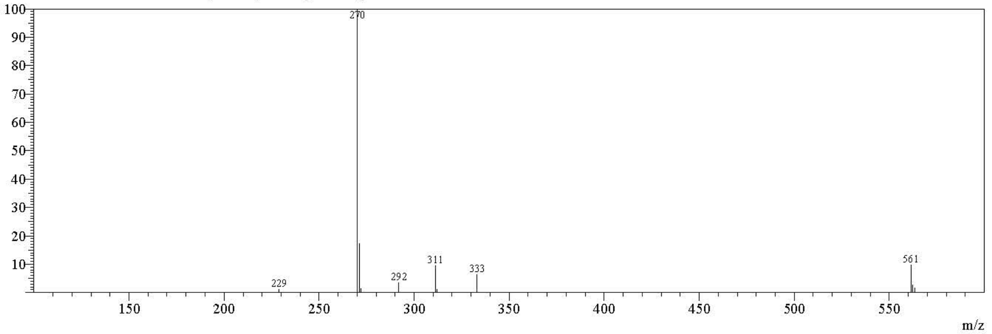

MS Spectrum Table

#:1 Ret.Time:
BG Mode:Calc 2.250<->2.510(226<->252)
Mass Peaks:11 Base Peak:270.05(167629) Polarity:Pos Segment1 - Event1

#	m/z	Abs.Inten.	Rel.Inten.	Charge	Polarity	Monoisotopic	#	m/z	Abs.Inten.	Rel.Inten.	Charge	Polarity	Monoisotopic
1	228.95	2033	1.21				7	312.20	1915	1.14			
2	270.05	167629	100.00				8	333.15	10548	6.29			
3	271.10	28885	17.23				9	561.30	16070	9.59			
4	272.15	2103	1.25				10	562.20	4684	2.79			
5	292.00	5834	3.48				11	563.35	2637	1.57			
6	311.10	15897	9.48										

Figure S4. LCMS spectrum of compound **4**.

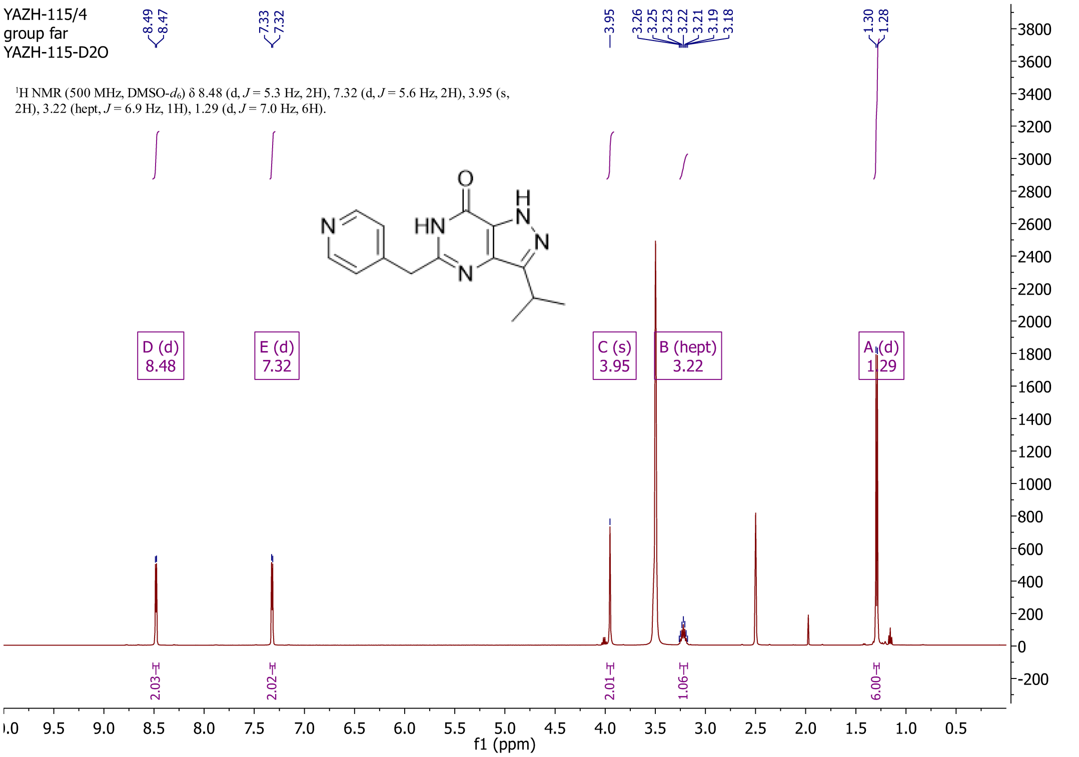

Figure S5. ^{1}H NMR spectrum of compound **4**.

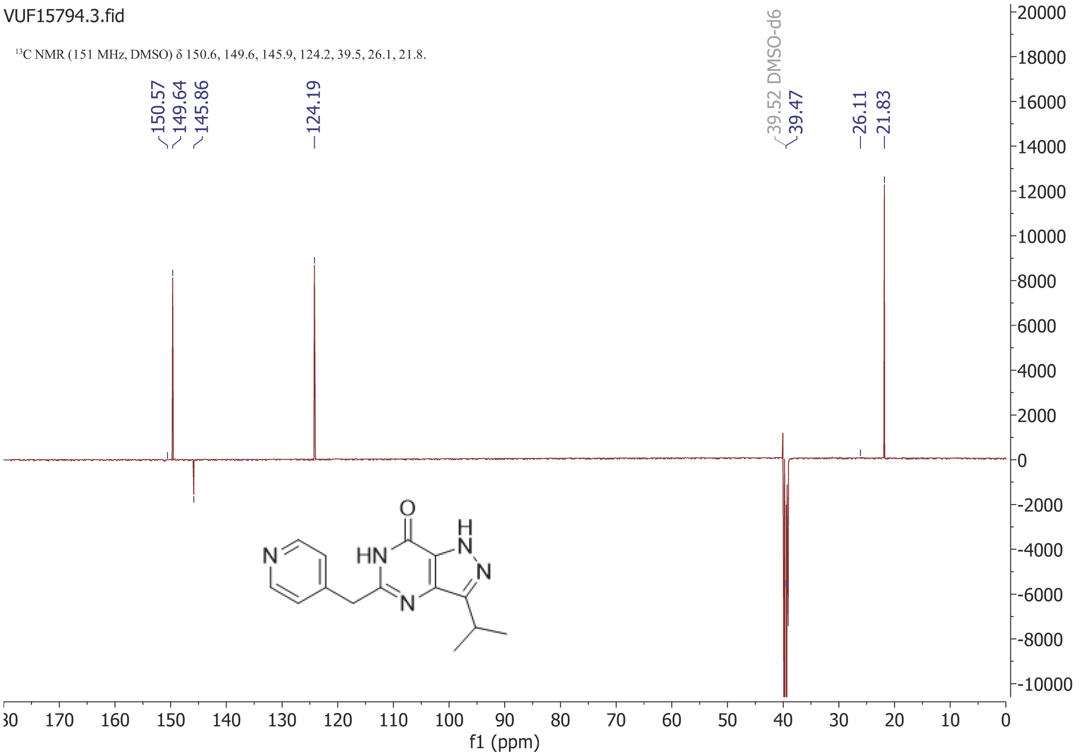

Figure S6. ^{13}C NMR spectrum of compound **4**.

Acquired by	: Admin
Date Acquired	: 18/5/2020 10:17:40 AM
Sample Name	: VUF14762
Sample ID	:
Tray#	: 1
Vial#	: 2
Injection Volume	: 5
Data File	: C:\LabSolutions\Data\2020\2020-wk21\VUF14762.lcd
Background File	: blanco18052020.lcd
Method File	: Method SCAN ACID standard.lcm
Report Format	: DefaultLCMS.lcr
Tuning File	: C:\LabSolutions\Tuning\Tuning-ESI-pos-neg01072015a.lct
Processed by	: Admin
Modified Date	: 18/5/2020 11:40:37 AM

PDA Graph

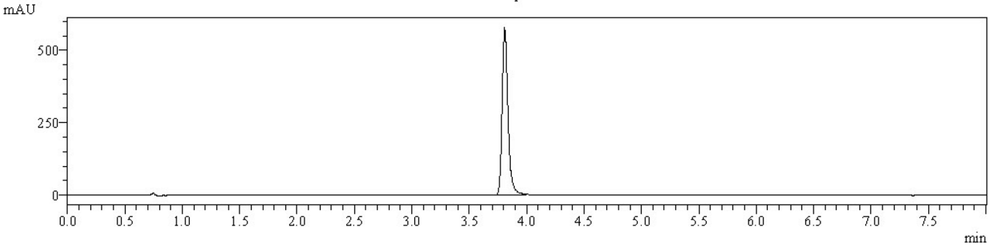

PeakTable

PDA Ch1 254nm 4nm

Peak#	Name	Ret. Time	Area	Area %
1		3.804	2001393	100.000

MS Chromatogram
VUF14762 C:\LabSolutions\Data\2020\2020-wk21\VUF14762.lcd

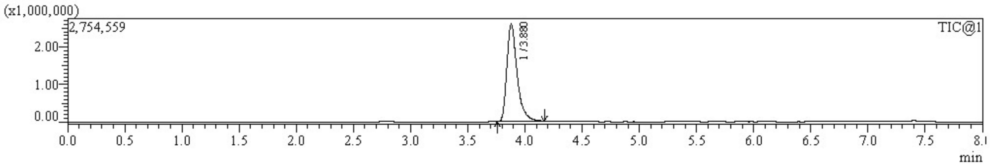

MS Spectrum Graph

#1 Ret.Time:Averaged 3.870-3.890(Scan#:388-390)
BG Mode:Calc 3.760<->4.170(377<->418)
Mass Peaks:15 Base Peak:285.15(1522515) Polarity:Pos Segment1 - Event1

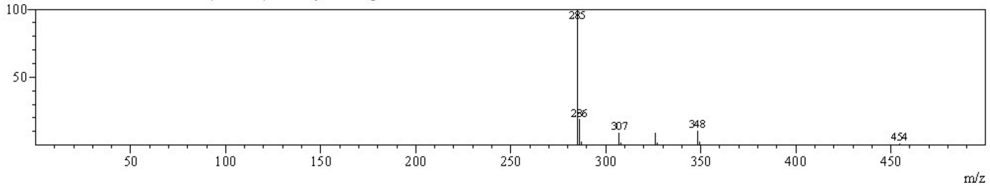

MS Spectrum Table

#:1 Ret.Time:
BG Mode:Calc 3.760<->4.170(377<->418)
Mass Peaks:15 Base Peak:285.15(1522515) Polarity:Pos Segment1 - Event1

#	m/z	Abs.Inten.	Rel.Inten.	Charge	Polarity	Monoisotopic
1	285.15	1522515	100.00			
2	286.15	289149	18.99			
3	287.15	29062	1.91			
4	307.15	136443	8.96			
5	308.15	26916	1.77			
6	326.20	128833	8.46			
7	327.20	27427	1.80			
8	348.15	158628	10.42			
9	349.25	36134	2.37			
10	454.55	16442	1.08			
11	591.40	46174	3.03			
12	592.40	15798	1.04			
13	596.40	26961	1.77			
14	596.70	48665	3.20			
15	597.75	22692	1.49			

Figure S7. LCMS spectrum of compound **5**.

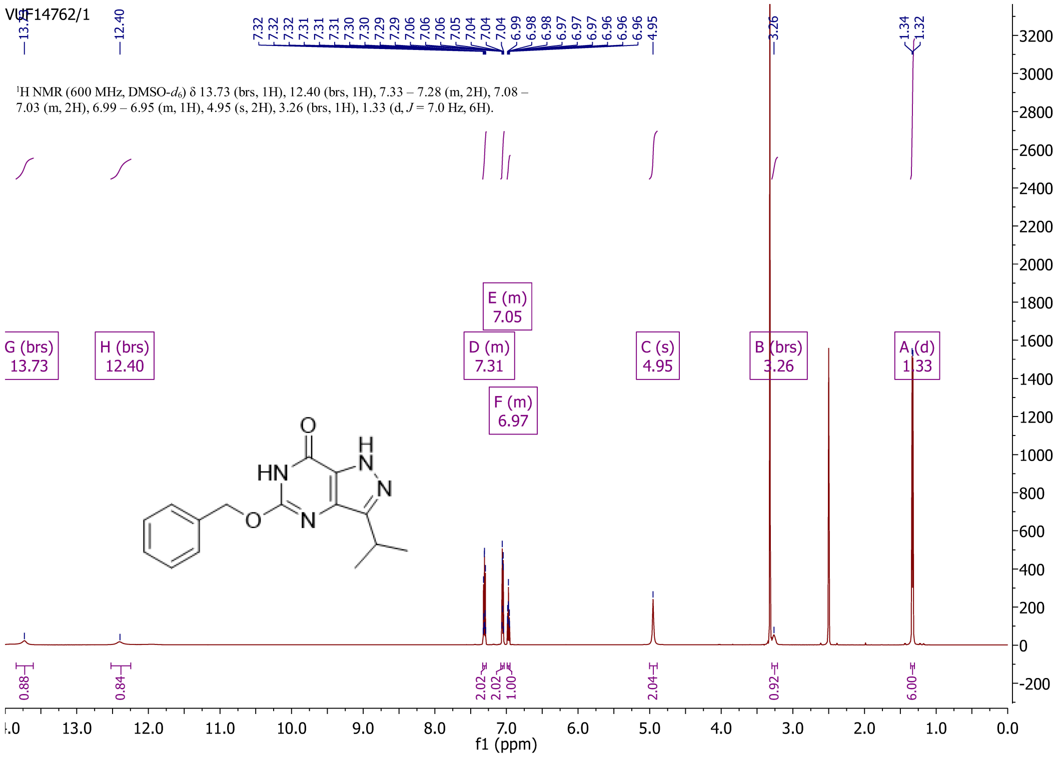

Figure S8. ^{1}H NMR spectrum of compound **5**.

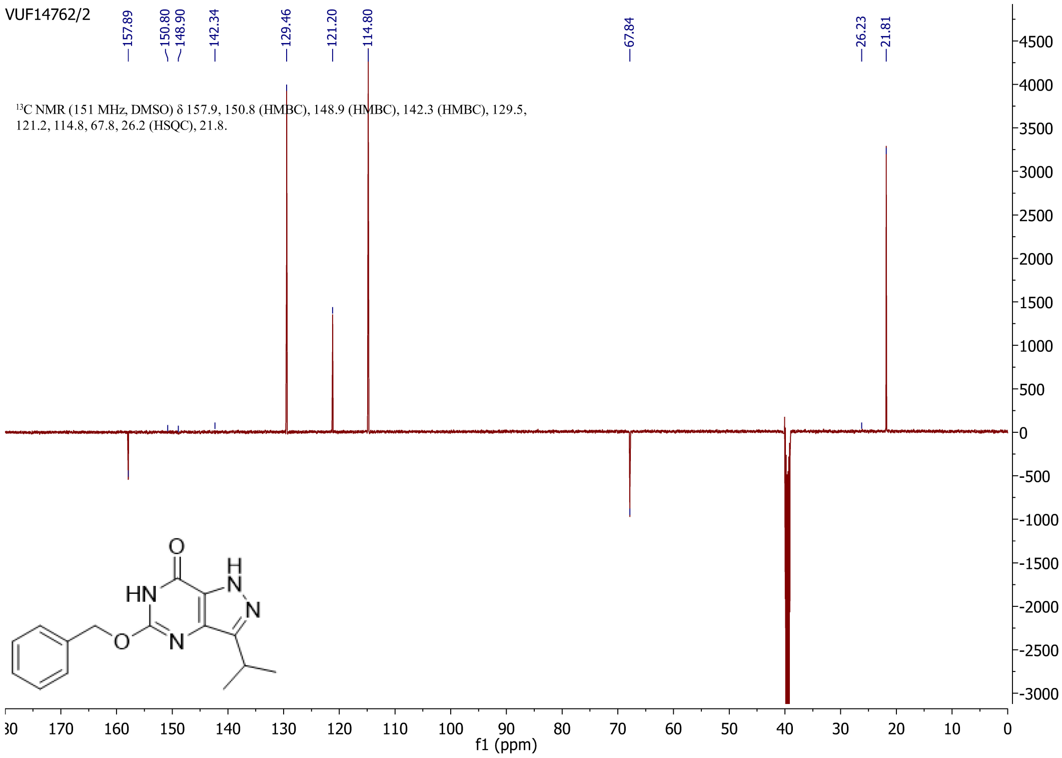

Figure S9. ^{13}C NMR spectrum of compound **5**.

Acquired by	: Admin
Date Acquired	: 7/14/2017 4:35:06 PM
Sample Name	: YAZH01-116
Sample ID	:
Tray#	: 1
Vial#	: 28
Injection Volume	: 10
Data File	: C:\LabSolutions\Data\2017\2017-wk28\YAZH01-116.lcd
Background File	: BLANCO_14072017.lcd
Method File	: Method SCAN ACID standard.lcm
Report Format	: DefaultLCMS.lcr
Tuning File	: C:\LabSolutions\Tuning\Tuning-ESI-pos-neg01072015a.lct
Processed by	: Admin
Modified Date	: 7/14/2017 5:24:01 PM

Chromatogram
YAZH01-116 C:\LabSolutions\Data\2017\2017-wk28\YAZH01-116.lcd

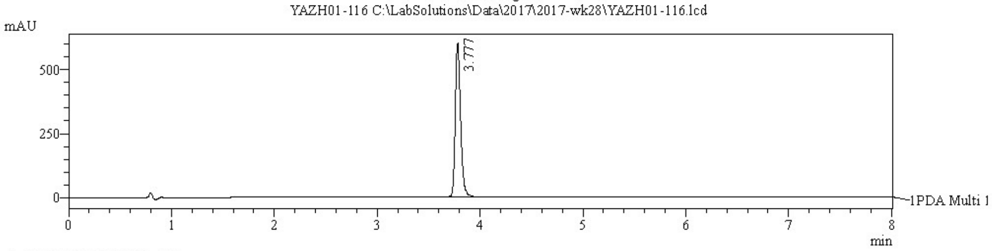

1 PDA Multi 1 / 254nm 4nm

PeakTable

PDA Ch1 254nm 4nm

Peak#	Name	Ret. Time	Area	Area %
1		3.777	2126142	100.000

MS Chromatogram
YAZH01-116 C:\LabSolutions\Data\2017\2017-wk28\YAZH01-116.lcd

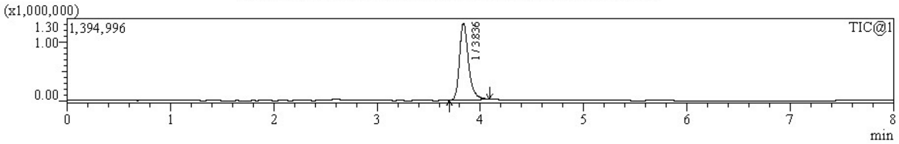

MS Spectrum Graph

#1 Ret.Time:Averaged 3.830-3.850(Scan#:384-386)
Mass Peaks:13 Base Peak:255.10(772560) Polarity:Pos Segment1 - Event1

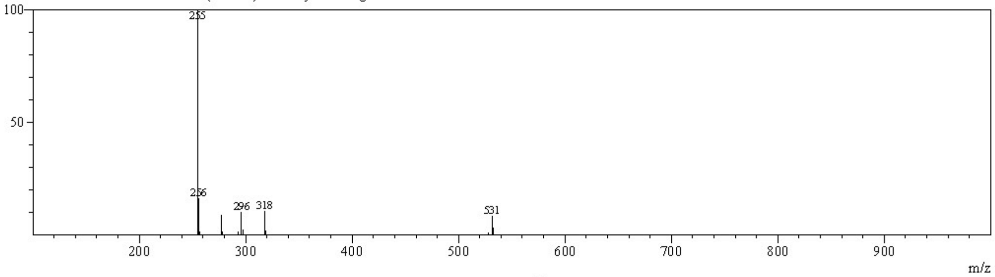

MS Spectrum Table

#:1 Ret.Time:
BG Mode:Calc 3.700<->4.090(371<->410)
Mass Peaks:13 Base Peak:255.10(772560) Polarity:Pos Segment1 - Event1

#	m/z	Abs.Inten.	Rel.Inten.	Charge	Polarity	Monoisotopic
1	255.10	772560	100.00			
2	256.10	125886	16.29			
3	257.10	10257	1.33			
4	277.10	66368	8.59			
5	278.10	9738	1.26			
6	293.00	11959	1.55			
7	296.10	76995	9.97			

#	m/z	Abs.Inten.	Rel.Inten.	Charge	Polarity	Monoisotopic
8	297.15	16084	2.08			
9	318.10	80834	10.46			
10	319.10	15800	2.05			
11	528.50	7861	1.02			
12	531.30	64127	8.30			
13	532.30	24020	3.11			

Figure S10. LCMS spectrum of compound **6**.

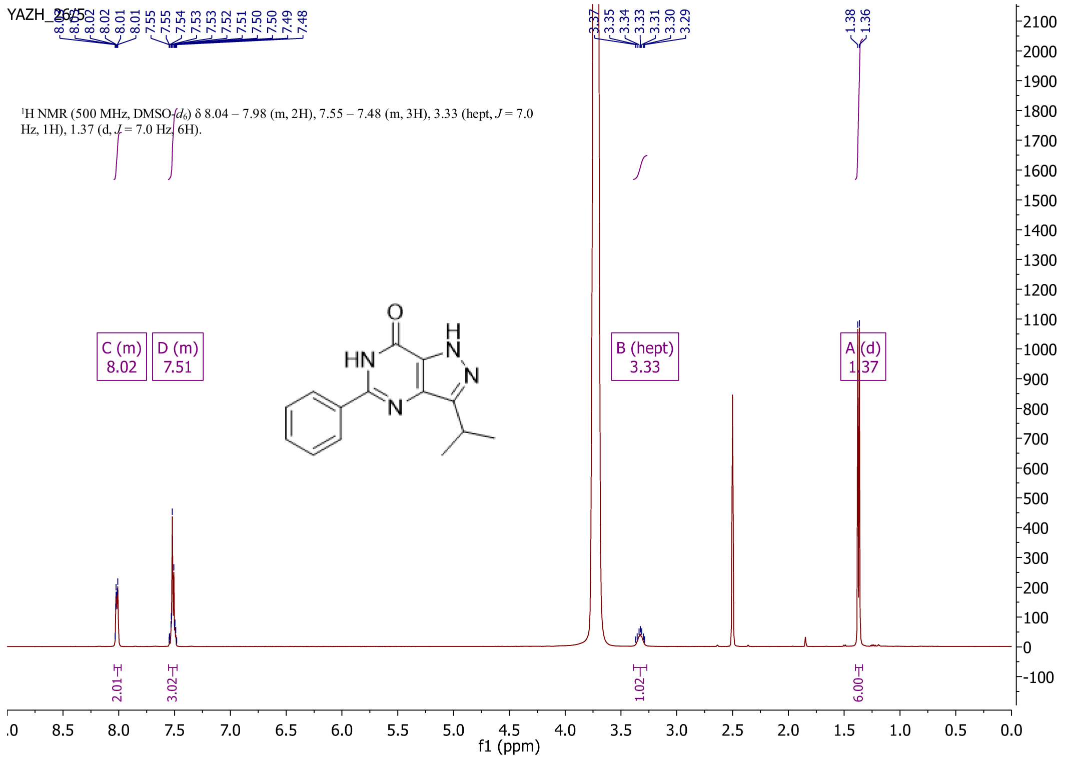

Figure S11. ^{1}H NMR spectrum of compound **6**.

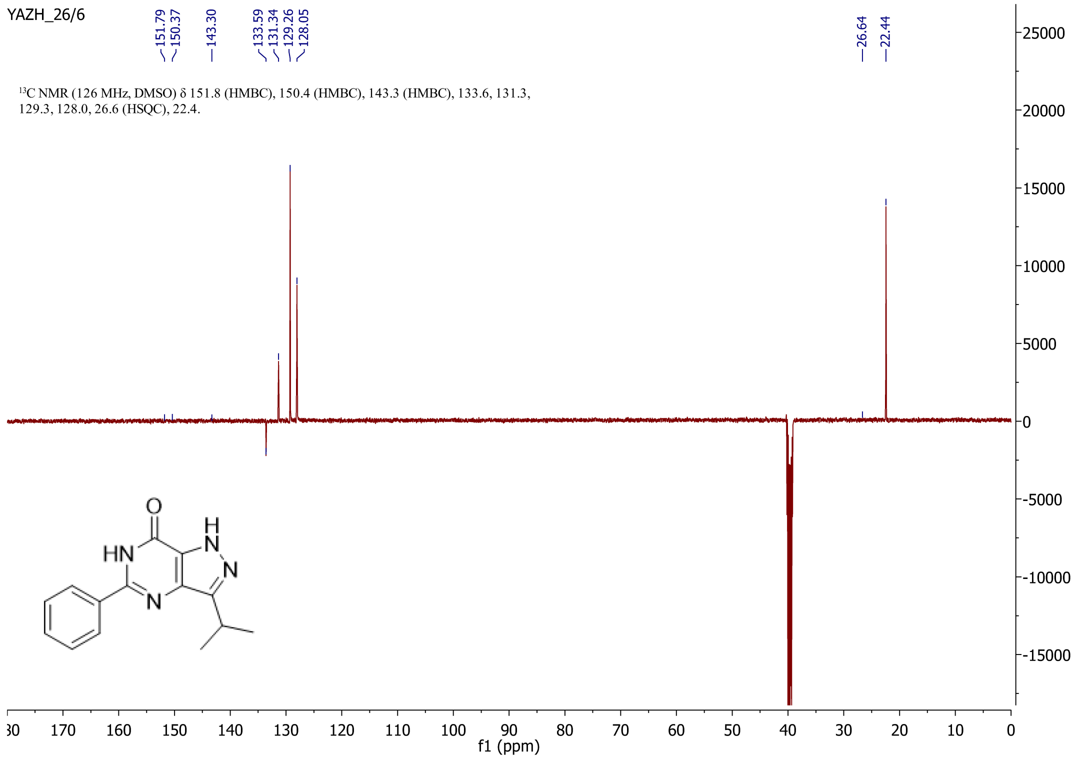

Figure S12. ^{13}C NMR spectrum of compound **6**.

Acquired by	: Admin
Date Acquired	: 12/3/2018 11:54:17 AM
Sample Name	: YAZH01-179
Sample ID	:
Tray#	: 1
Vial#	: 8
Injection Volume	: 5
Data File	: C:\LabSolutions\Data\2018\2018-wk11\YAZH01-179.lcd
Background File	: blanco_120318.lcd
Method File	: Method SCAN ACID standard.lcm
Report Format	: DefaultLCMS.lcr
Tuning File	: C:\LabSolutions\Tuning\Tuning-ESI-pos-neg01072015a.lct
Processed by	: Admin
Modified Date	: 7/6/2019 5:56:39 PM

PDA Graph

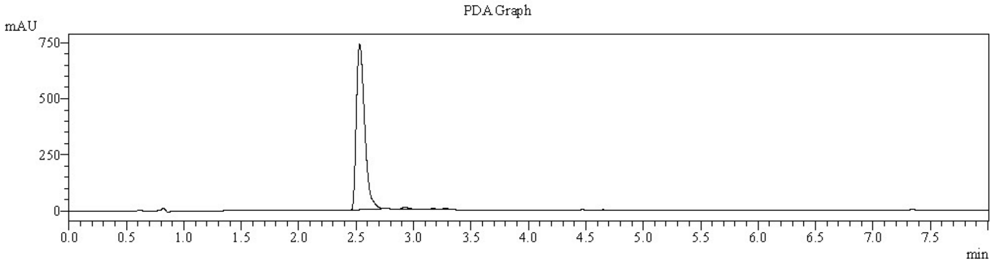

PeakTable

PDA Ch1 254nm 4nm

Peak#	Name	Ret. Time	Area	Area %
1		2.526	3685980	98.260
2		2.922	37216	0.992
3		3.163	13353	0.356
4		3.272	14686	0.392

MS Chromatogram
YAZH01-179 C:\LabSolutions\Data\2018\2018-wk11-20\2018-wk11\YAZH01-179.lcd

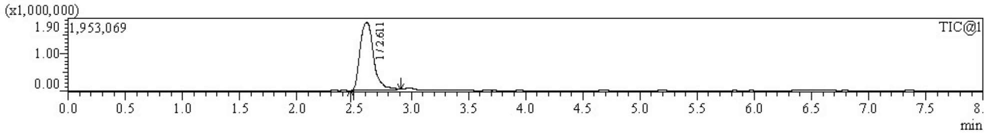

MS Spectrum Graph

#1 Ret.Time:Averaged 2.600-2.620(Scan#:261-263)
BG Mode:Calc 2.470<->2.910(248<->292)
Mass Peaks:8 Base Peak:353.15(1236663) Polarity:Pos Segment1 - Event1

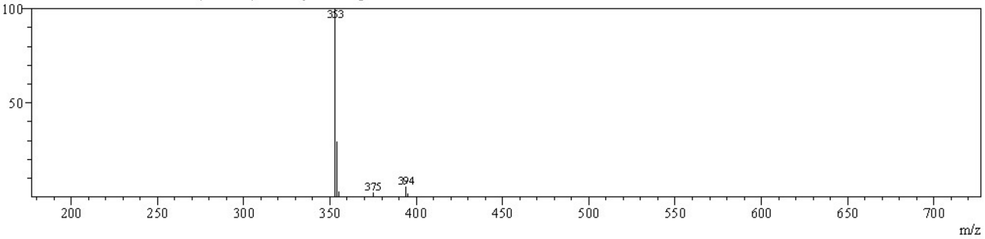

MS Spectrum Table

#:1 Ret.Time:
BG Mode:Calc 2.470<->2.910(248<->292)
Mass Peaks:8 Base Peak:353.15(1236663) Polarity:Pos Segment1 - Event1

#	m/z	Abs.Inten.	Rel.Inten.	Charge	Polarity	Monoisotopic
1	176.95	20460	1.65			
2	353.15	1236663	100.00			
3	354.15	365052	29.52			
4	355.15	31125	2.52			

#	m/z	Abs.Inten.	Rel.Inten.	Charge	Polarity	Monoisotopic
5	375.20	26493	2.14			
6	394.20	66244	5.36			
7	395.20	19763	1.60			
8	727.35	20062	1.62			

Figure S13. LCMS spectrum of compound **7**.

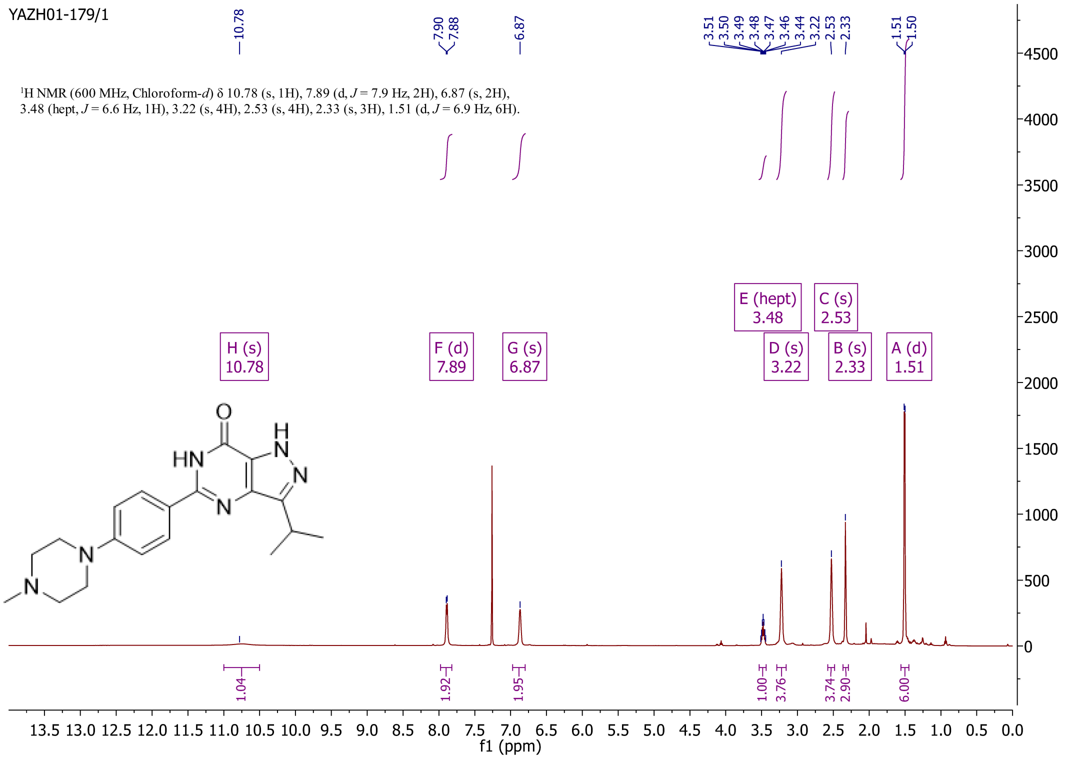

Figure S14. ^{1}H NMR spectrum of compound **7**.

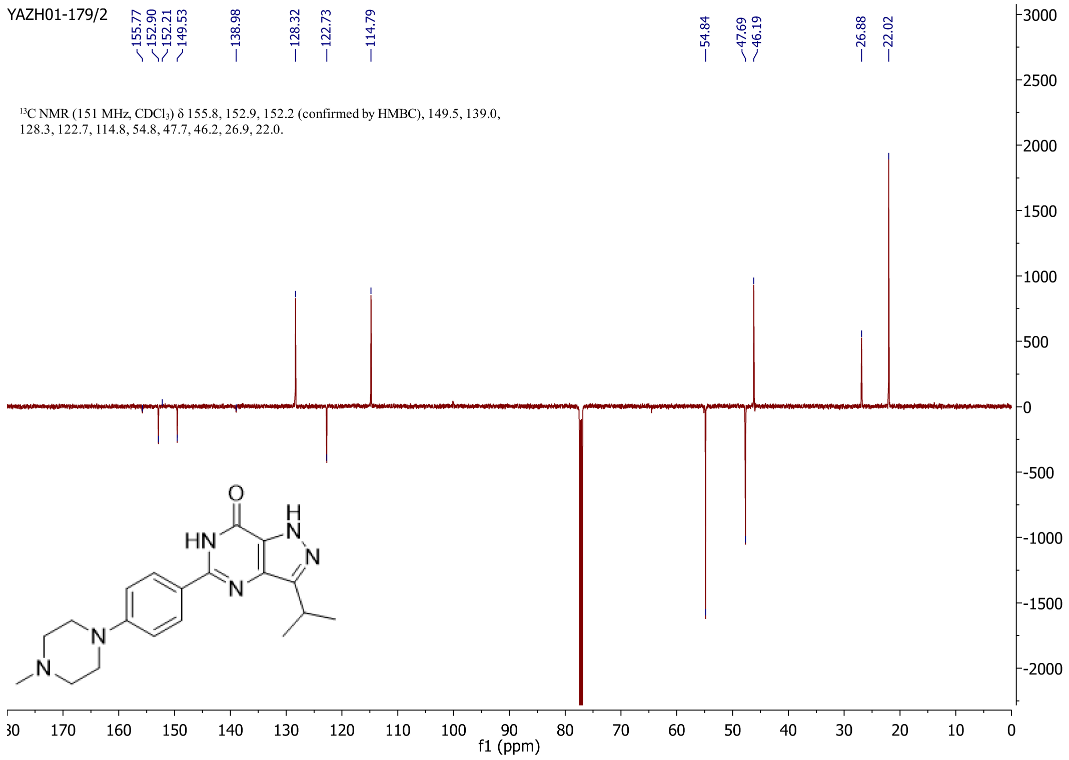

Figure S15. ^{13}C NMR spectrum of compound **7**.

Acquired by : Admin
Date Acquired : 18/6/2019 3:48:26 PM
Sample Name : VUF16503
Sample ID :
Tray# : 1
Vial# : 36
Injection Volume : 5
Data File : C:\LabSolutions\Data\2019\2019-wk25\VUF16503.lcd
Background File : blanco 18062019.lcd
Method File : Method SCAN ACID standard.lcm
Report Format : DefaultLCMS.lcr
Tuning File : C:\LabSolutions\Tuning\Tuning-ESI-pos-neg01072015a.lct
Processed by : Admin
Modified Date : 18/6/2019 4:24:20 PM

PDA Graph

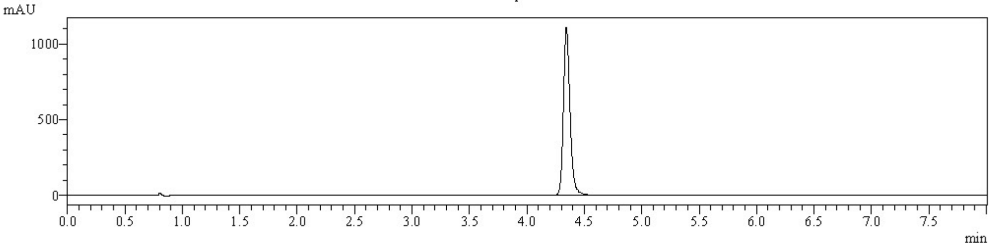

PeakTable

PDA Ch1 254nm 4nm

Peak#	Name	Ret. Time	Area	Area%
1		4.339	4313859	100.000

MS Chromatogram
VUF16503 C:\LabSolutions\Data\2019\2019-wk25\VUF16503.lcd

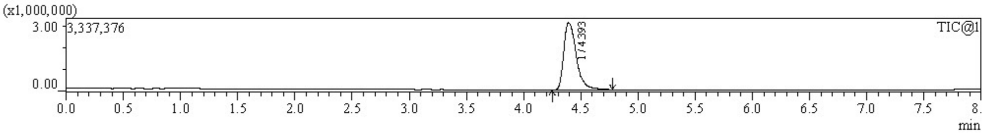

MS Spectrum Graph

#:1 Ret.Time:Averaged 4.380-4.400(Scan#:439-441)
BG Mode:Calc 4.250<->4.780(426<->479)
Mass Peaks:8 Base Peak:338.10(2315029) Polarity:Pos Segment1 - Event1

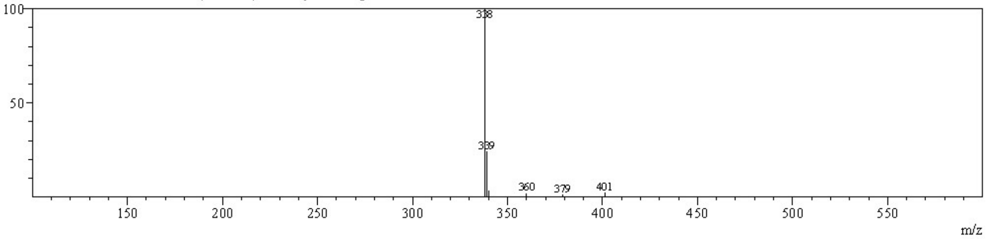

MS Spectrum Table

#:1 Ret.Time:
BG Mode:Calc 4.250<->4.780(426<->479)
Mass Peaks:8 Base Peak:338.10(2315029) Polarity:Pos Segment1 - Event1

#	m/z	Abs.Inten.	Rel.Inten.	Charge	Polarity	Monoisotopic
1	338.10	2315029	100.00			
2	339.15	558399	24.12			
3	340.15	69830	3.02			
4	360.05	44771	1.93			
5	379.15	26705	1.15			
6	401.15	51029	2.20			
7	697.40	39606	1.71			
8	702.95	24657	1.07			

Figure S16. LCMS spectrum of compound **8**.

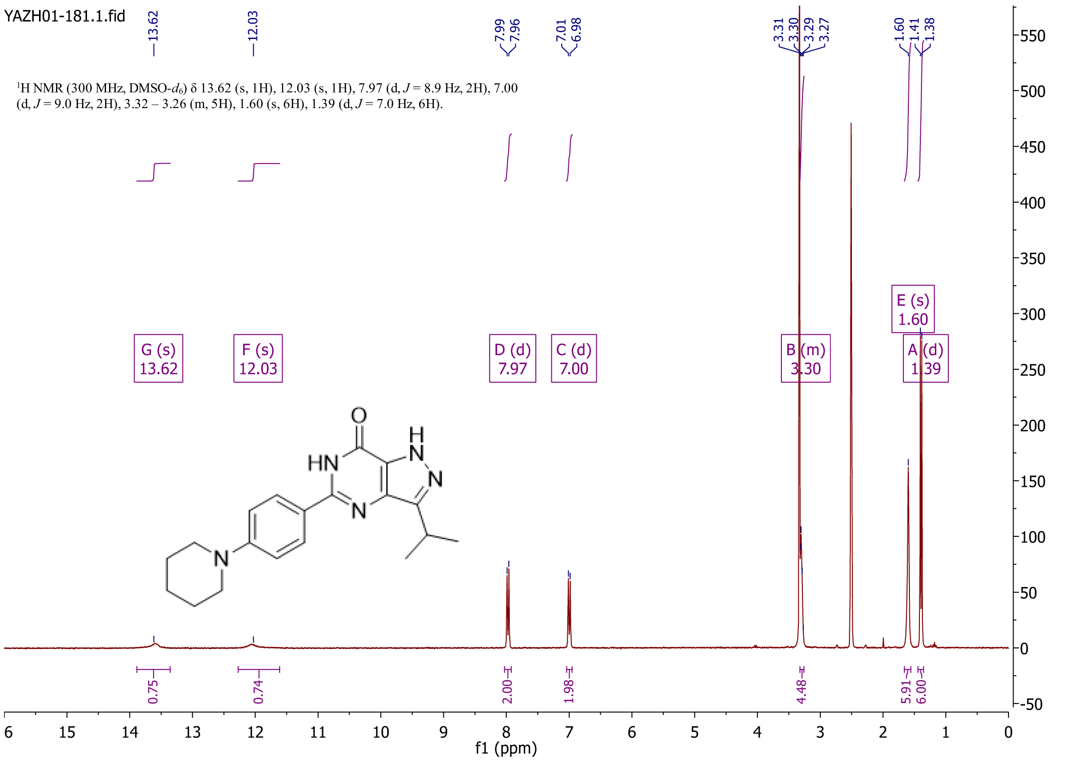

Figure S17. ^{1}H NMR spectrum of compound **8**.

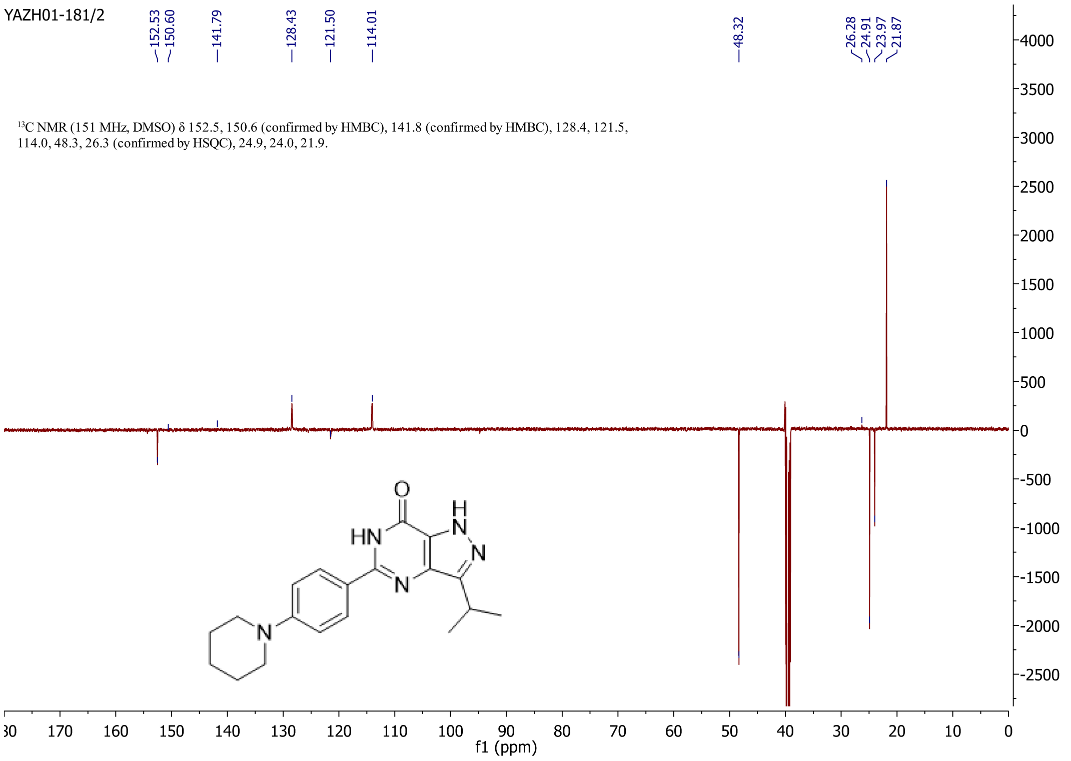

Figure S18. ^{13}C NMR spectrum of compound **8**.

Acquired by	: Admin
Date Acquired	: 5/17/2016 12:43:55 PM
Sample Name	: YAZH-131
Sample ID	:
Tray#	: 1
Vial#	: 20
Injection Volume	: 1
Data File	: C:\LabSolutions\Data\2016 - wk20\YAZH-131.lcd
Background File	: blanco 17052016.lcd
Method File	: Method SCAN ACID standard.lcm
Report Format	: DefaultLCMS.lcr
Tuning File	: C:\LabSolutions\Tuning\Tuning-ESI-pos-neg01072015a.lct
Processed by	: Admin
Modified Date	: 5/17/2016 2:59:15 PM

PDA Graph

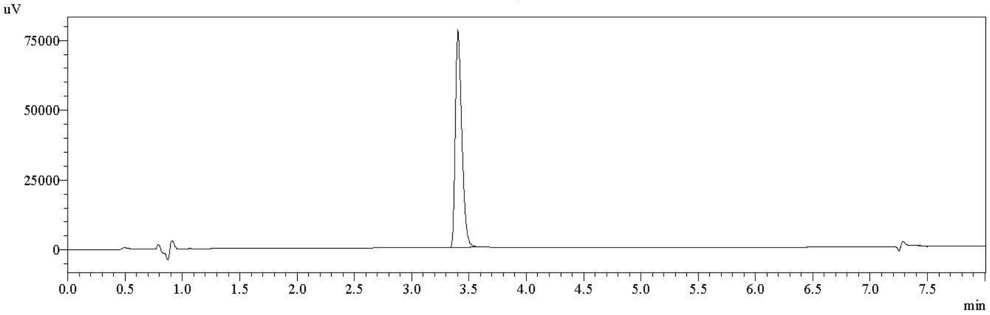

PDA Ch1 254nm 4nm

Peak#	Name	Ret. Time	Area	Area%
1		3.399	307369	100.000

MS Spectrum Graph

#:1 Ret.Time:Averaged 3.470-3.490(Scan#:348-350)
Mass Peaks:11 Base Peak:262.00(159271) Polarity:Pos Segment1 - Event1

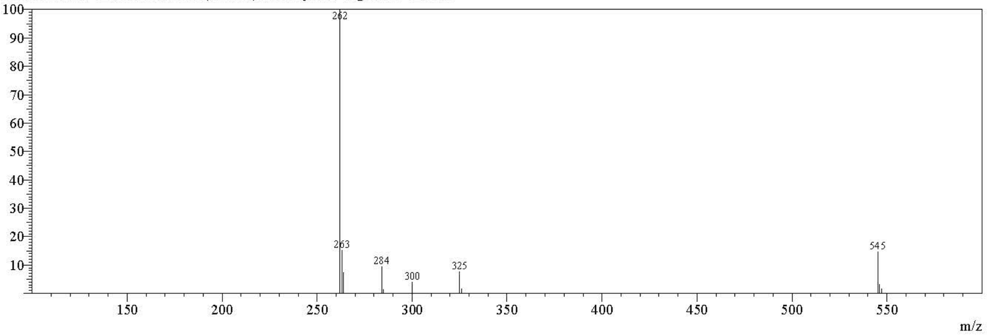

MS Spectrum Table

#:1 Ret.Time:
BG Mode:Calc 3.370<->3.640(338<->365)
Mass Peaks:11 Base Peak:262.00(159271) Polarity:Pos Segment1 - Event1

#	m/z	Abs.Inten.	Rel.Inten.	Charge	Polarity	Monoisotopic
1	262.00	159271	100.00			
2	263.05	24117	15.14			
3	264.00	11613	7.29			
4	283.95	15021	9.43			
5	285.05	2316	1.45			
6	300.05	6172	3.88			

#	m/z	Abs.Inten.	Rel.Inten.	Charge	Polarity	Monoisotopic
7	325.00	12159	7.63			
8	326.10	2444	1.53			
9	545.05	23434	14.71			
10	546.10	5129	3.22			
11	547.20	2335	1.47			

Figure S19. LCMS spectrum of compound **9**.

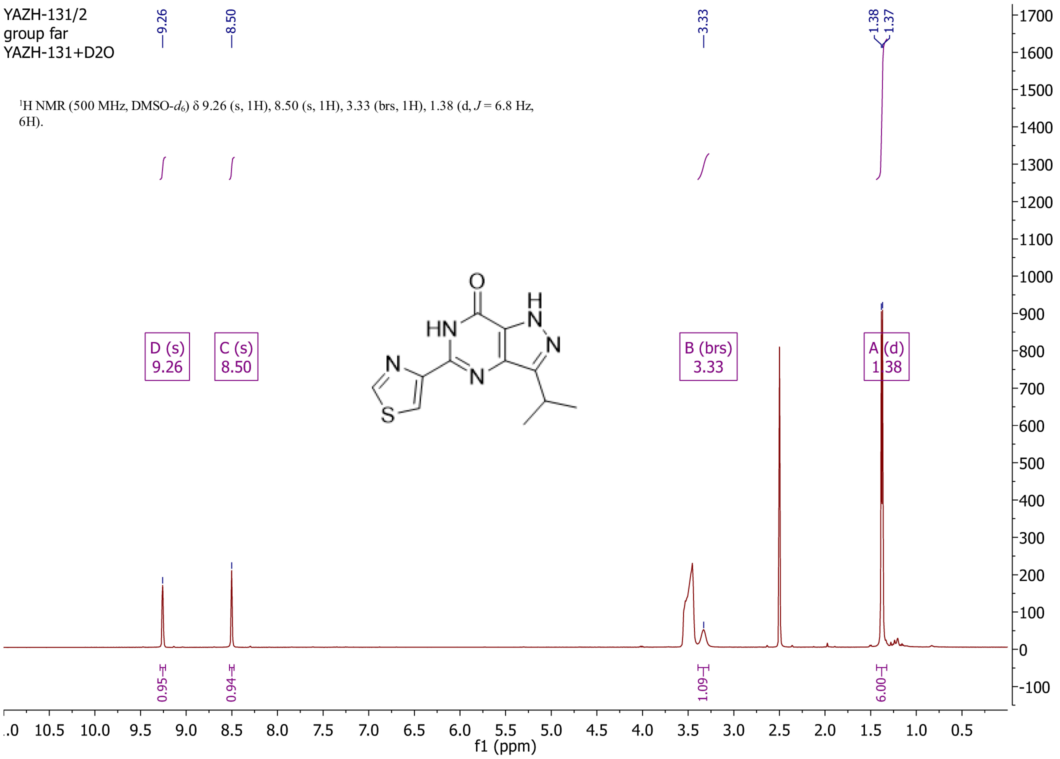

Figure S20. ^{1}H NMR spectrum of compound **9**.

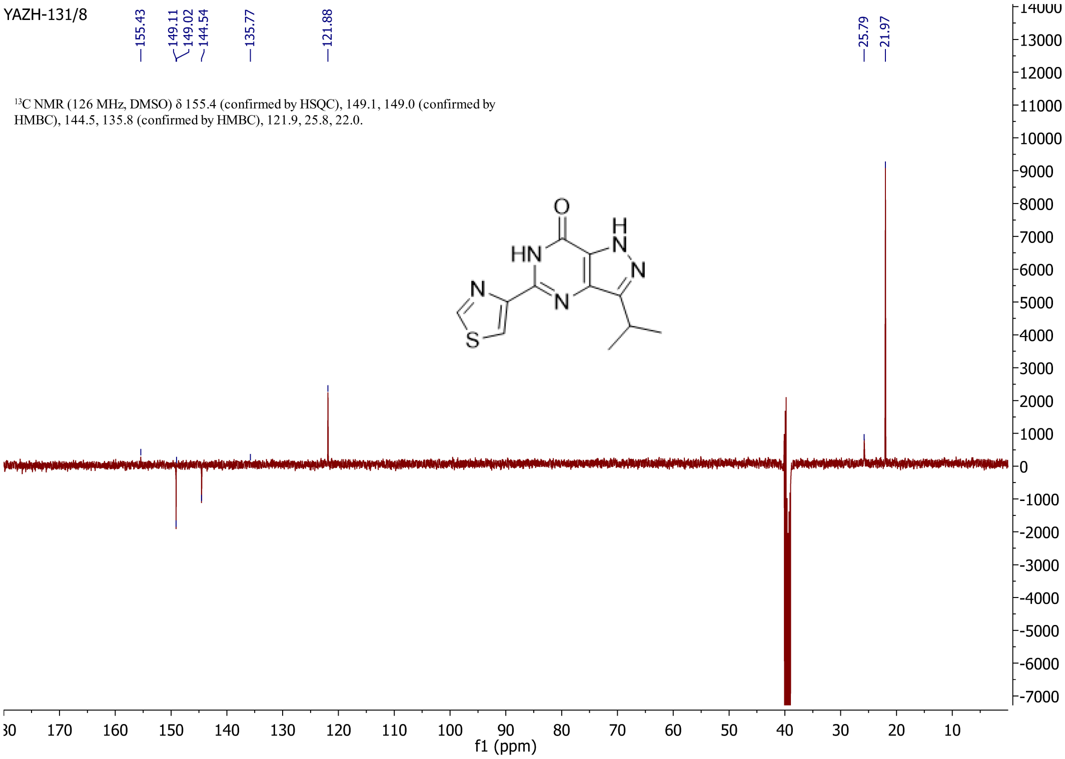

Figure S21. ^{13}C NMR spectrum of compound **9**.

Acquired by	: Admin
Date Acquired	: 4/5/2016 8:23:28 AM
Sample Name	: YAZH-127-1
Sample ID	:
Tray#	: 1
Vial#	: 33
Injection Volume	: 1
Data File	: C:\LabSolutions\Data\2016 - wk18\YAZH-127-1.lcd
Background File	: blanco04052016.lcd
Method File	: Method SCAN ACID standard.lcm
Report Format	: DefaultLCMS.lcr
Tuning File	: C:\LabSolutions\Tuning\Tuning-ESI-pos-neg01072015a.lct
Processed by	: Admin
Modified Date	: 17/11/2020 2:46:39 PM

PDA Graph

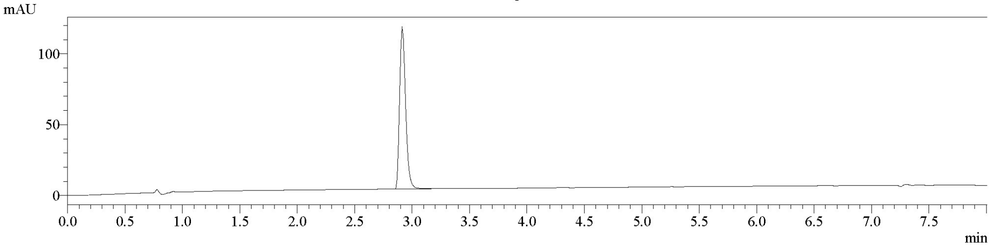

PeakTable

PDA Ch1 254nm 4nm

Peak#	Name	Ret. Time	Area	Area %
1		2.909	417185	100.000

MS Chromatogram
YAZH-127-1 C:\LabSolutions\Data\2016\2016 - wk11-wk20\2016 - wk18\YAZH-127-1.lcd

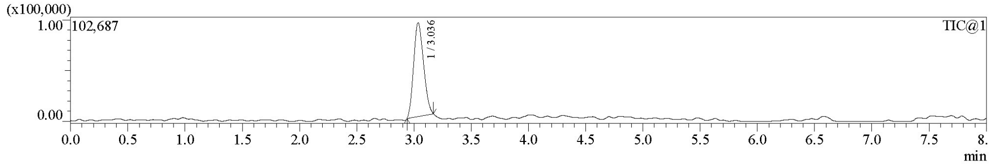

MS Spectrum Graph

#:1 Ret.Time:Averaged 3.030-3.050(Scan#:304-306)
BG Mode:Calc 2.940<->3.170(295<->318)
Mass Peaks:15 Base Peak:286.05(56359) Polarity:Pos Segment1 - Event1

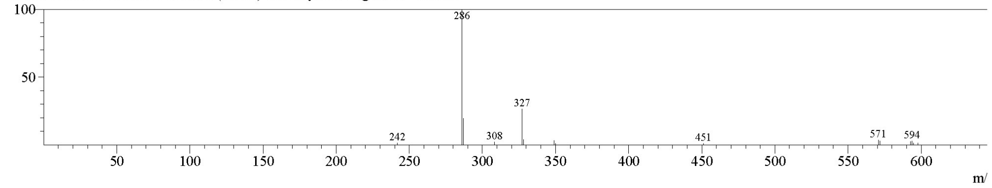

MS Spectrum Table

#:1 Ret.Time:
BG Mode:Calc 2.940<->3.170(295<->318)
Mass Peaks:15 Base Peak:286.05(56359) Polarity:Pos Segment1 - Event1

#	m/z	Abs.Inten.	Rel.Inten.	Charge	Polarity	Monoisotopic
1	241.90	759	1.35			
2	286.05	56359	100.00			
3	287.15	11052	19.61			
4	308.25	1162	2.06			
5	327.05	14987	26.59			
6	328.10	2223	3.94			
7	349.10	1884	3.34			
8	350.10	731	1.30			
9	451.00	731	1.30			
10	570.70	1990	3.53			
11	571.70	1742	3.09			
12	592.70	1462	2.59			
13	593.70	1612	2.86			
14	594.65	771	1.37			
15	597.70	810	1.44			

Figure S22. LCMS spectrum of compound **10**.

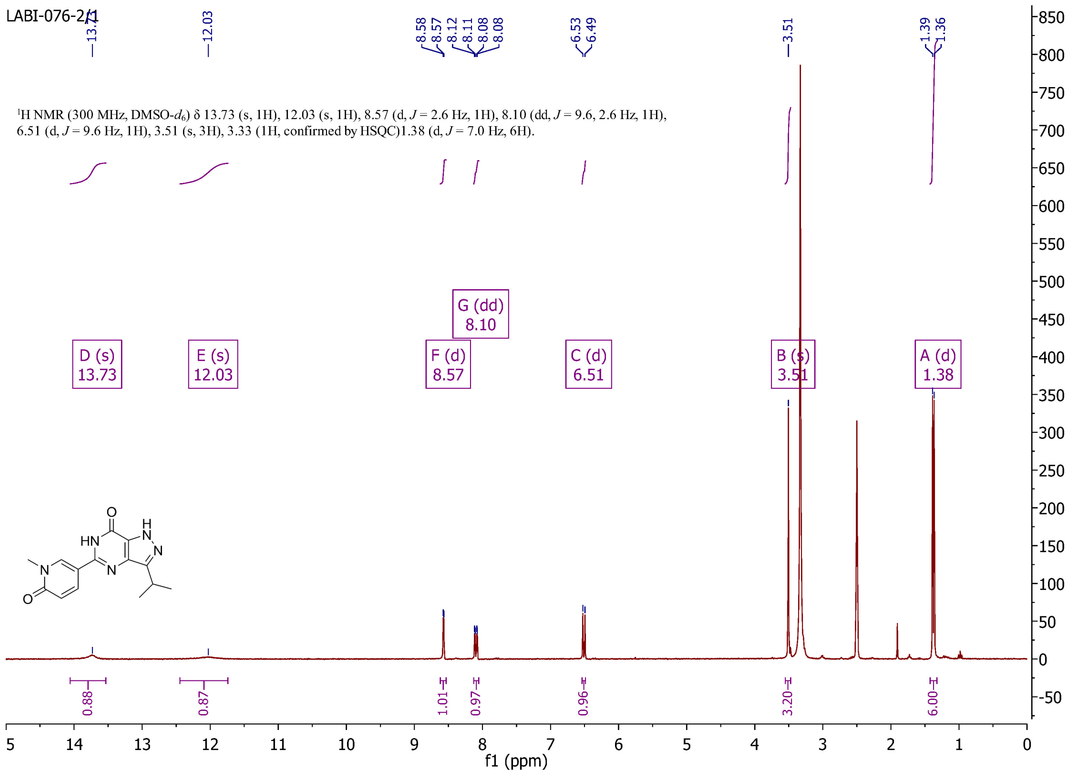

Figure S23. ^{1}H NMR spectrum of compound **10**.

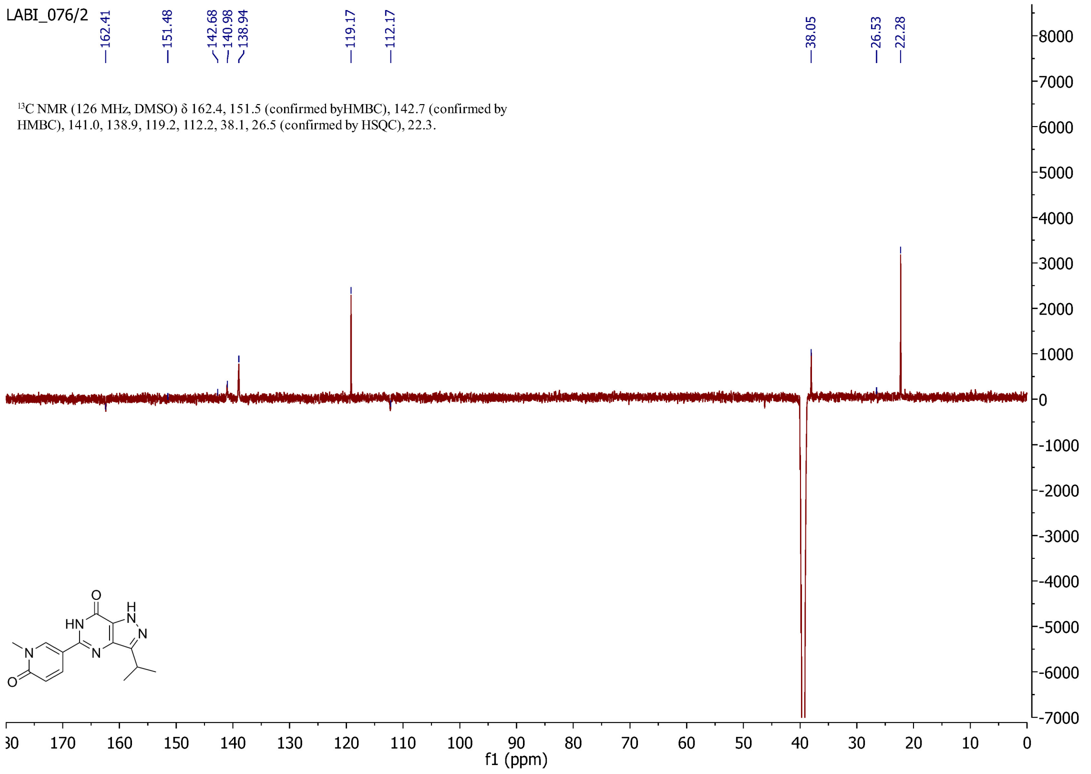

Figure 24. ^{13}C NMR spectrum of compound **10**.

Acquired by	: Admin
Date Acquired	: 5/13/2016 6:30:07 PM
Sample Name	: YAZH-124
Sample ID	:
Tray#	: 1
Vial#	: 30
Injection Volume	: 1
Data File	: C:\LabSolutions\Data\2016 - wk19\YAZH-124.lcd
Background File	: blanco 13052016.lcd
Method File	: Method SCAN ACID standard.lcm
Report Format	: DefaultLCMS.lcr
Tuning File	: C:\LabSolutions\Tuning\Tuning-ESI-pos-neg01072015a.lct
Processed by	: Admin
Modified Date	: 5/17/2016 8:53:23 AM

PDA Graph

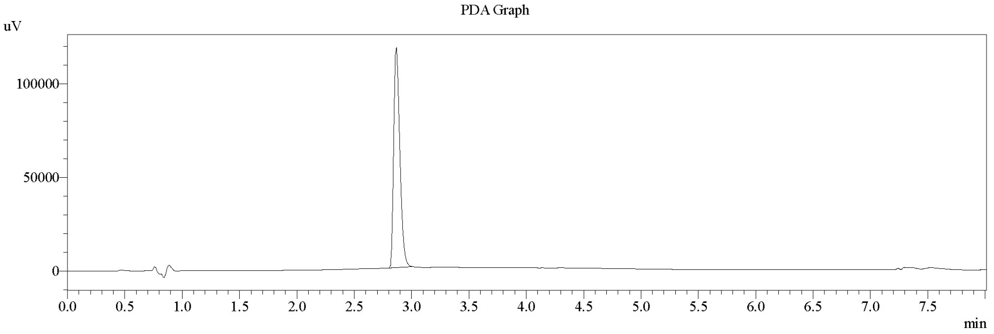

PDA Ch1 254nm 4nm

Peak#	Name	Ret. Time	Area	Area %
1		2.861	435212	100.000

MS Spectrum Graph

#:1 Ret.Time:Averaged 2.940-2.960(Scan#:295-297)
Mass Peaks:12 Base Peak:286.05(345547) Polarity:Pos Segment1 - Event1

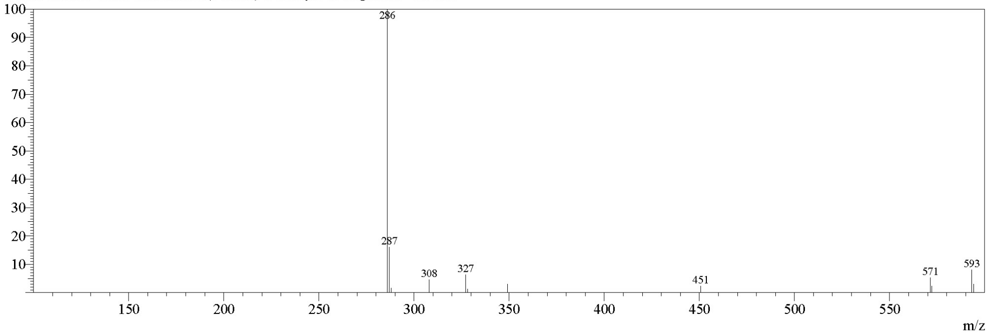

MS Spectrum Table

#:1 Ret.Time:
BG Mode:Calc 2.840<->3.140(285<->315)
Mass Peaks:12 Base Peak:286.05(345547) Polarity:Pos Segment1 - Event1

#	m/z	Abs.Inten.	Rel.Inten.	Charge	Polarity	Monoisotopic	#	m/z	Abs.Inten.	Rel.Inten.	Charge	Polarity	Monoisotopic
1	286.05	345547	100.00				7	349.10	10212	2.96			
2	287.05	55580	16.08				8	450.65	8293	2.40			
3	288.05	5645	1.63				9	571.30	18483	5.35			
4	308.00	15951	4.62				10	572.25	8141	2.36			
5	327.10	22082	6.39				11	593.20	27933	8.08			
6	328.05	4597	1.33				12	594.15	10147	2.94			

Figure S25. LCMS spectrum of compound **11**.

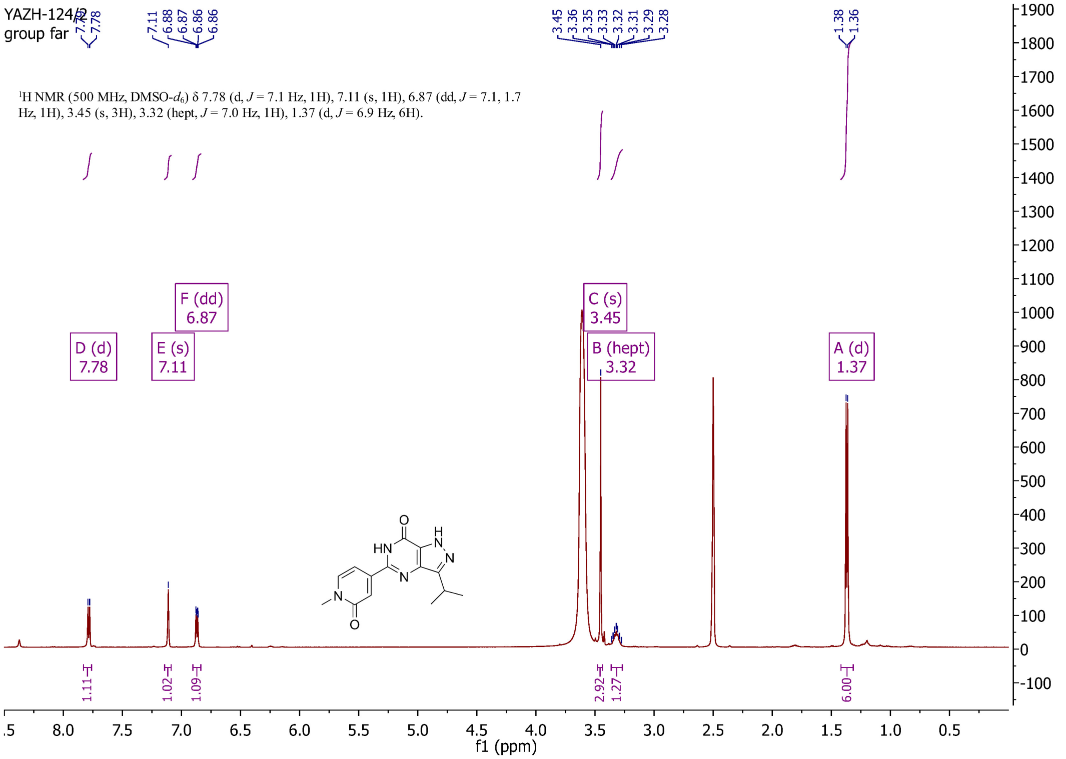

Figure S26. ^{1}H NMR spectrum of compound **11**.

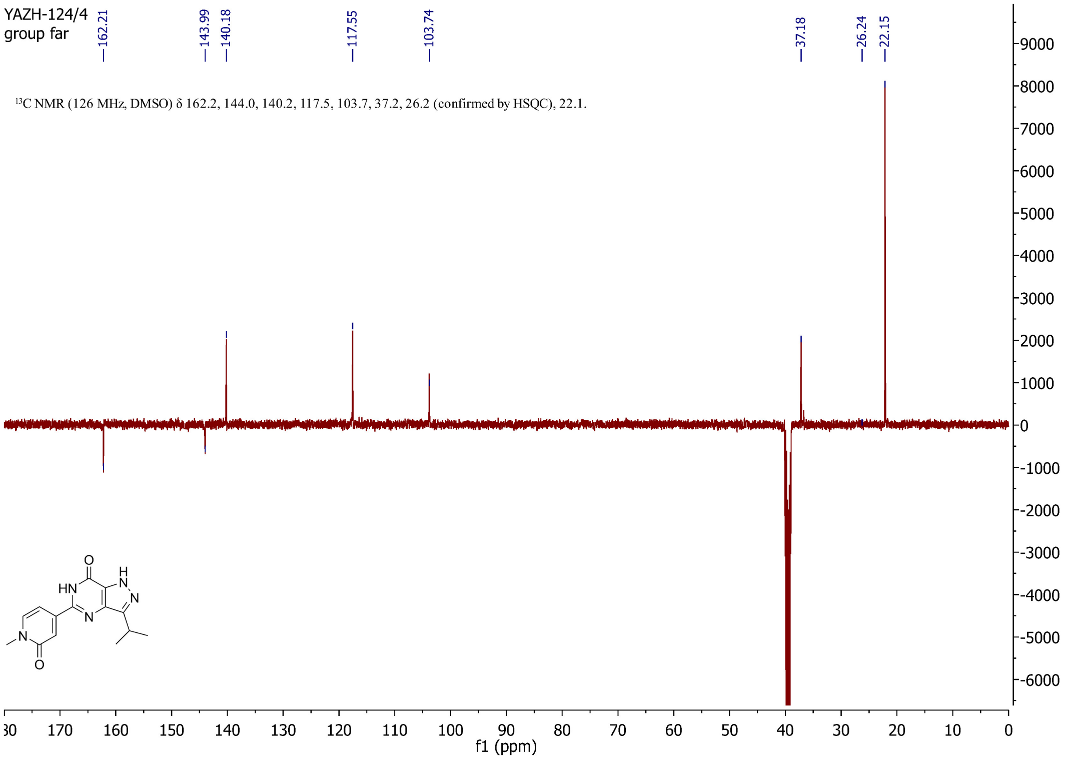

Figure S27. ^{13}C NMR spectrum of compound **11**.

Acquired by	: Admin
Date Acquired	: 13/12/2017 1:15:35 PM
Sample Name	: YAZH01-177-2
Sample ID	:
Tray#	: 1
Vial#	: 14
Injection Volume	: 3
Data File	: C:\LabSolutions\Data\2017\2017-wk50\YAZH01-177-2.lcd
Background File	: blanco 13122017.lcd
Method File	: Method SCAN ACID standard.lcm
Report Format	: DefaultLCMS.lcr
Tuning File	: C:\LabSolutions\Tuning\Tuning-ESI-pos-neg01072015a.lct
Processed by	: Admin
Modified Date	: 13/12/2017 3:51:59 PM

Chromatogram
YAZH01-177-2 C:\LabSolutions\Data\2017\2017-wk50\YAZH01-177-2.lcd

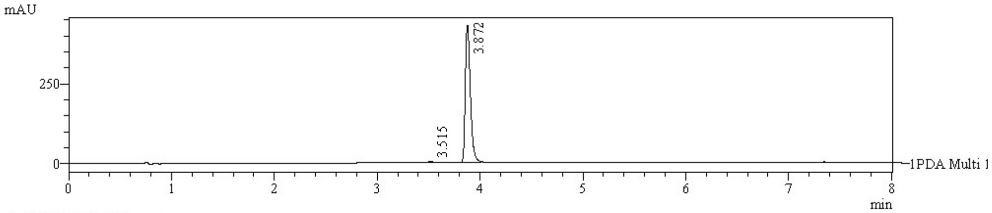

1 PDA Multi 1 / 254nm 4nm
PDA Ch1 254nm 4nm

Peak#	Name	Ret. Time	Area	Area%
1		3.515	10088	0.690
2		3.872	1451224	99.310

MS Chromatogram
YAZH01-177-2 C:\LabSolutions\Data\2017\2017-wk50\YAZH01-177-2.lcd

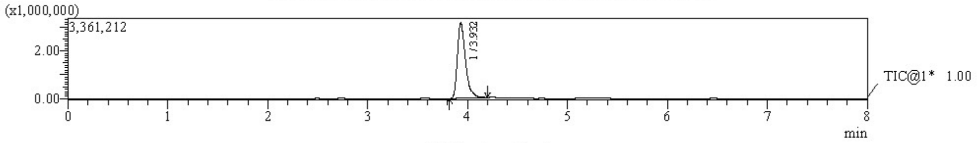

MS Spectrum Graph
#1 Ret.Time:Averaged 3.920-3.940(Scan#:393-395)
Mass Peaks:9 Base Peak:283.10(2292484) Polarity:Pos Segment1 - Event1

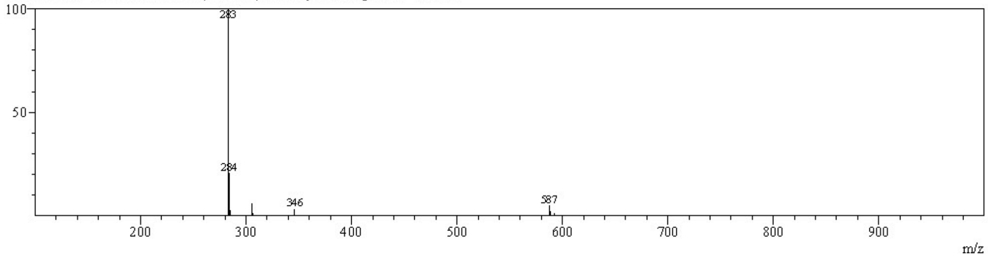

MS Spectrum Table
#:1 Ret.Time:
BG Mode:Calc 3.820<->4.200(383<->421)
Mass Peaks:9 Base Peak:283.10(2292484) Polarity:Pos Segment1 - Event1

#	m/z	Abs.Inten.	Rel.Inten.	Charge	Polarity	Monoisotopic
1	283.10	2292484	100.00			
2	284.10	466861	20.36			
3	285.15	56524	2.47			
4	305.10	128110	5.59			
5	306.10	23597	1.03			

#	m/z	Abs.Inten.	Rel.Inten.	Charge	Polarity	Monoisotopic
6	346.15	72424	3.16			
7	587.30	112077	4.89			
8	588.30	42963	1.87			
9	592.40	30587	1.33			

Figure S28. LCMS spectrum of compound **12**.

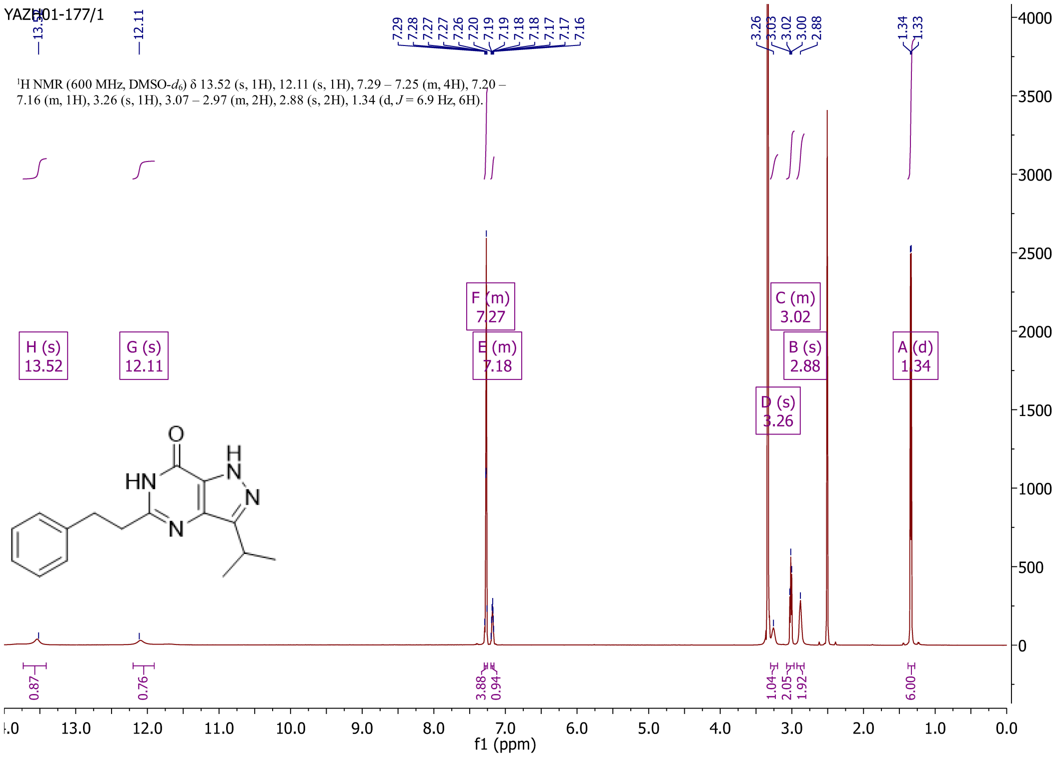

Figure S29. ¹H NMR spectrum of compound **12**.

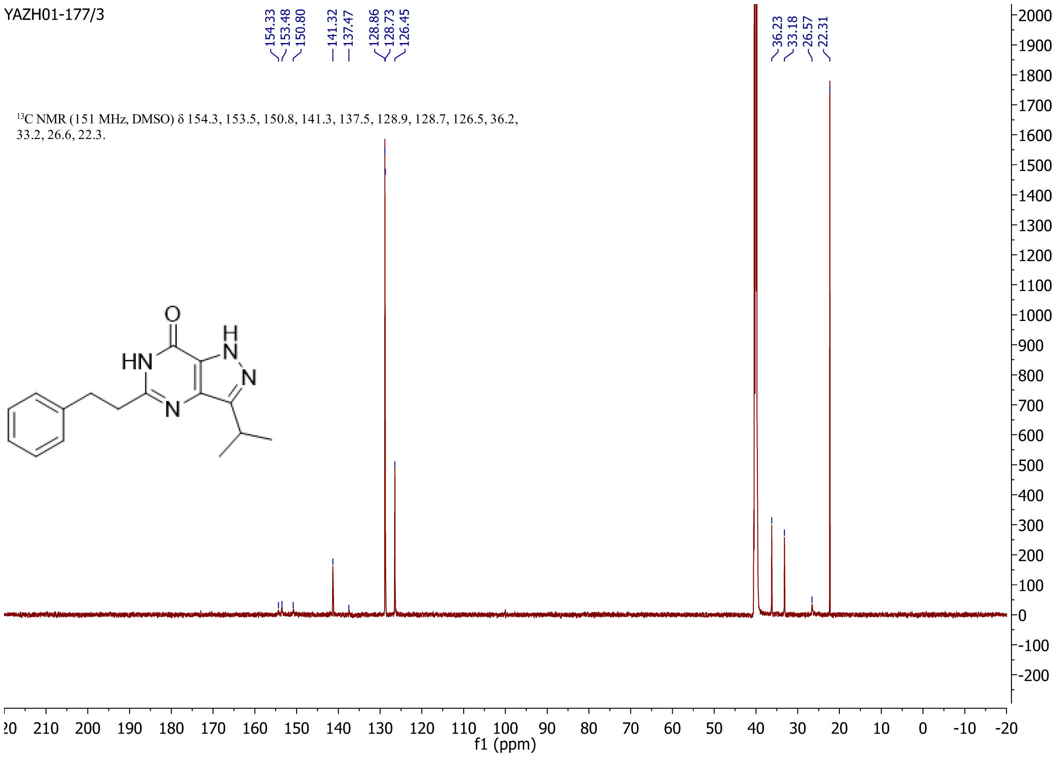

Figure S30. ¹³C NMR spectrum of compound **12**.

Acquired by	: Admin
Date Acquired	: 4/28/2016 1:32:12 PM
Sample Name	: YAZH-126-3
Sample ID	:
Tray#	: 1
Vial#	: 24
Injection Volume	: 1
Data File	: C:\LabSolutions\Data\2016 - wk17\YAZH-126-3.lcd
Background File	: blanco28042016.lcd
Method File	: Method SCAN ACID standard.lcm
Report Format	: DefaultLCMS.lcr
Tuning File	: C:\LabSolutions\Tuning\Tuning-ESI-pos-neg01072015a.lct
Processed by	: Admin
Modified Date	: 4/28/2016 1:48:32 PM

PDA Graph

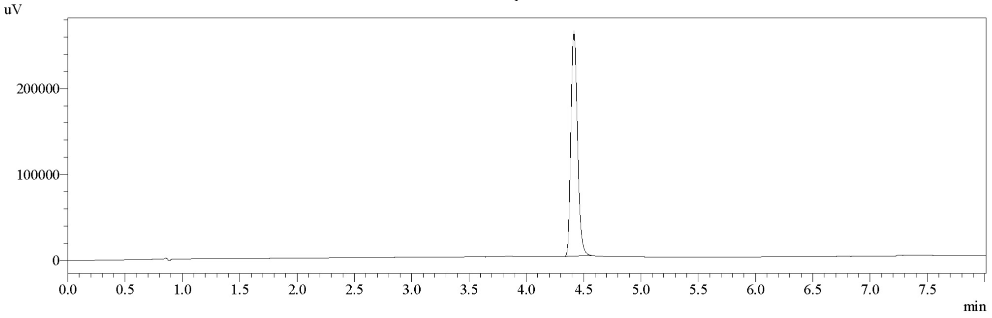

PDA Ch1 254nm 4nm

Peak#	Name	Ret. Time	Area	Area %
1		4.410	1049413	100.000

MS Spectrum Graph

#:1 Ret.Time:Averaged 4.520-4.540(Scan#:453-455)
Mass Peaks:8 Base Peak:283.05(468474) Polarity:Pos Segment1 - Event1

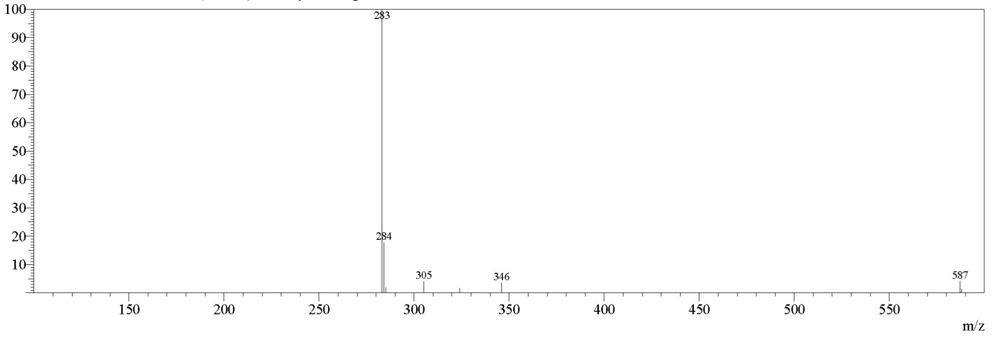

MS Spectrum Table

#:1 Ret.Time:
BG Mode:Calc 4.400<->4.720(441<->473)
Mass Peaks:8 Base Peak:283.05(468474) Polarity:Pos Segment1 - Event1

#	m/z	Abs.Inten.	Rel.Inten.	Charge	Polarity	Monoisotopic	#	m/z	Abs.Inten.	Rel.Inten.	Charge	Polarity	Monoisotopic
1	283.05	468474	100.00				5	324.10	7405	1.58			
2	284.15	83666	17.86				6	346.10	16760	3.58			
3	285.10	8605	1.84				7	587.25	19220	4.10			
4	305.10	19181	4.09				8	588.10	6105	1.30			

Figure S31. LCMS spectrum of compound **13**.

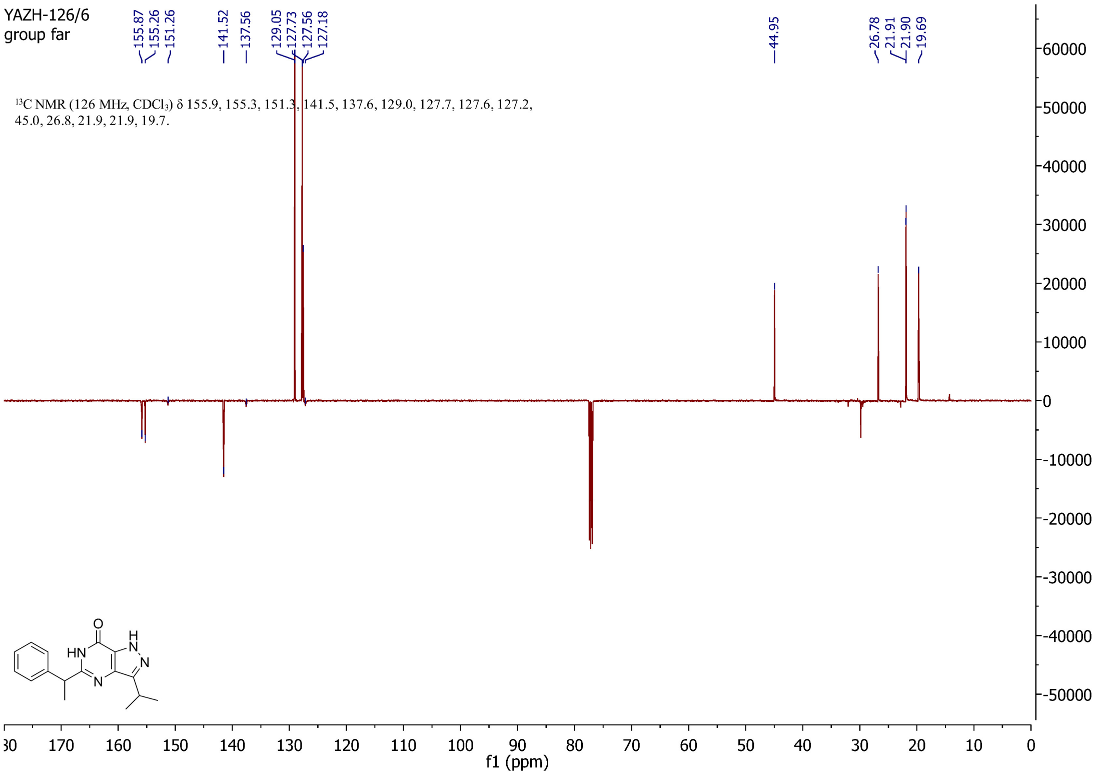

Figure S32. ^{1}H NMR spectrum of compound **13**.

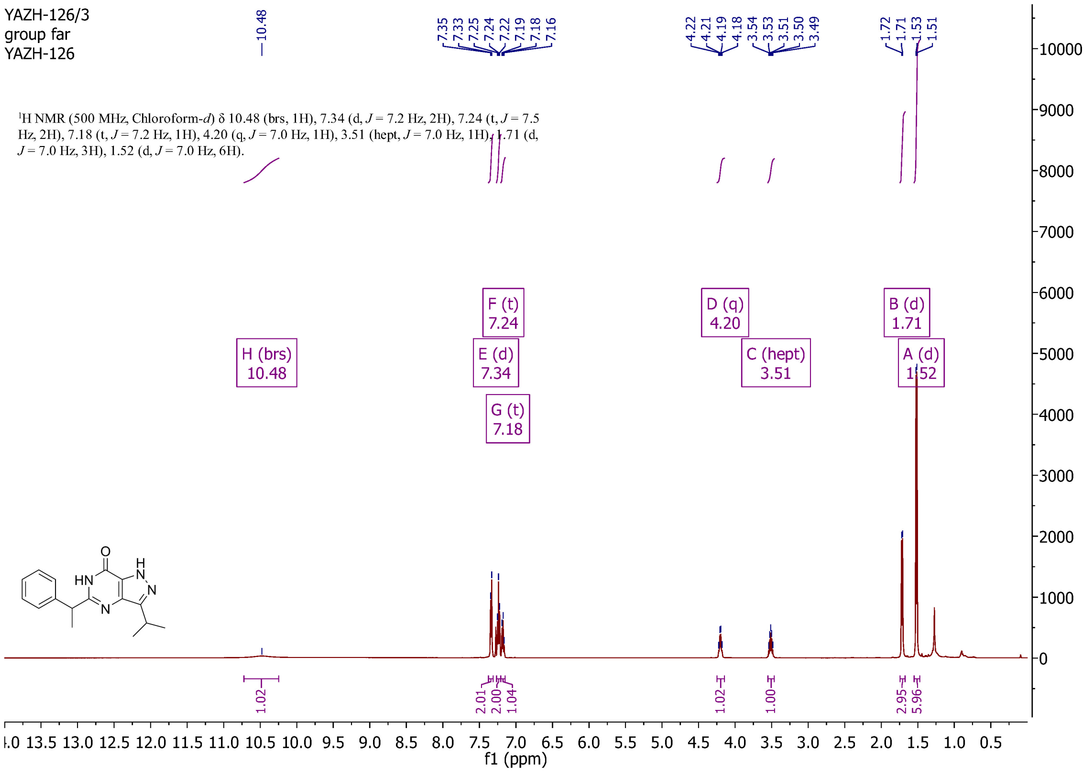

Figure S33. ^{13}C NMR spectrum of compound **13**.

Acquired by	: Admin
Date Acquired	: 16/7/2019 11:46:50 AM
Sample Name	: MOIB_38_final
Sample ID	:
Tray#	: 1
Vial#	: 22
Injection Volume	: 1
Data File	: C:\LabSolutions\Data\2019\2019-wk29\MOIB_38_final.lcd
Background File	: blanco 16072019.lcd
Method File	: Method SCAN ACID standard.lcm
Report Format	: DefaultLCMS.lcr
Tuning File	: C:\LabSolutions\Tuning\Tuning-ESI-pos-neg01072015a.lct
Processed by	: Admin
Modified Date	: 16/7/2019 12:01:13 PM

Chromatogram
MOIB_38_final C:\LabSolutions\Data\2019\2019-wk29\MOIB_38_final.lcd

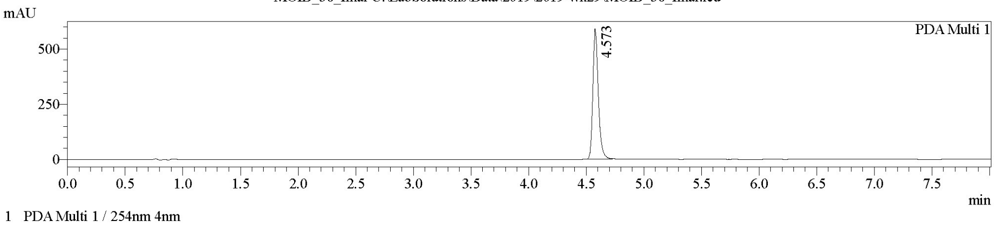

1 PDA Multi 1 / 254nm 4nm

PeakTabl

PDA Ch1 254nm 4nm

Peak#	Name	Ret. Time	Area	Area %
1		4.573	1918219	100.000

MS Chromatogram
MOIB_38_final C:\LabSolutions\Data\2019\2019-wk29\MOIB_38_final.lcd

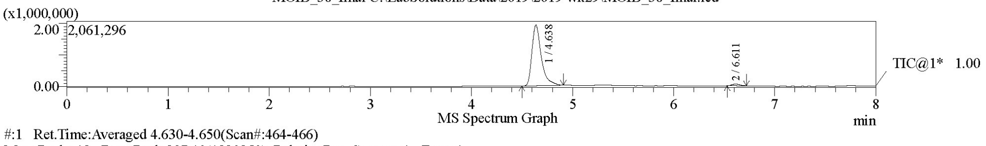

MS Spectrum Graph

#:1 Ret.Time:Averaged 4.630-4.650(Scan#:464-466)
Mass Peaks:12 Base Peak:297.10(1220350) Polarity:Pos Segment1 - Event1

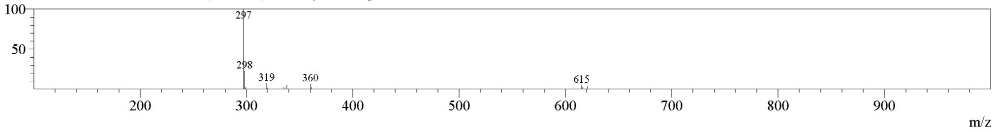

MS Spectrum Table

#:1 Ret.Time:
BG Mode:Calc 4.500<->4.910(451<->492)
Mass Peaks:12 Base Peak:297.10(1220350) Polarity:Pos Segment1 - Event1

#	m/z	Abs.Inten.	Rel.Inten.	Charge	Polarity	Monoisotopic	#	m/z	Abs.Inten.	Rel.Inten.	Charge	Polarity	Monoisotopic
1	297.10	1220350	100.00				7	338.10	63806	5.23			
2	298.10	273031	22.37				8	360.10	81633	6.69			
3	299.10	26922	2.21				9	361.15	22098	1.81			
4	319.10	85305	6.99				10	615.30	59689	4.89			
5	320.10	18541	1.52				11	616.30	24305	1.99			
6	335.05	22825	1.87				12	620.50	44655	3.66			

Figure S34. LCMS spectrum of compound **14**.

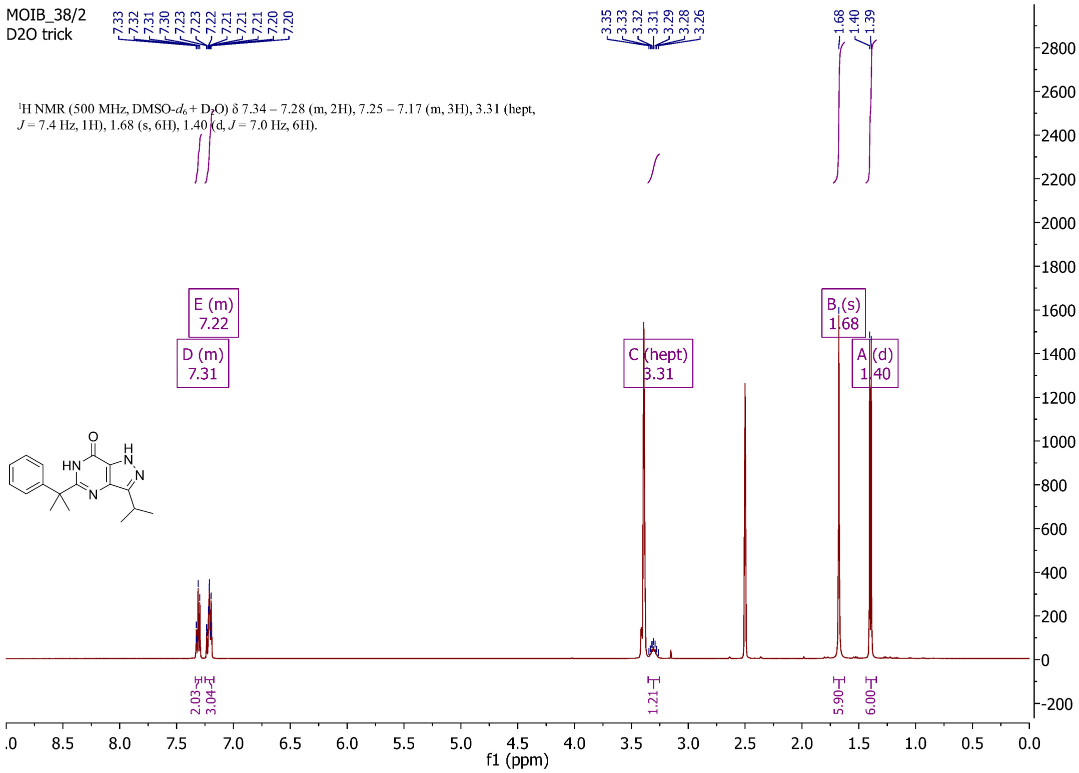

Figure S35. ^{1}H NMR spectrum of compound **14**.

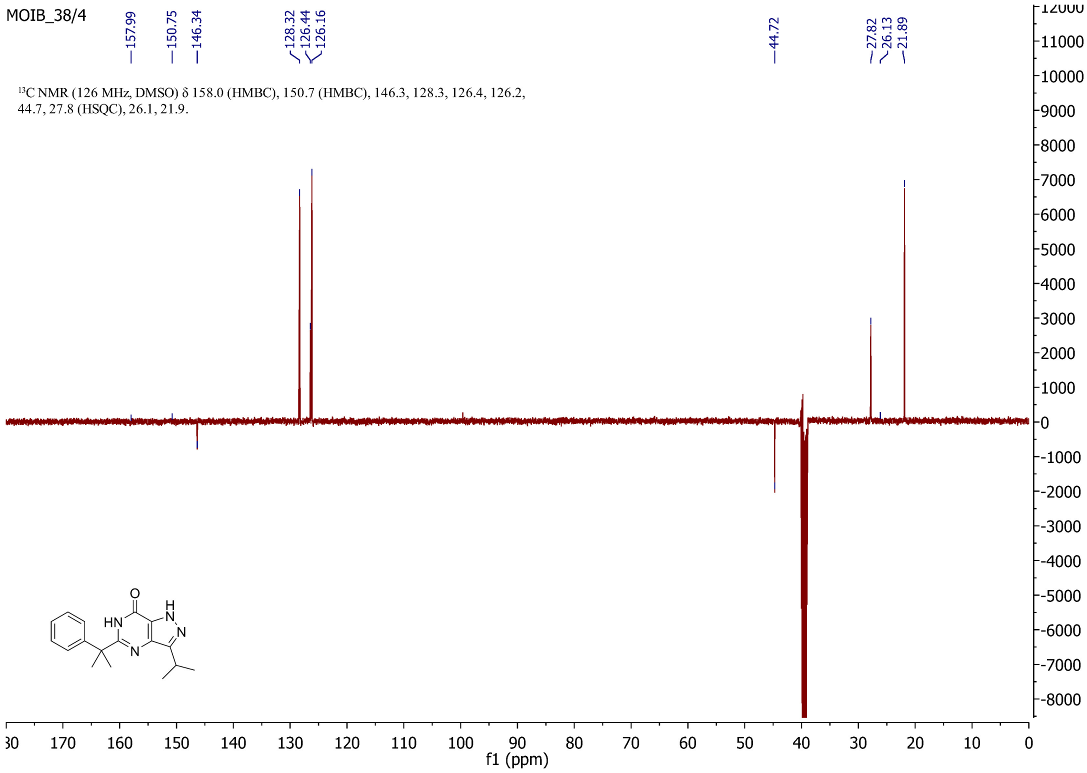

Figure S36. ^{13}C NMR spectrum of compound **14**.

Acquired by	: Admin
Date Acquired	: 16/7/2019 11:55:25 AM
Sample Name	: MOIB_39_final
Sample ID	:
Tray#	: 1
Vial#	: 23
Injection Volume	: 1
Data File	: C:\LabSolutions\Data\2019\2019-wk29\MOIB_39_final.lcd
Background File	: blanco 16072019.lcd
Method File	: Method SCAN ACID standard.lcm
Report Format	: DefaultLCMS.lcr
Tuning File	: C:\LabSolutions\Tuning\Tuning-ESI-pos-neg01072015a.lct
Processed by	: Admin
Modified Date	: 16/7/2019 12:04:00 PM

Chromatogram
MOIB_39_final C:\LabSolutions\Data\2019\2019-wk29\MOIB_39_final.lcd

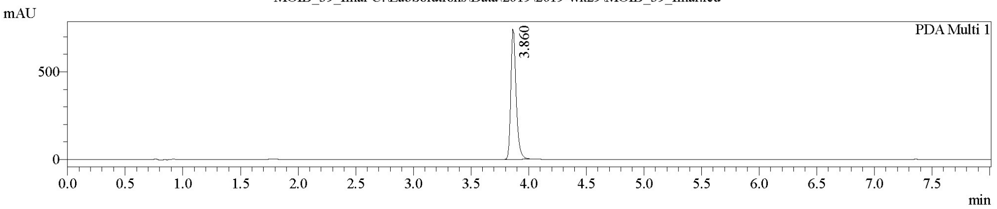

1 PDA Multi 1 / 254nm 4nm

PeakTabl

PDA Ch1 254nm 4nm

Peak#	Name	Ret. Time	Area	Area %
1		3.860	2365954	100.000

MS Chromatogram
MOIB_39_final C:\LabSolutions\Data\2019\2019-wk29\MOIB_39_final.lcd

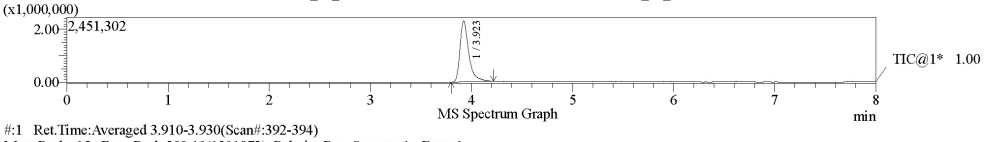

MS Spectrum Graph

#:1 Ret.Time:Averaged 3.910-3.930(Scan#:392-394)
Mass Peaks:15 Base Peak:299.10(1201872) Polarity:Pos Segment1 - Event1

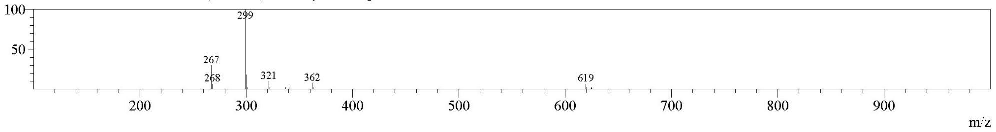

MS Spectrum Table

#:1 Ret.Time:
BG Mode:Calc 3.800<->4.220(381<->423)
Mass Peaks:15 Base Peak:299.10(1201872) Polarity:Pos Segment1 - Event1

#	m/z	Abs.Inten.	Rel.Inten.	Charge	Polarity	Monoisotopic
1	267.05	356809	29.69			
2	268.05	77318	6.43			
3	299.10	1201872	100.00			
4	300.05	219603	18.27			
5	301.05	23930	1.99			
6	321.05	118849	9.89			
7	322.10	25171	2.09			
8	337.00	27554	2.29			

#	m/z	Abs.Inten.	Rel.Inten.	Charge	Polarity	Monoisotopic
9	340.05	31975	2.66			
10	362.10	89695	7.46			
11	363.15	21610	1.80			
12	619.30	78602	6.54			
13	620.30	32324	2.69			
14	624.30	29045	2.42			
15	625.05	17260	1.44			

Figure S37. LCMS spectrum of compound **15**.

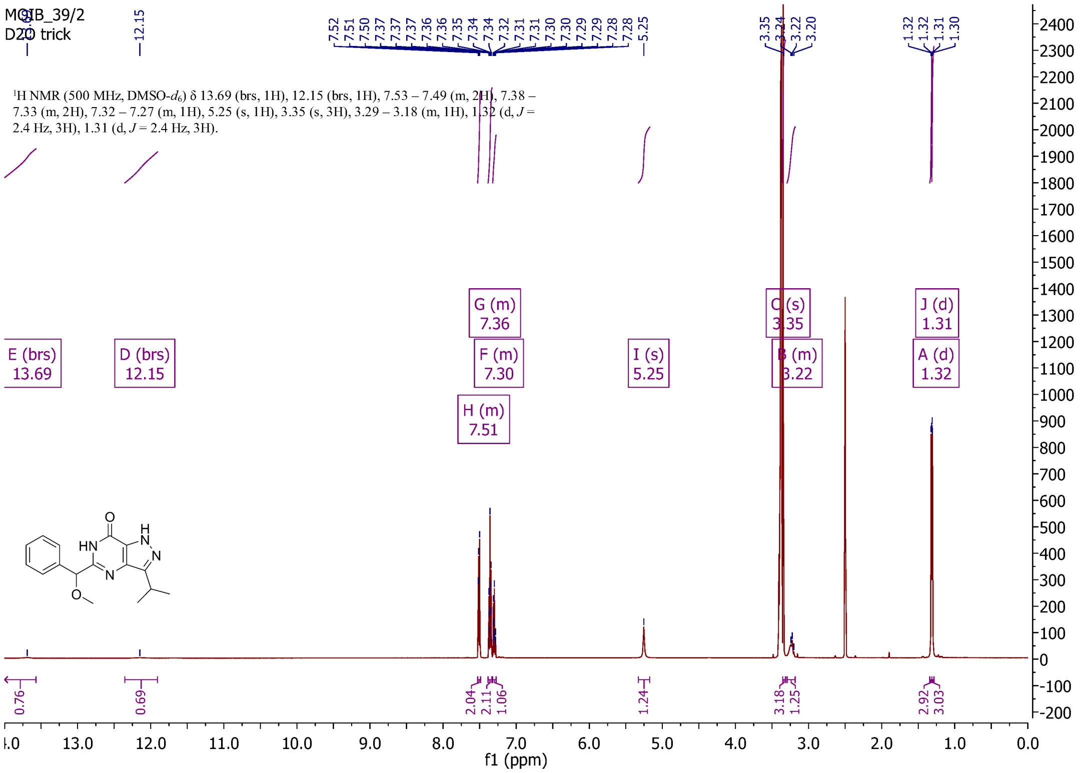

Figure S38. ^{1}H NMR spectrum of compound **15**.

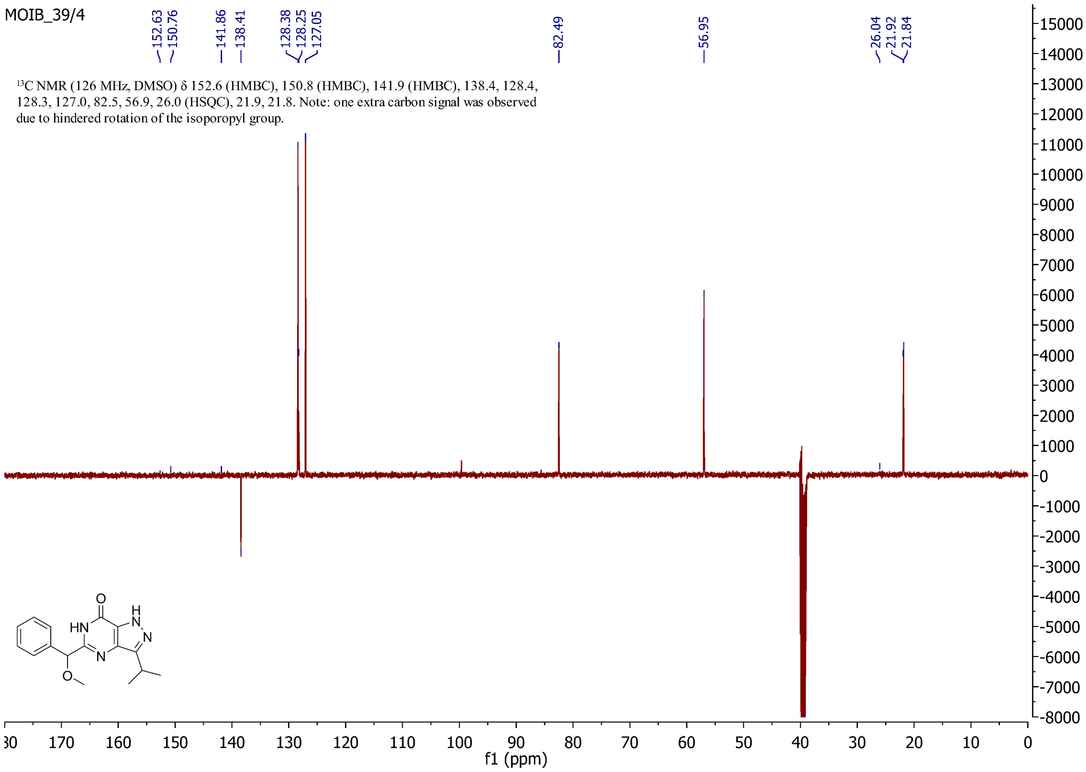

Figure S39. ^{13}C NMR spectrum of compound **15**.

Acquired by	: Admin
Date Acquired	: 18/7/2019 11:50:40 AM
Sample Name	: MOIB_41_final
Sample ID	:
Tray#	: 1
Vial#	: 23
Injection Volume	: 1
Data File	: C:\LabSolutions\Data\2019\2019-wk29\MOIB_41_final.lcd
Background File	: blanco 18072019.lcd
Method File	: Method SCAN ACID standard.lcm
Report Format	: DefaultLCMS.lcr
Tuning File	: C:\LabSolutions\Tuning\Tuning-ESI-pos-neg01072015a.lct
Processed by	: Admin
Modified Date	: 18/7/2019 12:02:44 PM

Chromatogram
MOIB_41_final C:\LabSolutions\Data\2019\2019-wk29\MOIB_41_final.lcd

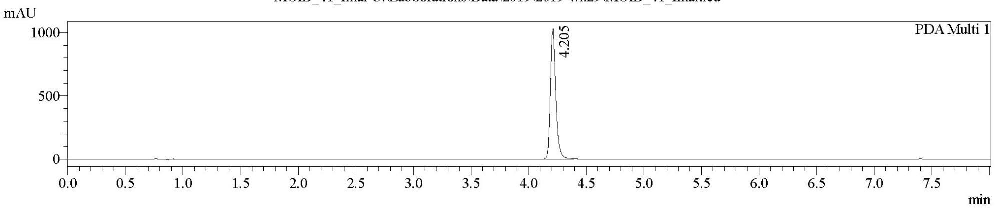

1 PDA Multi 1 / 254nm 4nm

PeakTabl

PDA Ch1 254nm 4nm

Peak#	Name	Ret. Time	Area	Area %
1		4.205	3333249	100.000

MS Chromatogram
MOIB_41_final C:\LabSolutions\Data\2019\2019-wk29\MOIB_41_final.lcd

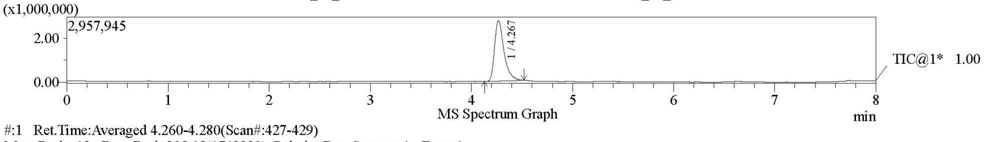

MS Spectrum Graph

#:1 Ret.Time:Averaged 4.260-4.280(Scan#:427-429)
Mass Peaks:13 Base Peak:295.05(1742339) Polarity:Pos Segment1 - Event1

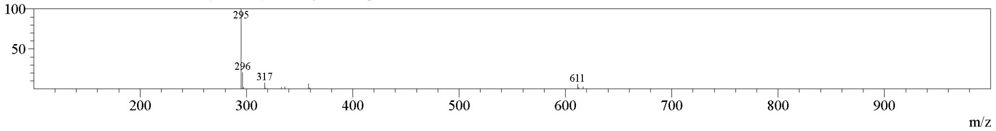

MS Spectrum Table

#:1 Ret.Time:
BG Mode:Calc 4.130<->4.520(414<->453)
Mass Peaks:13 Base Peak:295.05(1742339) Polarity:Pos Segment1 - Event1

#	m/z	Abs.Inten.	Rel.Inten.	Charge	Polarity	Monoisotopic
1	295.05	1742339	100.00			
2	296.05	356374	20.45			
3	297.15	36443	2.09			
4	317.10	131141	7.53			
5	318.10	25143	1.44			
6	333.05	37533	2.15			
7	336.15	44123	2.53			
8	358.10	114949	6.60			
9	359.10	29632	1.70			
10	611.30	101962	5.85			
11	612.30	34432	1.98			
12	616.35	26907	1.54			
13	616.65	51510	2.96			

Figure S40. LCMS spectrum of compound **16**.

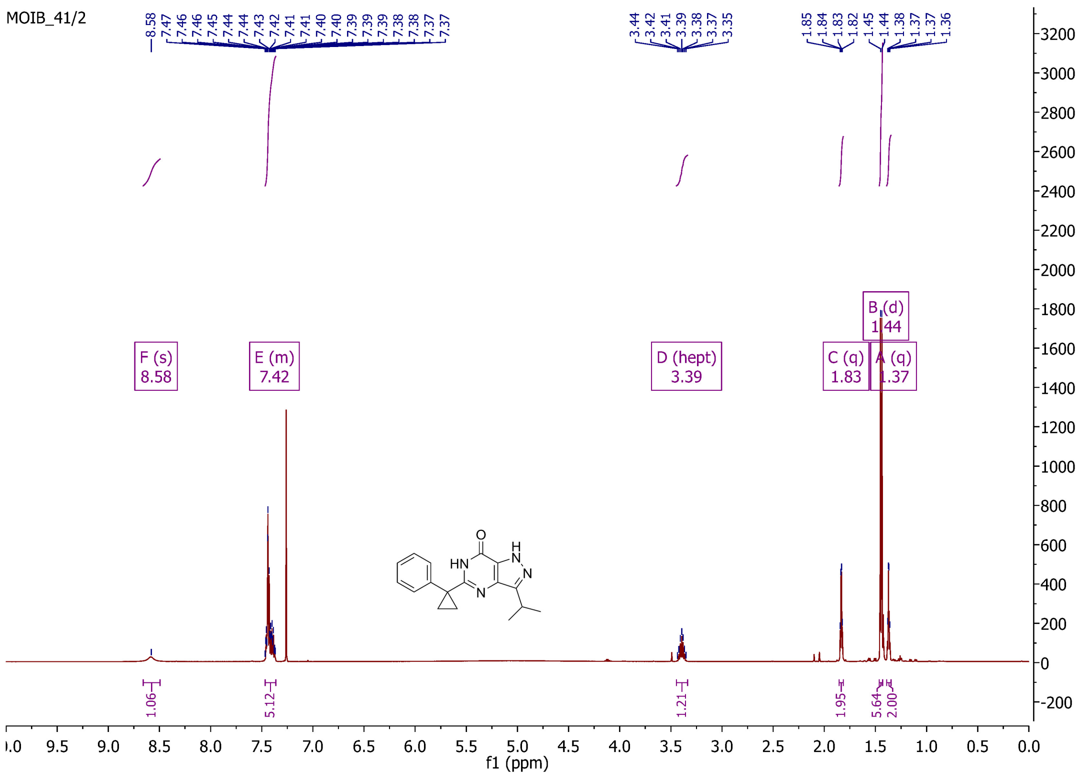

Figure S41. ^{1}H NMR spectrum of compound **16**.

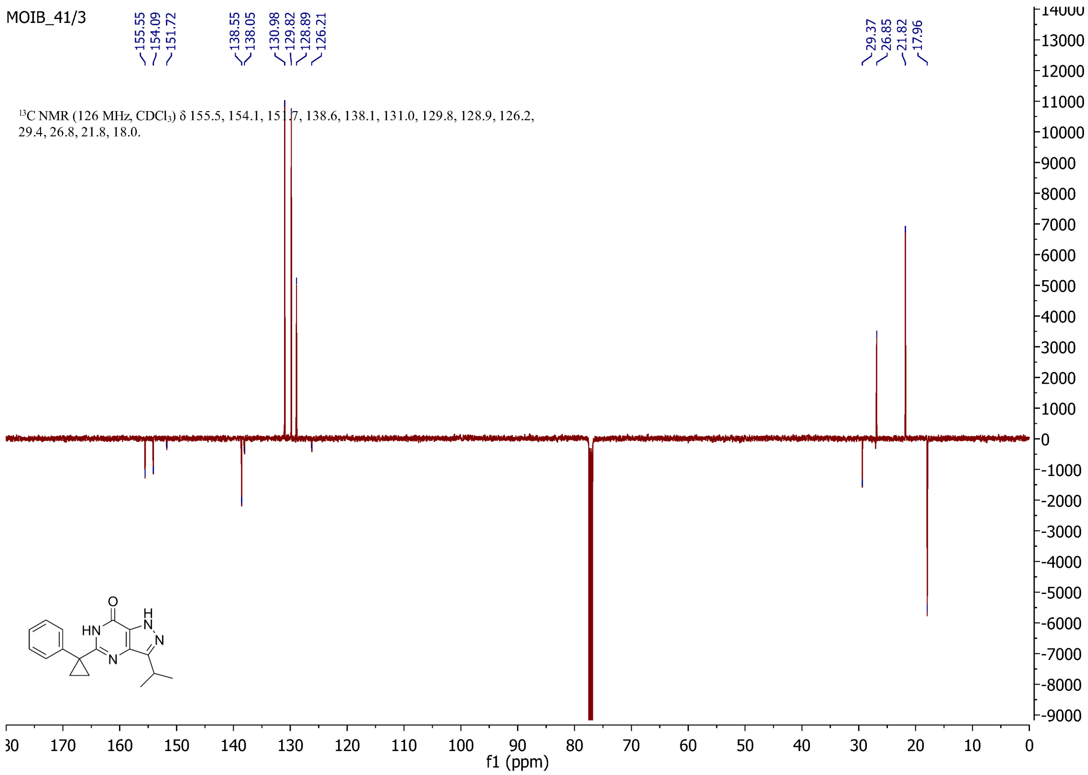

Figure S42. ^{13}C NMR spectrum of compound **16**.

Acquired by : Admin
Date Acquired : 18/7/2019 11:59:17 AM
Sample Name : MOIB_42_final
Sample ID :
Tray# : 1
Vial# : 24
Injection Volume : 1
Data File : C:\LabSolutions\Data\2019\2019-wk29\MOIB_42_final.lcd
Background File : blanco 18072019.lcd
Method File : Method SCAN ACID standard.lcm
Report Format : DefaultLCMS.lcr
Tuning File : C:\LabSolutions\Tuning\Tuning-ESI-pos-neg01072015a.lct
Processed by : Admin
Modified Date : 18/7/2019 12:24:07 PM

Chromatogram
MOIB_42_final C:\LabSolutions\Data\2019\2019-wk29\MOIB_42_final.lcd

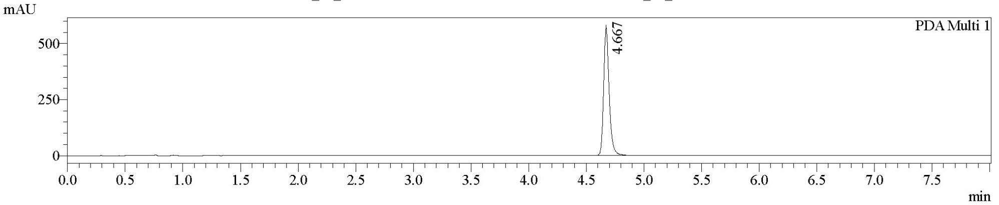

1 PDA Multi 1 / 254nm 4nm

PeakTabl

PDA Ch1 254nm 4nm

Peak#	Name	Ret. Time	Area	Area %
1		4.667	1918948	100.000

MS Chromatogram
MOIB_42_final C:\LabSolutions\Data\2019\2019-wk29\MOIB_42_final.lcd

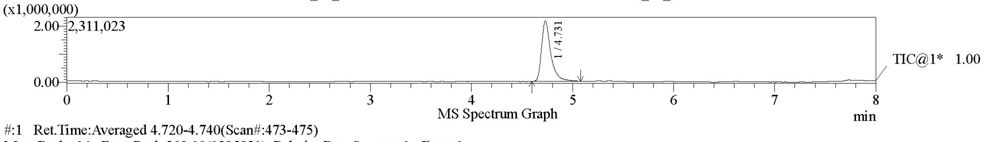

MS Spectrum Graph

#:1 Ret.Time:Averaged 4.720-4.740(Scan#:473-475)
Mass Peaks:14 Base Peak:309.10(1295831) Polarity:Pos Segment1 - Event1

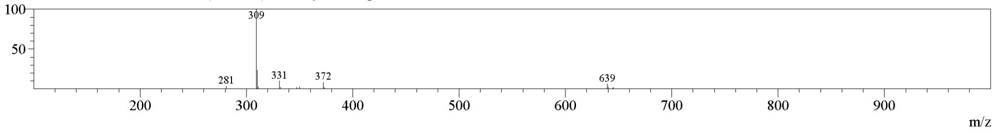

MS Spectrum Table

#:1 Ret.Time:
BG Mode:Calc 4.600<->5.080(461<->509)
Mass Peaks:14 Base Peak:309.10(1295831) Polarity:Pos Segment1 - Event1

#	m/z	Abs.Inten.	Rel.Inten.	Charge	Polarity	Monoisotopic
1	281.05	41089	3.17			
2	309.10	1295831	100.00			
3	310.10	305657	23.59			
4	311.05	32641	2.52			
5	331.10	128965	9.95			
6	332.10	25155	1.94			
7	347.10	30358	2.34			
8	350.10	38187	2.95			
9	372.10	110924	8.56			
10	373.10	26340	2.03			
11	639.30	79877	6.16			
12	640.30	34150	2.64			
13	644.55	14402	1.11			
14	644.60	30521	2.36			

Figure S43. LCMS spectrum of compound **17**.

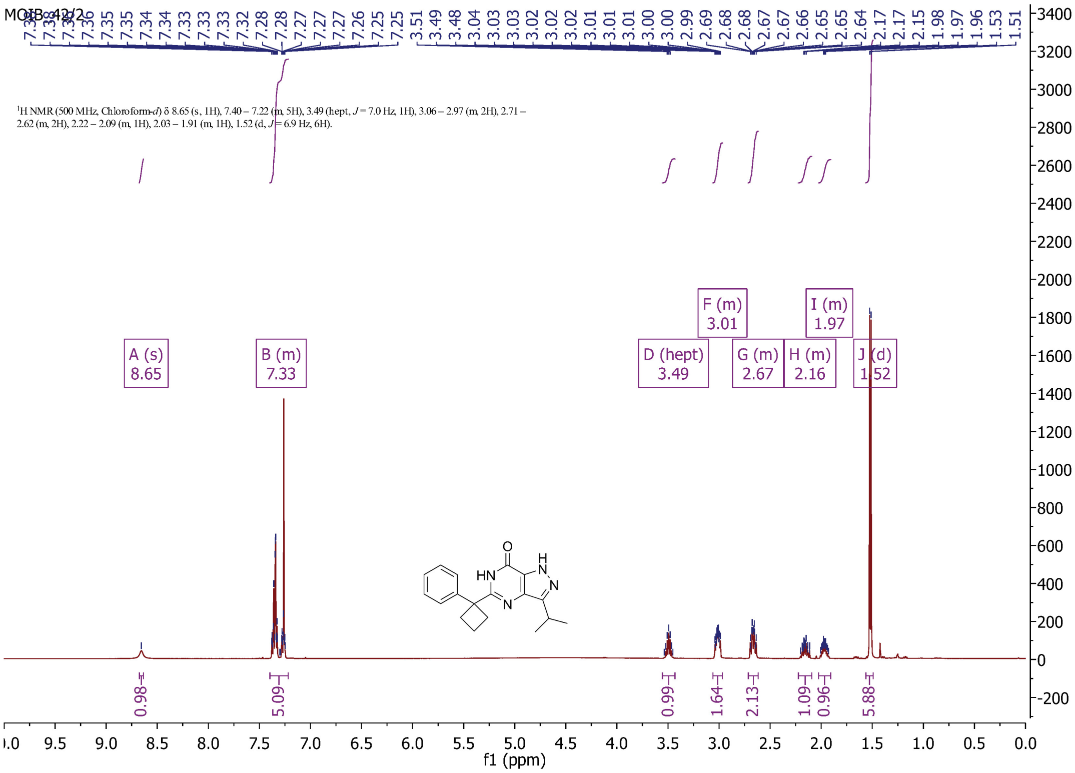

Figure S44. ^{1}H NMR spectrum of compound **17**.

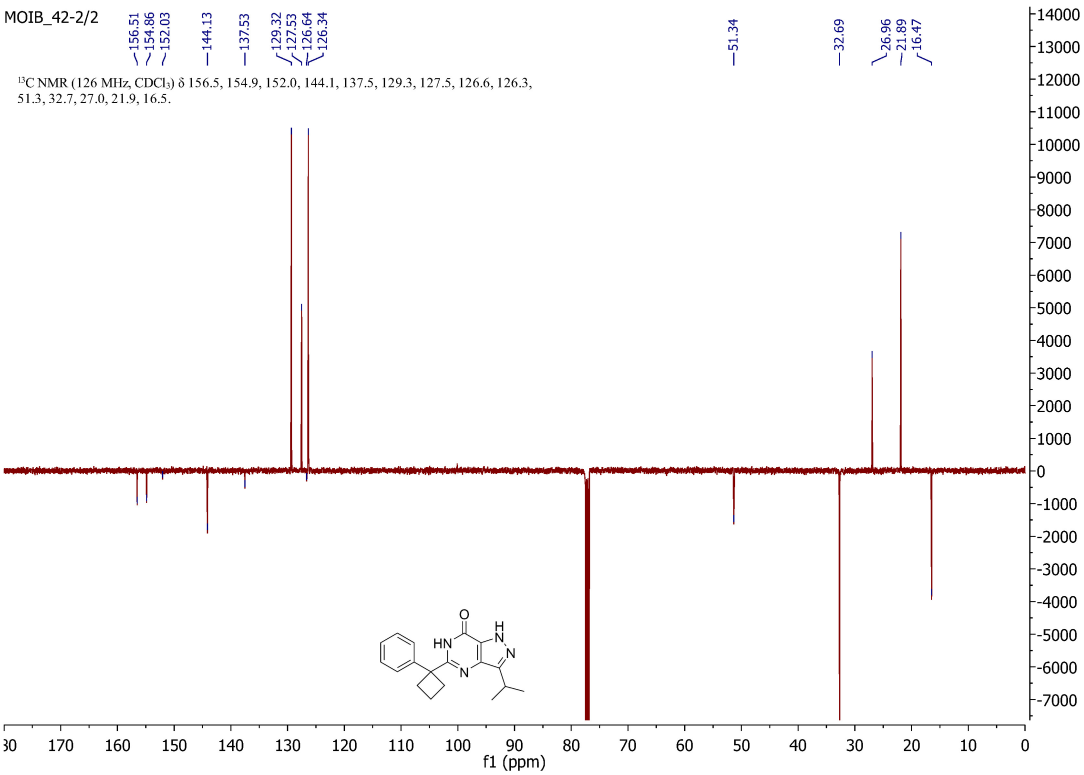

Figure S45. ^{13}C NMR spectrum of compound **17**.

Acquired by : Admin
Date Acquired : 23/4/2018 2:58:02 PM
Sample Name : YAZH01-202-3
Sample ID :
Tray# : 1
Vial# : 18
Injection Volume : 4
Data File : C:\LabSolutions\Data\2018\2018-wk17\YAZH01-202-3.lcd
Background File : blanco 23042018.lcd
Method File : Method SCAN ACID standard.lcm
Report Format : DefaultLCMS.lcr
Tuning File : C:\LabSolutions\Tuning\Tuning-ESI-pos-neg01072015a.lct
Processed by : Admin
Modified Date : 23/4/2018 4:34:04 PM

Chromatogram
YAZH01-202-3 C:\LabSolutions\Data\2018\2018-wk17\YAZH01-202-3.lcd

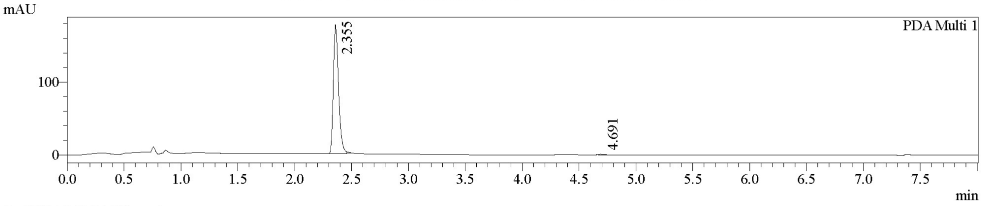

1 PDA Multi 1 / 254nm 4nm

PeakTabl

PDA Ch1 254nm 4nm

Peak#	Name	Ret. Time	Area	Area %
1		2.355	553886	99.546
2		4.691	2527	0.454

MS Chromatogram
YAZH01-202-3 C:\LabSolutions\Data\2018\2018-wk17\YAZH01-202-3.lcd

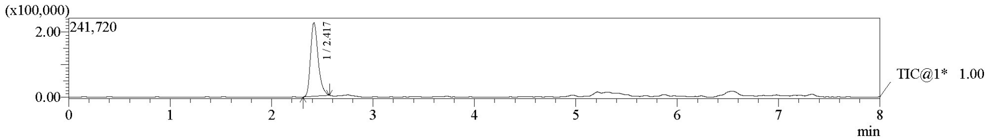

MS Spectrum Graph
#:1 Ret.Time:Averaged 2.410-2.430(Scan#:242-244)
Mass Peaks:15 Base Peak:179.00(133247) Polarity:Pos Segment1 - Event1

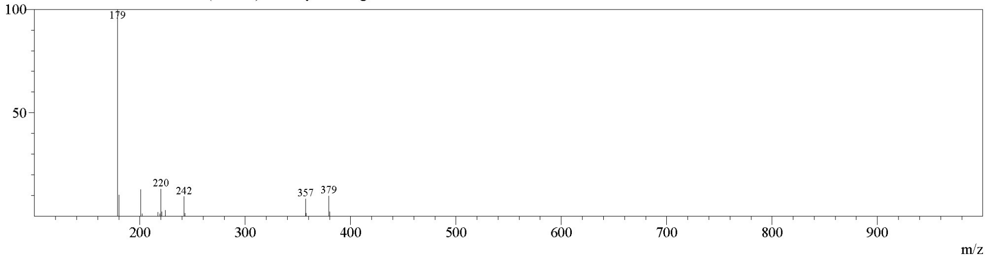

MS Spectrum Table
#:1 Ret.Time:
BG Mode:Calc 2.310<->2.570(232<->258)
Mass Peaks:15 Base Peak:179.00(133247) Polarity:Pos Segment1 - Event1

#	m/z	Abs.Inten.	Rel.Inten.	Charge	Polarity	Monoisotopic
1	179.00	133247	100.00			
2	180.05	13781	10.34			
3	200.95	17230	12.93			
4	202.00	1697	1.27			
5	217.05	2578	1.93			
6	219.00	1491	1.12			
7	220.10	17527	13.15			
8	221.00	2936	2.20			
9	224.10	3951	2.97			
10	242.00	12639	9.49			
11	243.10	1868	1.40			
12	357.20	11149	8.37			
13	357.85	2088	1.57			
14	379.10	13162	9.88			
15	380.15	2832	2.13			

Figure S46. LCMS spectrum of compound **18**.

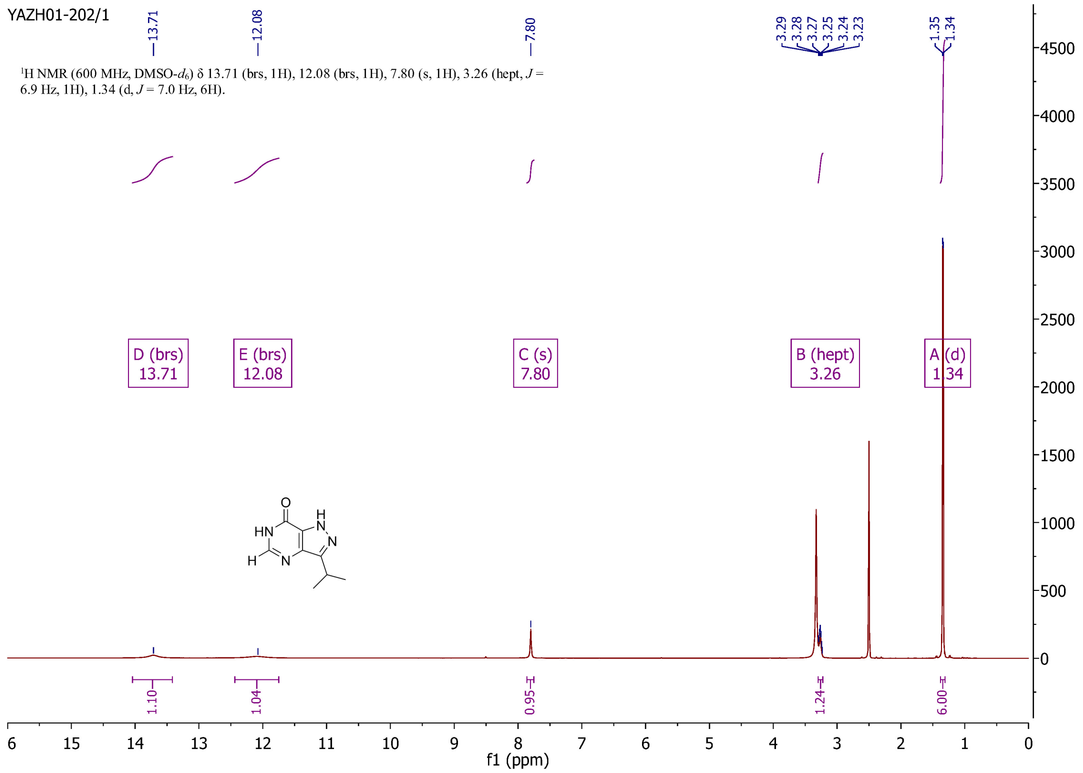

Figure S47. ^{1}H NMR spectrum of compound **18**.

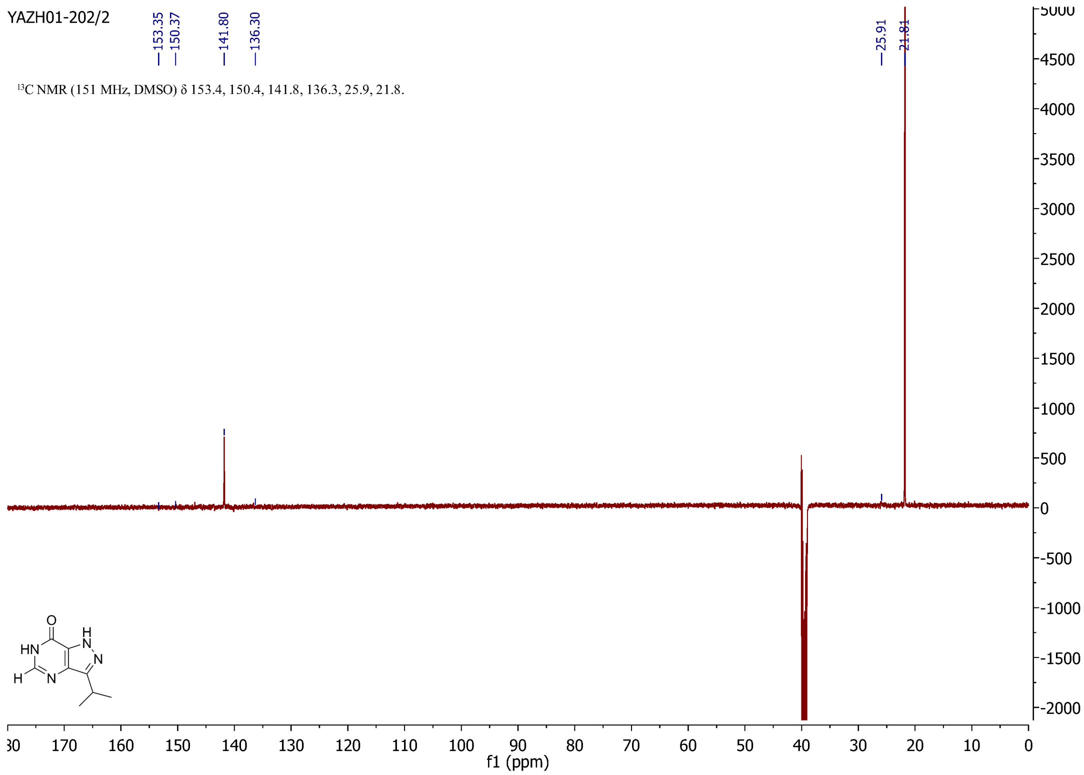

Figure S48. ^{13}C NMR spectrum of compound **18**.

Acquired by	: Admin
Date Acquired	: 19/4/2018 1:51:00 PM
Sample Name	: YAZH01-204
Sample ID	:
Tray#	: 1
Vial#	: 19
Injection Volume	: 4
Data File	: C:\LabSolutions\Data\2018\2018-wk16\YAZH01-204.lcd
Background File	: blanco 19042018.lcd
Method File	: Method SCAN ACID standard.lcm
Report Format	: DefaultLCMS.lcr
Tuning File	: C:\LabSolutions\Tuning\Tuning-ESI-pos-neg01072015a.lct
Processed by	: Admin
Modified Date	: 19/4/2018 3:41:19 PM

Chromatogram
YAZH01-204 C:\LabSolutions\Data\2018\2018-wk16\YAZH01-204.lcd

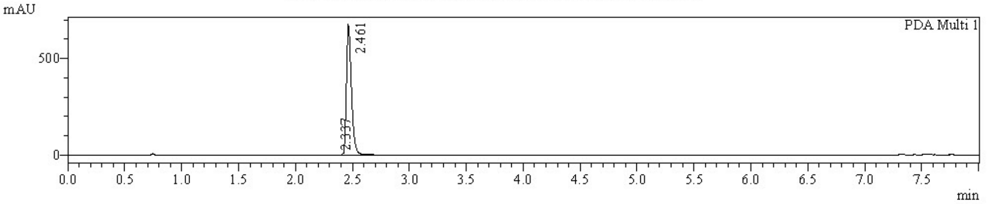

1 PDA Multi 1 / 254nm 4nm

PeakTable

PDA Ch1 254nm 4nm

Peak#	Name	Ret. Time	Area	Area %
1		2.337	446	0.022
2		2.461	2042814	99.978

MS Chromatogram
YAZH01-204 C:\LabSolutions\Data\2018\2018-wk16\YAZH01-204.lcd

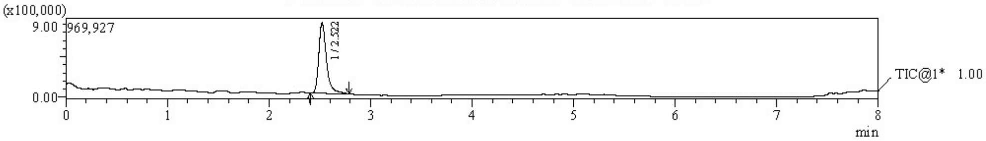

MS Spectrum Graph

#:1 Ret.Time: Averaged 2.510-2.530(Scan#:252-254)
Mass Peaks:9 Base Peak:193.05(675266) Polarity:Pos Segment1 - Event1

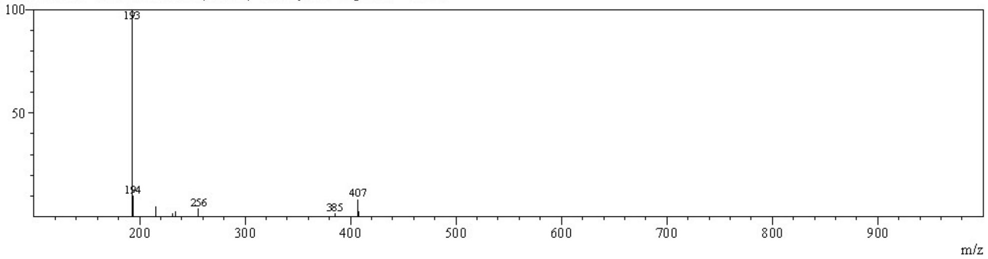

MS Spectrum Table

#:1 Ret.Time:
BG Mode:Calc 2.410<->2.790(242<->280)
Mass Peaks:9 Base Peak:193.05(675266) Polarity:Pos Segment1 - Event1

#	m/z	Abs.Inten.	Rel.Inten.	Charge	Polarity	Monoisotopic
1	193.05	675266	100.00			
2	194.05	65587	9.71			
3	215.00	32079	4.75			
4	231.00	8680	1.29			
5	234.05	16383	2.43			
6	256.05	24769	3.67			
7	385.05	9084	1.35			
8	407.15	55292	8.19			
9	408.15	15853	2.35			

Figure S49. LCMS spectrum of compound **19**.

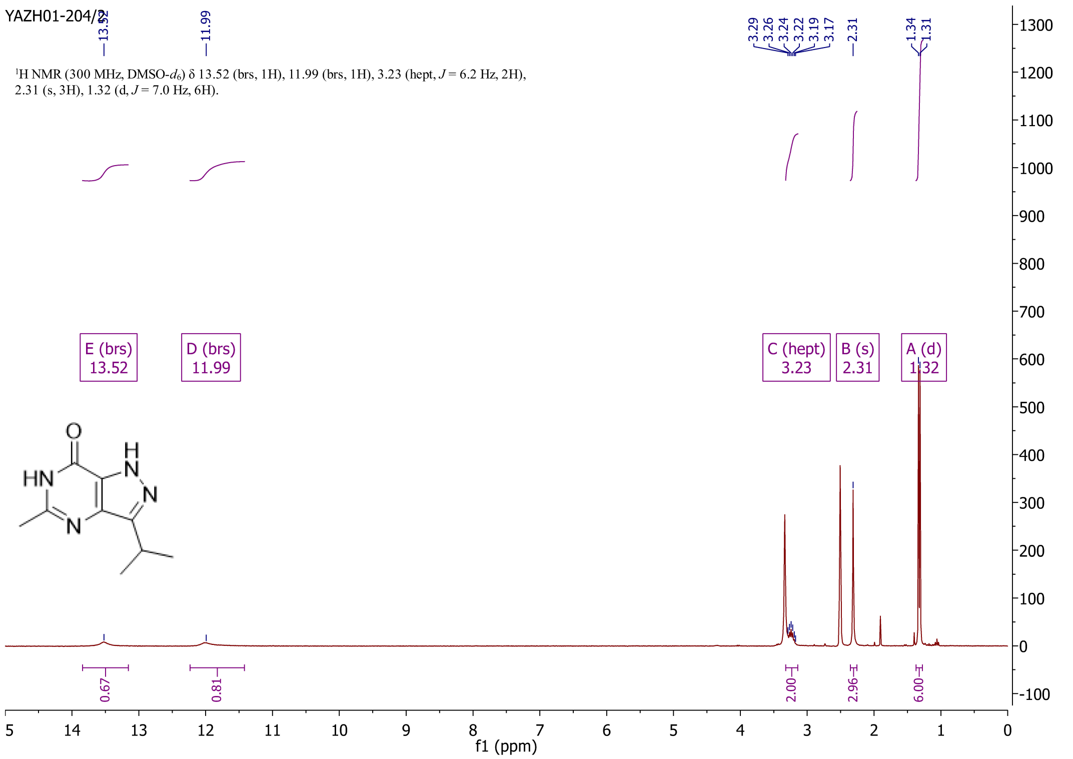

Figure S50. ^{1}H NMR spectrum of compound **19**.

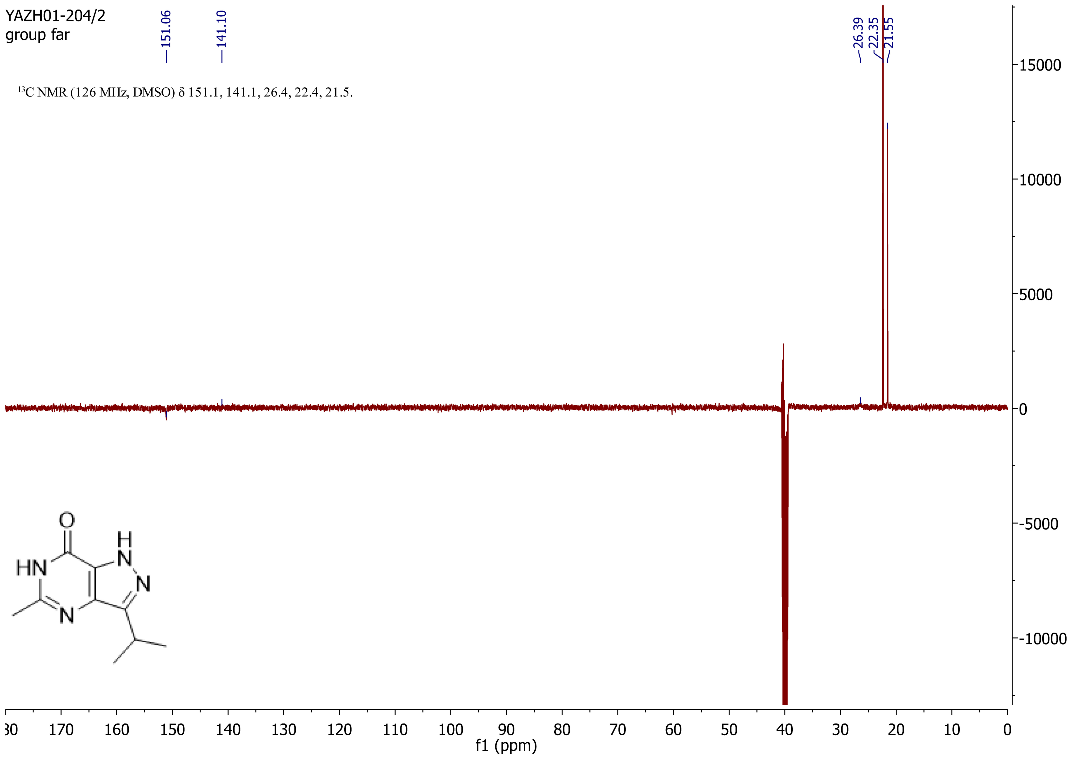

Figure S51. ^{13}C NMR spectrum of compound **19**.

Acquired by	: Admin
Date Acquired	: 19/4/2018 12:59:05 PM
Sample Name	: YAZH01-203-1
Sample ID	:
Tray#	: 1
Vial#	: 28
Injection Volume	: 4
Data File	: C:\LabSolutions\Data\2018\2018-wk16\YAZH01-203-1.lcd
Background File	: blanco 19042018.lcd
Method File	: Method SCAN ACID standard.lcm
Report Format	: DefaultLCMS.lcr
Tuning File	: C:\LabSolutions\Tuning\Tuning-ESI-pos-neg01072015a.lct
Processed by	: Admin
Modified Date	: 19/4/2018 1:10:48 PM

Chromatogram
YAZH01-203-1 C:\LabSolutions\Data\2018\2018-wk16\YAZH01-203-1.lcd

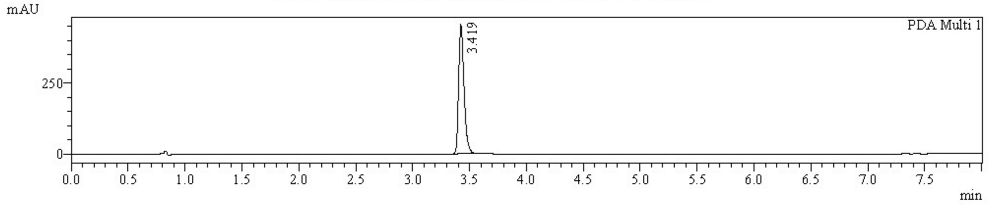

1 PDA Multi 1 / 254nm 4nm

PeakTable

PDA Ch1 254nm 4nm

Peak#	Name	Ret. Time	Area	Area %
1		3.419	1470710	100.000

MS Chromatogram
YAZH01-203-1 C:\LabSolutions\Data\2018\2018-wk16\YAZH01-203-1.lcd

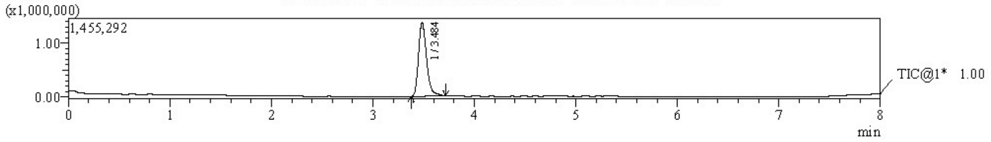

MS Spectrum Graph

#1 Ret.Time: Averaged 3.470-3.490(Scan#:348-350)
Mass Peaks:8 Base Peak:221.10(1031293) Polarity:Pos Segment1 - Event1

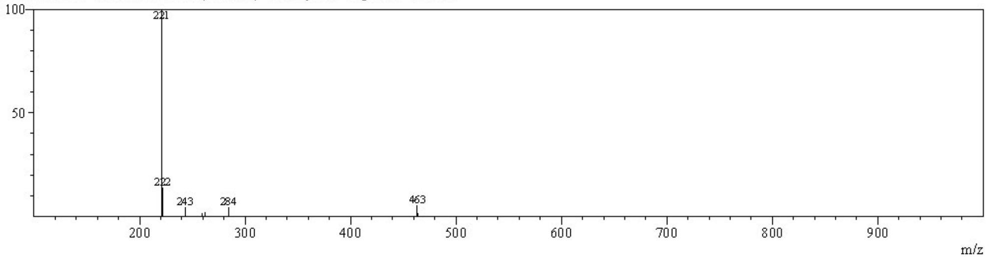

MS Spectrum Table

#:1 Ret.Time:
BG Mode:Calc 3.380<->3.720(339<->373)
Mass Peaks:8 Base Peak:221.10(1031293) Polarity:Pos Segment1 - Event1

#	m/z	Abs.Inten.	Rel.Inten.	Charge	Polarity	Monoisotopic	#	m/z	Abs.Inten.	Rel.Inten.	Charge	Polarity	Monoisotopic
1	221.10	1031293	100.00				5	262.05	20613	2.00			
2	222.05	140359	13.61				6	284.10	42671	4.14			
3	243.00	41841	4.06				7	463.25	50815	4.93			
4	259.10	11590	1.12				8	464.20	15306	1.48			

Figure S52. LCMS spectrum of compound **20**.

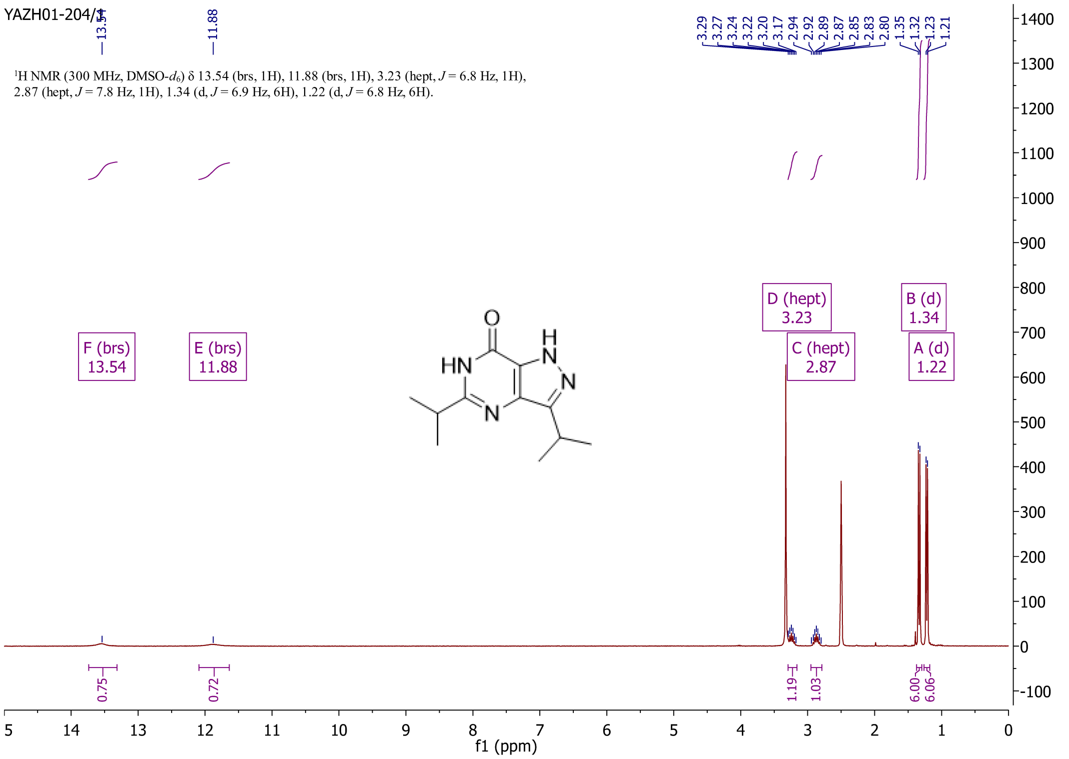

Figure S53. ^{1}H NMR spectrum of compound **20**.

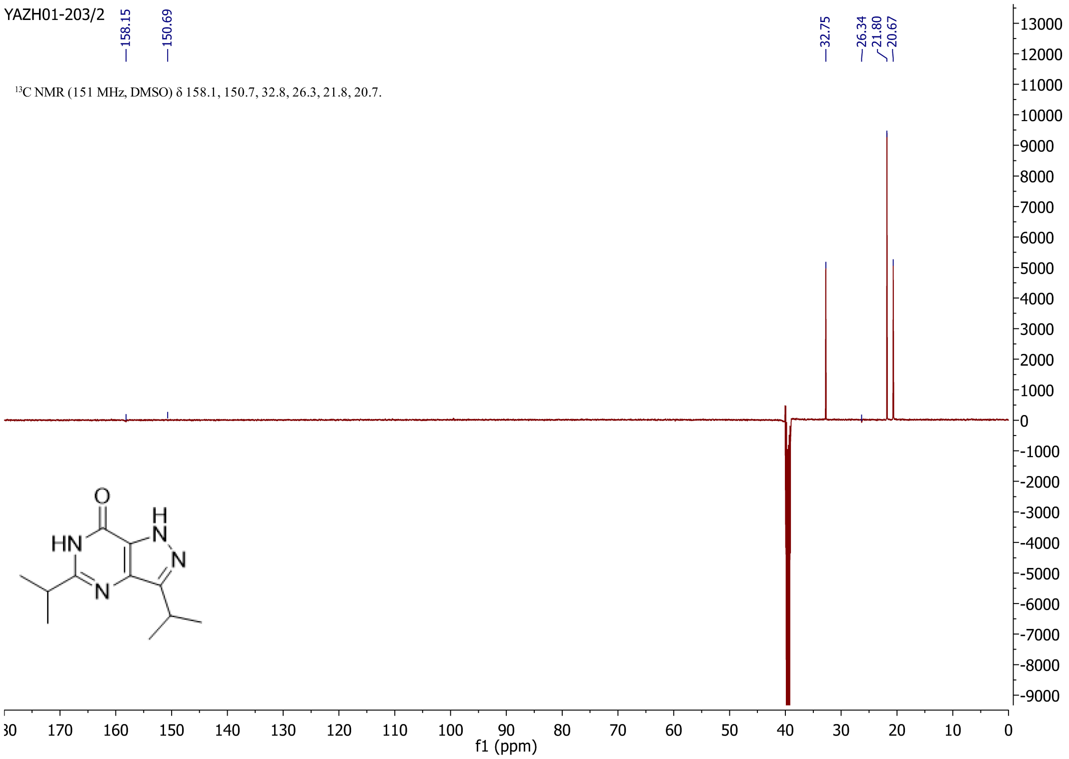

Figure S54. ^{13}C NMR spectrum of compound **20**.

Acquired by : Admin
Date Acquired : 1/4/2019 11:04:28 AM
Sample Name : YAZH_39
Sample ID :
Tray# : 1
Vial# : 8
Injection Volume : 3
Data File : C:\LabSolutions\Data\2019\2019-wk14\YAZH_39.lcd
Background File : blanco 01042019.lcd
Method File : Method SCAN ACID standard.lcm
Report Format : DefaultLCMS.lcr
Tuning File : C:\LabSolutions\Tuning\Tuning-ESI-pos-neg01072015a.lct
Processed by : Admin
Modified Date : 1/4/2019 11:34:36 AM

PDA Graph

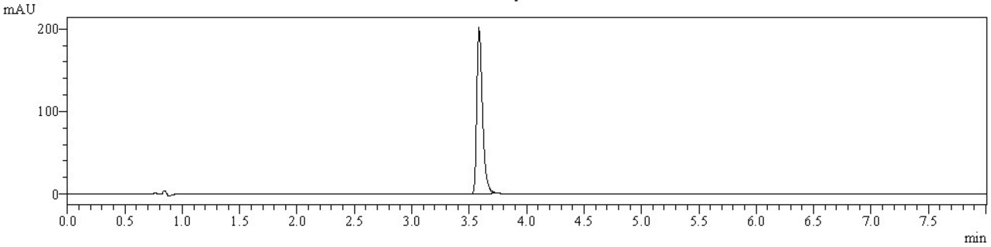

PeakTable

PDA Ch1 254nm 4nm

Peak#	Name	Ret. Time	Area	Area %
1		3.581	688185	100.000

MS Chromatogram
YAZH_39 C:\LabSolutions\Data\2019\2019-wk14\YAZH_39.lcd

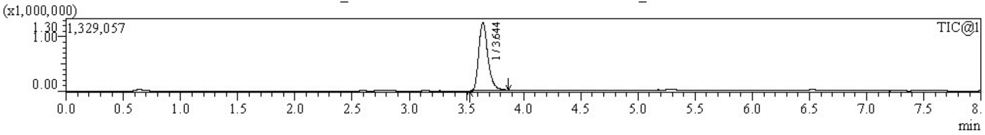

MS Spectrum Graph

#1 Ret.Time:Averaged 3.630-3.650(Scan#:364-366)
BG Mode:Calc 3.530<->3.870(354<->388)
Mass Peaks:6 Base Peak:235.05(956820) Polarity:Pos Segment1 - Event1

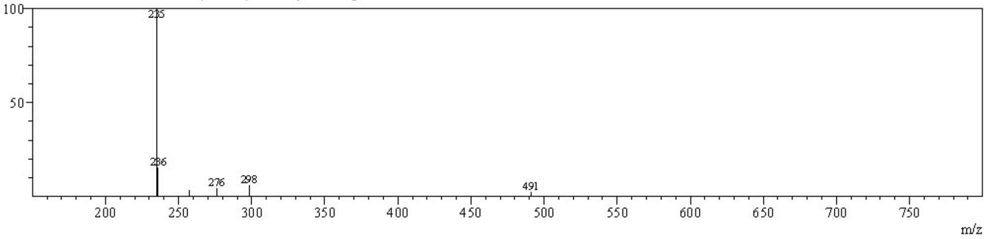

MS Spectrum Table

#:1 Ret.Time:
BG Mode:Calc 3.530<->3.870(354<->388)
Mass Peaks:6 Base Peak:235.05(956820) Polarity:Pos Segment1 - Event1

#	m/z	Abs.Inten.	Rel.Inten.	Charge	Polarity	Monoisotopic	#	m/z	Abs.Inten.	Rel.Inten.	Charge	Polarity	Monoisotopic
1	235.05	956820	100.00				4	276.10	38169	3.99			
2	236.05	144245	15.08				5	298.10	56634	5.92			
3	257.10	31609	3.30				6	491.30	21946	2.29			

Figure S55. LCMS spectrum of compound **21**.

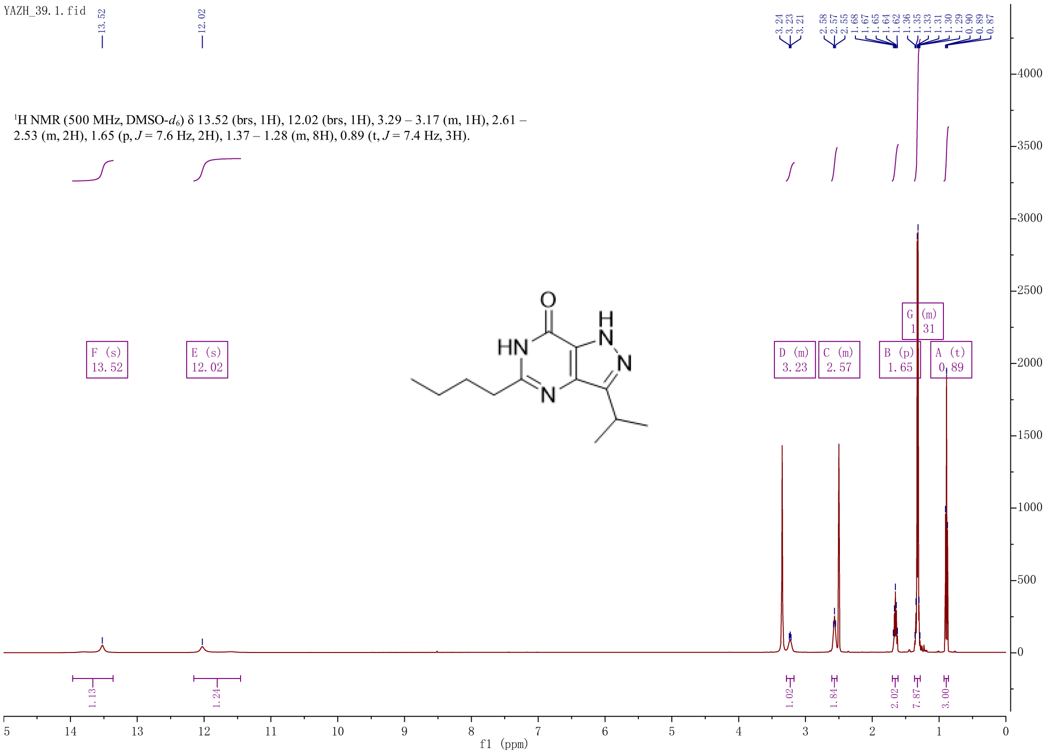

Figure S56. ^{1}H NMR spectrum of compound **21**.

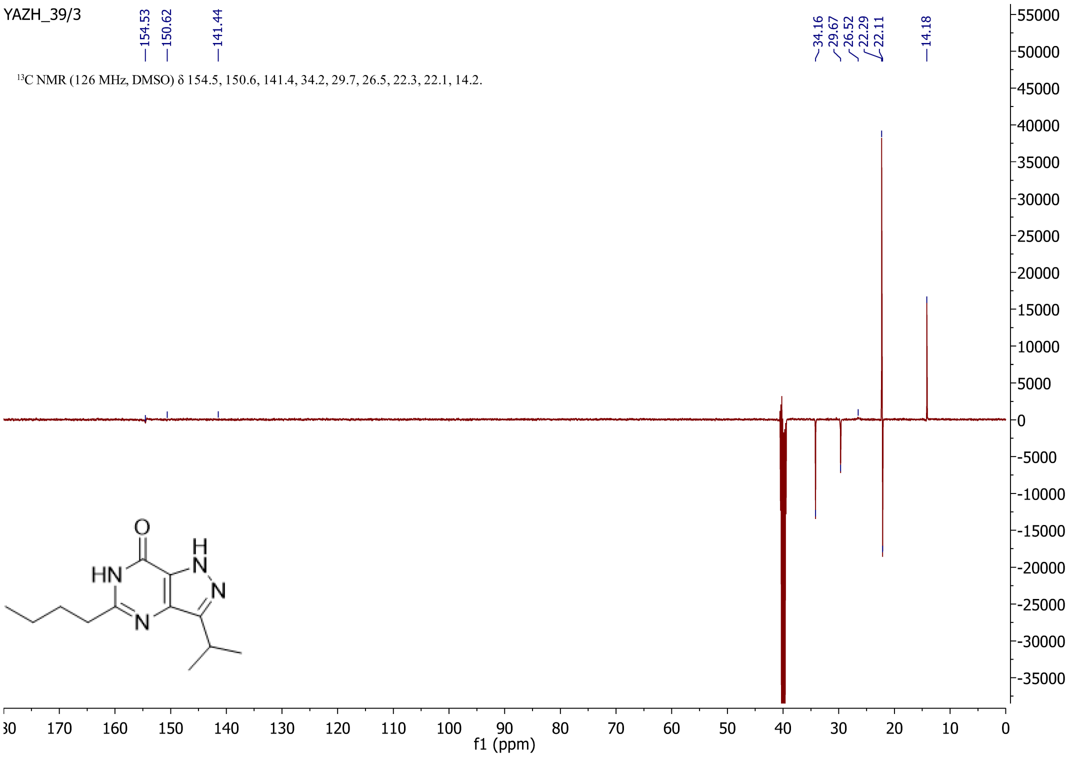

Figure S57. ^{13}C NMR spectrum of compound **21**.

Acquired by	: Admin
Date Acquired	: 29/3/2018 9:28:24 AM
Sample Name	: YAZH01-193-1
Sample ID	:
Tray#	: 1
Vial#	: 8
Injection Volume	: 3
Data File	: C:\LabSolutions\Data\2018\2018-wk13\YAZH01-193-1.lcd
Background File	: blanco 29032018.lcd
Method File	: Method SCAN ACID standard.lcm
Report Format	: DefaultLCMS.lcr
Tuning File	: C:\LabSolutions\Tuning\Tuning-ESI-pos-neg01072015a.lct
Processed by	: Admin
Modified Date	: 29/3/2018 10:22:55 AM

PDA Graph

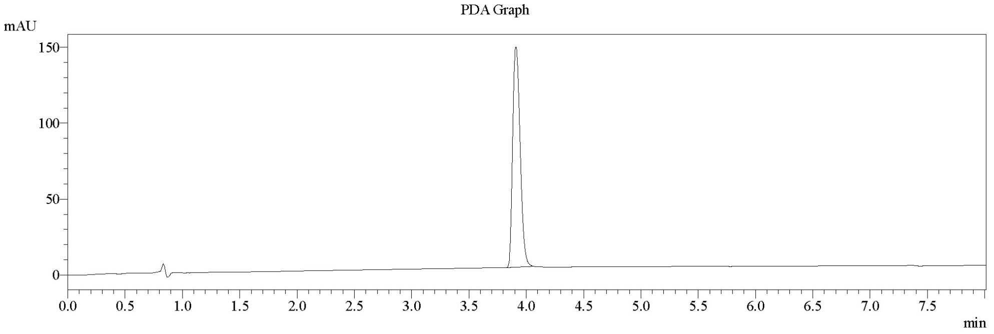

PDA Ch1 254nm 4nm

Peak#	Name	Ret. Time	Area	Area %
1		3.904	654641	100.000

MS Spectrum Graph

#:1 Ret.Time:Averaged 3.950-3.970(Scan#:396-398)
Mass Peaks:8 Base Peak:247.10(655951) Polarity:Pos Segment1 - Event1

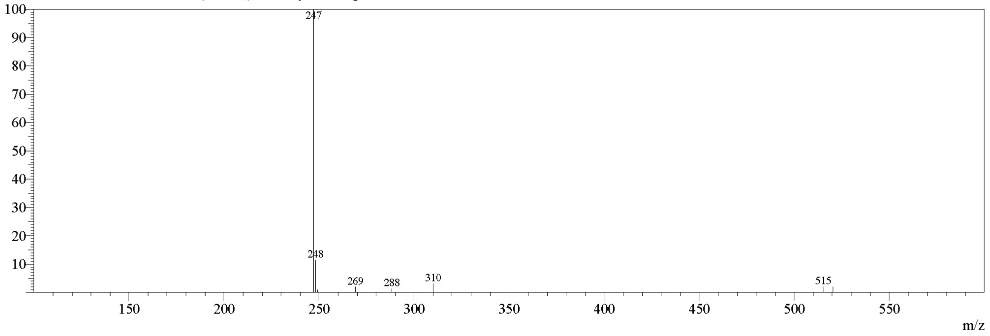

MS Spectrum Table

#:1 Ret.Time:
BG Mode:Calc 3.850<->4.140(386<->415)
Mass Peaks:8 Base Peak:247.10(655951) Polarity:Pos Segment1 - Event1

#	m/z	Abs.Inten.	Rel.Inten.	Charge	Polarity	Monoisotopic	#	m/z	Abs.Inten.	Rel.Inten.	Charge	Polarity	Monoisotopic
1	247.10	655951	100.00				5	288.15	8974	1.37			
2	248.05	74336	11.33				6	310.10	20141	3.07			
3	249.05	6630	1.01				7	515.30	13021	1.99			
4	269.10	12557	1.91				8	520.45	12860	1.96			

Figure S58. LCMS spectrum of compound **22**.

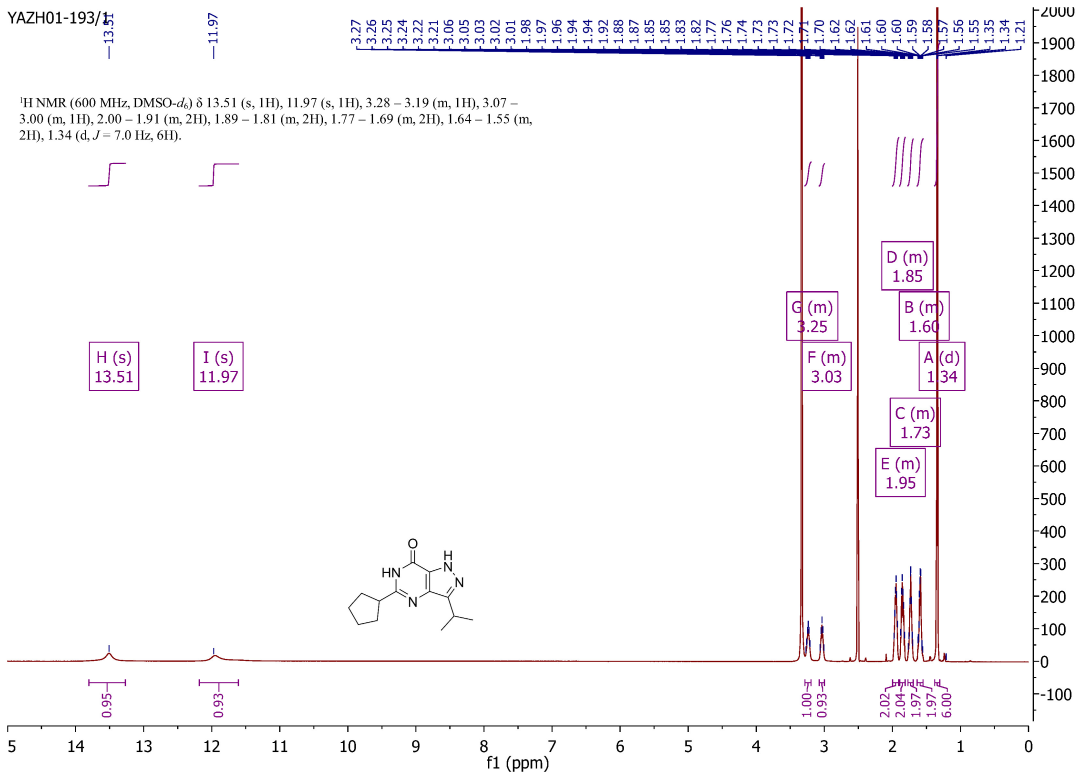

Figure S59. ^{1}H NMR spectrum of compound **22**.

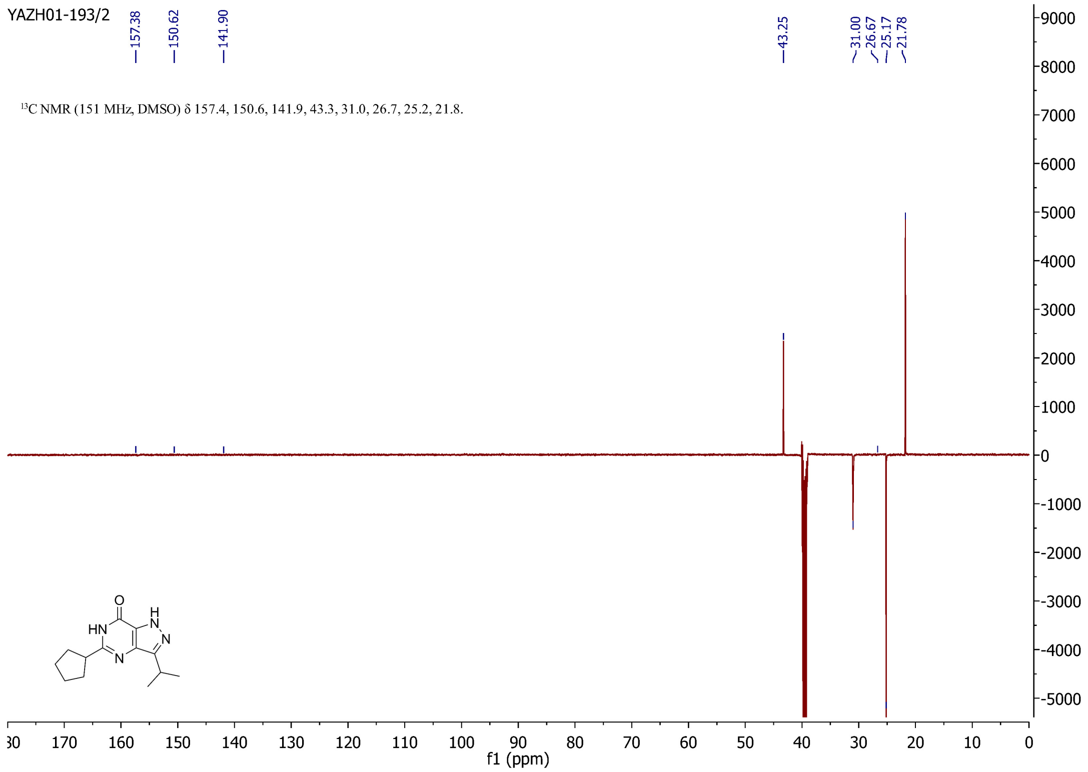

Figure S60. ^{13}C NMR spectrum of compound **22**.

Acquired by	: Admin
Date Acquired	: 7/8/2018 10:15:24 AM
Sample Name	: YAZH01-232
Sample ID	:
Tray#	: 1
Vial#	: 5
Injection Volume	: 3
Data File	: C:\LabSolutions\Data\2018\2018-wk32\YAZH01-232.lcd
Background File	: blanco 070818.lcd
Method File	: Method SCAN ACID standard.lcm
Report Format	: DefaultLCMS.lcr
Tuning File	: C:\LabSolutions\Tuning\Tuning-ESI-pos-neg01072015a.lct
Processed by	: Admin
Modified Date	: 7/8/2018 10:32:58 AM

Chromatogram
YAZH01-232 C:\LabSolutions\Data\2018\2018-wk32\YAZH01-232.lcd

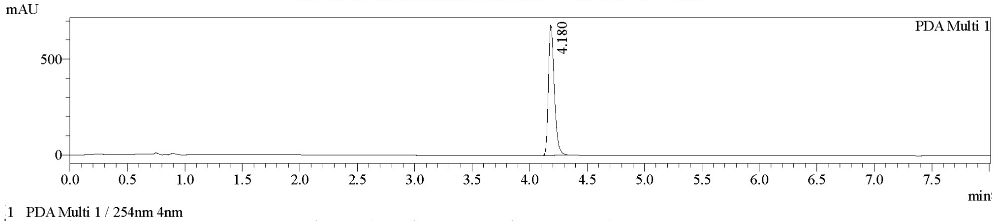

1 PDA Multi 1 / 254nm 4nm

Peak#	Name	Ret. Time	Area	Area %
1		4.180	2401388	100.000

MS Chromatogram
YAZH01-232 C:\LabSolutions\Data\2018\2018-wk32\YAZH01-232.lcd

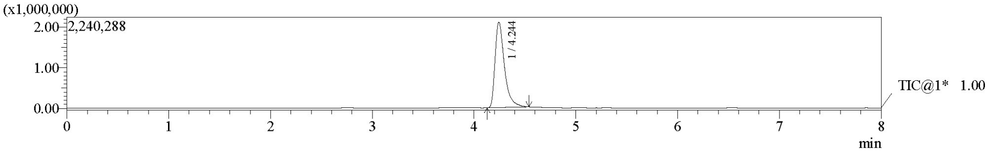

MS Spectrum Graph
#:1 Ret.Time:Averaged 4.230-4.250(Scan#:424-426)
Mass Peaks:8 Base Peak:261.15(1599192) Polarity:Pos Segment1 - Event1

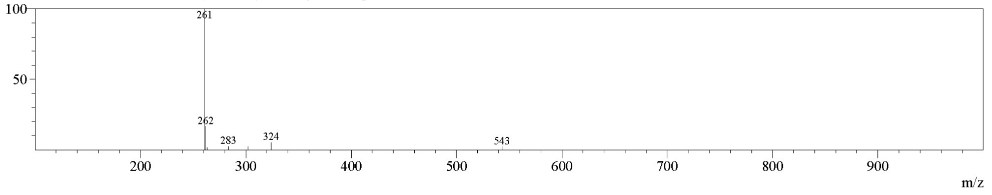

MS Spectrum Table

#:1 Ret.Time:
BG Mode:Calc 4.130<->4.540(414<->455)
Mass Peaks:8 Base Peak:261.15(1599192) Polarity:Pos Segment1 - Event1

#	m/z	Abs.Inten.	Rel.Inten.	Charge	Polarity	Monoisotopic	#	m/z	Abs.Inten.	Rel.Inten.	Charge	Polarity	Monoisotopic
1	261.15	1599192	100.00				5	302.25	35840	2.24			
2	262.15	263903	16.50				6	324.20	81342	5.09			
3	263.20	27666	1.73				7	543.45	34252	2.14			
4	283.15	36865	2.31				8	548.75	22480	1.41			

Figure S61. LCMS spectrum of compound **23**.

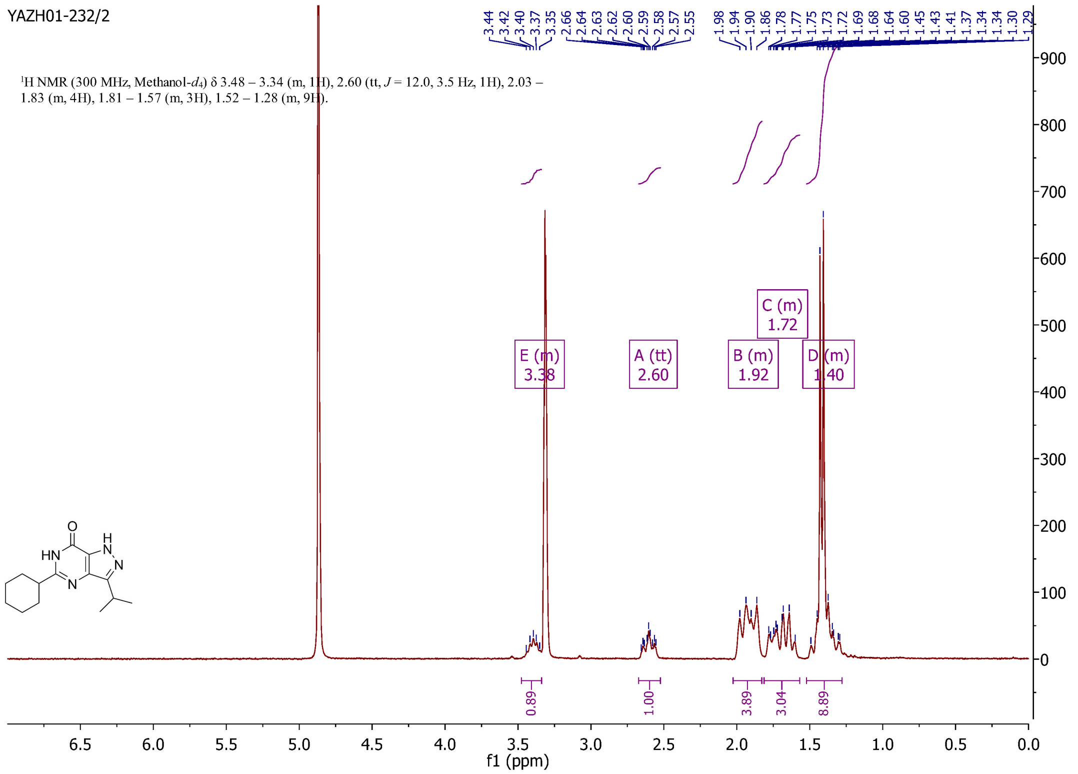

Figure S62. ^{1}H NMR spectrum of compound **23**.

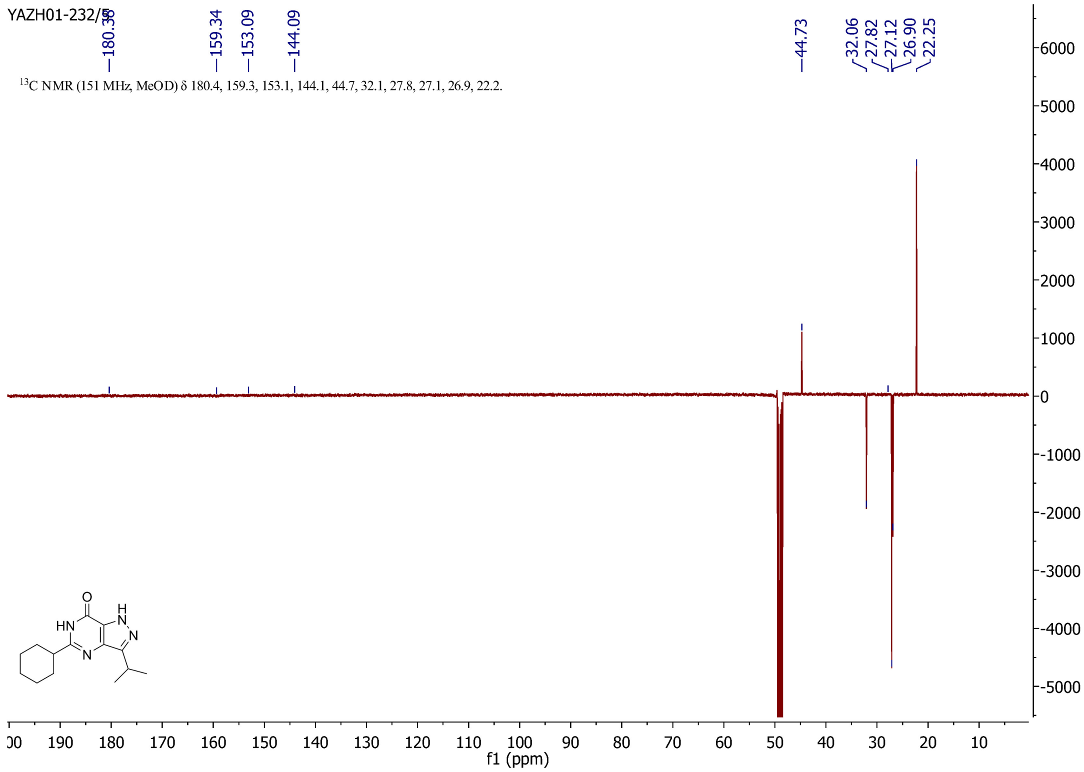

Figure S63. ^{13}C NMR spectrum of compound **23**.

Acquired by	: Admin
Date Acquired	: 8/11/2018 10:22:48 AM
Sample Name	: YAZH01-275
Sample ID	:
Tray#	: 1
Vial#	: 13
Injection Volume	: 5
Data File	: C:\LabSolutions\Data\2018\2018-wk45\YAZH01-275.lcd
Background File	: blanco 08112018.lcd
Method File	: Method SCAN ACID standard.lcm
Report Format	: DefaultLCMS.lcr
Tuning File	: C:\LabSolutions\Tuning\Tuning-ESI-pos-neg01072015a.lct
Processed by	: Admin
Modified Date	: 8/11/2018 10:43:08 AM

Chromatogram
YAZH01-275 C:\LabSolutions\Data\2018\2018-wk45\YAZH01-275.lcd

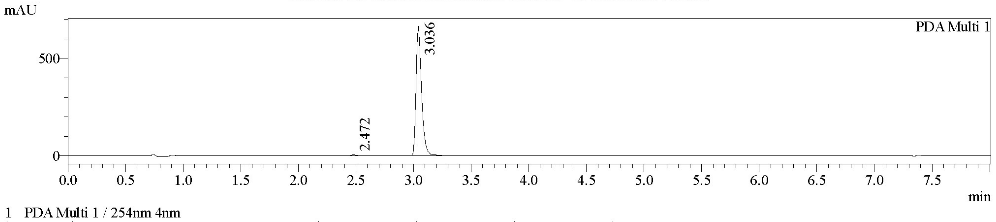

1 PDA Multi 1 / 254nm 4nm

Peak#	Name	Ret. Time	Area	Area %
1		2.472	15653	0.706
2		3.036	2201037	99.294

MS Chromatogram
YAZH01-275 C:\LabSolutions\Data\2018\2018-wk45\YAZH01-275.lcd

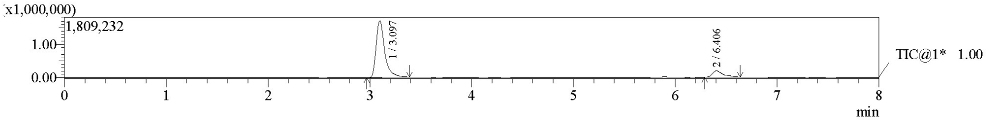

MS Spectrum Graph

#:1 Ret.Time:Averaged 3.090-3.110(Scan#:310-312)
Mass Peaks:9 Base Peak:263.15(1277053) Polarity:Pos Segment1 - Event1

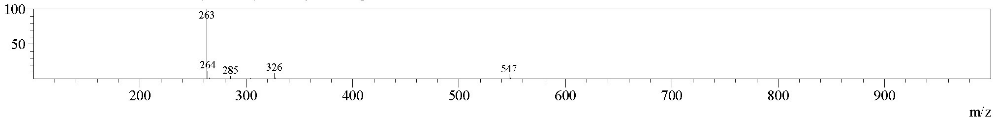

MS Spectrum Table

#:1 Ret.Time:
BG Mode:Calc 2.970<->3.390(298<->340)
Mass Peaks:9 Base Peak:263.15(1277053) Polarity:Pos Segment1 - Event1

#	m/z	Abs.Inten.	Rel.Inten.	Charge	Polarity	Monoisotopic
1	263.15	1277053	100.00			
2	264.10	148539	11.63			
3	265.15	20956	1.64			
4	285.10	50169	3.93			
5	304.20	13827	1.08			

#	m/z	Abs.Inten.	Rel.Inten.	Charge	Polarity	Monoisotopic
6	326.15	106660	8.35			
7	327.10	19079	1.49			
8	547.25	80385	6.29			
9	548.35	17444	1.37			

Figure S64. LCMS spectrum of compound **24**.

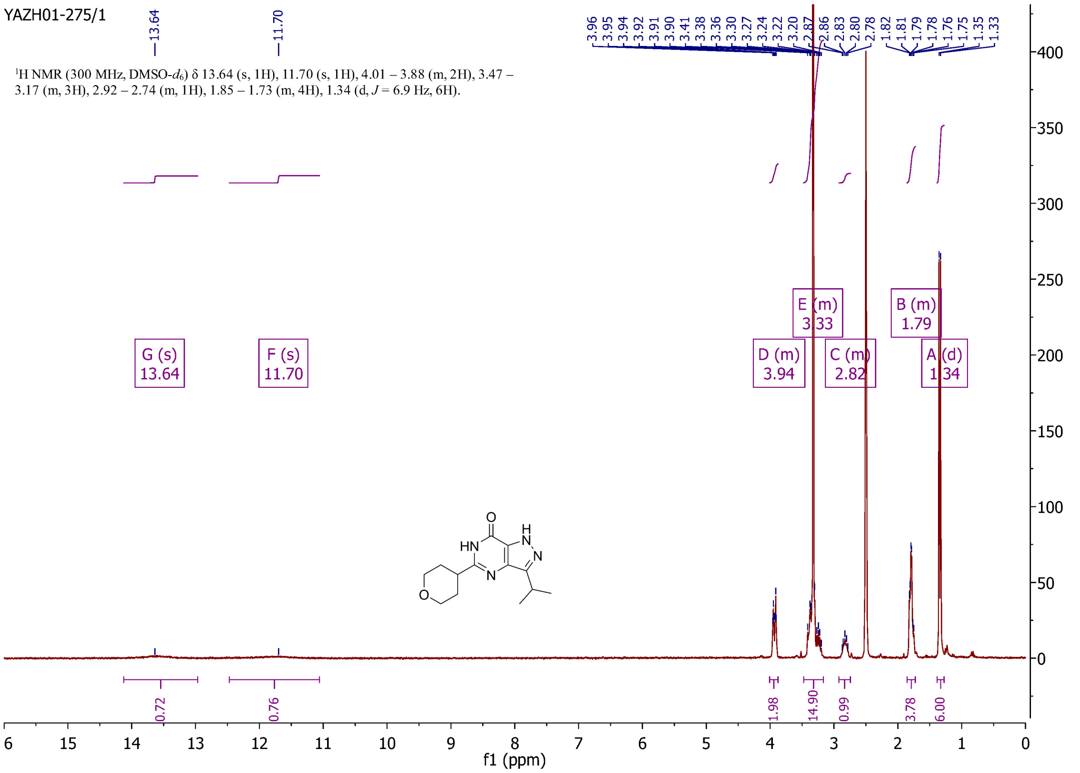

Figure S65. ^{1}H NMR spectrum of compound **24**.

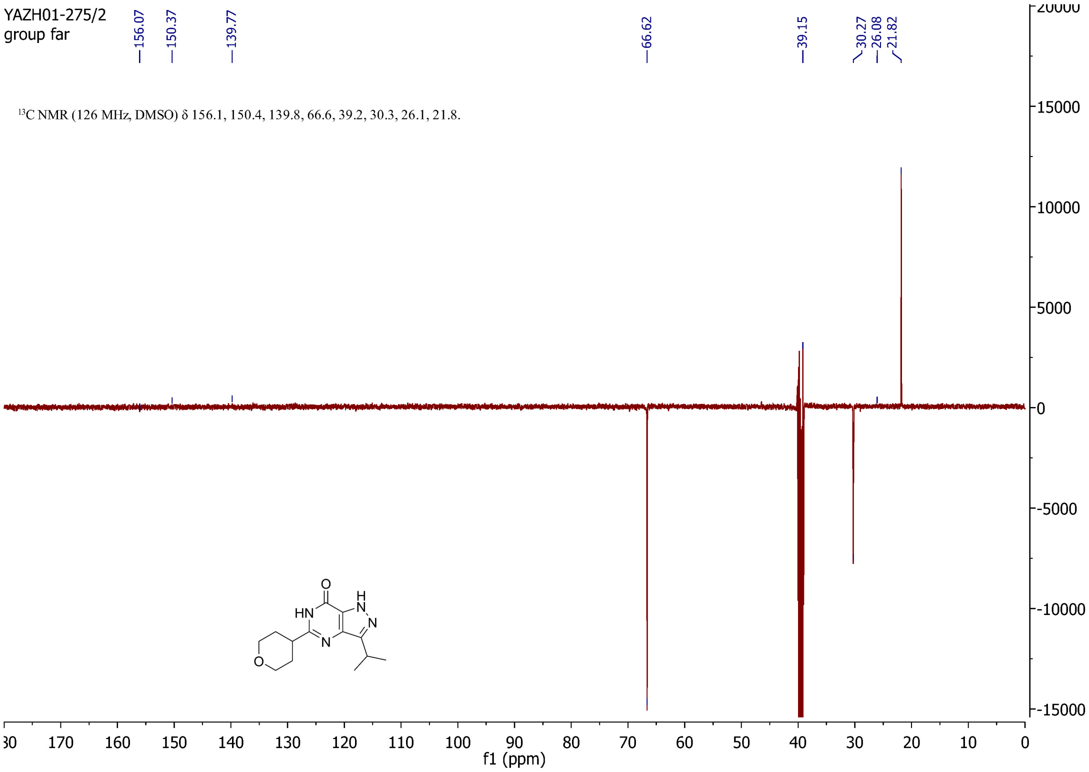

Figure S66. ^{13}C NMR spectrum of compound **24**.

Acquired by	: Admin
Date Acquired	: 5/4/2018 5:27:19 PM
Sample Name	: YAZH01-194
Sample ID	:
Tray#	: 1
Vial#	: 14
Injection Volume	: 3
Data File	: C:\LabSolutions\Data\2018\2018-wk14\YAZH01-194.lcd
Background File	: blanco 05042018.lcd
Method File	: Method SCAN ACID standard.lcm
Report Format	: DefaultLCMS.lcr
Tuning File	: C:\LabSolutions\Tuning\Tuning-ESI-pos-neg01072015a.lct
Processed by	: Admin
Modified Date	: 6/4/2018 9:00:06 AM

PDA Graph

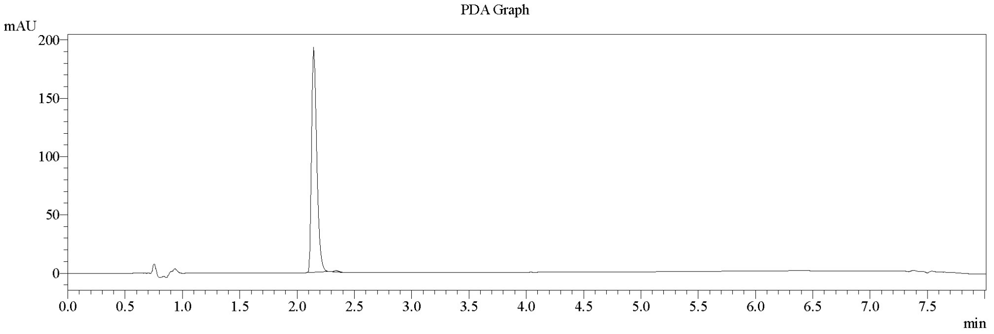

PDA Ch1 254nm 4nm

Peak#	Name	Ret. Time	Area	Area %
1		2.140	605229	99.419
2		2.335	3536	0.581

MS Spectrum Graph

#:1 Ret.Time:Averaged 2.190-2.210(Scan#:220-222)
Mass Peaks:8 Base Peak:276.10(636136) Polarity:Pos Segment1 - Event1

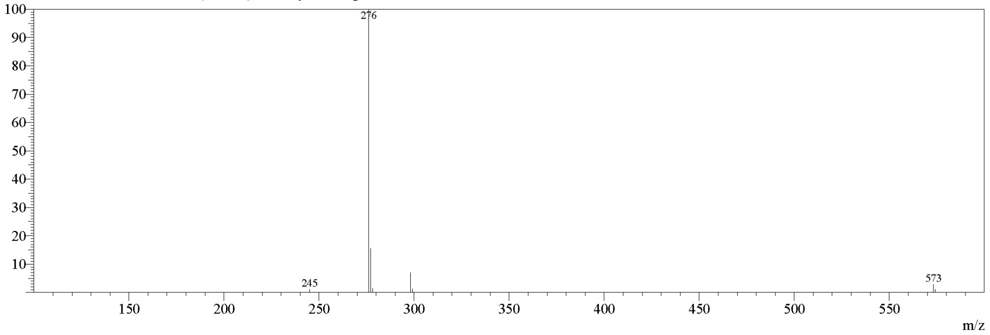

MS Spectrum Table

#:1 Ret.Time:
BG Mode:Calc 2.100<->2.350(211<->236)
Mass Peaks:8 Base Peak:276.10(636136) Polarity:Pos Segment1 - Event1

#	m/z	Abs.Inten.	Rel.Inten.	Charge	Polarity	Monoisotopic
1	245.05	7520	1.18			
2	276.10	636136	100.00			
3	277.10	98297	15.45			
4	278.15	8866	1.39			
5	298.10	44279	6.96			
6	299.15	8104	1.27			
7	573.25	17879	2.81			
8	574.25	6830	1.07			

Figure S67. LCMS spectrum of compound **25**.

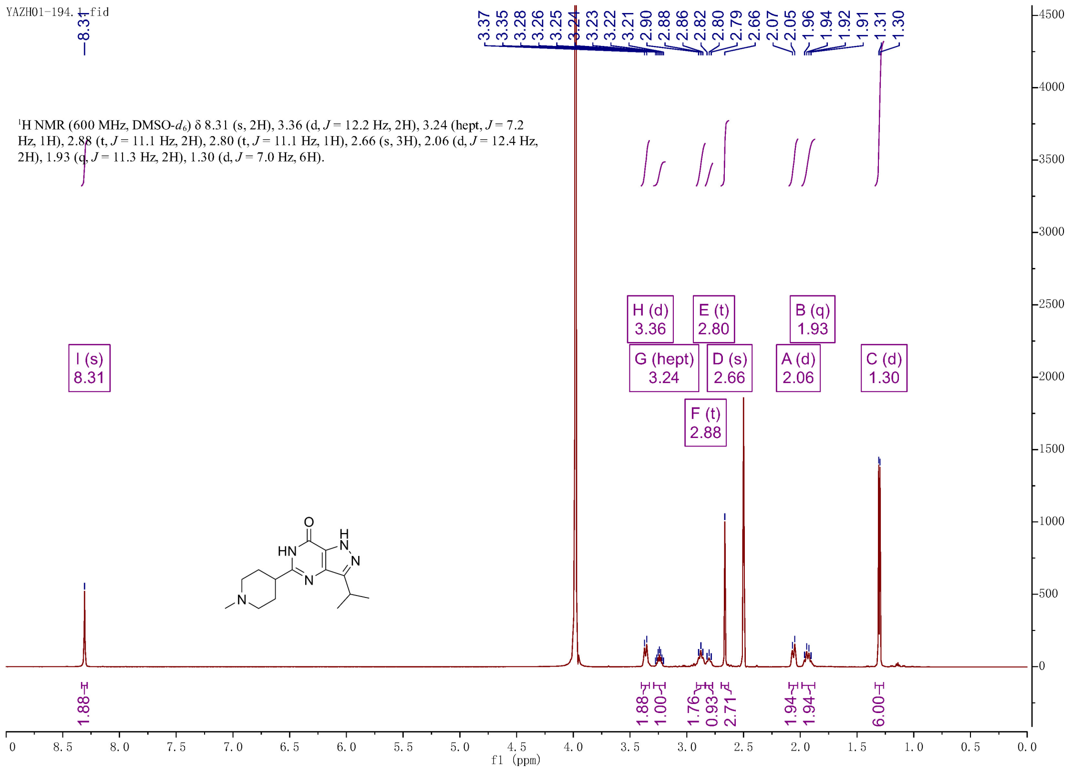

Figure S68. ^{1}H NMR spectrum of compound **25**.

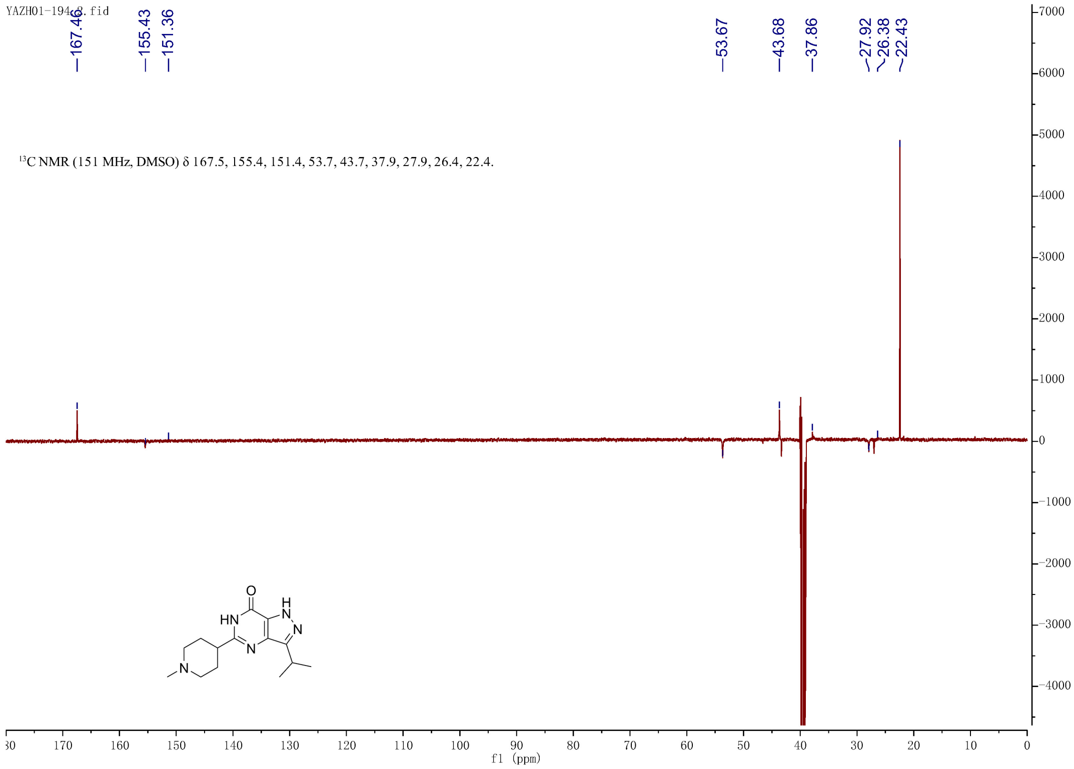

Figure S69. ^{13}C NMR spectrum of compound **25**.

Acquired by : Admin
Date Acquired : 7/12/2018 10:21:27 AM
Sample Name : YAZH01-276-P1
Sample ID :
Tray# : 1
Vial# : 7
Injection Volume : 3
Data File : C:\LabSolutions\Data\2018\2018-wk49\YAZH01-276-P1.lcd
Background File : blanco 07122018.lcd
Method File : Method SCAN ACID standard MW50.lcm
Report Format : DefaultLCMS.lcr
Tuning File : C:\LabSolutions\Tuning\Tuning-ESI-pos-neg01072015a.lct
Processed by : Admin
Modified Date : 7/12/2018 10:37:14 AM

Chromatogram
YAZH01-276-P1 C:\LabSolutions\Data\2018\2018-wk49\YAZH01-276-P1.lcd

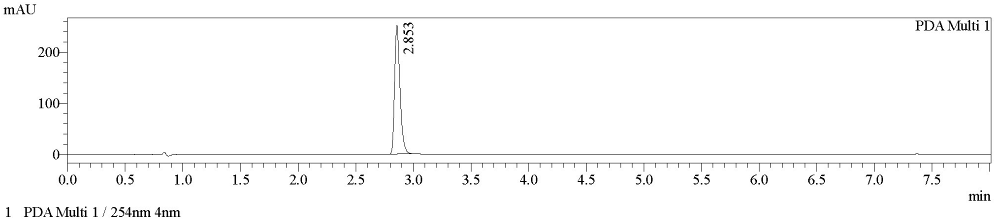

1 PDA Multi 1 / 254nm 4nm

PeakTabl

PDA Ch1 254nm 4nm

Peak#	Name	Ret. Time	Area	Area %
1		2.853	807774	100.000

MS Chromatogram
YAZH01-276-P1 C:\LabSolutions\Data\2018\2018-wk49\YAZH01-276-P1.lcd

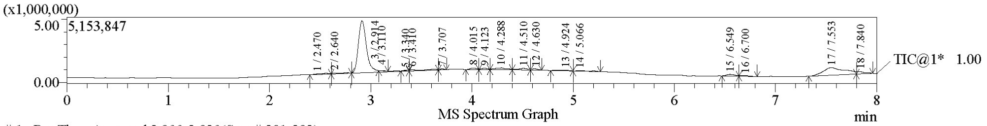

MS Spectrum Graph

#:1 Ret.Time:Averaged 2.900-2.920(Scan#:291-293)
Mass Peaks:9 Base Peak:277.10(2838476) Polarity:Pos Segment1 - Event1

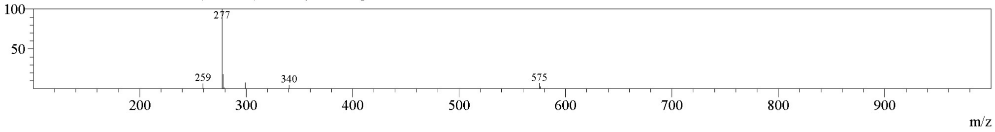

MS Spectrum Table

#:1 Ret.Time:
BG Mode:Calc 2.810<->3.380(282<->339)
Mass Peaks:9 Base Peak:277.10(2838476) Polarity:Pos Segment1 - Event1

#	m/z	Abs.Inten.	Rel.Inten.	Charge	Polarity	Monoisotopic
1	259.15	177815	6.26			
2	260.10	28686	1.01			
3	277.10	2838476	100.00			
4	278.10	508217	17.90			
5	279.15	46144	1.63			
6	299.10	215360	7.59			
7	340.15	128198	4.52			
8	575.35	194211	6.84			
9	576.40	75235	2.65			

Figure S70. LCMS spectrum of compound **26**.

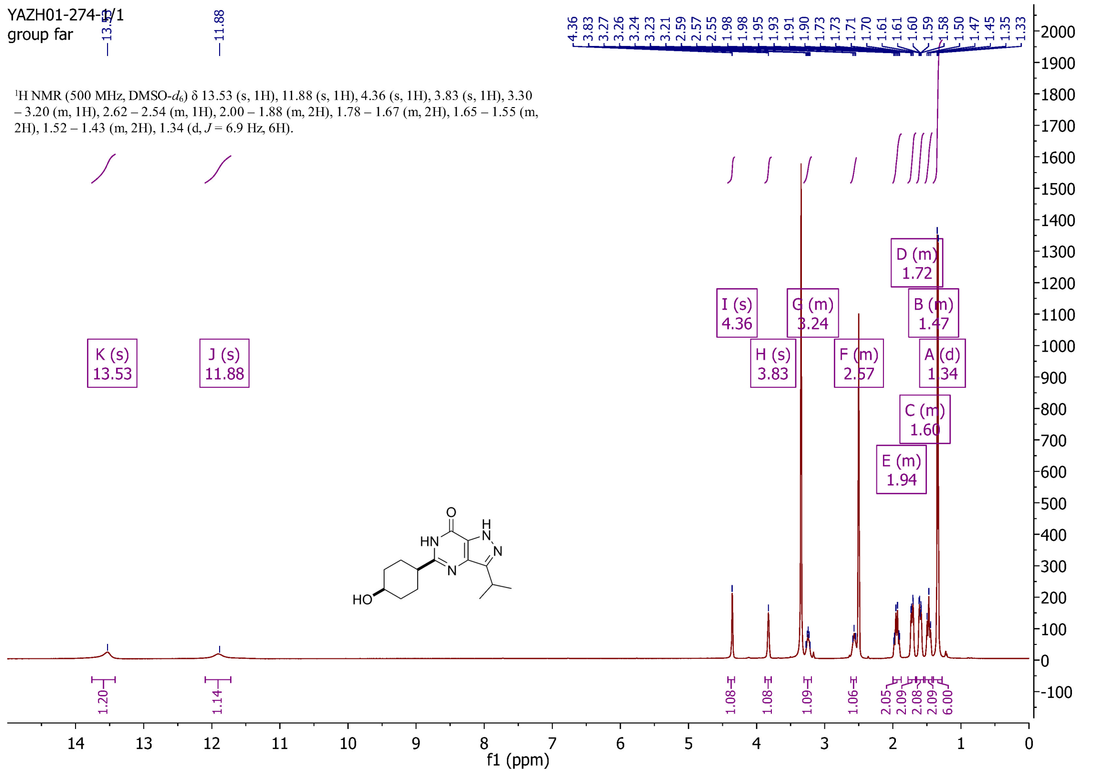

Figure S71. ^{1}H NMR spectrum of compound **26**.

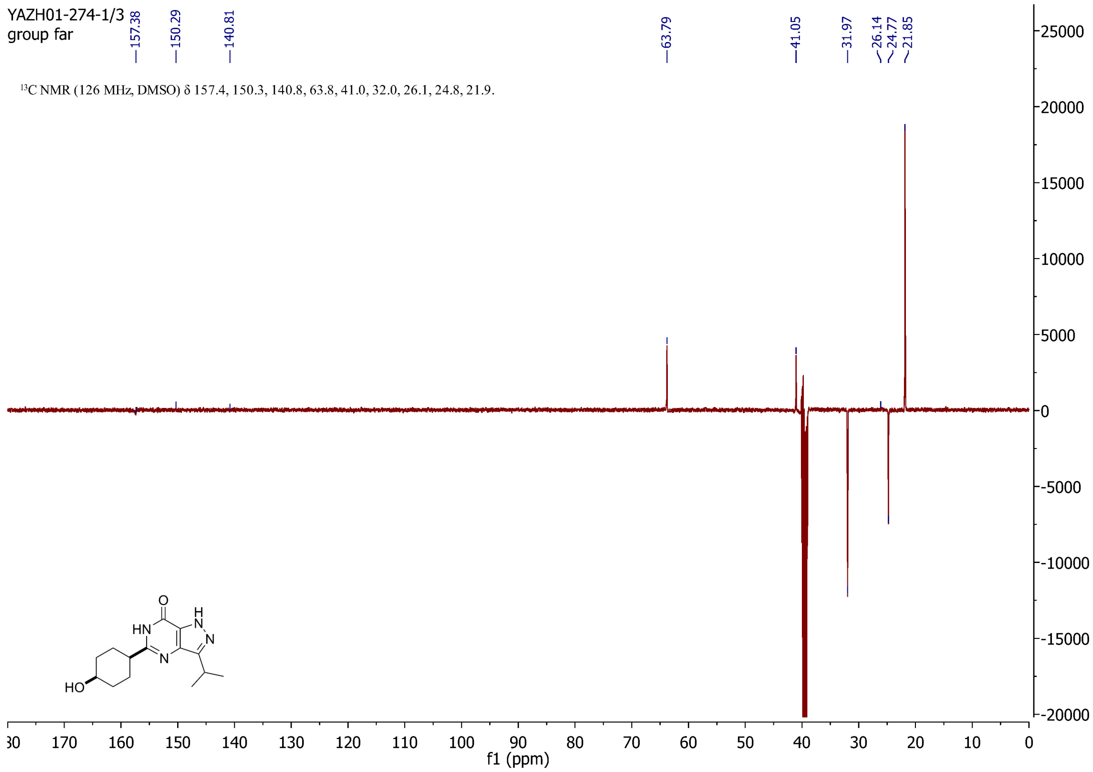

Figure S72. ^{13}C NMR spectrum of compound **26**.

Acquired by	: Admin
Date Acquired	: 7/12/2018 10:30:05 AM
Sample Name	: YAZH01-276-P2
Sample ID	:
Tray#	: 1
Vial#	: 8
Injection Volume	: 3
Data File	: C:\LabSolutions\Data\2018\2018-wk49\YAZH01-276-P2.lcd
Background File	: blanco 07122018.lcd
Method File	: Method SCAN ACID standard MW50.lcm
Report Format	: DefaultLCMS.lcr
Tuning File	: C:\LabSolutions\Tuning\Tuning-ESI-pos-neg01072015a.lct
Processed by	: Admin
Modified Date	: 7/12/2018 10:38:36 AM

Chromatogram
YAZH01-276-P2 C:\LabSolutions\Data\2018\2018-wk49\YAZH01-276-P2.lcd

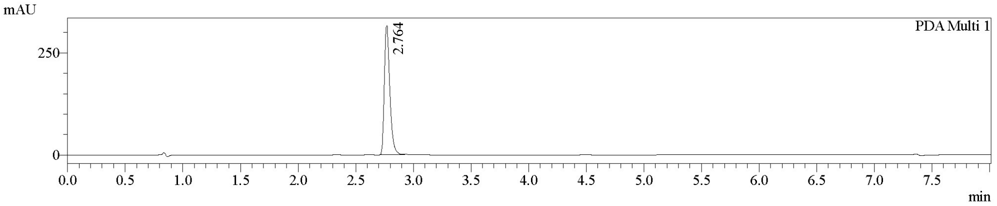

1 PDA Multi 1 / 254nm 4nm

PeakTabl

PDA Ch1 254nm 4nm

Peak#	Name	Ret. Time	Area	Area %
1		2.764	1036667	100.000

MS Chromatogram
YAZH01-276-P2 C:\LabSolutions\Data\2018\2018-wk49\YAZH01-276-P2.lcd

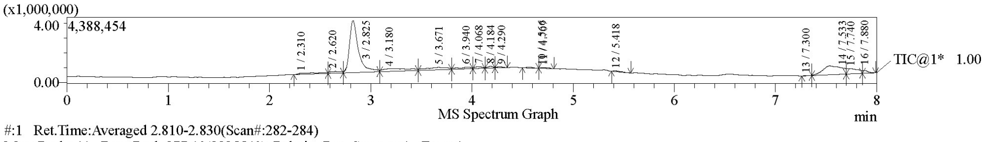

MS Spectrum Graph

#:1 Ret.Time:Averaged 2.810-2.830(Scan#:282-284)
Mass Peaks:11 Base Peak:277.10(2235540) Polarity:Pos Segment1 - Event1

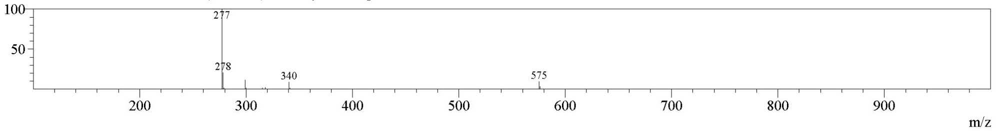

MS Spectrum Table

#:1 Ret.Time:
BG Mode:Calc 2.730<->3.090(274<->310)
Mass Peaks:11 Base Peak:277.10(2235540) Polarity:Pos Segment1 - Event1

#	m/z	Abs.Inten.	Rel.Inten.	Charge	Polarity	Monoisotopic	#	m/z	Abs.Inten.	Rel.Inten.	Charge	Polarity	Monoisotopic
1	277.10	2235540	100.00				7	318.15	46313	2.07			
2	278.15	459389	20.55				8	340.15	198712	8.89			
3	279.10	39279	1.76				9	341.05	28770	1.29			
4	299.10	257502	11.52				10	575.35	213346	9.54			
5	300.15	31026	1.39				11	576.40	73707	3.30			
6	315.15	35250	1.58										

Figure S73. LCMS spectrum of compound **27**.

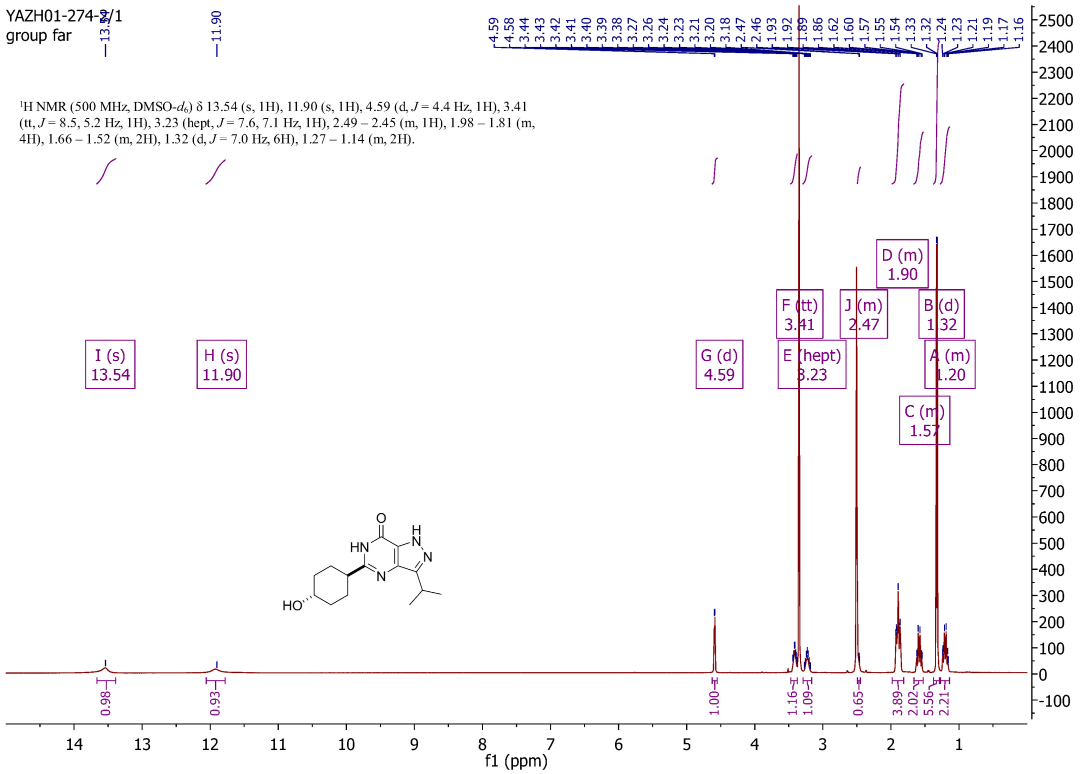

Figure S74. ^{1}H NMR spectrum of compound **27**.

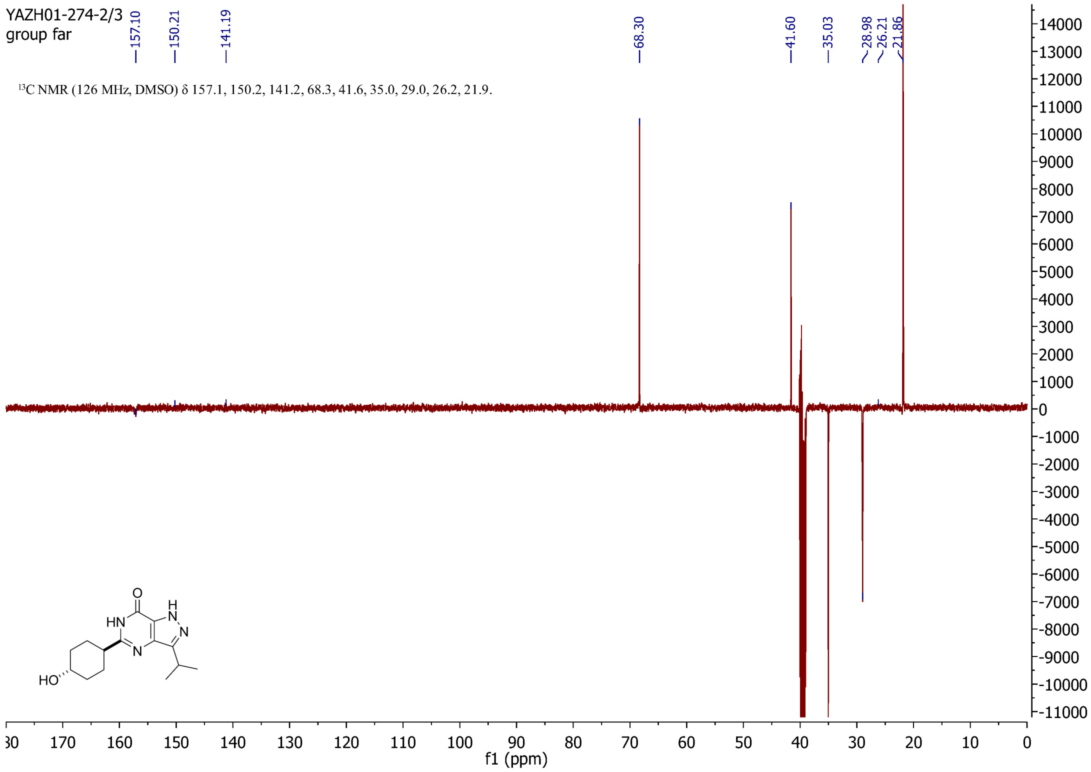

Figure S75. ^{13}C NMR spectrum of compound **27**.

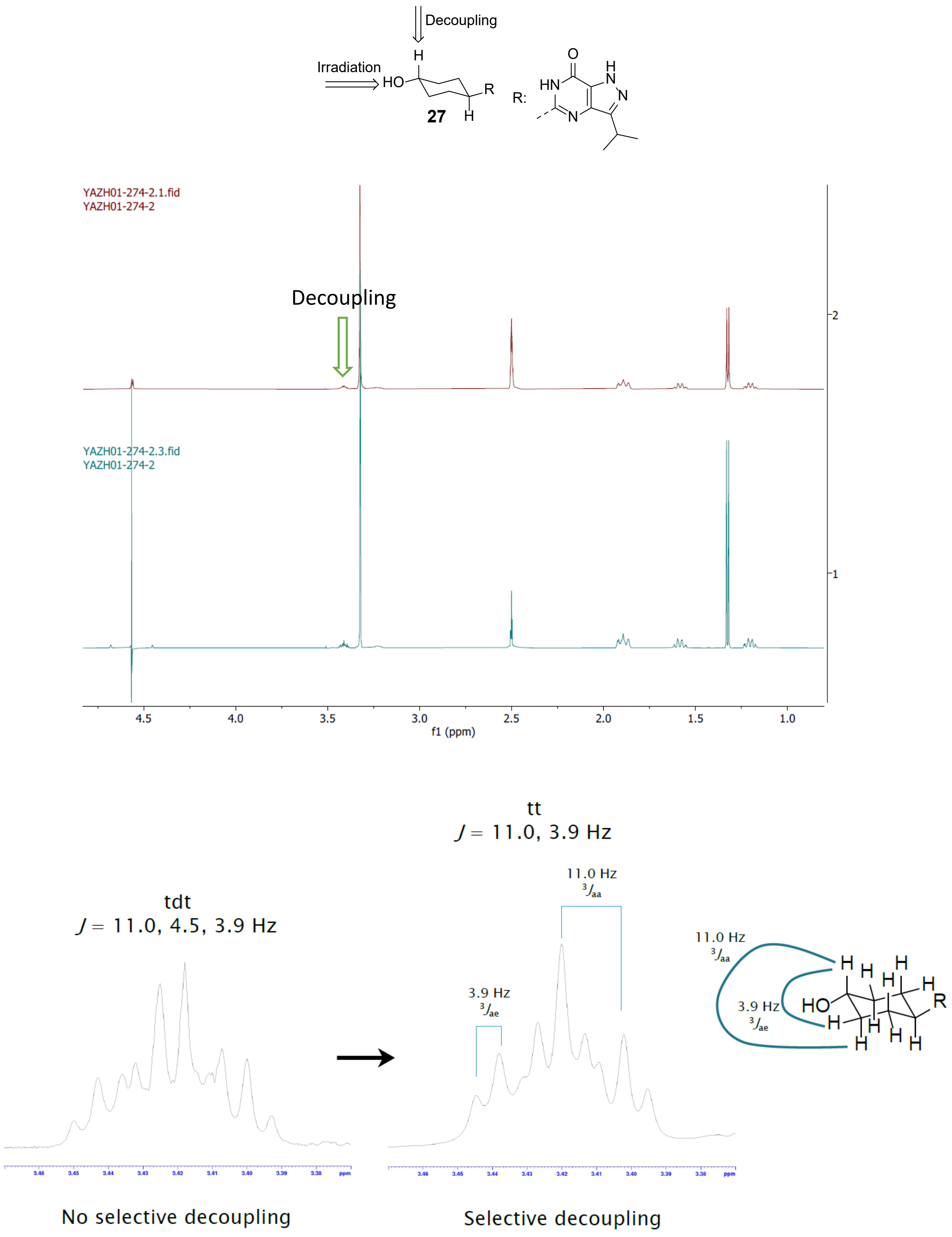

Figure S76. ^{1}H NMR spectrum of compound **27** before (red, left) and after decoupling (green, right).

Acquired by	: Admin
Date Acquired	: 8/11/2018 10:31:25 AM
Sample Name	: YAZH01-277
Sample ID	:
Tray#	: 1
Vial#	: 14
Injection Volume	: 5
Data File	: C:\LabSolutions\Data\2018\2018-wk45\YAZH01-277.lcd
Background File	: blanco 08112018.lcd
Method File	: Method SCAN ACID standard.lcm
Report Format	: DefaultLCMS.lcr
Tuning File	: C:\LabSolutions\Tuning\Tuning-ESI-pos-neg01072015a.lct
Processed by	: Admin
Modified Date	: 8/11/2018 10:44:00 AM

Chromatogram
YAZH01-277 C:\LabSolutions\Data\2018\2018-wk45\YAZH01-277.lcd

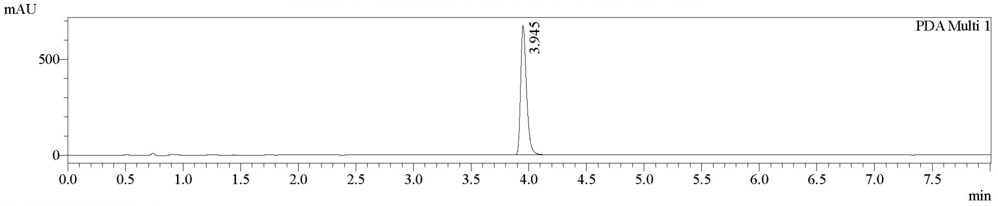

1 PDA Multi 1 / 254nm 4nm

Peak#	Name	Ret. Time	Area	Area %
1		3.945	2382178	100.000

MS Chromatogram
YAZH01-277 C:\LabSolutions\Data\2018\2018-wk45\YAZH01-277.lcd

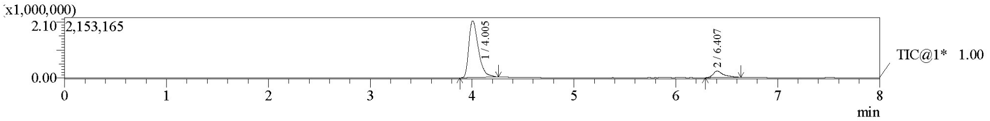

MS Spectrum Graph
#:1 Ret.Time:Averaged 3.990-4.010(Scan#:400-402)
Mass Peaks:12 Base Peak:297.10(1233911) Polarity:Pos Segment1 - Event1

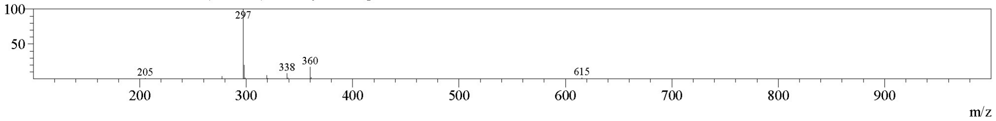

MS Spectrum Table
#:1 Ret.Time:
BG Mode:Calc 3.880<->4.260(389<->427)
Mass Peaks:12 Base Peak:297.10(1233911) Polarity:Pos Segment1 - Event1

#	m/z	Abs.Inten.	Rel.Inten.	Charge	Polarity	Monoisotopic
1	205.05	12632	1.02			
2	277.10	48769	3.95			
3	297.10	1233911	100.00			
4	298.10	244714	19.83			
5	299.15	20673	1.68			
6	319.15	65083	5.27			

#	m/z	Abs.Inten.	Rel.Inten.	Charge	Polarity	Monoisotopic
7	320.15	13279	1.08			
8	338.15	102768	8.33			
9	339.10	19654	1.59			
10	360.15	210847	17.09			
11	361.15	29914	2.42			
12	615.30	21819	1.77			

Figure S77. LCMS spectrum of compound **28**.

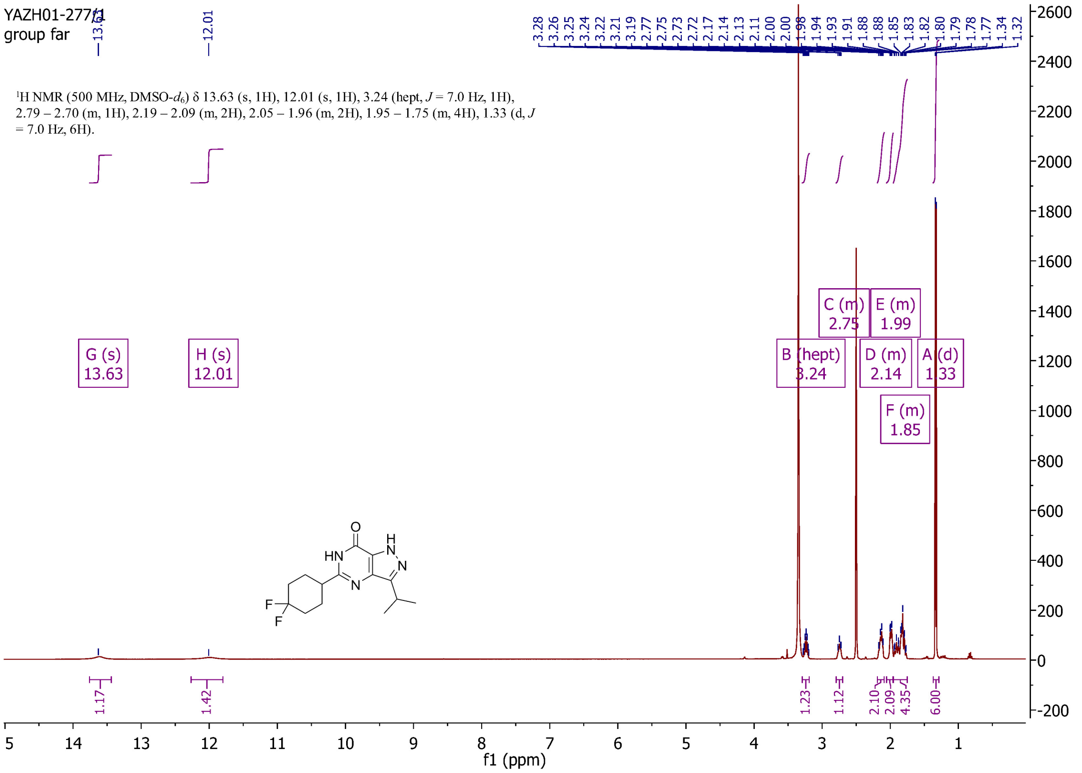

Figure S78. ^{1}H NMR spectrum of compound **28**.

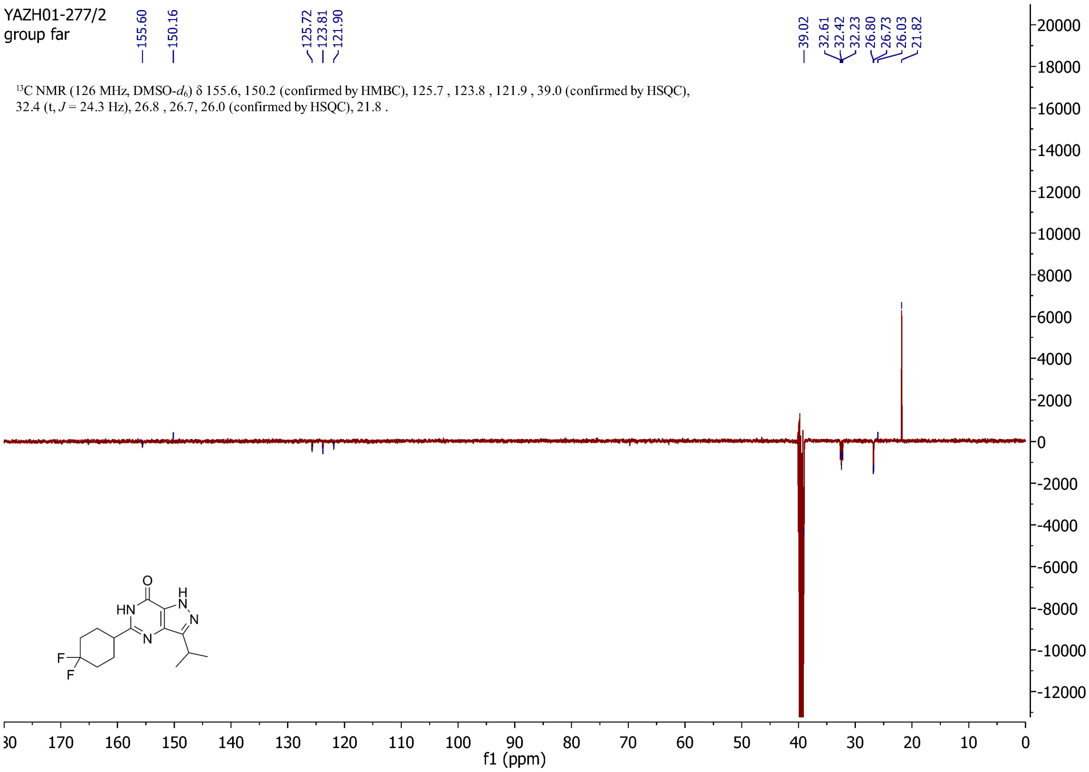

Figure S79. ^{13}C NMR spectrum of compound **28**.

Acquired by : Admin
Date Acquired : 8/11/2018 10:48:39 AM
Sample Name : YAZH01-278
Sample ID :
Tray# : 1
Vial# : 15
Injection Volume : 5
Data File : C:\LabSolutions\Data\2018\2018-wk45\YAZH01-278.lcd
Background File : blanco 08112018.lcd
Method File : Method SCAN ACID standard.lcm
Report Format : DefaultLCMS.lcr
Tuning File : C:\LabSolutions\Tuning\Tuning-ESI-pos-neg01072015a.lct
Processed by : Admin
Modified Date : 8/11/2018 12:52:21 PM

Chromatogram
YAZH01-278 C:\LabSolutions\Data\2018\2018-wk45\YAZH01-278.lcd

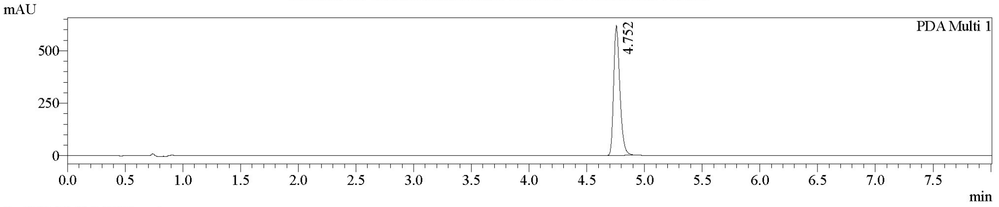

1 PDA Multi 1 / 254nm 4nm

Peak#	Name	Ret. Time	Area	Area %
1		4.752	2298190	100.000

MS Chromatogram
YAZH01-278 C:\LabSolutions\Data\2018\2018-wk45\YAZH01-278.lcd

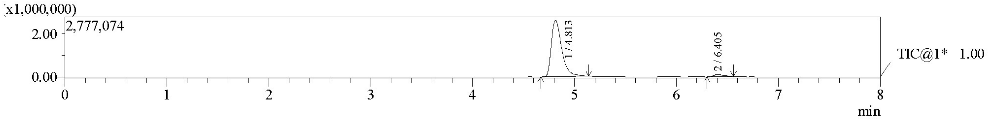

MS Spectrum Graph
#:1 Ret.Time:Averaged 4.800-4.820(Scan#:481-483)
Mass Peaks:10 Base Peak:287.15(1711185) Polarity:Pos Segment1 - Event1

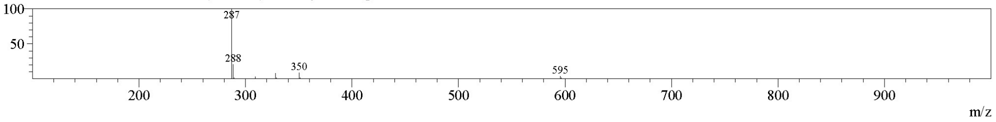

MS Spectrum Table
#:1 Ret.Time:
BG Mode:Calc 4.670<->5.140(468<->515)
Mass Peaks:10 Base Peak:287.15(1711185) Polarity:Pos Segment1 - Event1

#	m/z	Abs.Inten.	Rel.Inten.	Charge	Polarity	Monoisotopic	#	m/z	Abs.Inten.	Rel.Inten.	Charge	Polarity	Monoisotopic
1	287.15	1711185	100.00				6	329.10	31704	1.85			
2	288.15	355026	20.75				7	350.20	150225	8.78			
3	289.15	32716	1.91				8	351.15	30744	1.80			
4	309.10	48299	2.82				9	595.40	70389	4.11			
5	328.20	142971	8.36				10	596.35	26376	1.54			

Figure S80. LCMS spectrum of compound **29**.

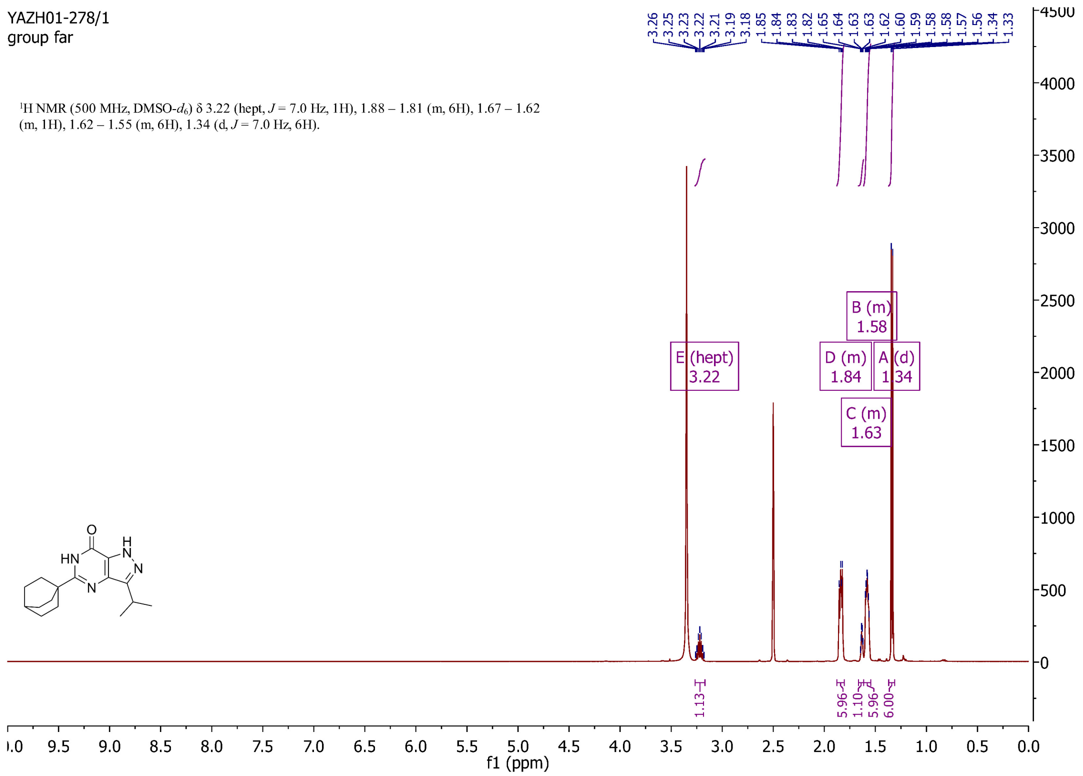

Figure S81. ^{1}H NMR spectrum of compound **29**.

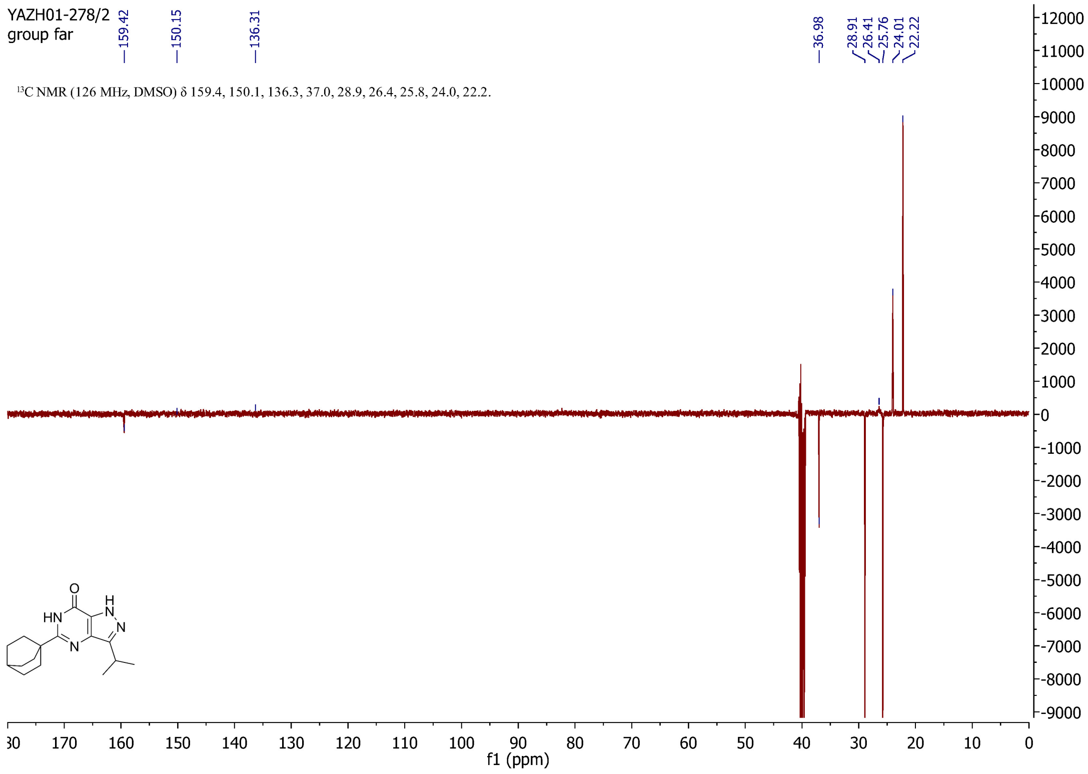

Figure S82. ^{13}C NMR spectrum of compound **29**.

Acquired by : Admin
Date Acquired : 8/11/2018 10:57:20 AM
Sample Name : YAZH01-279
Sample ID :
Tray# : 1
Vial# : 16
Injection Volume : 5
Data File : C:\LabSolutions\Data\2018\2018-wk45\YAZH01-279.lcd
Background File : blanco 08112018.lcd
Method File : Method SCAN ACID standard.lcm
Report Format : DefaultLCMS.lcr
Tuning File : C:\LabSolutions\Tuning\Tuning-ESI-pos-neg01072015a.lct
Processed by : Admin
Modified Date : 8/11/2018 12:53:33 PM

Chromatogram
YAZH01-279 C:\LabSolutions\Data\2018\2018-wk45\YAZH01-279.lcd

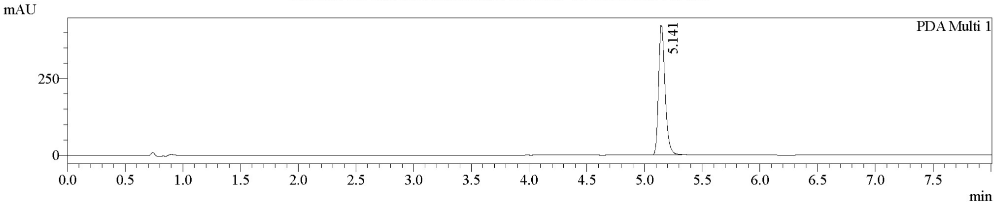

1 PDA Multi 1 / 254nm 4nm

Peak#	Name	Ret. Time	Area	Area %
1		5.141	1644561	100.000

MS Chromatogram
YAZH01-279 C:\LabSolutions\Data\2018\2018-wk45\YAZH01-279.lcd

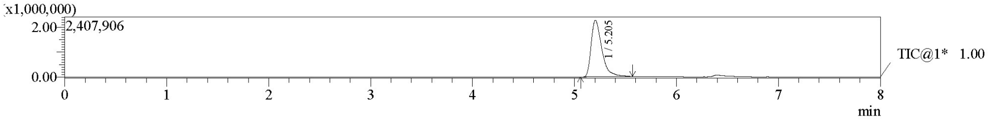

MS Spectrum Graph
#:1 Ret.Time:Averaged 5.190-5.210(Scan#:520-522)
Mass Peaks:10 Base Peak:313.10(1480473) Polarity:Pos Segment1 - Event1

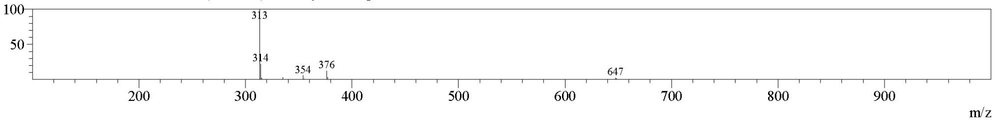

MS Spectrum Table
#:1 Ret.Time:
BG Mode:Calc 5.060<->5.570(507<->558)
Mass Peaks:10 Base Peak:313.10(1480473) Polarity:Pos Segment1 - Event1

#	m/z	Abs.Inten.	Rel.Inten.	Charge	Polarity	Monoisotopic
1	313.10	1480473	100.00			
2	314.15	325362	21.98			
3	315.20	34953	2.36			
4	335.20	50531	3.41			
5	354.20	83713	5.65			
6	355.20	18518	1.25			
7	376.20	185578	12.54			
8	377.20	42025	2.84			
9	647.45	38316	2.59			
10	648.40	24151	1.63			

Figure S83. LCMS spectrum of compound **30**.

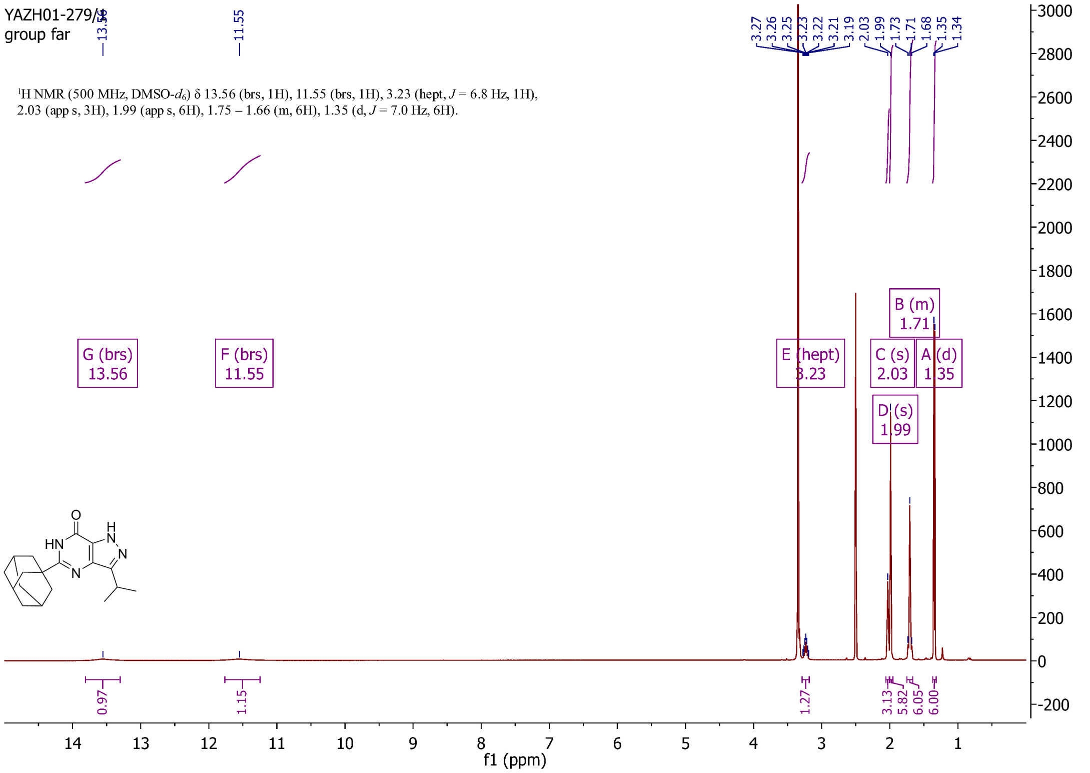

Figure S84. ^{1}H NMR spectrum of compound **30**.

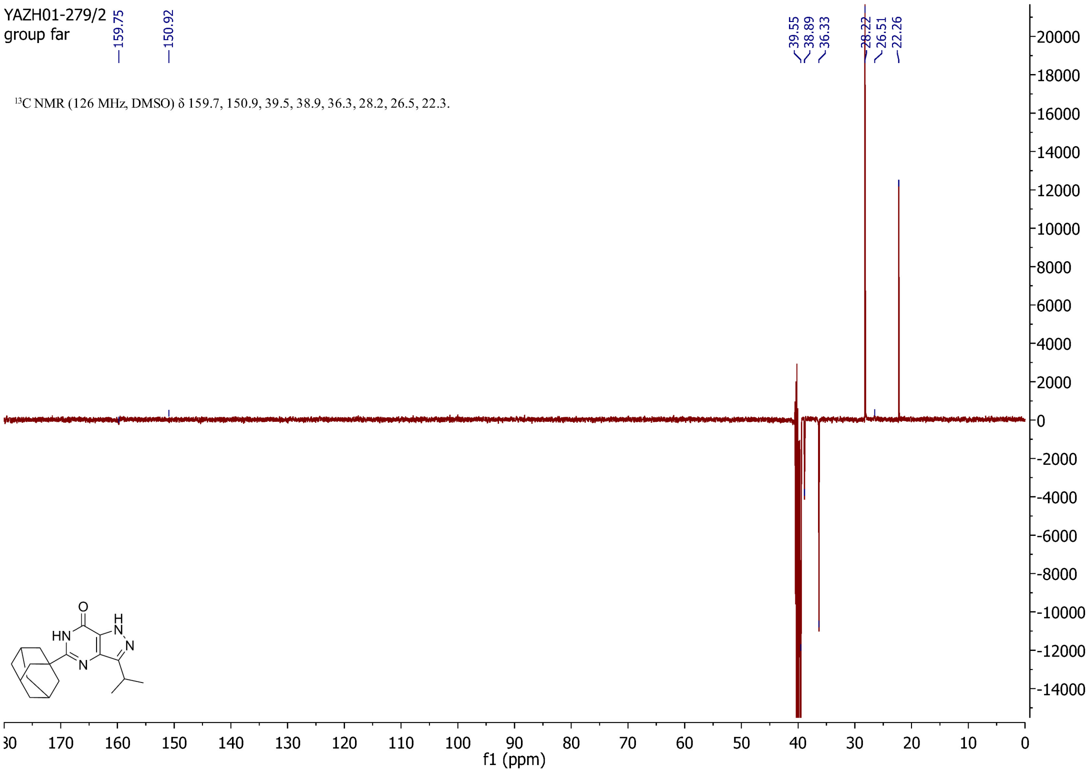

Figure S85. ^{13}C NMR spectrum of compound **30**.

Acquired by	: Admin
Date Acquired	: 20/12/2018 12:19:58 PM
Sample Name	: YAZH01-288-1
Sample ID	:
Tray#	: 1
Vial#	: 8
Injection Volume	: 5
Data File	: C:\LabSolutions\Data\2018\2018-wk51\YAZH01-288-1.lcd
Background File	: blanco 20122018.lcd
Method File	: Method SCAN ACID standard.lcm
Report Format	: DefaultLCMS.lcr
Tuning File	: C:\LabSolutions\Tuning\Tuning-ESI-pos-neg01072015a.lct
Processed by	: Admin
Modified Date	: 20/12/2018 2:52:13 PM

Chromatogram
YAZH01-288-1 C:\LabSolutions\Data\2018\2018-wk51\YAZH01-288-1.lcd

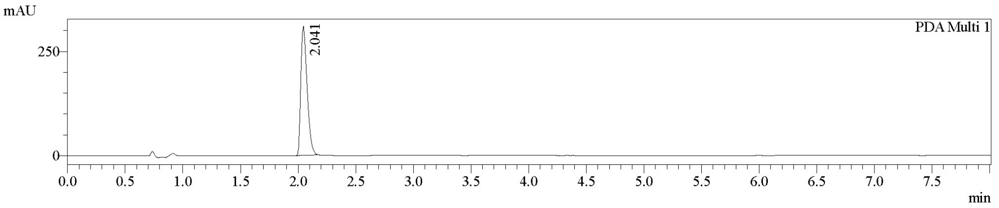

1 PDA Multi 1 / 254nm 4nm

PeakTabl

PDA Ch1 254nm 4nm

Peak#	Name	Ret. Time	Area	Area %
1		2.041	1163945	100.000

MS Chromatogram
YAZH01-288-1 C:\LabSolutions\Data\2018\2018-wk51\YAZH01-288-1.lcd

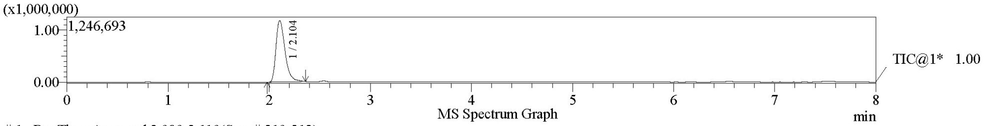

MS Spectrum Graph

#:1 Ret.Time:Averaged 2.090-2.110(Scan#:210-212)
Mass Peaks:8 Base Peak:262.15(897458) Polarity:Pos Segment1 - Event1

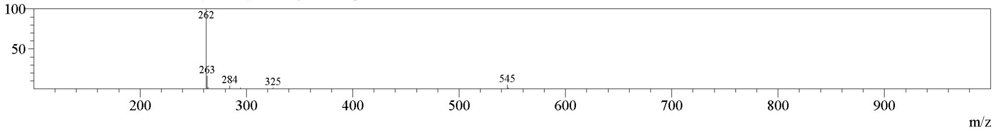

MS Spectrum Table

#:1 Ret.Time:
BG Mode:Calc 1.980<->2.360(199<->237)
Mass Peaks:8 Base Peak:262.15(897458) Polarity:Pos Segment1 - Event1

#	m/z	Abs.Inten.	Rel.Inten.	Charge	Polarity	Monoisotopic	#	m/z	Abs.Inten.	Rel.Inten.	Charge	Polarity	Monoisotopic
1	262.15	897458	100.00				5	294.20	11406	1.27			
2	263.15	145492	16.21				6	325.10	13609	1.52			
3	264.10	16532	1.84				7	545.35	45979	5.12			
4	284.15	36952	4.12				8	546.30	14333	1.60			

Figure S86. LCMS spectrum of compound **32**.

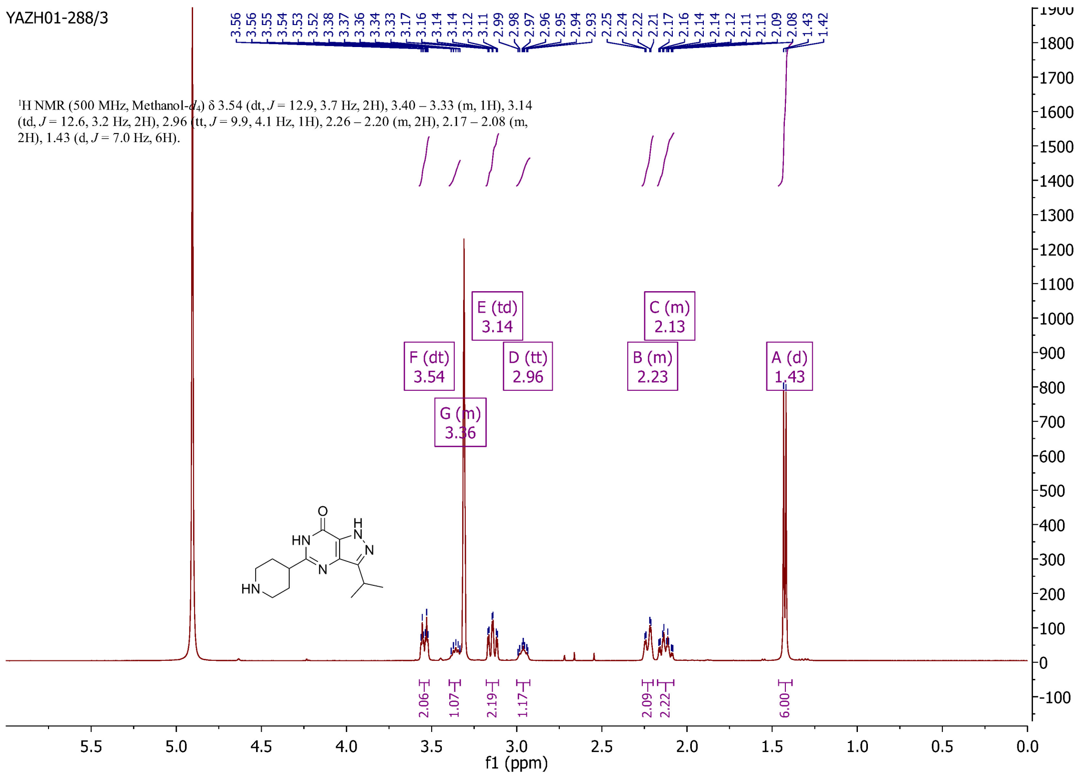

Figure S87. ^{1}H NMR spectrum of compound **32**.

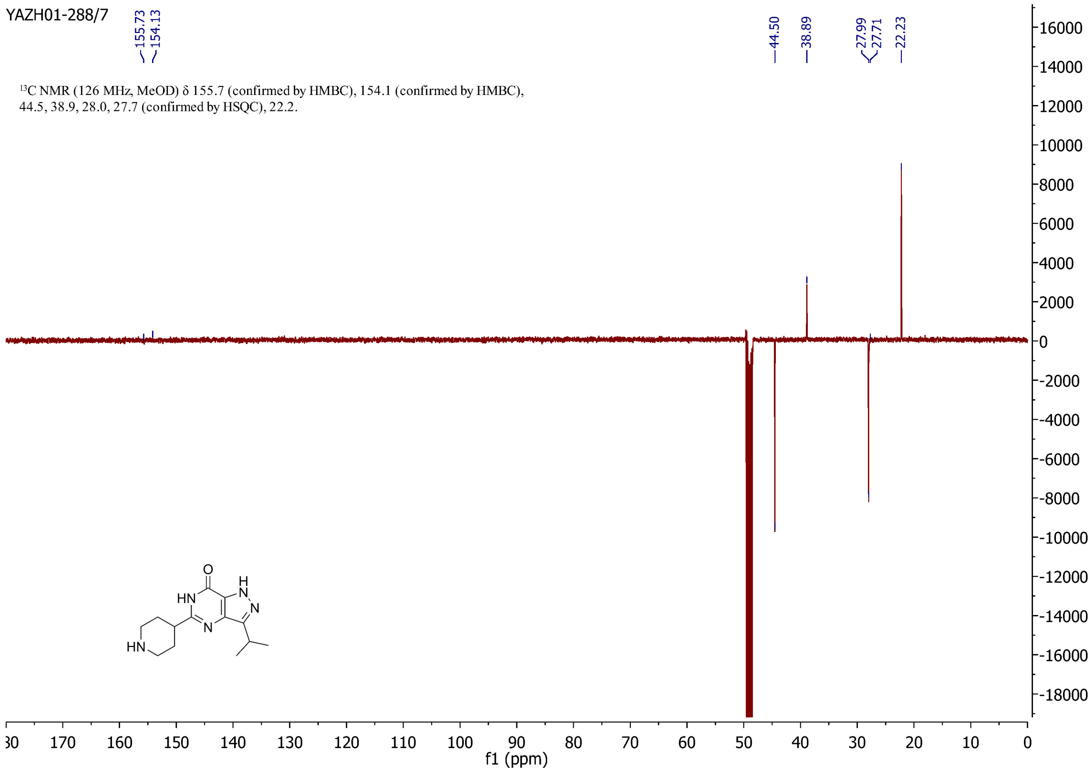

Figure S88. ^{13}C NMR spectrum of compound **32**.

Acquired by	: Admin
Date Acquired	: 3/9/2018 2:19:49 PM
Sample Name	: YAZH01-248-1
Sample ID	:
Tray#	: 1
Vial#	: 13
Injection Volume	: 1
Data File	: C:\LabSolutions\Data\2018\2018-wk36\YAZH01-248-1.lcd
Background File	: blanco 03092018.lcd
Method File	: Method SCAN ACID standard MW50.lcm
Report Format	: DefaultLCMS.lcr
Tuning File	: C:\LabSolutions\Tuning\Tuning-ESI-pos-neg01072015a.lct
Processed by	: Admin
Modified Date	: 3/9/2018 2:52:36 PM

Chromatogram
YAZH01-248-1 C:\LabSolutions\Data\2018\2018-wk36\YAZH01-248-1.lcd

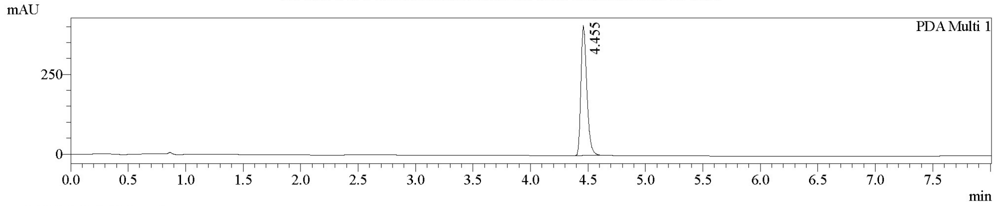

1 PDA Multi 1 / 254nm 4nm

Peak#	Name	Ret. Time	Area	Area %
1		4.455	1443459	100.000

MS Chromatogram
YAZH01-248-1 C:\LabSolutions\Data\2018\2018-wk36\YAZH01-248-1.lcd

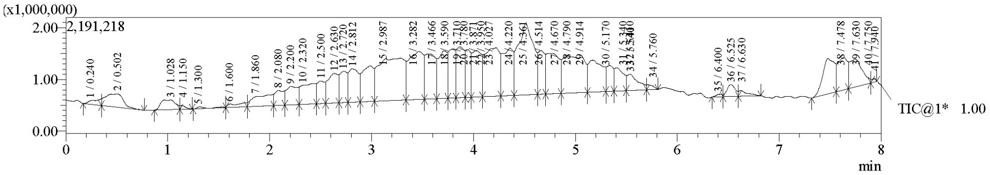

MS Spectrum Graph
#:1 Ret.Time:Averaged 4.500-4.520(Scan#:451-453)
Mass Peaks:11 Base Peak:228.05(343753) Polarity:Pos Segment1 - Event1

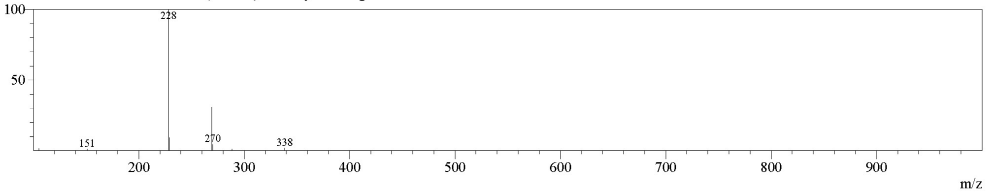

MS Spectrum Table

#:1 Ret.Time:
BG Mode:Calc 4.400<->4.630(441<->464)
Mass Peaks:11 Base Peak:228.05(343753) Polarity:Pos Segment1 - Event1

#	m/z	Abs.Inten.	Rel.Inten.	Charge	Polarity	Monoisotopic
1	59.05	9624	2.80			
2	83.05	95735	27.85			
3	84.05	3885	1.13			
4	105.05	5189	1.51			
5	151.00	3535	1.03			
6	228.05	343753	100.00			
7	229.05	31162	9.07			
8	269.15	106700	31.04			
9	270.15	15272	4.44			
10	288.30	3630	1.06			
11	338.30	5872	1.71			

Figure S89. LCMS spectrum of compound **34a**.

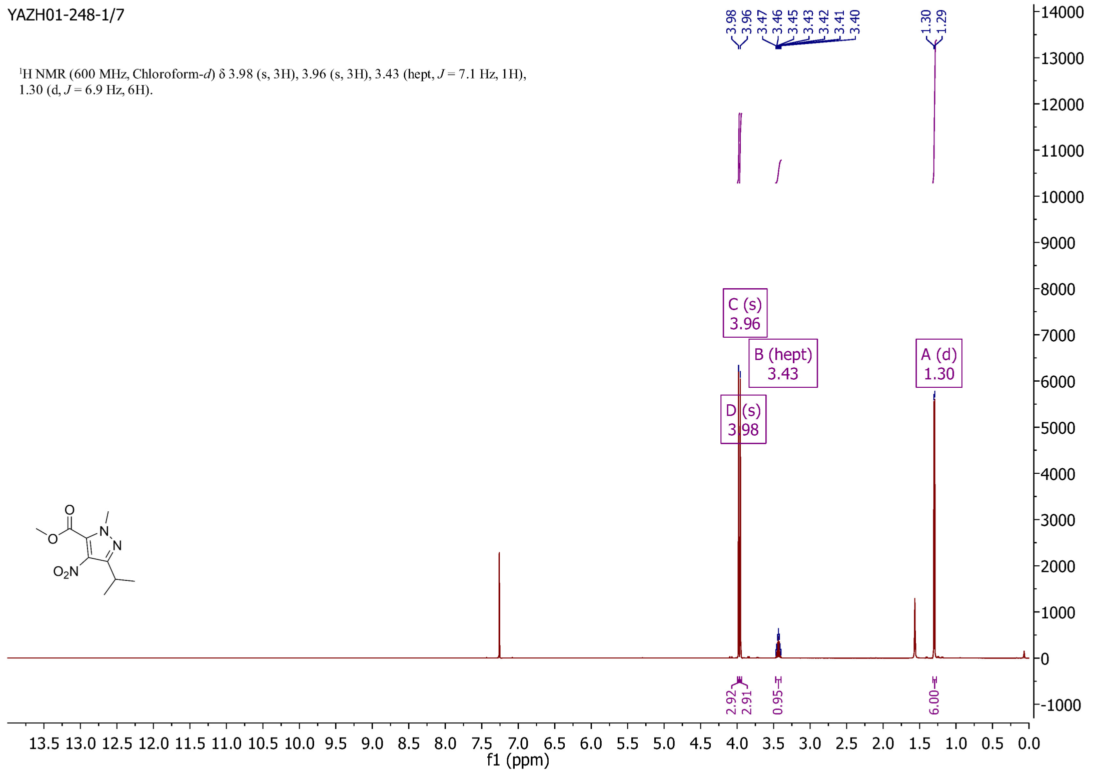

Figure S90. ^{1}H NMR spectrum of compound **34a**.

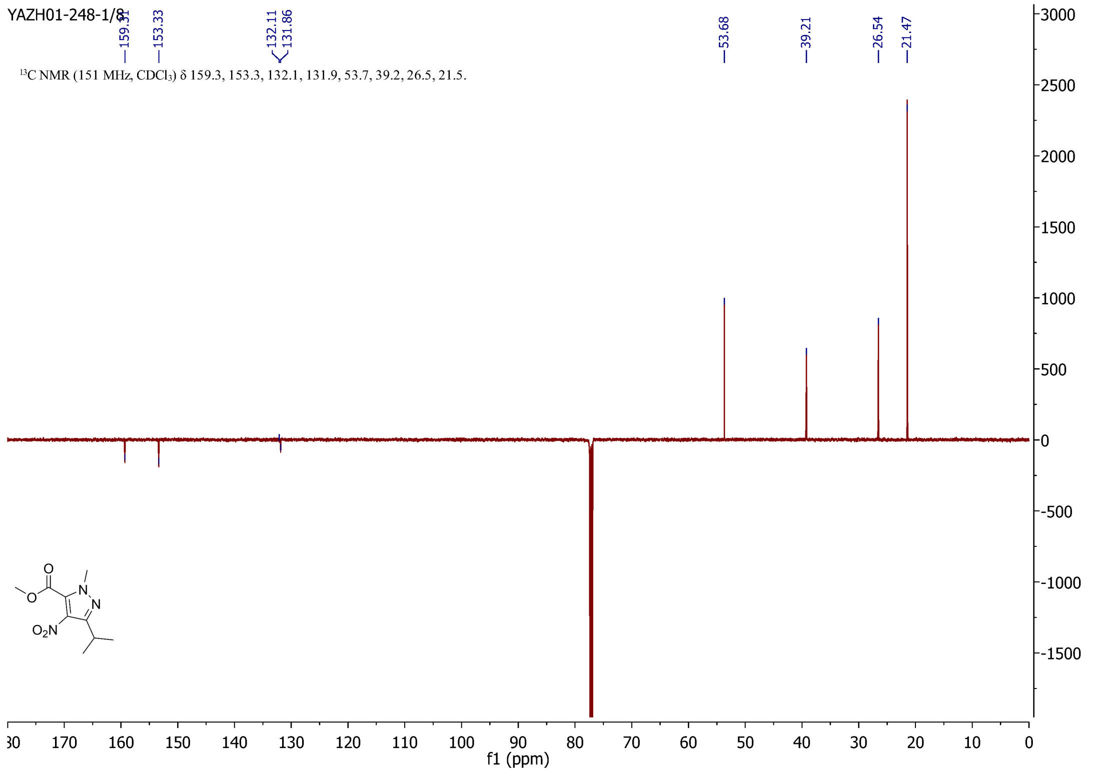

Figure S91. ^{13}C NMR spectrum of compound **34a**.

Acquired by	: Admin
Date Acquired	: 3/9/2018 2:38:23 PM
Sample Name	: YAZH01-248-2
Sample ID	:
Tray#	: 1
Vial#	: 14
Injection Volume	: 1
Data File	: C:\LabSolutions\Data\2018\2018-wk36\YAZH01-248-2.lcd
Background File	: blanco 03092018.lcd
Method File	: Method SCAN ACID standard MW50.lcm
Report Format	: DefaultLCMS.lcr
Tuning File	: C:\LabSolutions\Tuning\Tuning-ESI-pos-neg01072015a.lct
Processed by	: Admin
Modified Date	: 3/9/2018 2:53:35 PM

Chromatogram
YAZH01-248-2 C:\LabSolutions\Data\2018\2018-wk36\YAZH01-248-2.lcd

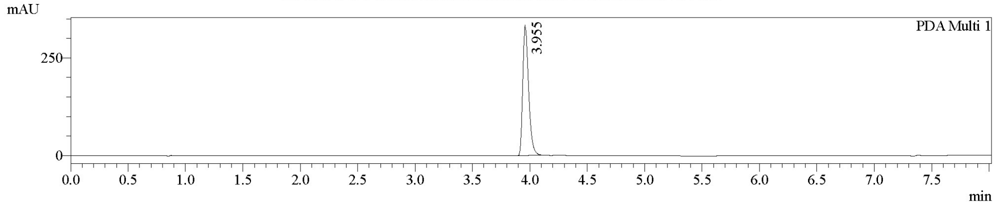

1 PDA Multi 1 / 254nm 4nm

Peak#	Name	Ret. Time	Area	Area %
1		3.955	1168339	100.000

MS Chromatogram
YAZH01-248-2 C:\LabSolutions\Data\2018\2018-wk36\YAZH01-248-2.lcd

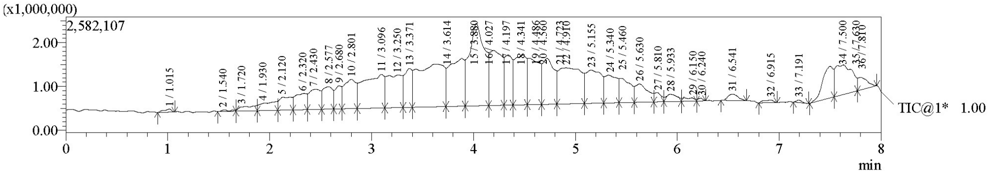

MS Spectrum Graph

#:1 Ret.Time:Averaged 4.020-4.040(Scan#:403-405)
Mass Peaks:17 Base Peak:228.05(435416) Polarity:Pos Segment1 - Event1

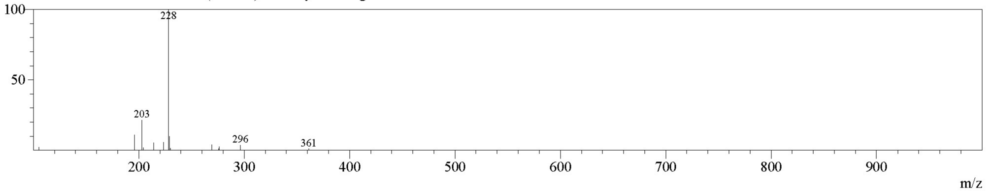

MS Spectrum Table

#:1 Ret.Time:
BG Mode:Calc 3.920<->4.150(393<->416)
Mass Peaks:17 Base Peak:228.05(435416) Polarity:Pos Segment1 - Event1

#	m/z	Abs.Inten.	Rel.Inten.	Charge	Polarity	Monoisotopic
1	59.05	11139	2.56			
2	83.05	30697	7.05			
3	105.05	10233	2.35			
4	196.00	47038	10.80			
5	203.00	93195	21.40			
6	204.20	8468	1.94			
7	214.10	23006	5.28			
8	223.50	18131	4.16			
9	223.60	24965	5.73			
10	228.05	435416	100.00			
11	229.10	43077	9.89			
12	230.05	7305	1.68			
13	269.15	17962	4.13			
14	275.50	4931	1.13			
15	276.10	10672	2.45			
16	296.10	15696	3.60			
17	361.10	4417	1.01			

Figure S92. LCMS spectrum of compound **34b**.

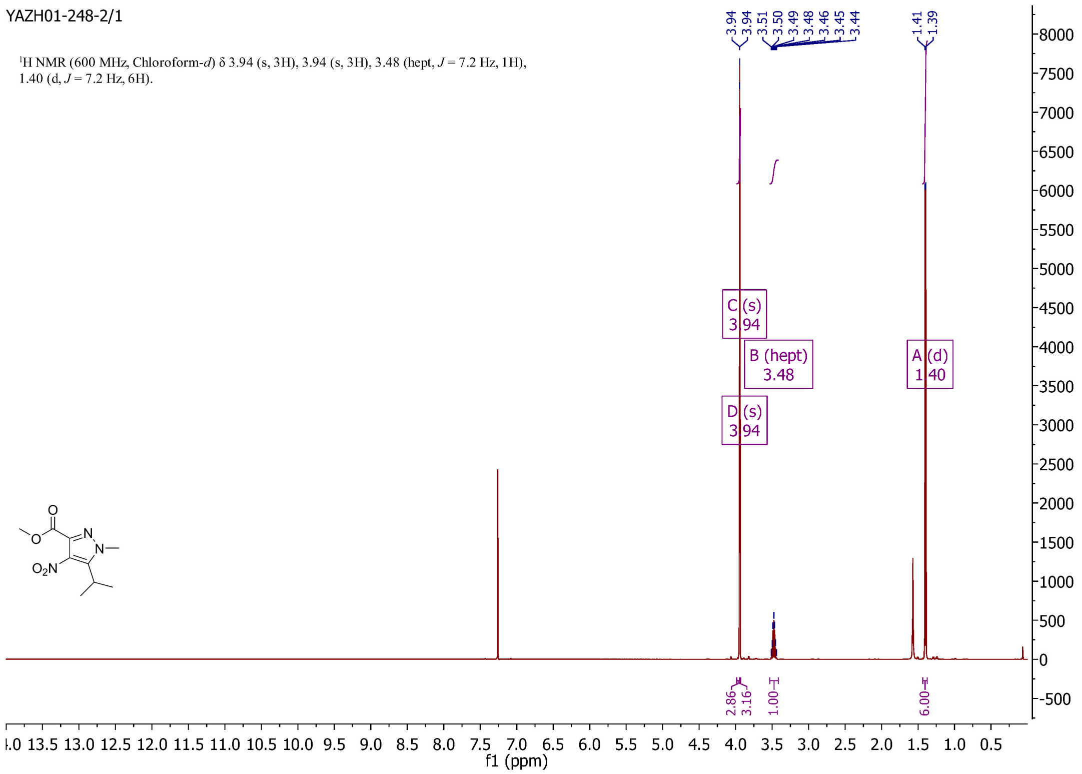

Figure S93. ^{1}H NMR spectrum of compound **34b**.

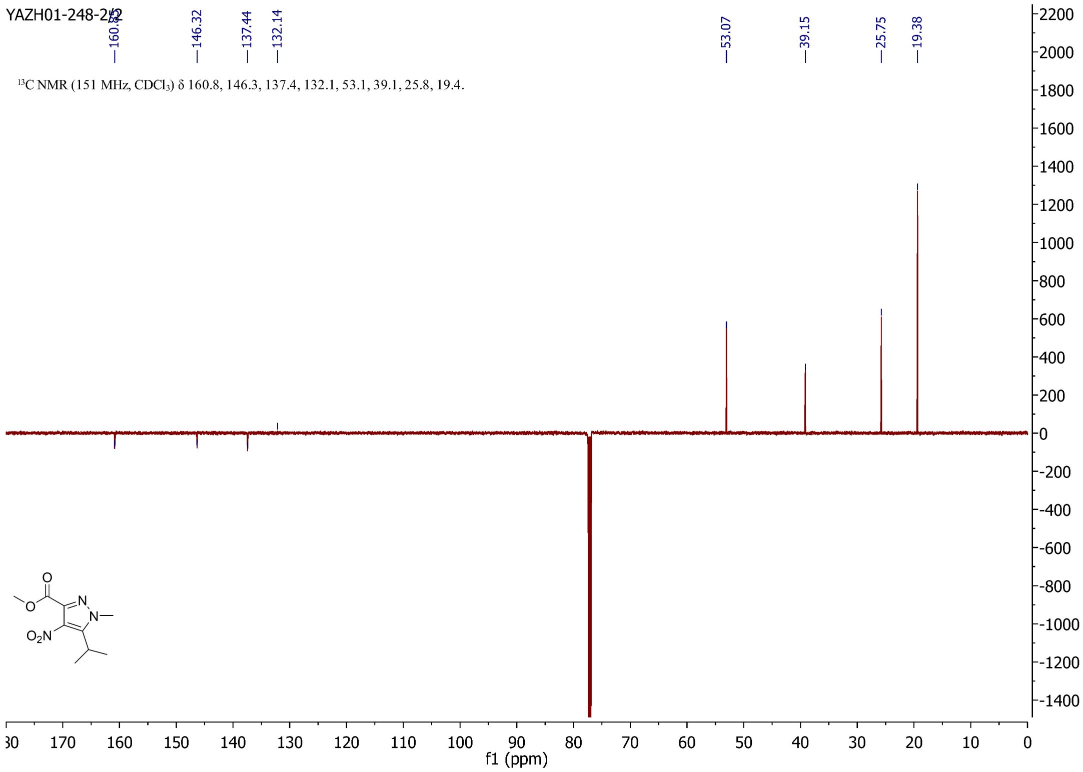

Figure S94. ^{13}C NMR spectrum of compound **34b**.

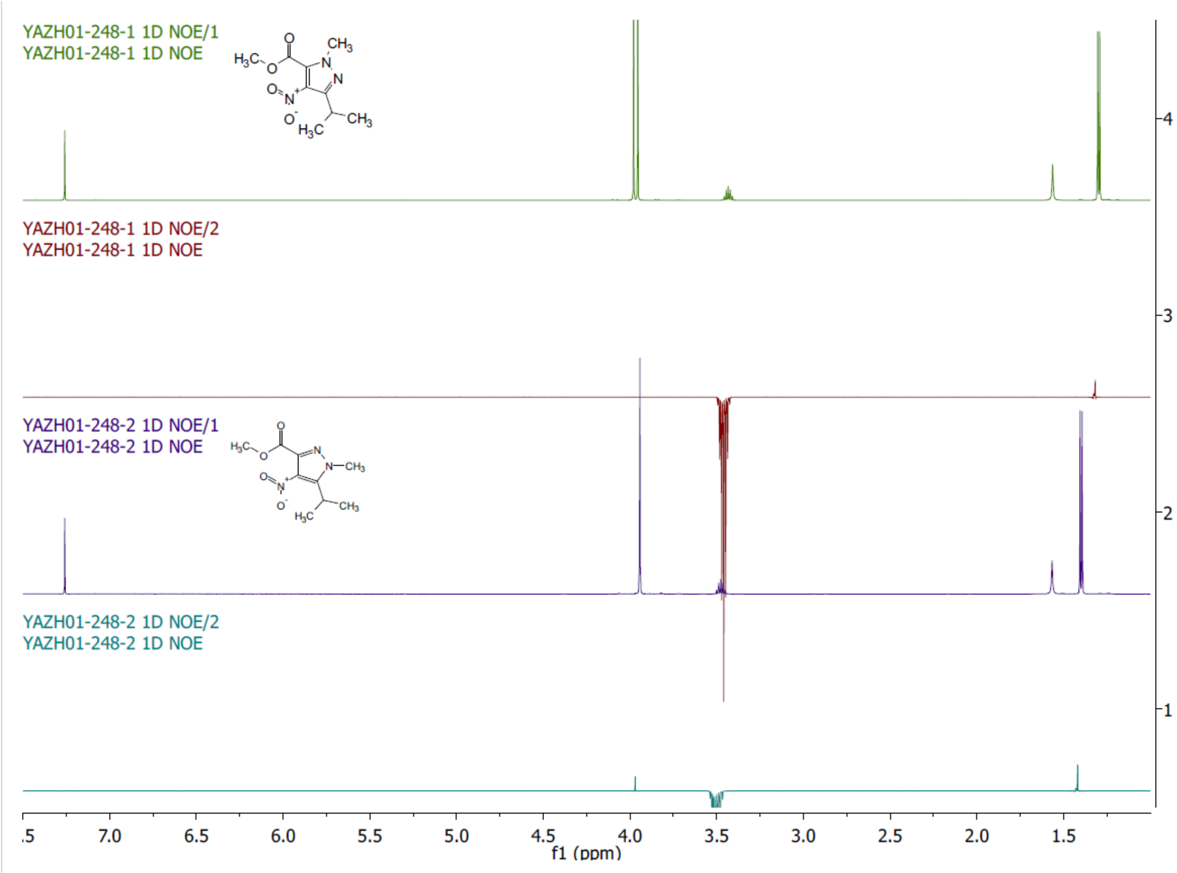

Figure S95. 1D NOESY spectrum of compound **34a**/**34b**.

Acquired by	: Admin
Date Acquired	: 13/9/2018 4:39:27 PM
Sample Name	: YAZH01-257
Sample ID	:
Tray#	: 1
Vial#	: 27
Injection Volume	: 1
Data File	: C:\LabSolutions\Data\2018\2018-wk37\YAZH01-257.lcd
Background File	: blanco 13092018.lcd
Method File	: Method SCAN ACID standard MW100.lcm
Report Format	: DefaultLCMS.lcr
Tuning File	: C:\LabSolutions\Tuning\Tuning-ESI-pos-neg01072015a.lct
Processed by	: Admin
Modified Date	: 13/9/2018 5:21:56 PM

Chromatogram
YAZH01-257 C:\LabSolutions\Data\2018\2018-wk37\YAZH01-257.lcd

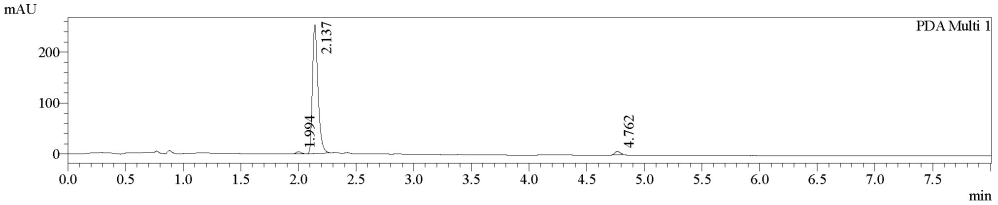

1 PDA Multi 1 / 254nm 4nm

Peak#	Name	Ret. Time	Area	Area %
1		1.994	10665	1.228
2		2.137	838126	96.509
3		4.762	19653	2.263

MS Chromatogram
YAZH01-257 C:\LabSolutions\Data\2018\2018-wk37\YAZH01-257.lcd

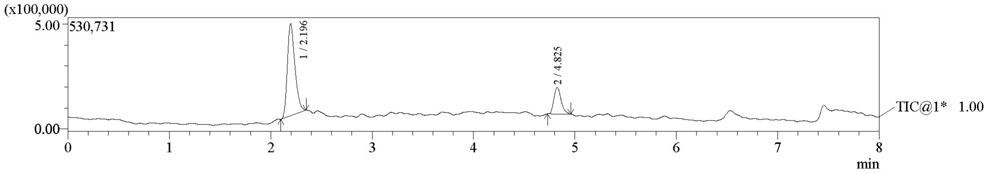

MS Spectrum Graph
#:1 Ret.Time:Averaged 2.190-2.210(Scan#:220-222)
Mass Peaks:7 Base Peak:183.05(315118) Polarity:Pos Segment1 - Event1

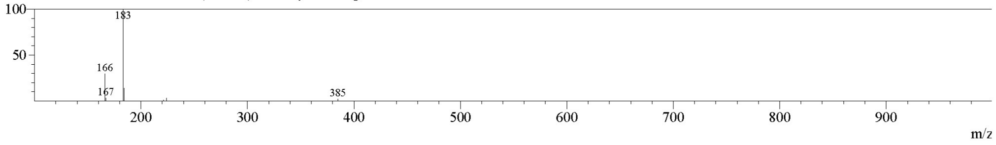

MS Spectrum Table
#:1 Ret.Time:
BG Mode:Calc 2.100<->2.350(211<->236)
Mass Peaks:7 Base Peak:183.05(315118) Polarity:Pos Segment1 - Event1

#	m/z	Abs.Inten.	Rel.Inten.	Charge	Polarity	Monoisotopic
1	166.00	92920	29.49			
2	167.10	9564	3.04			
3	183.05	315118	100.00			
4	184.05	44037	13.97			
5	221.10	3883	1.23			
6	224.05	9755	3.10			
7	385.15	6722	2.13			

Figure S96. LCMS spectrum of compound **36a**.

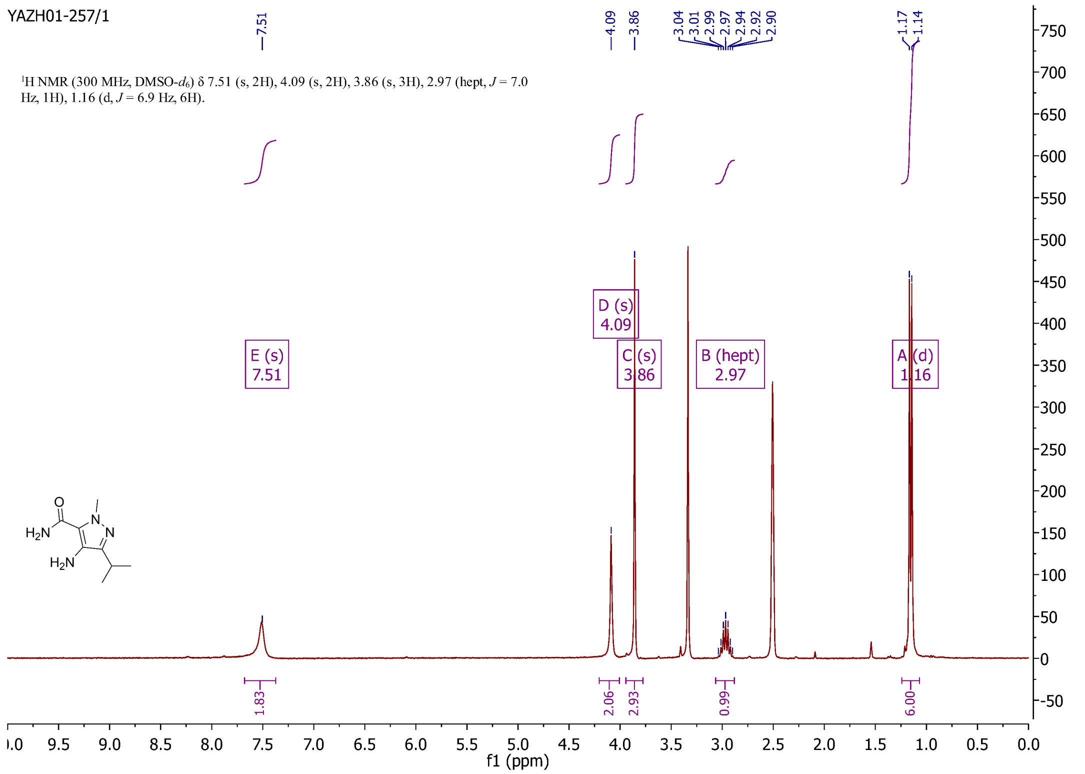

Figure S97. ^{1}H NMR spectrum of compound **36a**.

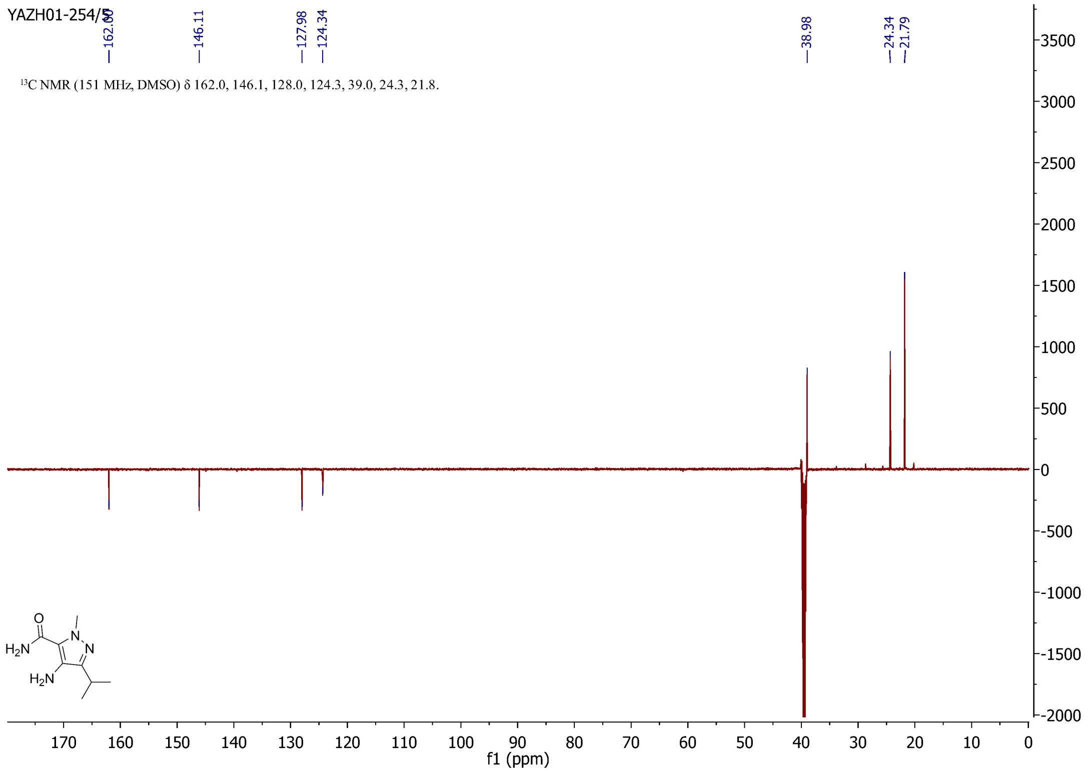

Figure S98. ^{13}C NMR spectrum of compound **36a**.

Acquired by	: Admin
Date Acquired	: 13/9/2018 4:48:05 PM
Sample Name	: YAZH01-258
Sample ID	:
Tray#	: 1
Vial#	: 28
Injection Volume	: 1
Data File	: C:\LabSolutions\Data\2018\2018-wk37\YAZH01-258.lcd
Background File	: blanco 13092018.lcd
Method File	: Method SCAN ACID standard MW100.lcm
Report Format	: DefaultLCMS.lcr
Tuning File	: C:\LabSolutions\Tuning\Tuning-ESI-pos-neg01072015a.lct
Processed by	: Admin
Modified Date	: 13/9/2018 5:22:57 PM

Chromatogram
YAZH01-258 C:\LabSolutions\Data\2018\2018-wk37\YAZH01-258.lcd

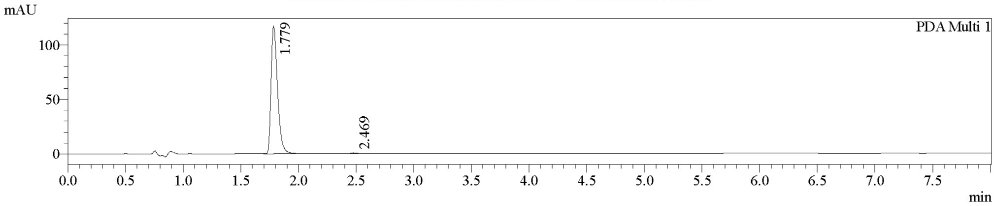

1 PDA Multi 1 / 254nm 4nm

Peak#	Name	Ret. Time	Area	Area %
1		1.779	453050	99.404
2		2.469	2715	0.596

MS Chromatogram
YAZH01-258 C:\LabSolutions\Data\2018\2018-wk37\YAZH01-258.lcd

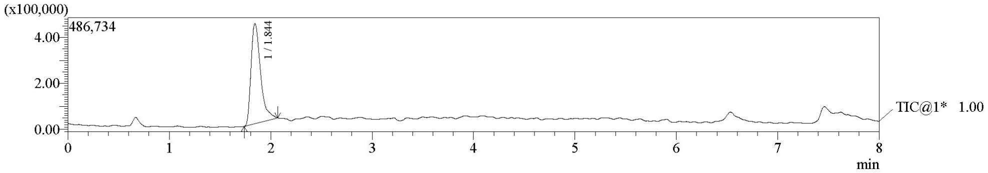

MS Spectrum Graph
#:1 Ret.Time:Averaged 1.830-1.850(Scan#:184-186)
Mass Peaks:10 Base Peak:183.05(279952) Polarity:Pos Segment1 - Event1

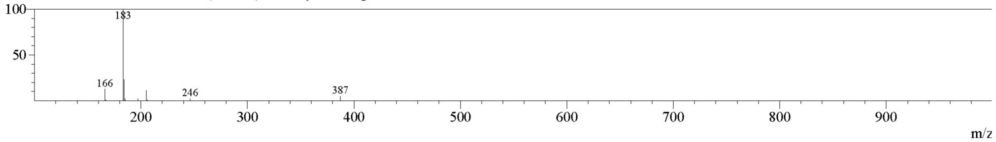

MS Spectrum Table
#:1 Ret.Time:
BG Mode:Calc 1.740<->2.070(175<->208)
Mass Peaks:10 Base Peak:183.05(279952) Polarity:Pos Segment1 - Event1

#	m/z	Abs.Inten.	Rel.Inten.	Charge	Polarity	Monoisotopic
1	166.10	35487	12.68			
2	167.00	3201	1.14			
3	183.05	279952	100.00			
4	184.05	65044	23.23			
5	185.05	5591	2.00			

#	m/z	Abs.Inten.	Rel.Inten.	Charge	Polarity	Monoisotopic
6	197.00	5190	1.85			
7	205.00	31743	11.34			
8	206.00	3173	1.13			
9	246.10	6568	2.35			
10	387.15	13120	4.69			

Figure S99. LCMS spectrum of compound **36b**.

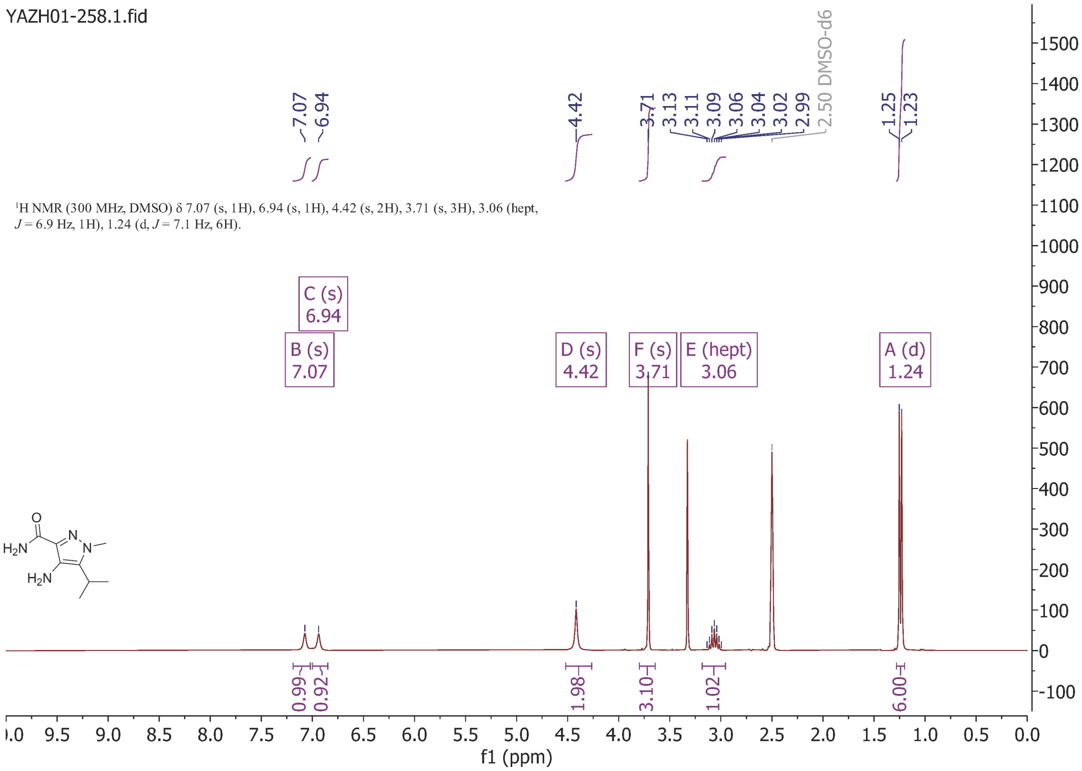

Figure S100. ^{1}H NMR spectrum of compound **36b**.

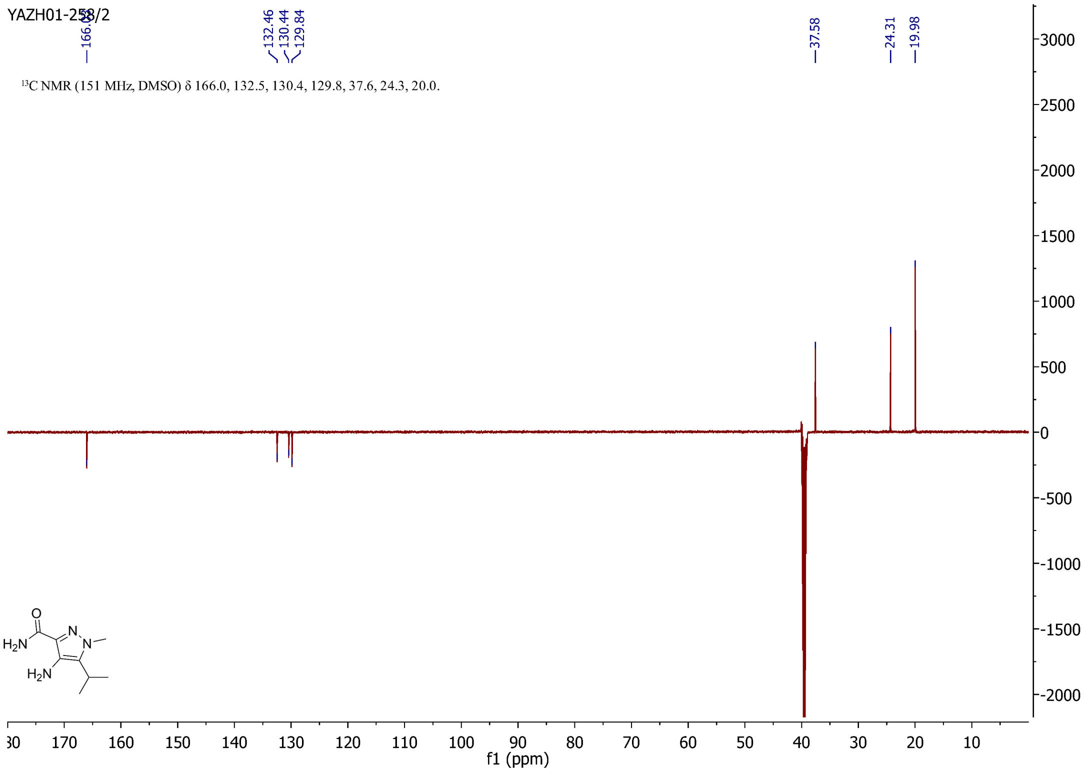

Figure S101. ^{13}C NMR spectrum of compound **36b**.

Acquired by	: Admin
Date Acquired	: 10/4/2019 11:56:17 AM
Sample Name	: YAZH_33
Sample ID	:
Tray#	: 1
Vial#	: 48
Injection Volume	: 3
Data File	: C:\LabSolutions\Data\2019\2019-wk15\YAZH_33.lcd
Background File	: blanco 10042019.lcd
Method File	: Method SCAN ACID standard.lcm
Report Format	: DefaultLCMS.lcr
Tuning File	: C:\LabSolutions\Tuning\Tuning-ESI-pos-neg01072015a.lct
Processed by	: Admin
Modified Date	: 10/4/2019 12:37:23 PM

PDA Graph

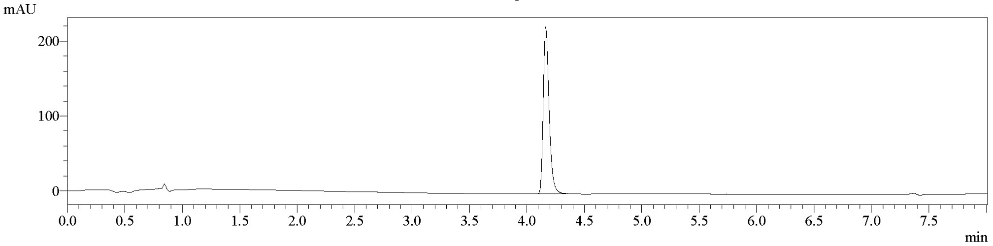

PeakTable

PDA Ch1 254nm 4nm

Peak#	Name	Ret. Time	Area	Area %
1		4.159	787857	100.000

MS Chromatogram

YAZH_33 C:\LabSolutions\Data\2019\2019-wk15\YAZH_33.lcd

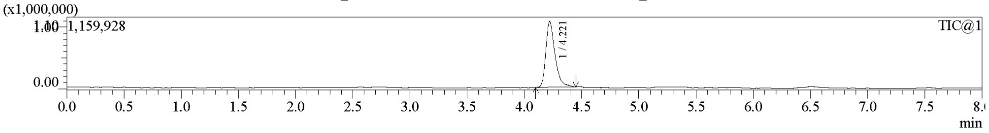

MS Spectrum Graph

#:1 Ret.Time:Averaged 4.210-4.230(Scan#:422-424)
BG Mode:Calc 4.100<->4.450(411<->446)
Mass Peaks:6 Base Peak:283.10(901404) Polarity:Pos Segment1 - Event1

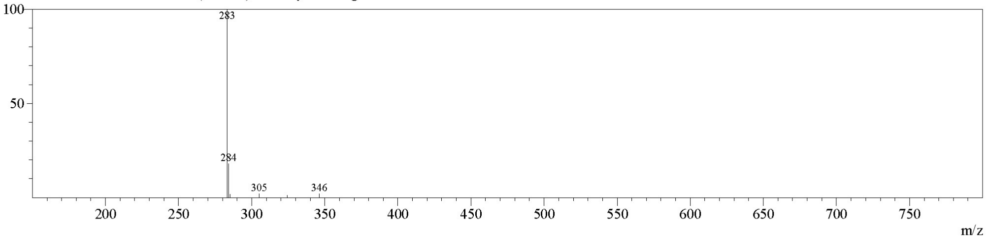

MS Spectrum Table

#:1 Ret.Time:
BG Mode:Calc 4.100<->4.450(411<->446)
Mass Peaks:6 Base Peak:283.10(901404) Polarity:Pos Segment1 - Event1

#	m/z	Abs.Inten.	Rel.Inten.	Charge	Polarity	Monoisotopic	#	m/z	Abs.Inten.	Rel.Inten.	Charge	Polarity	Monoisotopic
1	283.10	901404	100.00				4	305.15	17493	1.94			
2	284.10	161921	17.96				5	324.15	11299	1.25			
3	285.20	14828	1.64				6	346.15	18518	2.05			

Figure S102. LCMS spectrum of compound **37a**.

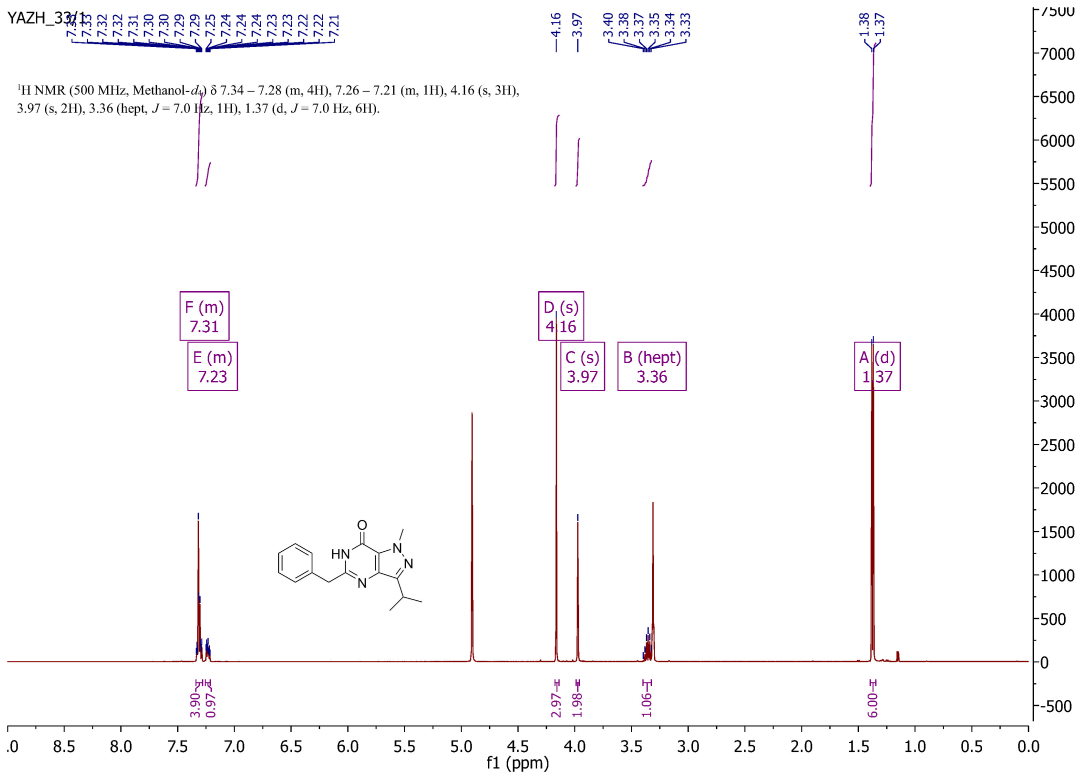

Figure S103. ^{1}H NMR spectrum of compound **37a**.

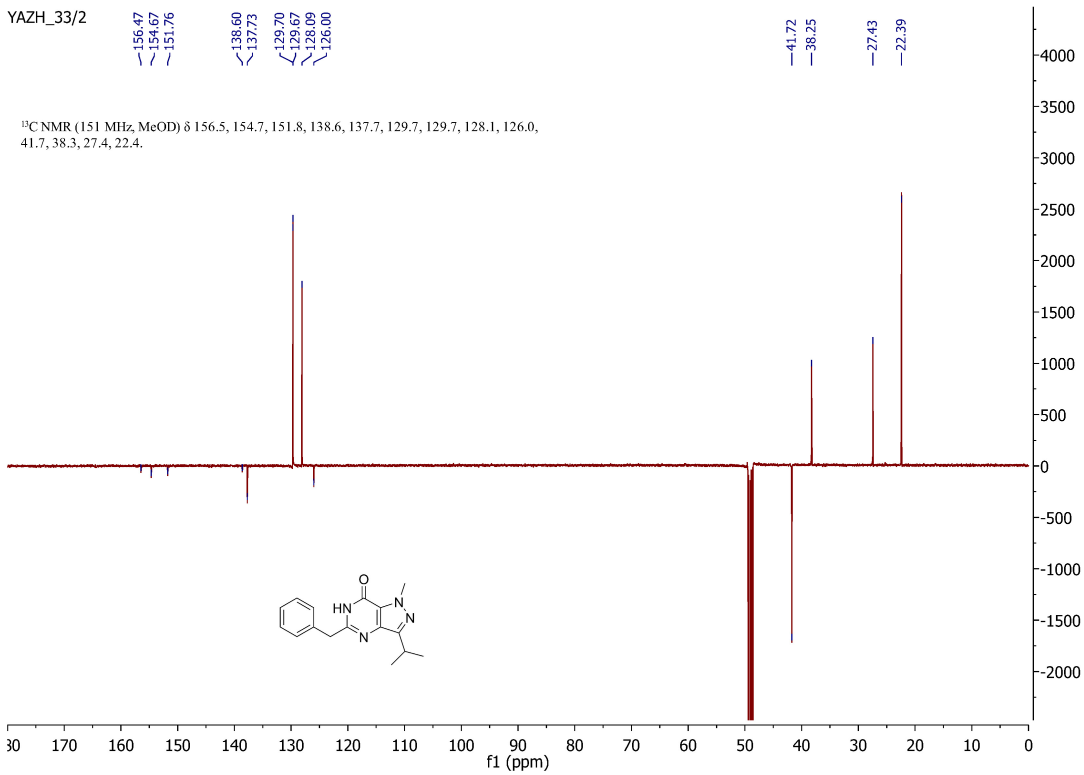

Figure S104. ^{13}C NMR spectrum of compound **37a**.

Acquired by	: Admin
Date Acquired	: 11/4/2019 12:33:10 PM
Sample Name	: YAZH_31
Sample ID	:
Tray#	: 1
Vial#	: 16
Injection Volume	: 3
Data File	: C:\LabSolutions\Data\2019\2019-wk15\YAZH_31.lcd
Background File	: blanco 11042019.lcd
Method File	: Method SCAN ACID standard.lcm
Report Format	: DefaultLCMS.lcr
Tuning File	: C:\LabSolutions\Tuning\Tuning-ESI-pos-neg01072015a.lct
Processed by	: Admin
Modified Date	: 11/4/2019 2:02:29 PM

PDA Graph

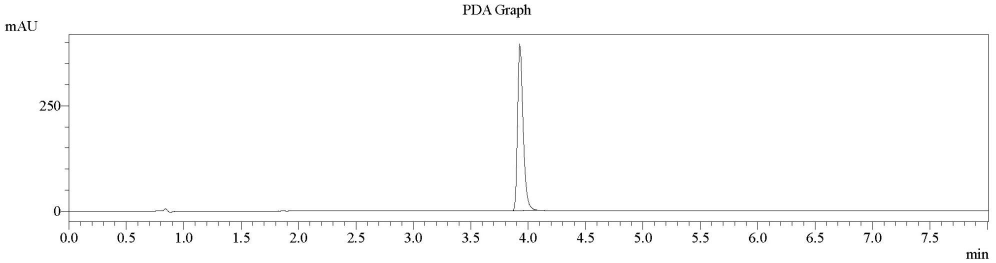

PeakTable

PDA Ch1 254nm 4nm

Peak#	Name	Ret. Time	Area	Area %
1		3.923	1338326	100.000

MS Chromatogram
YAZH_31 C:\LabSolutions\Data\2019\2019-wk15\YAZH_31.lcd

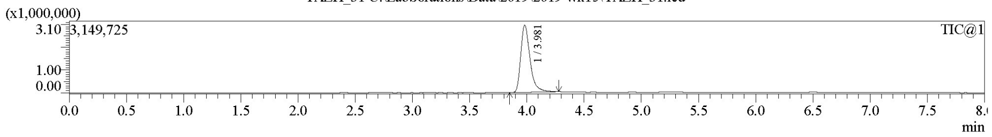

MS Spectrum Graph

#:1 Ret.Time:Averaged 3.970-3.990(Scan#:398-400)
BG Mode:Calc 3.850<->4.280(386<->429)
Mass Peaks:8 Base Peak:283.10(2112647) Polarity:Pos Segment1 - Event1

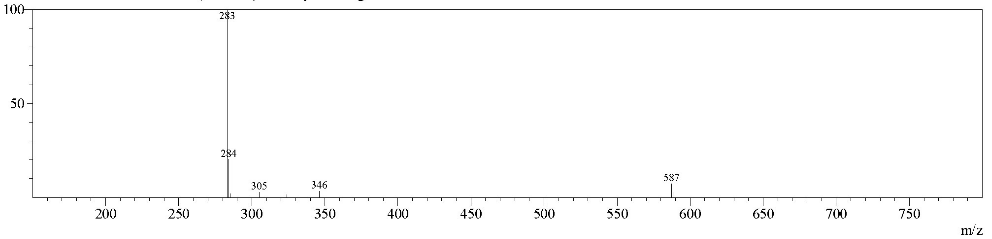

MS Spectrum Table

#:1 Ret.Time:
BG Mode:Calc 3.850<->4.280(386<->429)
Mass Peaks:8 Base Peak:283.10(2112647) Polarity:Pos Segment1 - Event1

#	m/z	Abs.Inten.	Rel.Inten.	Charge	Polarity	Monoisotopic
1	283.10	2112647	100.00			
2	284.15	426066	20.17			
3	285.15	40415	1.91			
4	305.05	56694	2.68			
5	324.05	27628	1.31			
6	346.15	68700	3.25			
7	587.30	154265	7.30			
8	588.30	57906	2.74			

Figure S105. LCMS spectrum of compound **37b**.

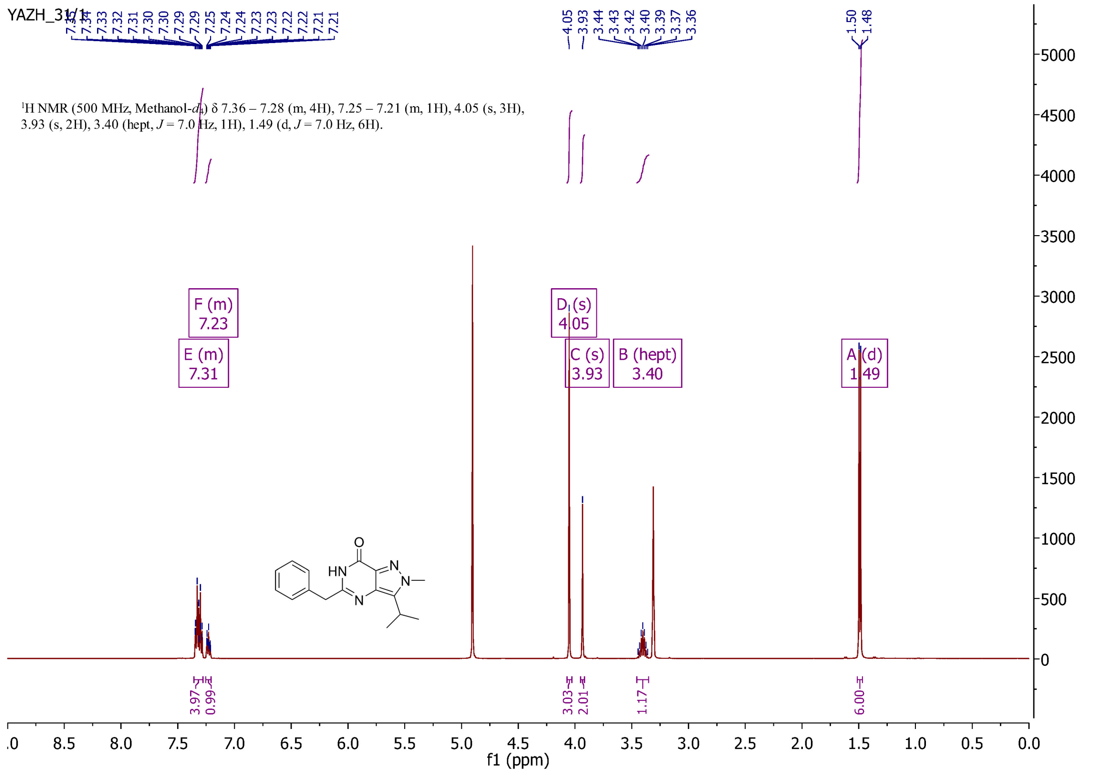

Figure S106. ^{1}H NMR spectrum of compound **37b**.

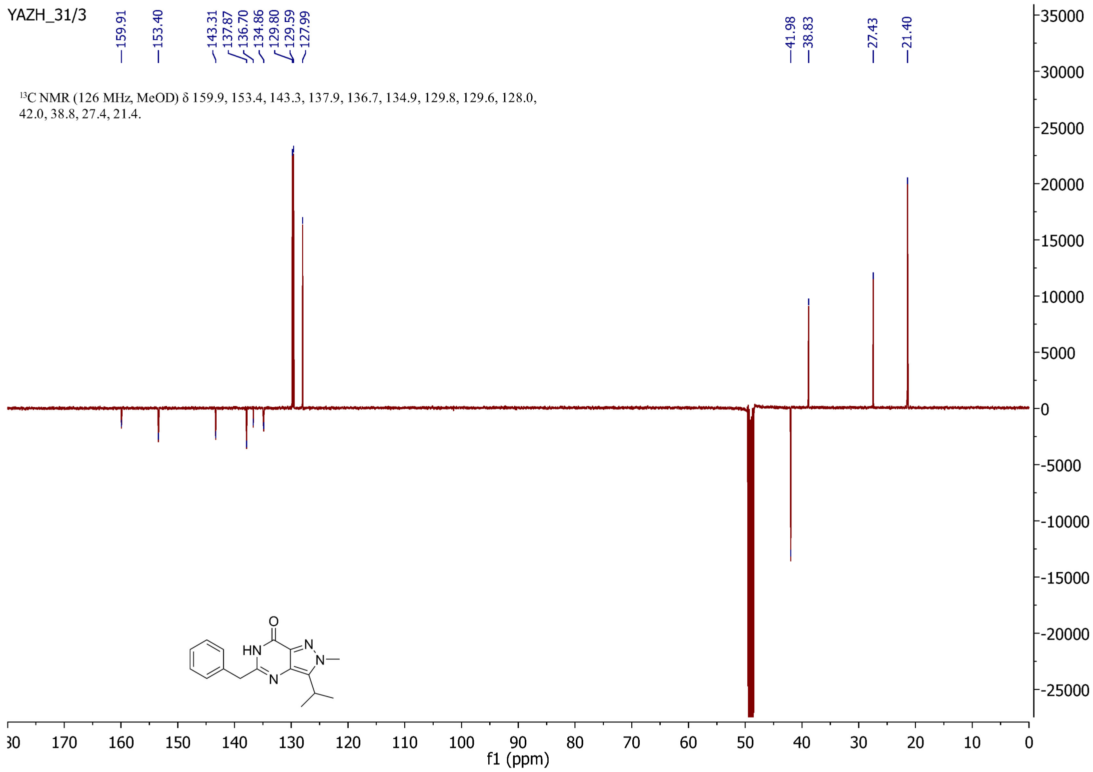

Figure S107. ^{13}C NMR spectrum of compound **37b**.

Acquired by	: Admin
Date Acquired	: 25/3/2019 11:22:17 AM
Sample Name	: YAZH_34
Sample ID	:
Tray#	: 1
Vial#	: 8
Injection Volume	: 3
Data File	: C:\LabSolutions\Data\2019\2019-wk12\YAZH_34.lcd
Background File	: blanco 25032019.lcd
Method File	: Method SCAN ACID standard.lcm
Report Format	: DefaultLCMS.lcr
Tuning File	: C:\LabSolutions\Tuning\Tuning-ESI-pos-neg01072015a.lct
Processed by	: Admin
Modified Date	: 18/6/2019 10:18:35 AM

PDA Graph

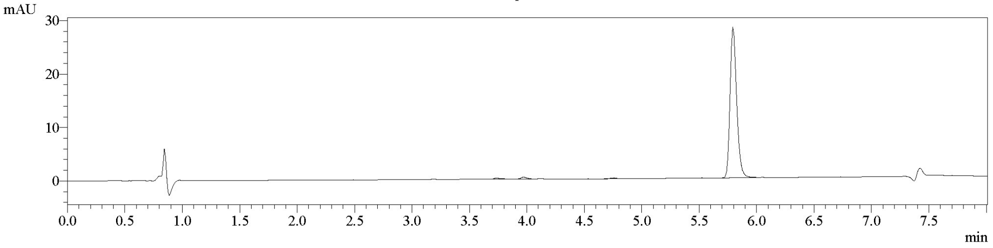

PeakTable

PDA Ch1 254nm 4nm

Peak#	Name	Ret. Time	Area	Area %
1		3.729	960	0.837
2		3.965	1168	1.018
3		4.752	874	0.762
4		5.789	111803	97.384

MS Chromatogram

YAZH_34 C:\LabSolutions\Data\2019\2019-wk11-20\2019-wk13\YAZH_34.lcd

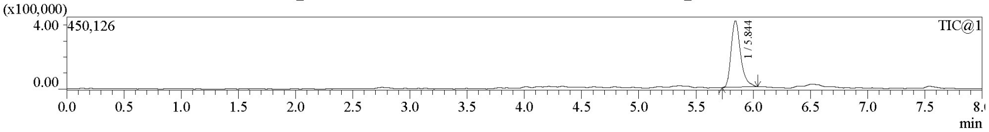

MS Spectrum Graph

#:1 Ret.Time:Averaged 5.830-5.850(Scan#:584-586)
BG Mode:Calc 5.730<->6.040(574<->605)
Mass Peaks:4 Base Peak:327.15(346300) Polarity:Pos Segment1 - Event1

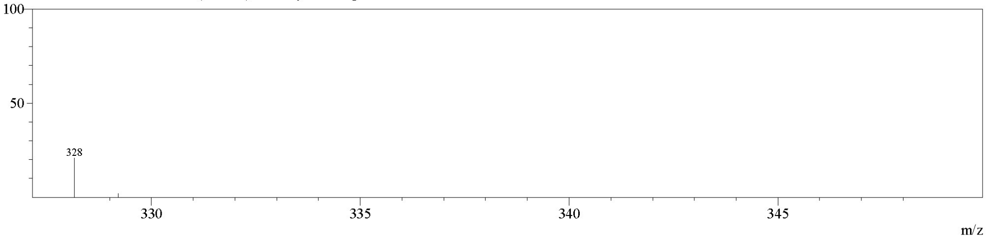

MS Spectrum Table

#:1 Ret.Time:
BG Mode:Calc 5.730<->6.040(574<->605)
Mass Peaks:4 Base Peak:327.15(346300) Polarity:Pos Segment1 - Event1

#	m/z	Abs.Inten.	Rel.Inten.	Charge	Polarity	Monoisotopic
1	327.15	346300	100.00			
2	328.15	71718	20.71			
3	329.20	6716	1.94			
4	349.90	3808	1.10			

Figure S108. LCMS spectrum of compound **38a**.

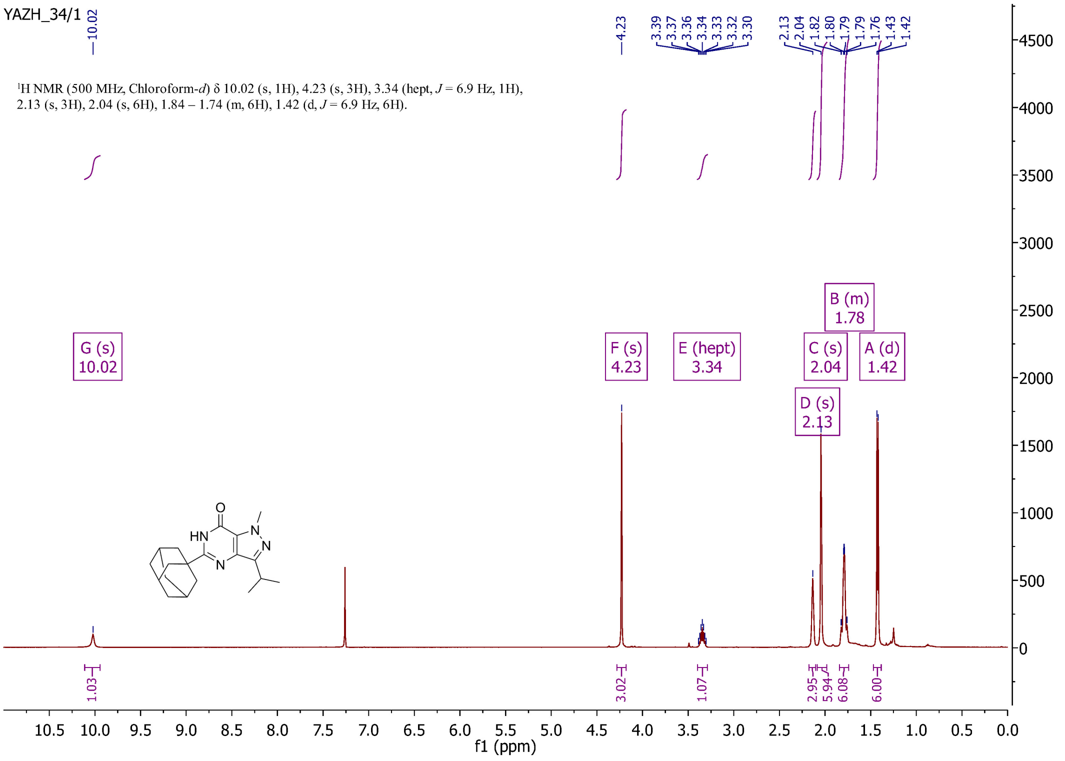

Figure S109. ^{1}H NMR spectrum of compound **38a**.

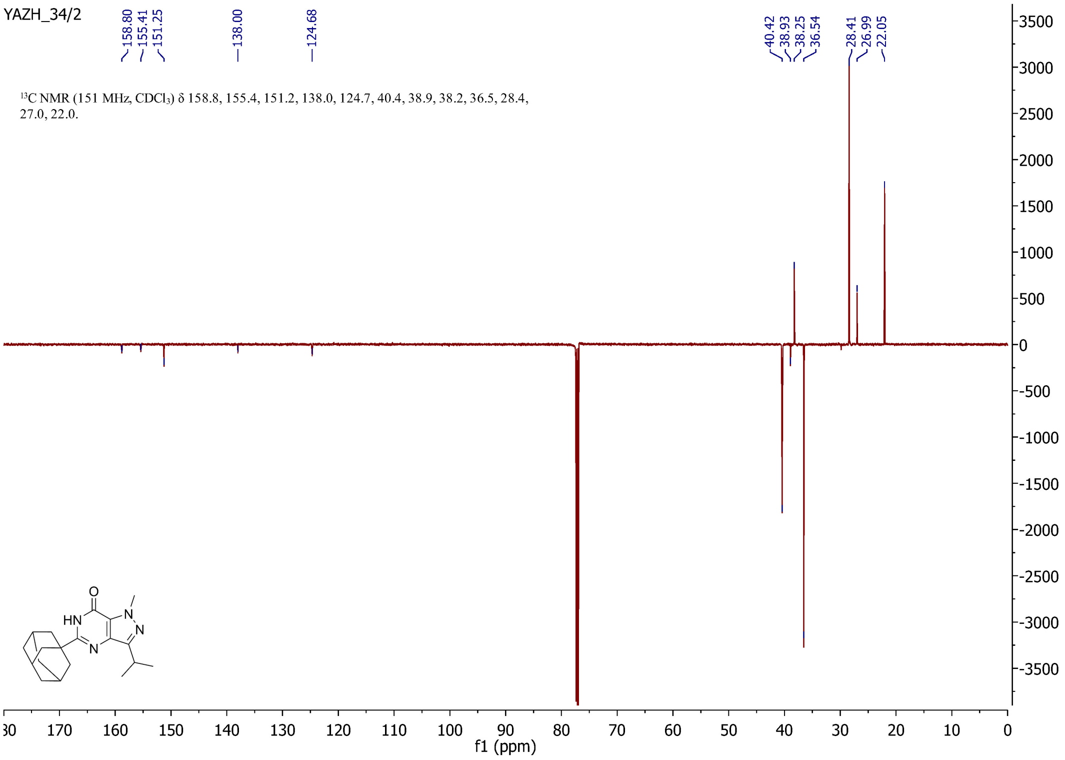

Figure S110. ^{13}C NMR spectrum of compound **38a**.

Acquired by	: Admin
Date Acquired	: 1/4/2019 10:21:09 AM
Sample Name	: YAZH_32
Sample ID	:
Tray#	: 1
Vial#	: 5
Injection Volume	: 3
Data File	: C:\LabSolutions\Data\2019\2019-wk14\YAZH_32.lcd
Background File	: blanco 01042019.lcd
Method File	: Method SCAN ACID standard.lcm
Report Format	: DefaultLCMS.lcr
Tuning File	: C:\LabSolutions\Tuning\Tuning-ESI-pos-neg01072015a.lct
Processed by	: Admin
Modified Date	: 1/4/2019 10:48:49 AM

PDA Graph

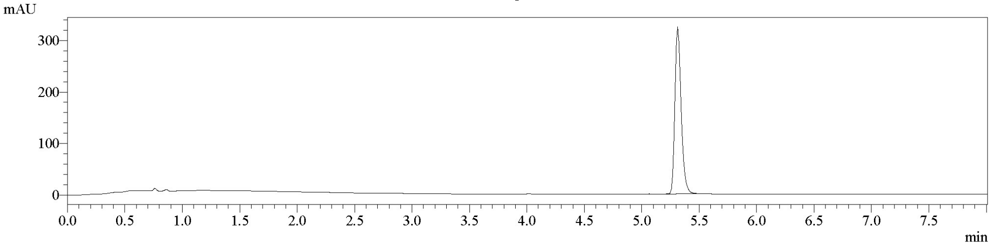

PeakTable

PDA Ch1 254nm 4nm

Peak#	Name	Ret. Time	Area	Area %
1		5.307	1228769	100.000

MS Chromatogram
YAZH_32 C:\LabSolutions\Data\2019\2019-wk14\YAZH_32.lcd

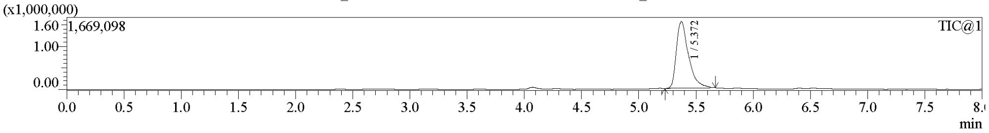

MS Spectrum Graph

#:1 Ret.Time:Averaged 5.360-5.380(Scan#:537-539)
BG Mode:Calc 5.230<->5.670(524<->568)
Mass Peaks:9 Base Peak:327.20(1028791) Polarity:Pos Segment1 - Event1

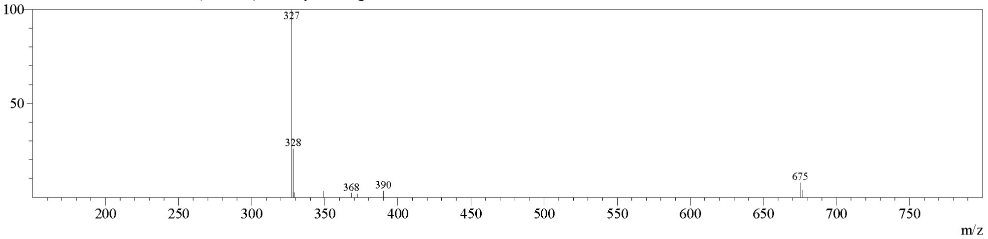

MS Spectrum Table

#:1 Ret.Time:
BG Mode:Calc 5.230<->5.670(524<->568)
Mass Peaks:9 Base Peak:327.20(1028791) Polarity:Pos Segment1 - Event1

#	m/z	Abs.Inten.	Rel.Inten.	Charge	Polarity	Monoisotopic	#	m/z	Abs.Inten.	Rel.Inten.	Charge	Polarity	Monoisotopic
1	327.20	1028791	100.00				6	372.30	16481	1.60			
2	328.20	265664	25.82				7	390.15	34662	3.37			
3	329.15	26096	2.54				8	675.40	80149	7.79			
4	349.10	32693	3.18				9	676.45	37982	3.69			
5	368.15	21306	2.07										

Figure S111. LCMS spectrum of compound **38b**.

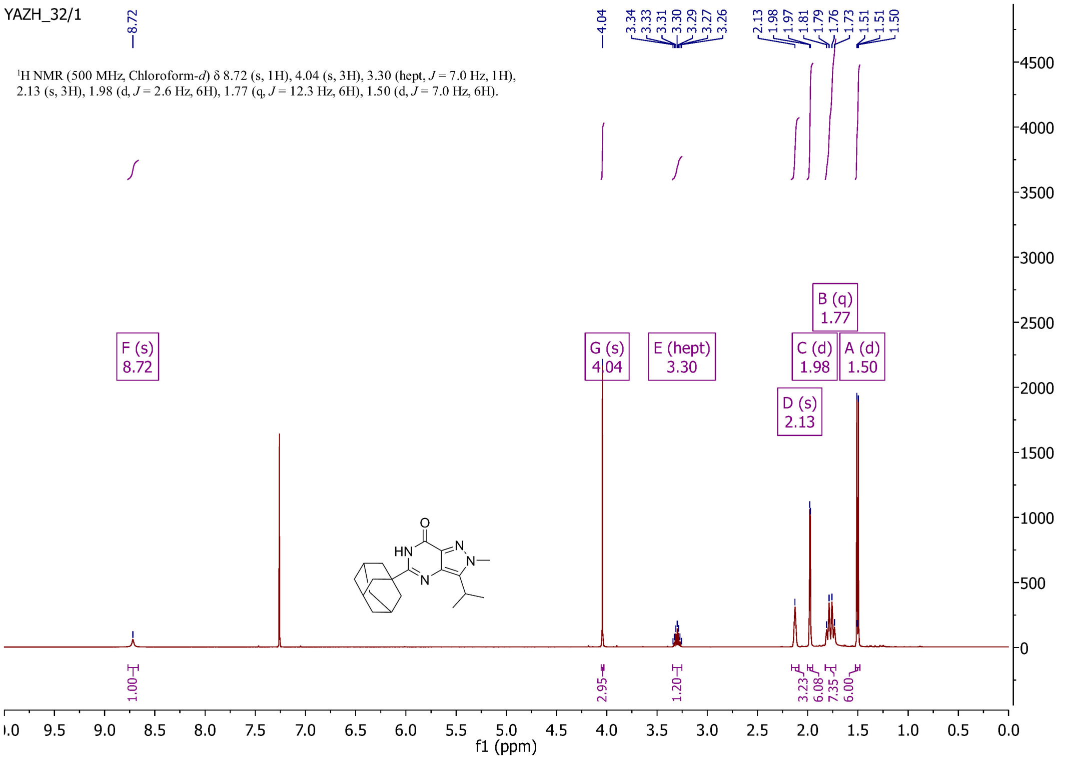

Figure S112. ^{1}H NMR spectrum of compound **38b**.

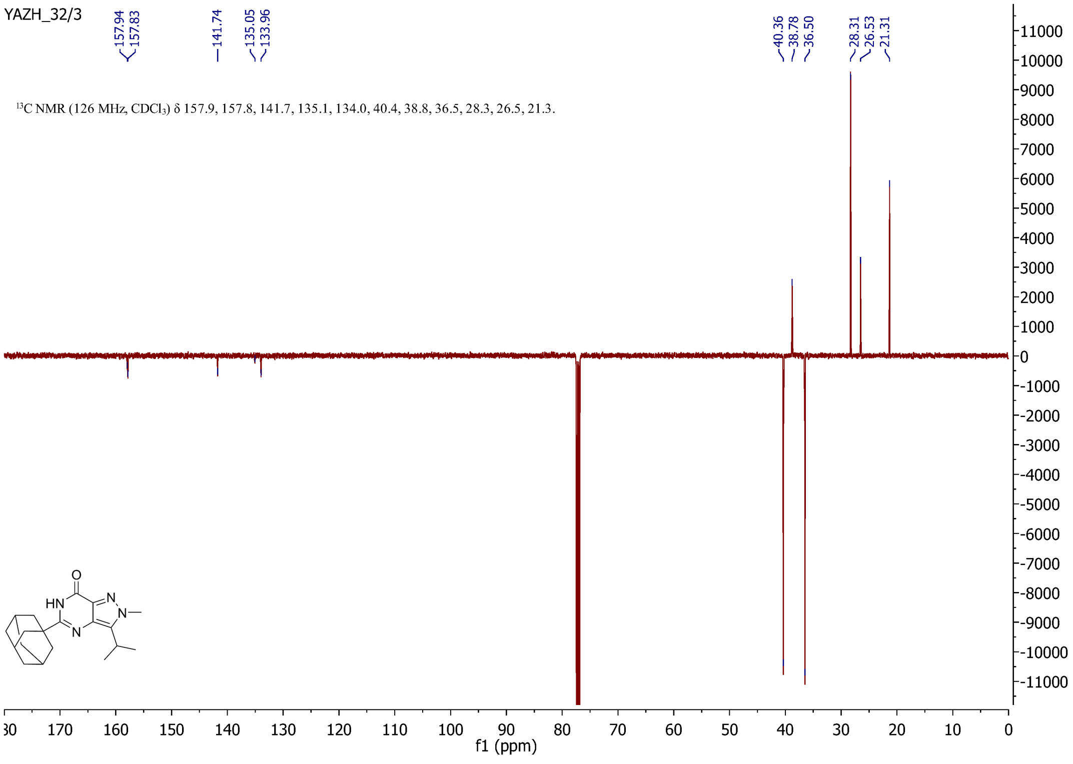

Figure S113. ^{13}C NMR spectrum of compound **38b**.

Acquired by	: Admin
Date Acquired	: 16/7/2018 10:51:14 AM
Sample Name	: LABA-022
Sample ID	:
Tray#	: 1
Vial#	: 9
Injection Volume	: 1
Data File	: C:\LabSolutions\Data\2018\2018-wk29\LABA-022.lcd
Background File	: blanco 16072018.lcd
Method File	: Method SCAN ACID standard neg.lcm
Report Format	: DefaultLCMS.lcr
Tuning File	: C:\LabSolutions\Tuning\Tuning-ESI-pos-neg01072015a.lct
Processed by	: Admin
Modified Date	: 16/7/2018 11:08:48 AM

Chromatogram
LABA-022 C:\LabSolutions\Data\2018\2018-wk29\LABA-022.lcd

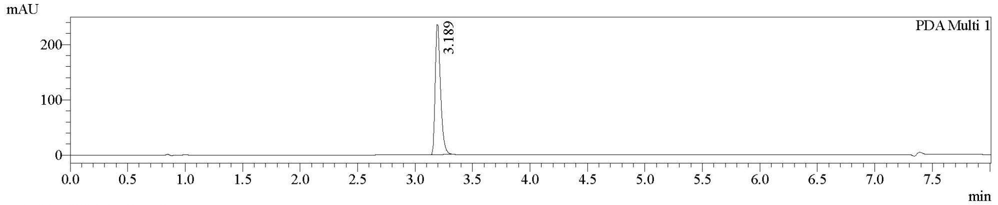

1 PDA Multi 1 / 254nm 4nm

Peak#	Name	Ret. Time	Area	Area %
1		3.189	765106	100.000

MS Chromatogram
LABA-022 C:\LabSolutions\Data\2018\2018-wk29\LABA-022.lcd

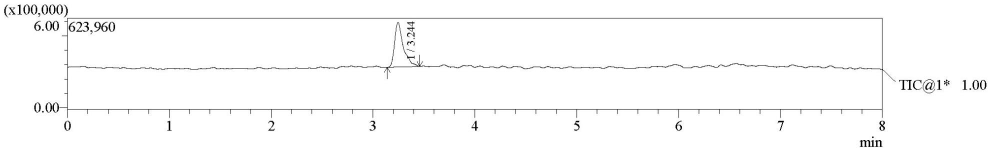

MS Spectrum Graph

#:1 Ret.Time:Averaged 3.230-3.250(Scan#:324-326)
Mass Peaks:24 Base Peak:179.00(141779) Polarity:Neg Segment1 - Event1

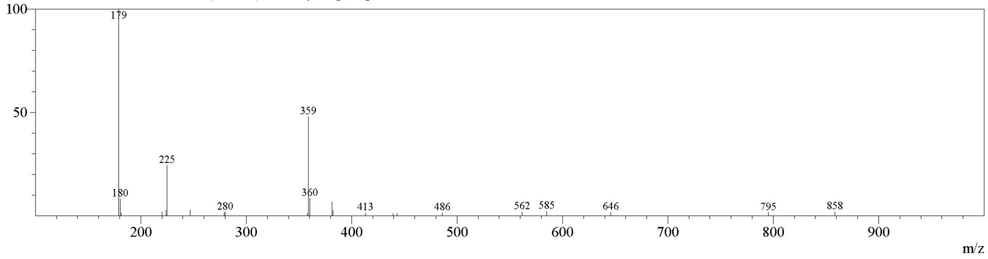

MS Spectrum Table

#:1 Ret.Time:
BG Mode:Calc 3.140<->3.460(315<->347)
Mass Peaks:24 Base Peak:179.00(141779) Polarity:Neg Segment1 - Event1

#	m/z	Abs.Inten.	Rel.Inten.	Charge	Polarity	Monoisotopic
1	179.00	141779	100.00			
2	180.25	11417	8.05			
3	181.20	1755	1.24			
4	220.15	2520	1.78			
5	224.15	3579	2.52			
6	225.05	34564	24.38			
7	247.05	4057	2.86			
8	279.10	2230	1.57			
9	280.00	2476	1.75			
10	358.10	1454	1.03			
11	359.15	67854	47.86			
12	360.15	11941	8.42			
13	381.25	9413	6.64			
14	382.20	3611	2.55			
15	413.10	2066	1.46			
16	439.15	1523	1.07			
17	443.25	1420	1.00			
18	486.00	1824	1.29			
19	561.95	2607	1.84			
20	585.05	2845	2.01			
21	645.70	2233	1.57			
22	795.40	2152	1.52			
23	858.30	2564	1.81			
24	1051.00	2181	1.54			

Figure S114. LCMS spectrum of compound **42b**.

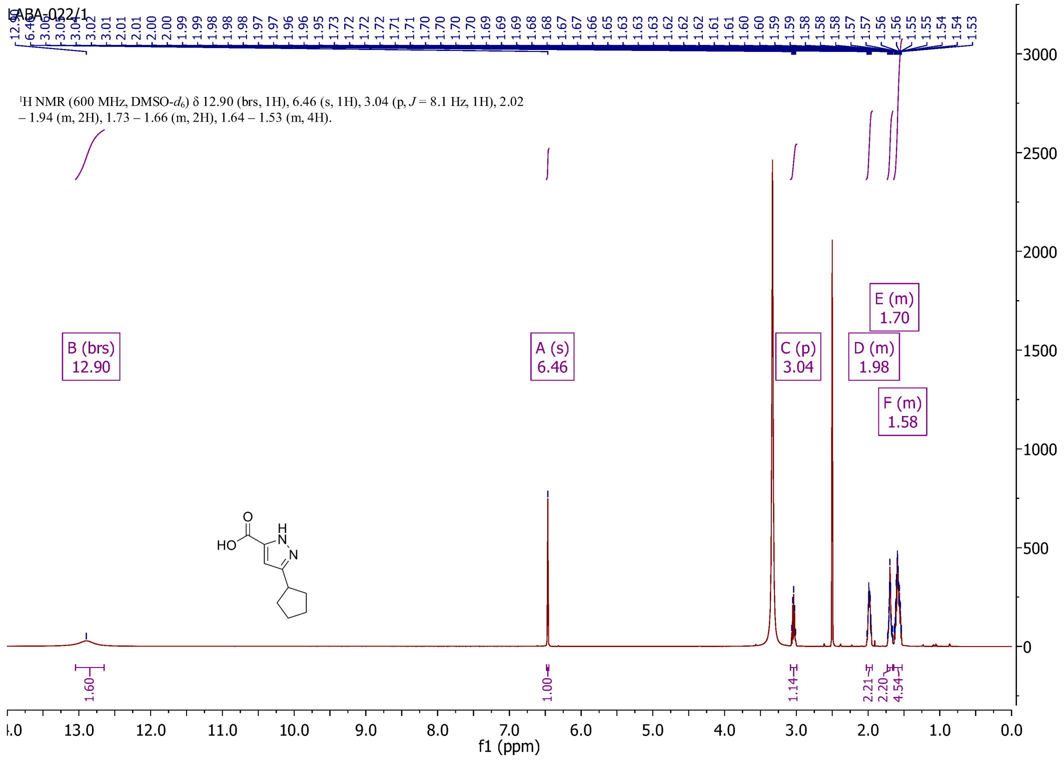

Figure S115. ^{1}H NMR spectrum of compound **42b**.

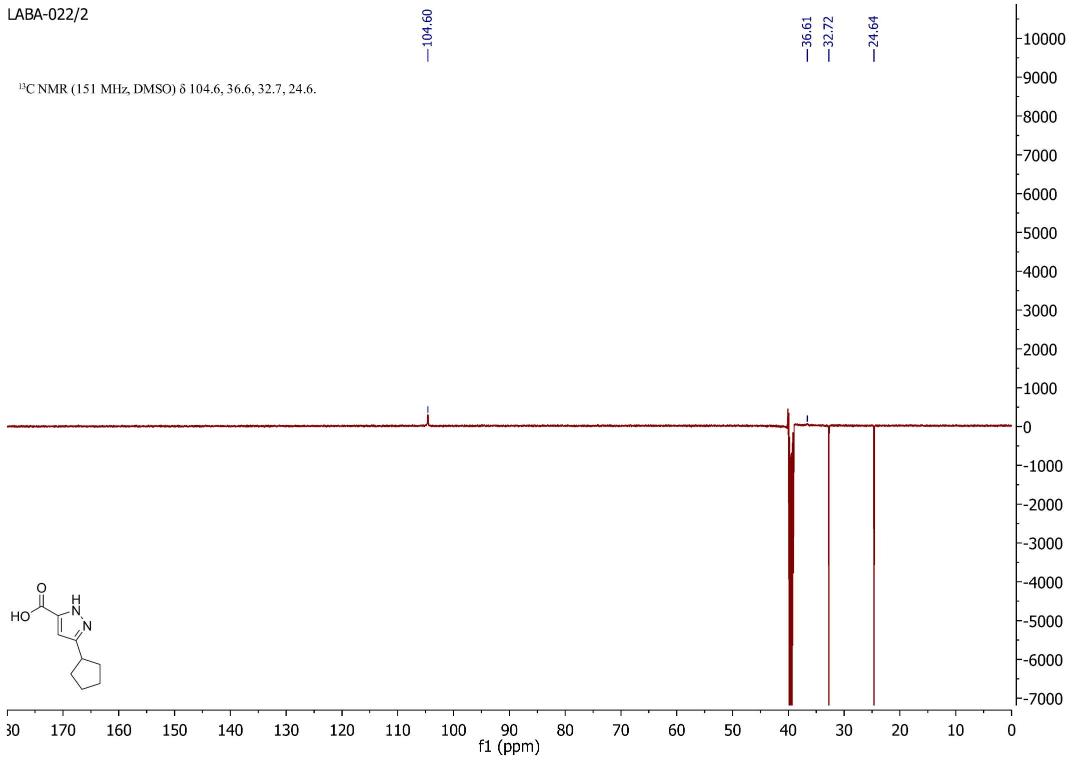

Figure S116. ^{13}C NMR spectrum of compound **42b**.

Acquired by	: Admin
Date Acquired	: 8/8/2017 12:43:48 PM
Sample Name	: YAZH01-126-1
Sample ID	:
Tray#	: 1
Vial#	: 2
Injection Volume	: 10
Data File	: C:\LabSolutions\Data\2017\2017-wk32\YAZH01-126-1.lcd
Background File	: blanco08082017.lcd
Method File	: Method SCAN ACID standard neg.lcm
Report Format	: DefaultLCMS.lcr
Tuning File	: C:\LabSolutions\Tuning\Tuning-ESI-pos-neg01072015a.lct
Processed by	: Admin
Modified Date	: 8/9/2017 3:58:23 PM

Chromatogram
YAZH01-126-1 C:\LabSolutions\Data\2017\2017-wk32\YAZH01-126-1.lcd

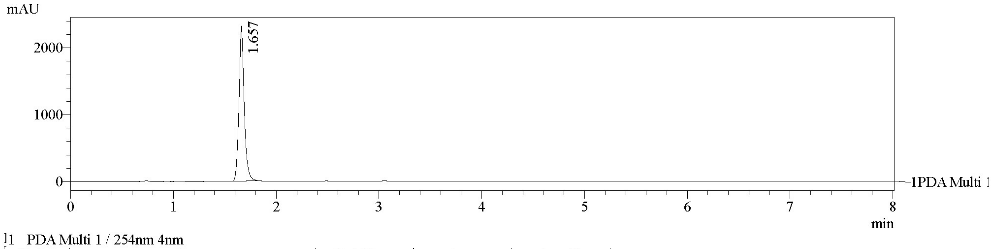

1 PDA Multi 1 / 254nm 4nm

Peak#	Name	Ret. Time	Area	Area %
1		1.657	8181085	100.000

MS Chromatogram
YAZH01-126-1 C:\LabSolutions\Data\2017\2017-wk32\YAZH01-126-1.lcd

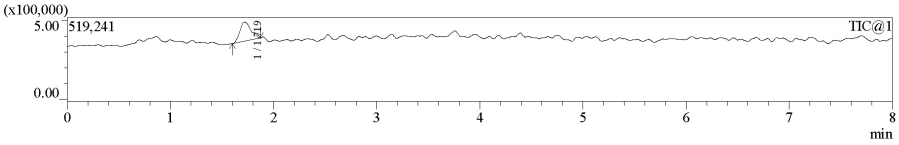

MS Spectrum Graph
#:1 Ret.Time:Averaged 1.710-1.730(Scan#:172-174)
Mass Peaks:34 Base Peak:268.00(47172) Polarity:Neg Segment1 - Event1

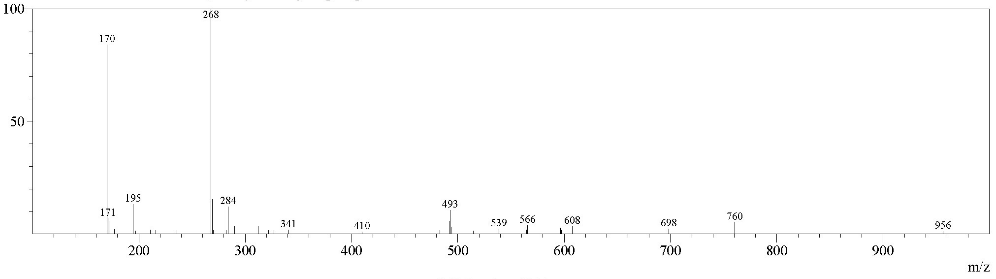

MS Spectrum Table
#:1 Ret.Time:
BG Mode:Calc 1.600<->1.870(161<->188)
Mass Peaks:34 Base Peak:268.00(47172) Polarity:Neg Segment1 - Event1

#	m/z	Abs.Inten.	Rel.Inten.	Charge	Polarity	Monoisotopic
1	169.95	39630	84.01			
2	171.00	3308	7.01			
3	172.00	2757	5.84			
4	177.00	942	2.00			
5	194.85	6203	13.15			
6	196.85	633	1.34			
7	210.95	915	1.94			
8	215.95	793	1.68			
9	235.80	770	1.63			
10	268.00	47172	100.00			
11	269.05	7198	15.26			
12	270.05	811	1.72			
13	282.10	728	1.54			
14	284.00	5680	12.04			
15	290.20	1608	3.41			
16	312.05	1592	3.37			

Figure S117. LCMS spectrum of compound **43a**.

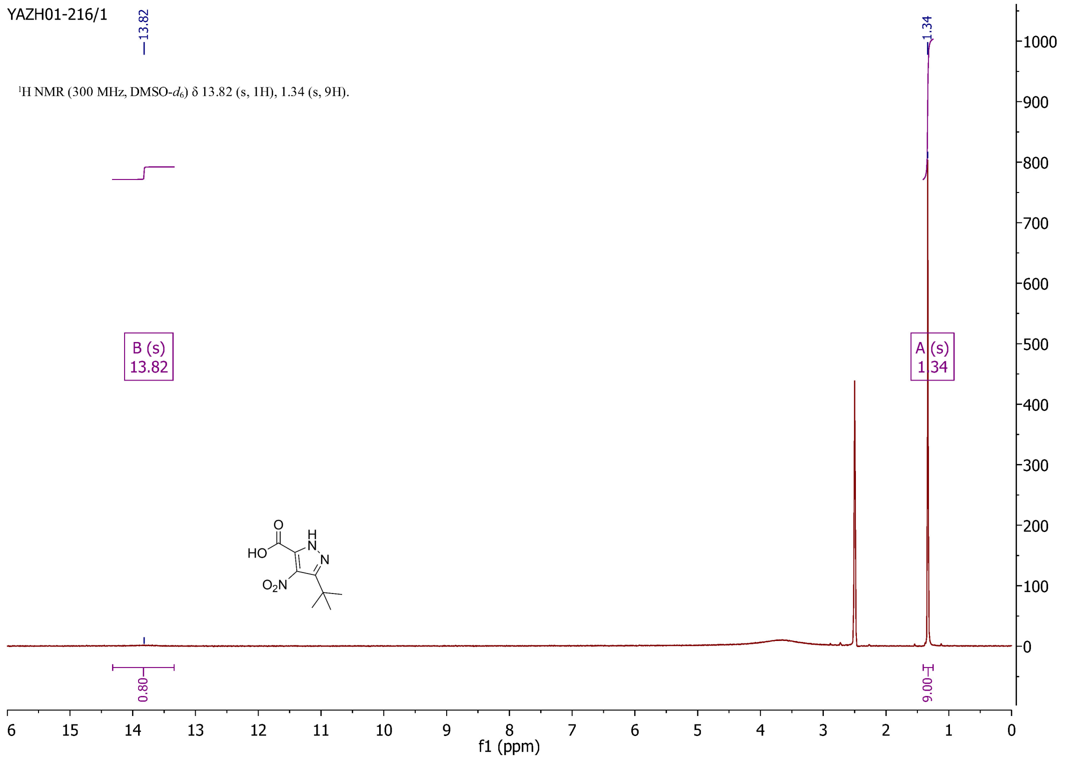

Figure S118. ^{1}H NMR spectrum of compound **43a**.

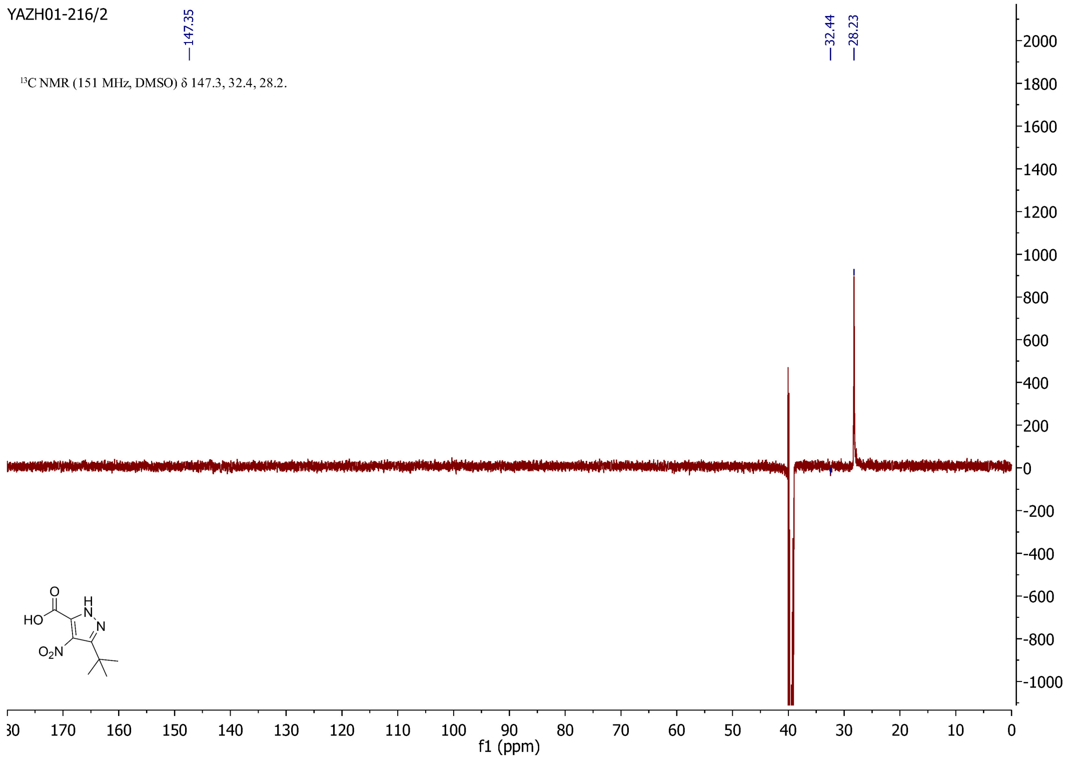

Figure S119. ^{13}C NMR spectrum of compound **43a**.

Acquired by	: Admin
Date Acquired	: 17/7/2018 3:35:18 PM
Sample Name	: LABA-024-AFTERFILT
Sample ID	:
Tray#	: 1
Vial#	: 31
Injection Volume	: 1
Data File	: C:\LabSolutions\Data\2018\2018-wk29\LABA-024-AFTERFILT.lcd
Background File	: blanco 17072018.lcd
Method File	: Method SCAN ACID standard neg.lcm
Report Format	: DefaultLCMS.lcr
Tuning File	: C:\LabSolutions\Tuning\Tuning-ESI-pos-neg01072015a.lct
Processed by	: Admin
Modified Date	: 17/11/2020 11:01:33 AM

PDA Graph

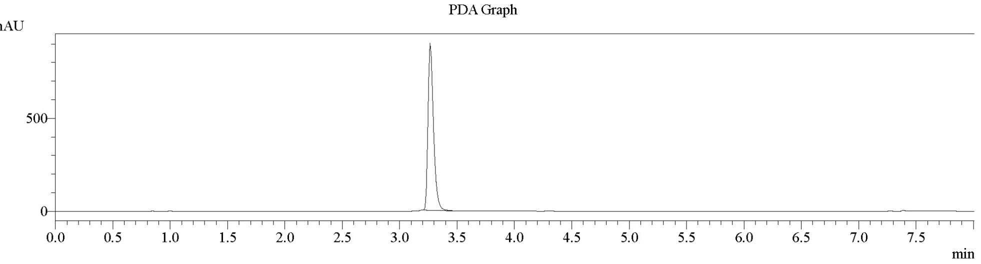

PeakTable

PDA Ch1 254nm 4nm

Peak#	Name	Ret. Time	Area	Area %
1		3.261	2956934	100.000

MS Chromatogram
LABA-024-AFTERFILT C:\LabSolutions\Data\2018\2018-wk21-30\2018-wk29\LABA-024-AFTERFILT.lcd

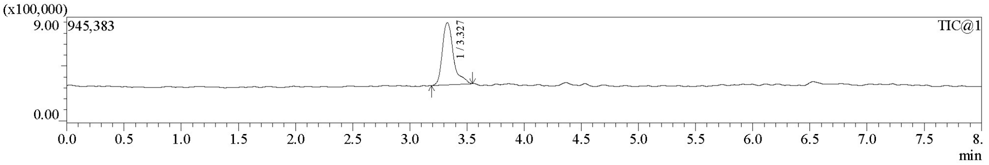

MS Spectrum Graph

#:1 Ret.Time:Averaged 3.320-3.340(Scan#:333-335)
BG Mode:Calc 3.190<->3.550(320<->356)
Mass Peaks:11 Base Peak:224.00(265539) Polarity:Neg Segment1 - Event1

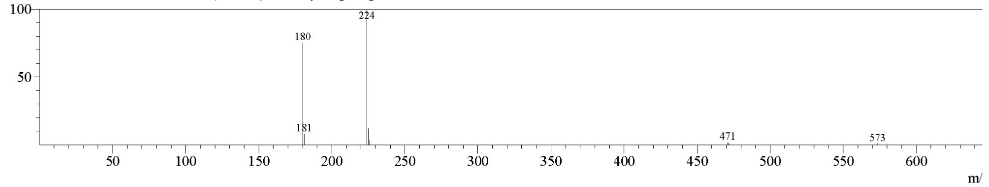

MS Spectrum Table

#:1 Ret.Time:
BG Mode:Calc 3.190<->3.550(320<->356)
Mass Peaks:11 Base Peak:224.00(265539) Polarity:Neg Segment1 - Event1

#	m/z	Abs.Inten.	Rel.Inten.	Charge	Polarity	Monoisotopic
1	180.05	199863	75.27			
2	181.00	20977	7.90			
3	224.00	265539	100.00			
4	225.05	31685	11.93			
5	225.95	8659	3.26			
6	471.20	4857	1.83			

#	m/z	Abs.Inten.	Rel.Inten.	Charge	Polarity	Monoisotopic
7	471.70	3493	1.32			
8	573.50	2672	1.01			
9	712.20	6994	2.63			
10	718.20	4035	1.52			
11	728.20	3693	1.39			

Figure S120. LCMS spectrum of compound **43b**.

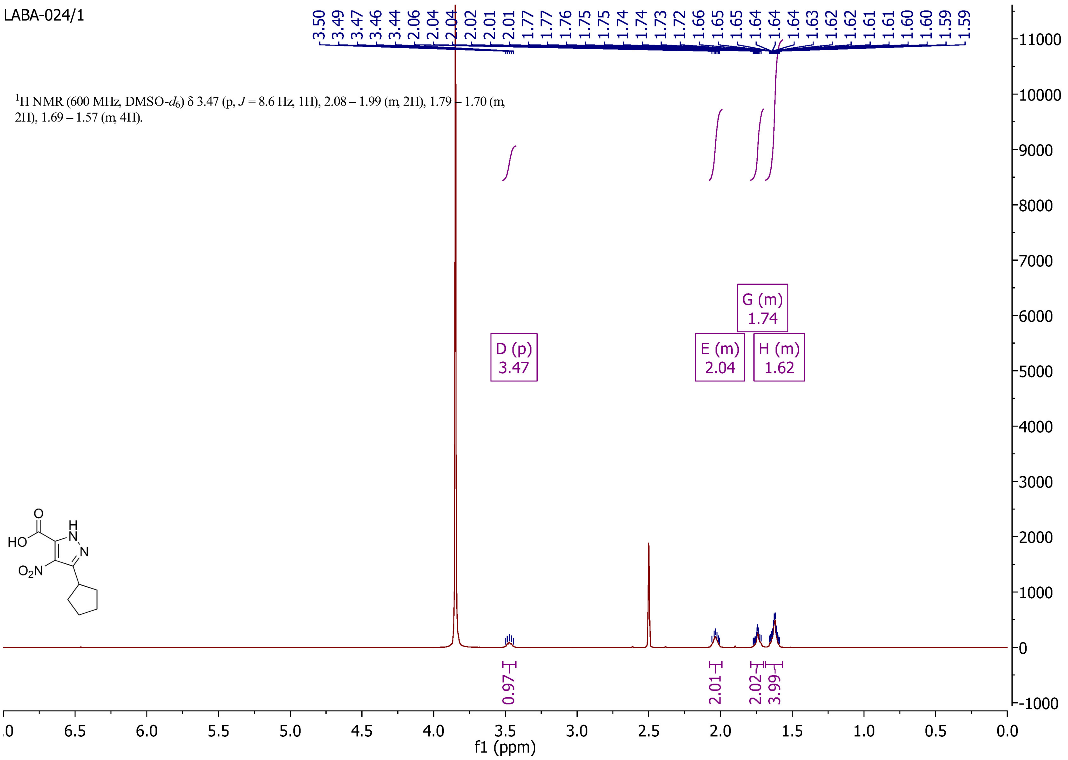

Figure S121. ^{1}H NMR spectrum of compound **43b**.

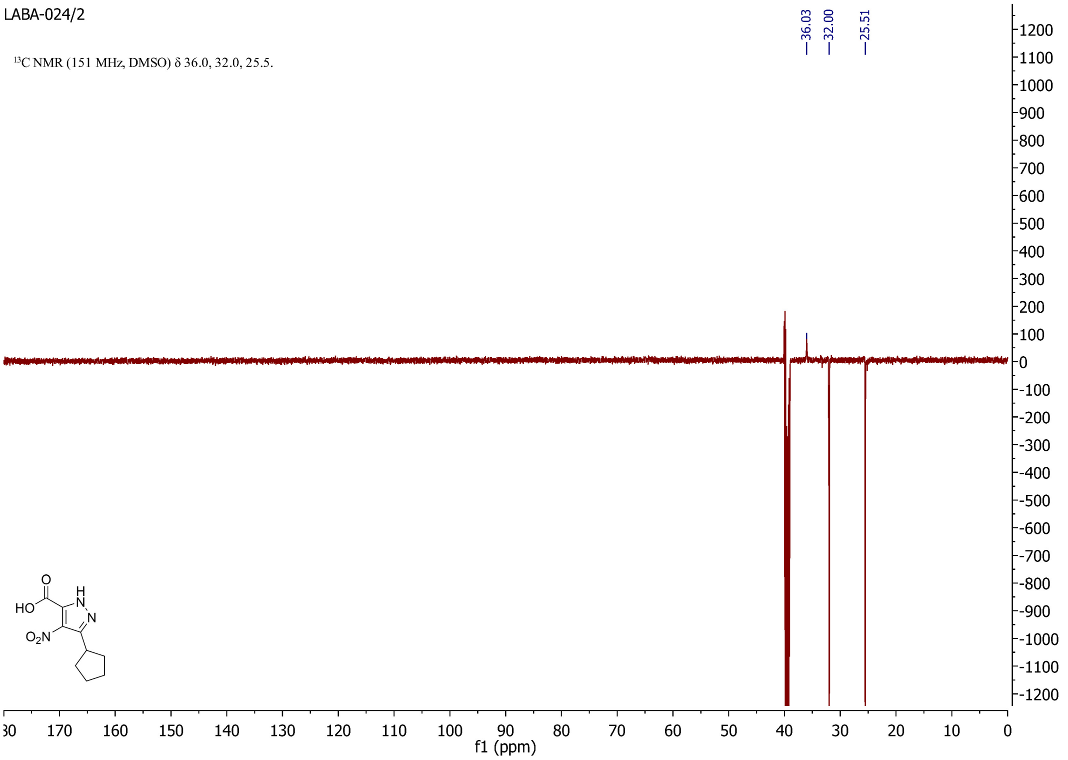

Figure S122. ^{13}C NMR spectrum of compound **43b**.

Acquired by : Admin
Date Acquired : 10/4/2019 12:04:54 PM
Sample Name : YAZH_35
Sample ID :
Tray# : 1
Vial# : 49
Injection Volume : 3
Data File : C:\LabSolutions\Data\2019\2019-wk15\YAZH_35.lcd
Background File : blanco 10042019.lcd
Method File : Method SCAN ACID standard.lcm
Report Format : DefaultLCMS.lcr
Tuning File : C:\LabSolutions\Tuning\Tuning-ESI-pos-neg01072015a.lct
Processed by : Admin
Modified Date : 10/4/2019 12:38:19 PM

PDA Graph

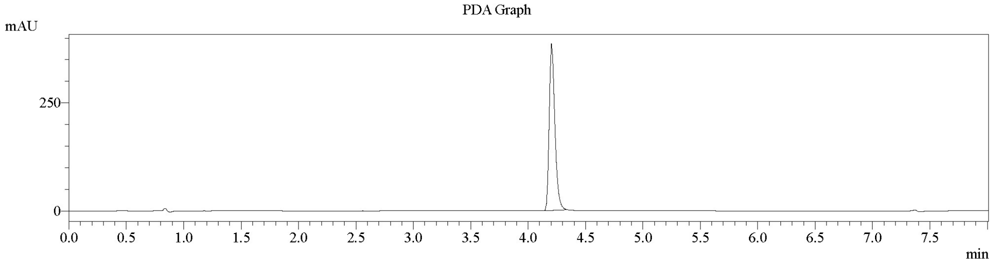

PeakTable

PDA Ch1 254nm 4nm

Peak#	Name	Ret. Time	Area	Area %
1		4.199	1311255	100.000

MS Chromatogram
YAZH_35 C:\LabSolutions\Data\2019\2019-wk15\YAZH_35.lcd

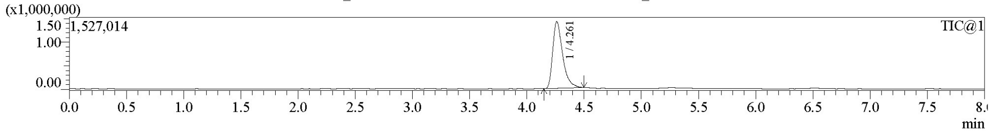

MS Spectrum Graph

#:1 Ret.Time:Averaged 4.250-4.270(Scan#:426-428)
BG Mode:Calc 4.150<->4.500(416<->451)
Mass Peaks:13 Base Peak:283.15(868168) Polarity:Pos Segment1 - Event1

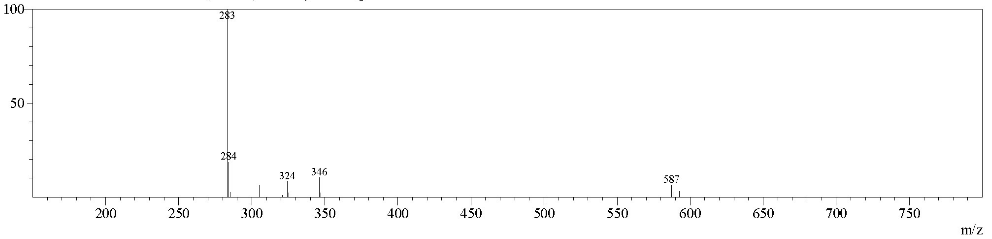

MS Spectrum Table

#:1 Ret.Time:
BG Mode:Calc 4.150<->4.500(416<->451)
Mass Peaks:13 Base Peak:283.15(868168) Polarity:Pos Segment1 - Event1

#	m/z	Abs.Inten.	Rel.Inten.	Charge	Polarity	Monoisotopic
1	283.15	868168	100.00			
2	284.10	160539	18.49			
3	285.10	21276	2.45			
4	305.05	52972	6.10			
5	321.05	8756	1.01			
6	324.15	70833	8.16			
7	325.20	18297	2.11			
8	346.15	88549	10.20			
9	347.15	18860	2.17			
10	587.30	54193	6.24			
11	588.30	23663	2.73			
12	592.50	26807	3.09			
13	592.80	14499	1.67			

Figure S123. LCMS spectrum of compound **46a**.

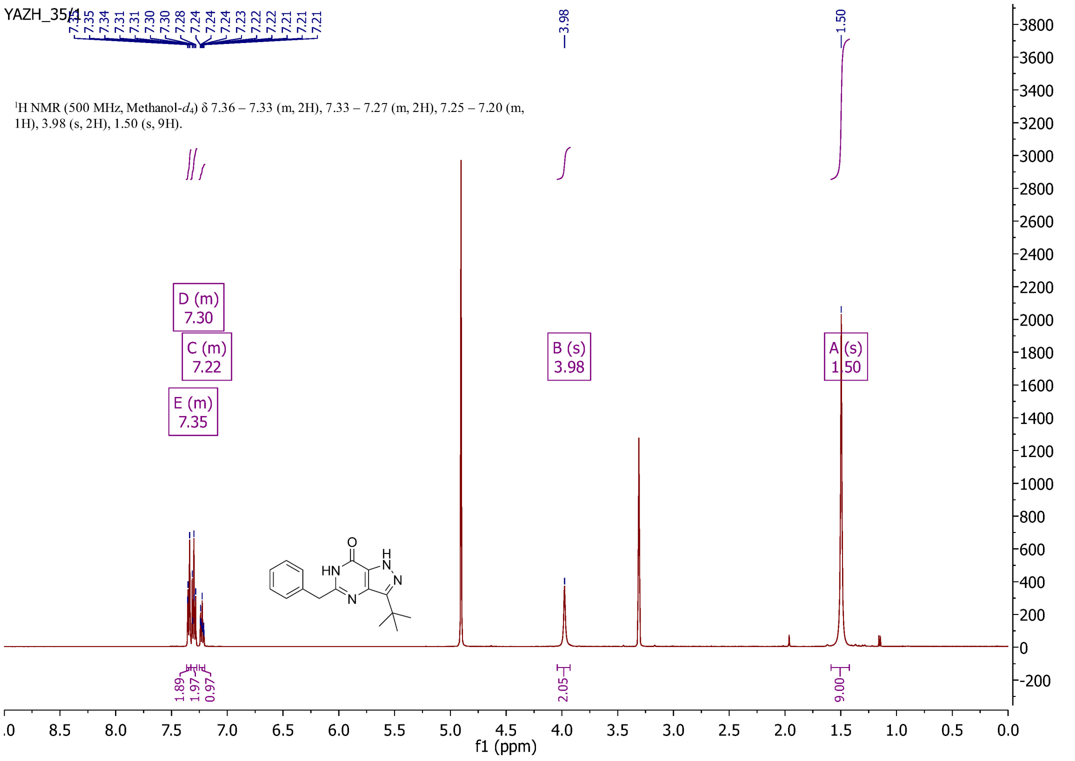

Figure S124. ^{1}H NMR spectrum of compound **46a**.

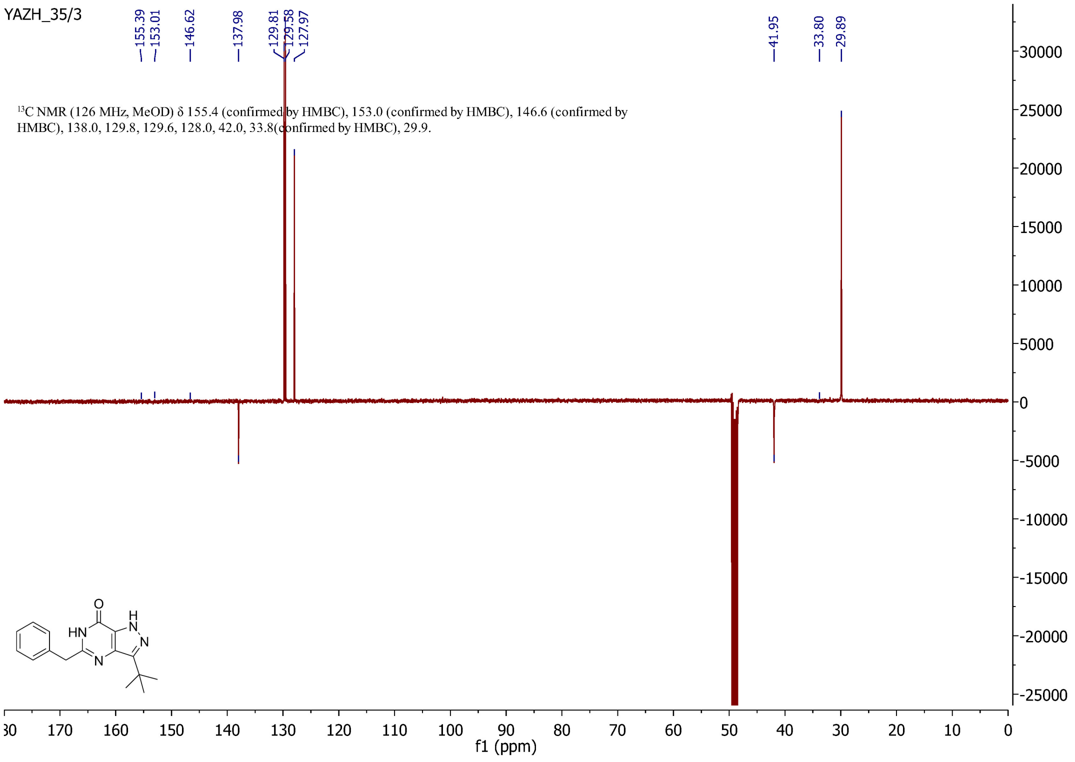

Figure S125. ^{13}C NMR spectrum of compound **46a**.

Acquired by	: Admin
Date Acquired	: 30/1/2019 10:08:35 AM
Sample Name	: YAZH01-294-1
Sample ID	:
Tray#	: 1
Vial#	: 2
Injection Volume	: 1
Data File	: C:\LabSolutions\Data\2019\2019-wk04\YAZH01-294-1.lcd
Background File	: blanco30012019.lcd
Method File	: Method SCAN ACID standard.lcm
Report Format	: DefaultLCMS.lcr
Tuning File	: C:\LabSolutions\Tuning\Tuning-ESI-pos-neg01072015a.lct
Processed by	: Admin
Modified Date	: 30/1/2019 10:41:09 AM

Chromatogram
YAZH01-294-1 C:\LabSolutions\Data\2019\2019-wk04\YAZH01-294-1.lcd

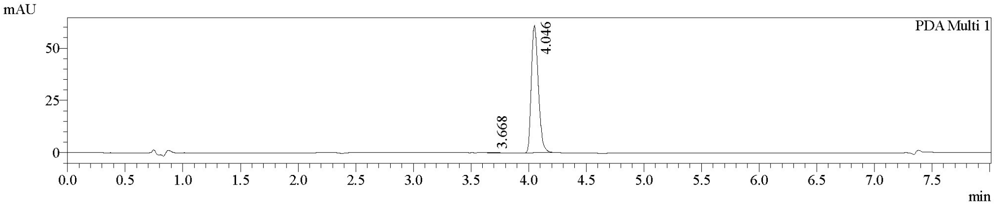

1 PDA Multi 1 / 254nm 4nm

PeakTabl

PDA Ch1 254nm 4nm

Peak#	Name	Ret. Time	Area	Area %
1		3.668	794	0.310
2		4.046	255743	99.690

MS Chromatogram
YAZH01-294-1 C:\LabSolutions\Data\2019\2019-wk04\YAZH01-294-1.lcd

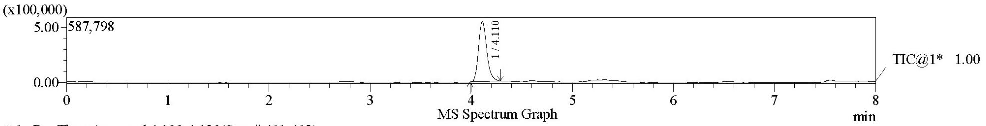

MS Spectrum Graph

#:1 Ret.Time:Averaged 4.100-4.120(Scan#:411-413)
Mass Peaks:10 Base Peak:295.10(390432) Polarity:Pos Segment1 - Event1

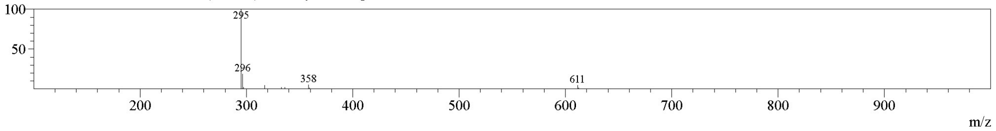

MS Spectrum Table

#:1 Ret.Time:
BG Mode:Calc 3.990<->4.290(400<->430)
Mass Peaks:10 Base Peak:295.10(390432) Polarity:Pos Segment1 - Event1

#	m/z	Abs.Inten.	Rel.Inten.	Charge	Polarity	Monoisotopic
1	295.10	390432	100.00			
2	296.15	73812	18.91			
3	297.10	7165	1.84			
4	317.10	17514	4.49			
5	333.00	7485	1.92			
6	336.10	9158	2.35			
7	358.15	20482	5.25			
8	359.15	4901	1.26			
9	611.25	17194	4.40			
10	612.25	5322	1.36			

Figure S126. LCMS spectrum of compound **46b**.

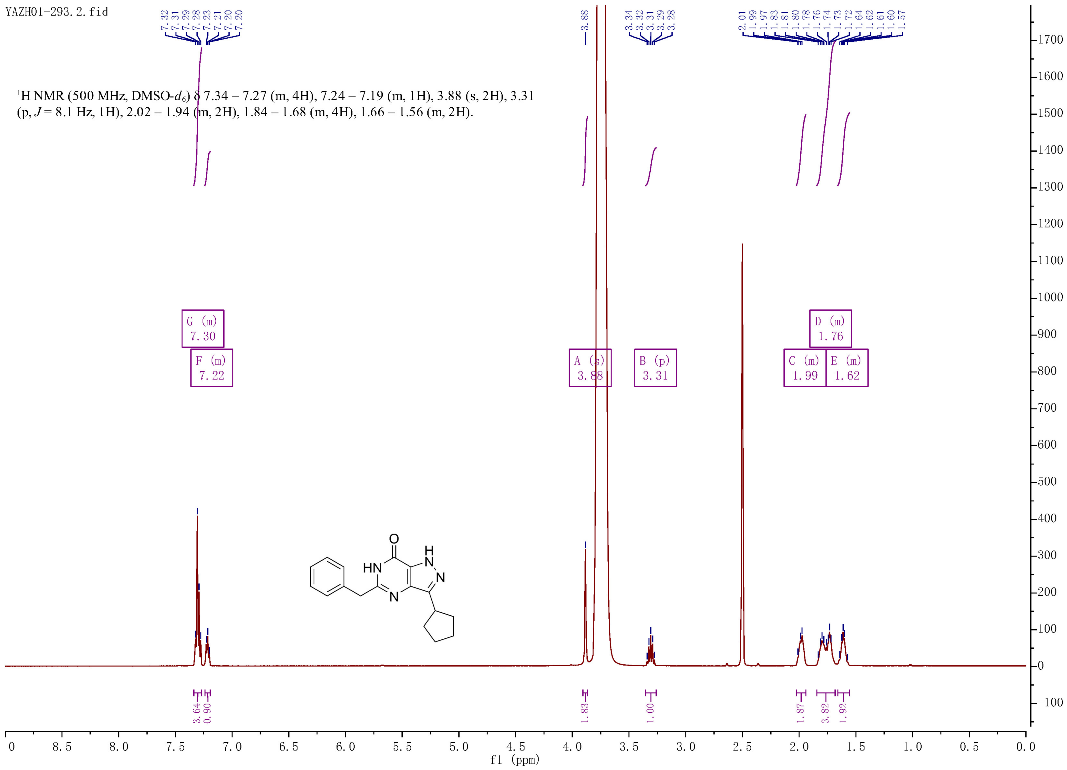

Figure S127. ^{1}H NMR spectrum of compound **46b**.

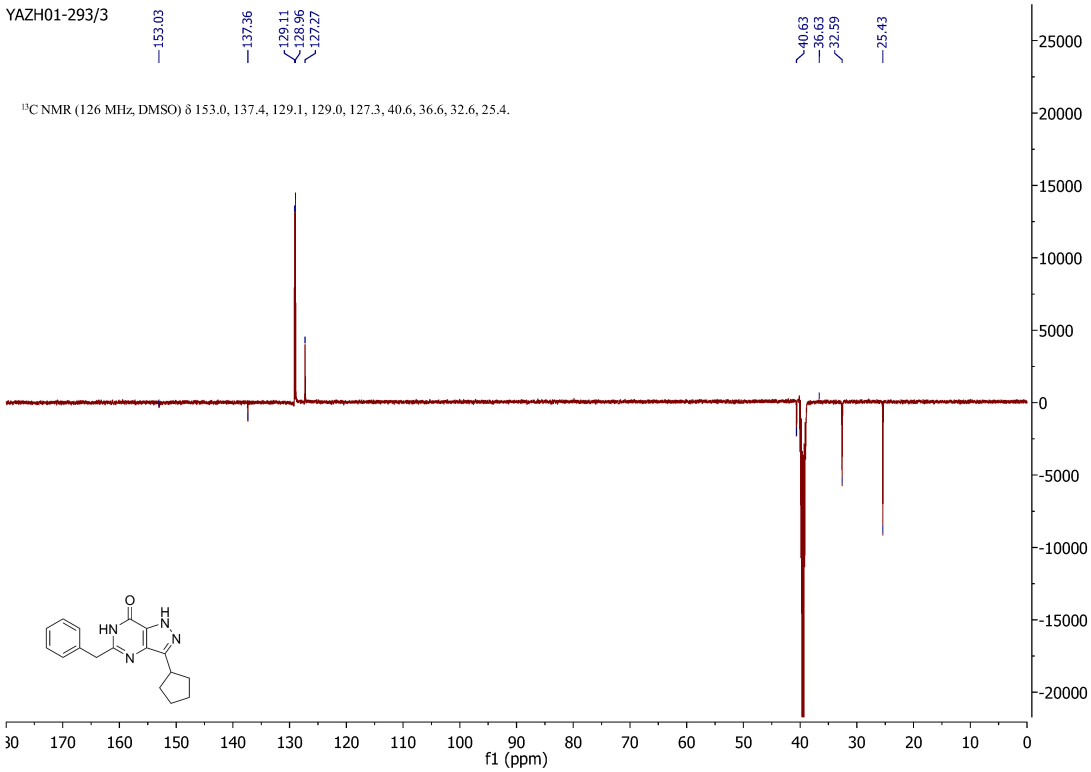

Figure S128. ^{13}C NMR spectrum of compound **46b**.

Acquired by	: Admin
Date Acquired	: 1/4/2019 10:29:47 AM
Sample Name	: YAZH_36
Sample ID	:
Tray#	: 1
Vial#	: 6
Injection Volume	: 3
Data File	: C:\LabSolutions\Data\2019\2019-wk14\YAZH_36.lcd
Background File	: blanco 01042019.lcd
Method File	: Method SCAN ACID standard.lcm
Report Format	: DefaultLCMS.lcr
Tuning File	: C:\LabSolutions\Tuning\Tuning-ESI-pos-neg01072015a.lct
Processed by	: Admin
Modified Date	: 1/4/2019 10:46:16 AM

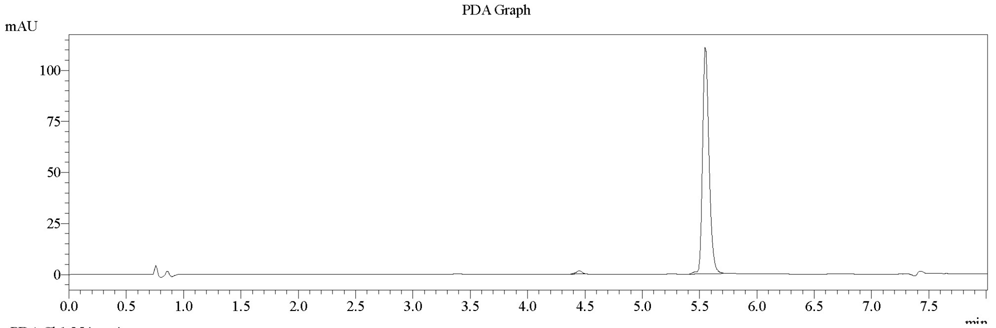

PDA Ch1 254nm 4nm

Peak#	Name	Ret. Time	Area	Area %
1		4.445	5355	1.223
2		5.546	432555	98.777

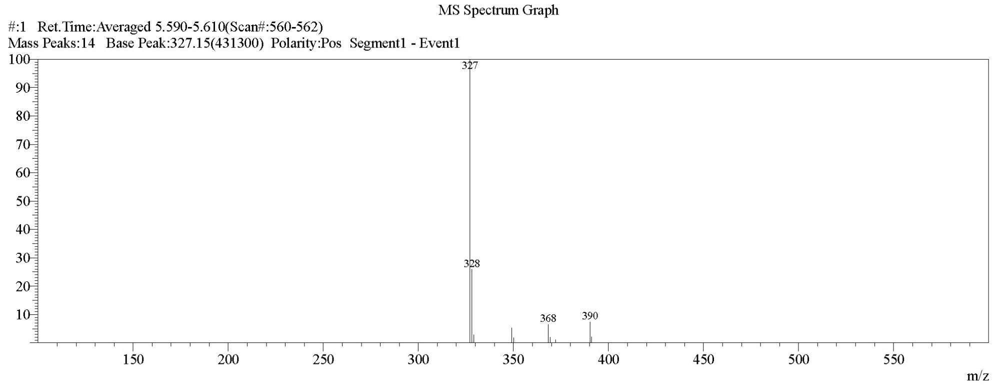

MS Spectrum Table

#:1 Ret.Time:
BG Mode:Calc 5.470<->5.800(548<->581)
Mass Peaks:14 Base Peak:327.15(431300) Polarity:Pos Segment1 - Event1

#	m/z	Abs.Inten.	Rel.Inten.	Charge	Polarity	Monoisotopic
1	327.15	431300	100.00			
2	328.15	111634	25.88			
3	329.15	11967	2.77			
4	349.15	22488	5.21			
5	350.20	7721	1.79			
6	368.25	28144	6.53			
7	369.25	8672	2.01			
8	372.15	4997	1.16			
9	390.20	32076	7.44			
10	391.15	9329	2.16			
11	675.45	12626	2.93			
12	680.40	7078	1.64			
13	681.05	8122	1.88			
14	681.90	6035	1.40			

Figure S129. LCMS spectrum of compound **47a**.

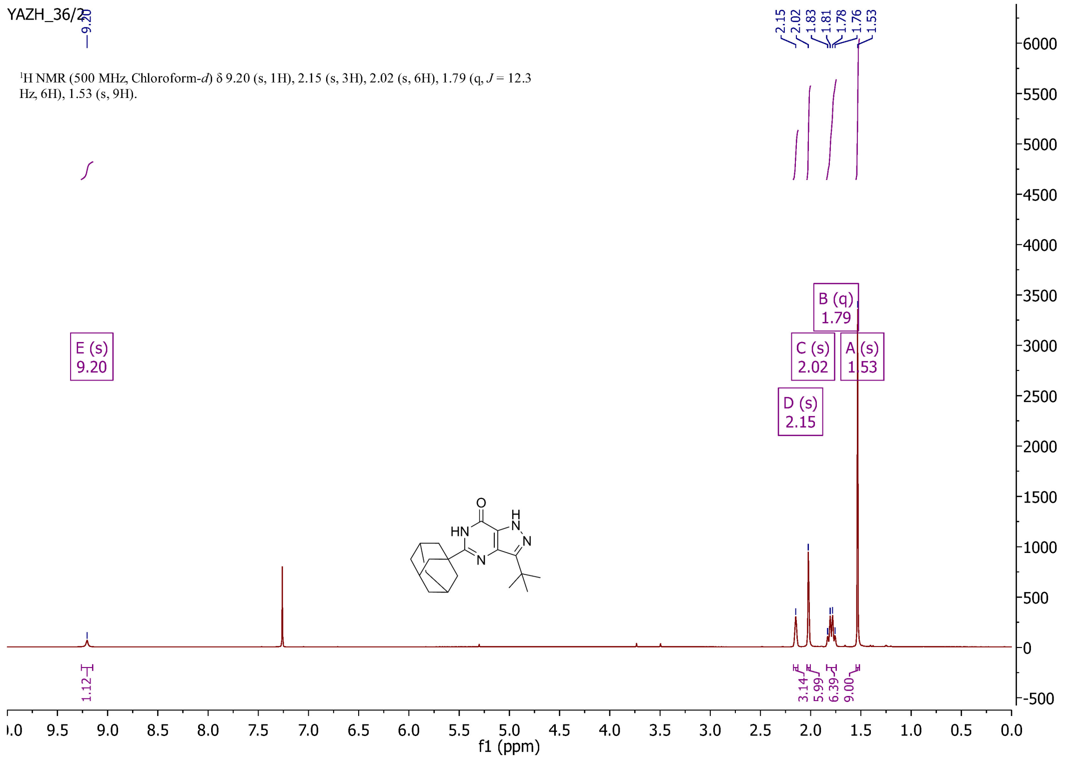

Figure S130. ^{1}H NMR spectrum of compound **47a**.

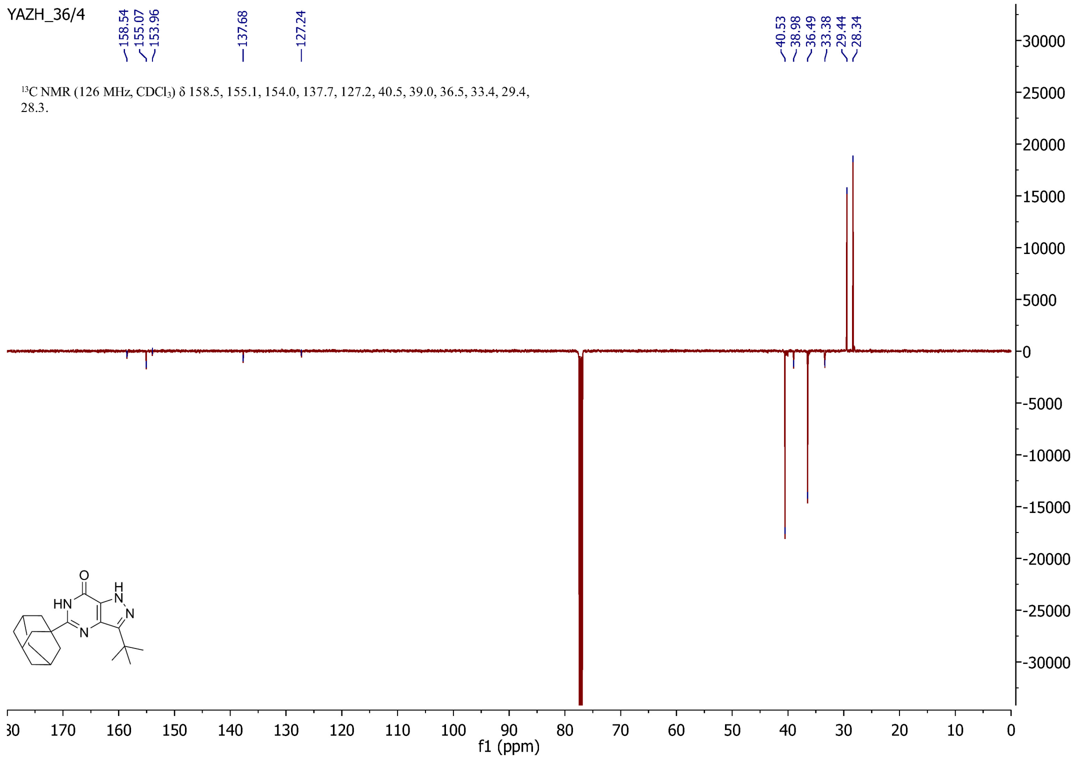

Figure S131. ^{13}C NMR spectrum of compound **47a**.

Acquired by	: Admin
Date Acquired	: 1/4/2019 10:55:50 AM
Sample Name	: YAZH_38
Sample ID	:
Tray#	: 1
Vial#	: 7
Injection Volume	: 3
Data File	: C:\LabSolutions\Data\2019\2019-wk14\YAZH_38.lcd
Background File	: blanco 01042019.lcd
Method File	: Method SCAN ACID standard.lcm
Report Format	: DefaultLCMS.lcr
Tuning File	: C:\LabSolutions\Tuning\Tuning-ESI-pos-neg01072015a.lct
Processed by	: Admin
Modified Date	: 1/4/2019 11:32:33 AM

PDA Graph

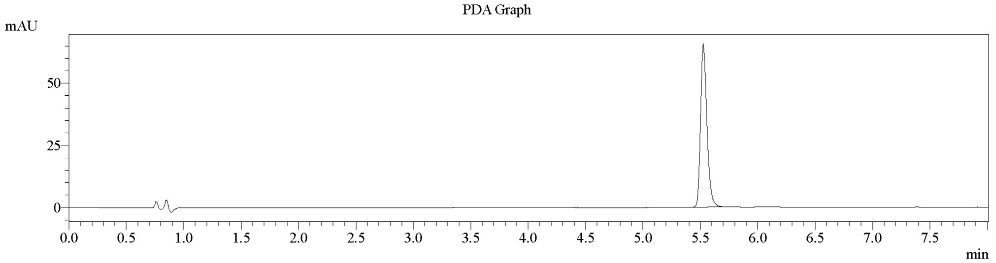

PeakTable

PDA Ch1 254nm 4nm

Peak#	Name	Ret. Time	Area	Area %
1		5.521	254356	100.000

MS Chromatogram
YAZH_38 C:\LabSolutions\Data\2019\2019-wk14\YAZH_38.lcd

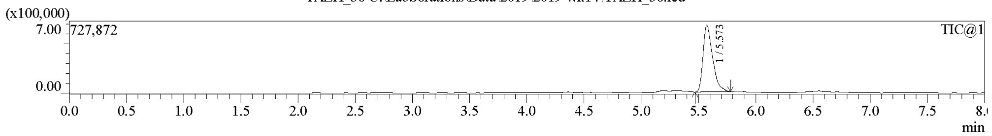

MS Spectrum Graph

#:1 Ret.Time:Averaged 5.560-5.580(Scan#:557-559)
BG Mode:Calc 5.470<->5.780(548<->579)
Mass Peaks:12 Base Peak:339.15(483960) Polarity:Pos Segment1 - Event1

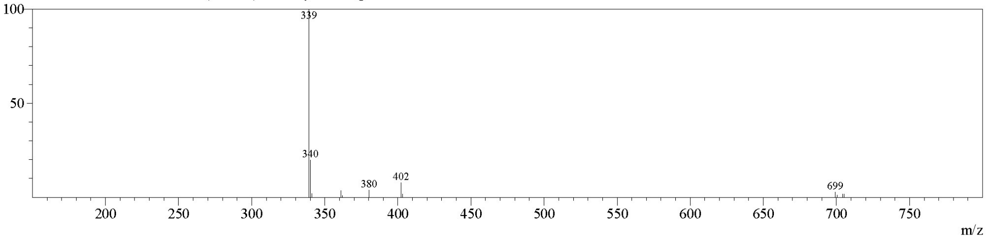

MS Spectrum Table

#:1 Ret.Time:
BG Mode:Calc 5.470<->5.780(548<->579)
Mass Peaks:12 Base Peak:339.15(483960) Polarity:Pos Segment1 - Event1

#	m/z	Abs.Inten.	Rel.Inten.	Charge	Polarity	Monoisotopic
1	339.15	483960	100.00			
2	340.20	96147	19.87			
3	341.30	9476	1.96			
4	361.10	17293	3.57			
5	362.05	4905	1.01			
6	380.25	18775	3.88			

#	m/z	Abs.Inten.	Rel.Inten.	Charge	Polarity	Monoisotopic
7	402.15	37272	7.70			
8	403.20	8603	1.78			
9	699.35	13443	2.78			
10	700.40	6162	1.27			
11	704.40	7587	1.57			
12	705.15	7469	1.54			

Figure S132. LCMS spectrum of compound **47b**.

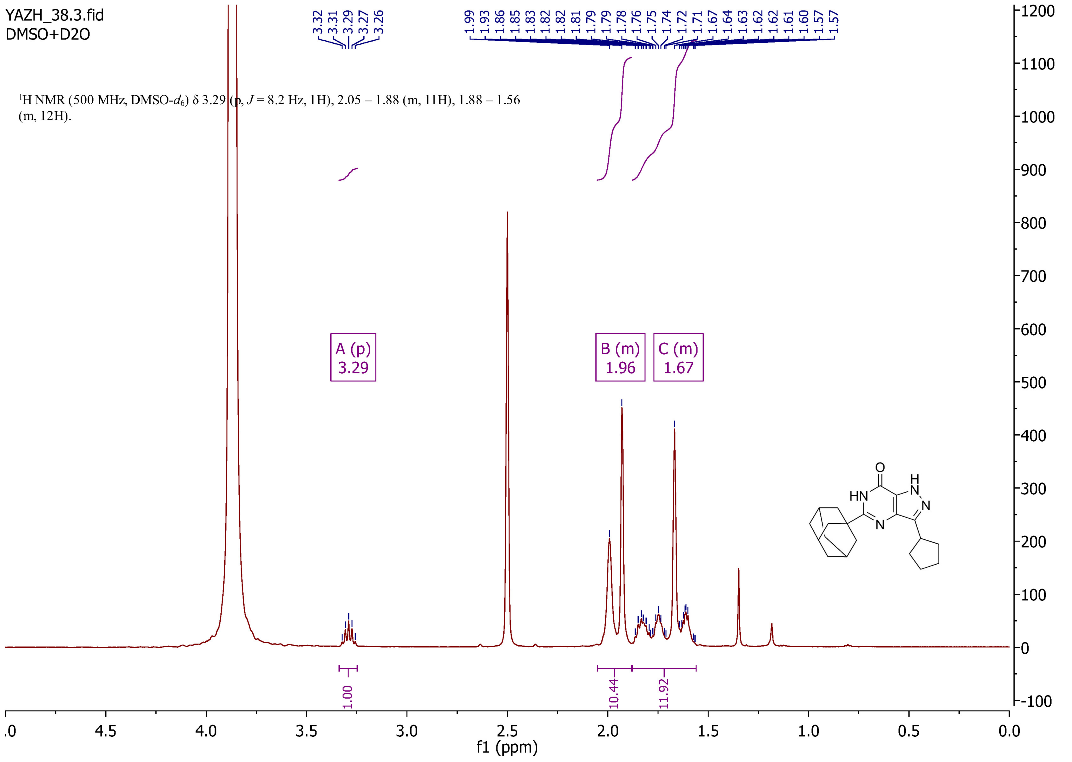

Figure S133. ^{1}H NMR spectrum of compound **47b**.

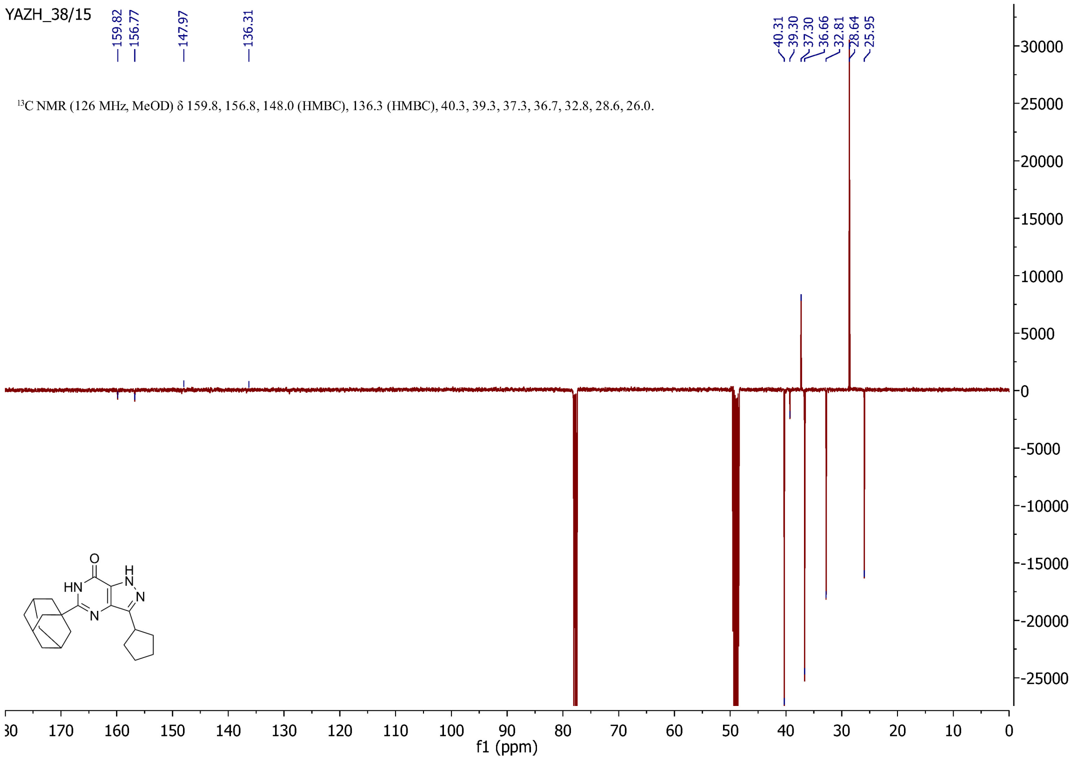

Figure S134. ^{13}C NMR spectrum of compound **47b**.
